# Tailored Tolane‐Perfluorotolane Assembly as Supramolecular Base Pair Replacement in DNA

**DOI:** 10.1002/anie.202214456

**Published:** 2022-12-01

**Authors:** Hermann Neitz, Irene Bessi, Valentin Kachler, Manuela Michel, Claudia Höbartner

**Affiliations:** ^1^ Institute of Organic Chemistry University of Würzburg Am Hubland 97074 Würzburg Germany; ^2^ Center for Nanosystems Chemistry (CNC) University of Würzburg Theodor-Boveri-Weg 97074 Würzburg Germany

**Keywords:** Arene-Fluoroarene, Artificial Base Pair, DNA, Supramolecular Interaction, XNA

## Abstract

Arene‐fluoroarene interactions offer outstanding possibilities for engineering of supramolecular systems, including nucleic acids. Here, we implement the tolane‐perfluorotolane interaction as base pair replacement in DNA. Tolane (THH) and perfluorotolane (TFF) moieties were connected to acyclic backbone units, comprising glycol nucleic acid (GNA) or butyl nucleic acid (BuNA) building blocks, that were incorporated via phosphoramidite chemistry at opposite positions in a DNA duplex. Thermodynamic analyses by UV thermal melting revealed a compelling stabilization by THH/TFF heteropairs only when connected to the BuNA backbone, but not with the shorter GNA linker. Detailed NMR studies confirmed the preference of the BuNA backbone for enhanced polar π‐stacking. This work defines how orthogonal supramolecular interactions can be tailored by small constitutional changes in the DNA backbone, and it inspires future studies of arene‐fluoroarene‐programmed assembly of DNA.

## Graphical abstract

Polar π‐interactions and preferential stacking were observed by attaching tolane and perfluorotolanes as nucleobase surrogates to acyclic backbone replacements in an otherwise unmodified DNA duplex. The butyl nucleic acid (BuNA) was identified as privileged scaffold for exploiting the supramolecular arene‐fluoroarene recognition in DNA.

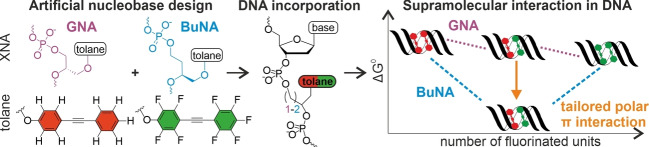

Noncovalent interactions are crucial in chemical and biological systems for driving structural organization as well as for controlling molecular recognition. Xeno nucleic acids (XNA) with artificial backbones^[1]^ and unnatural base pairs (UBPs)^[2]^ that exploit alternative hydrogen bonding patterns,^[3]^ metal coordination^[4]^ or hydrophobic interactions^[5]^ are heavily investigated for applications in nucleic acid nanotechnology as well as chemical and synthetic biology.^[6]^ Examples reported by Leumann,^[7]^ Häner,^[8]^ Iverson,^[9]^ and Asanuma^[10]^ used electron‐rich and electron‐deficient aromatic systems to control the assembly of DNA strands. In contrast to the majority of known unnatural base pairs, which rely on hydrogen bonding or hydrophobic shape complementarity, in this work we exploit the stabilizing electrostatic interaction of opposite‐sign quadrupole moments in arenes and fluoroarenes, which has previously been harnessed for the controlled assembly of organic materials,^[11]^ polymers,^[12]^ and crystals.^[13]^ Using arene‐fluoroarene interactions in aqueous solution is challenging due to the weak affinity and the hydrophobic effect, which can lead to uncontrolled aggregation.^[14]^ Nevertheless, several supramolecular systems using arene‐fluoroarene interactions in water have been reported,^[15]^ including peptides^[16]^ and nucleic acids. In DNA, Kool,^[17]^ Hunziker,^[18]^ Leumann^[19]^ and others explored various fluorinated analogues, in which observed stabilizations were mostly of entropic origin due to the increased hydrophobicity of the fluorinated compound. Alternative structural scaffolds are therefore needed to fully exploit the potential of opposite‐sign quadrupole moments and enhanced electrostatic interactions.^[20]^ Thus, the construction of an artificial arene‐fluoroarene based recognition element in DNA that integrates well in the DNA double helix remained to be explored.

Here, we combine acyclic XNA backbones with aromatic and fluorinated aromatic hydrocarbons and report a bioorthogonal supramolecular recognition motif that serves as a base pair replacement in DNA. We chose the tolane (diphenylacetylene) moiety (THH, Figure 1A) and its perfluorinated analogue (TFF, Figure 1B) as substitutes for Watson–Crick base pairs. The length of the tolane unit provides an excellent fit to the diameter of a DNA double helix,^[21]^ and the phosphodiester linkage can be tailored for optimizing the stacking geometry. We compared glycol nucleic acid (GNA, Figure 1C) with butyl nucleic acid (BuNA, Figure 1D) units connected to the tolane/perfluorotolane. GNA contains a 1,2‐propanediol phosphodiester backbone^[22]^ and formally represents an acyclic version of the threofuranosyl nucleic acid (TNA). Butyl nucleic acid (BuNA)^[23]^ was introduced as an acyclic mimic of the ribose backbone and contains one additional methylene unit in the linker compared to GNA. To comprehensively evaluate the arene‐fluoroarene interactions in the DNA context, the XNA tolane units were placed within the synthetic DNA dodecamer duplex shown in Figure 1E at position X7 and Y18 (Table 1 and S1).

**Figure 1:**
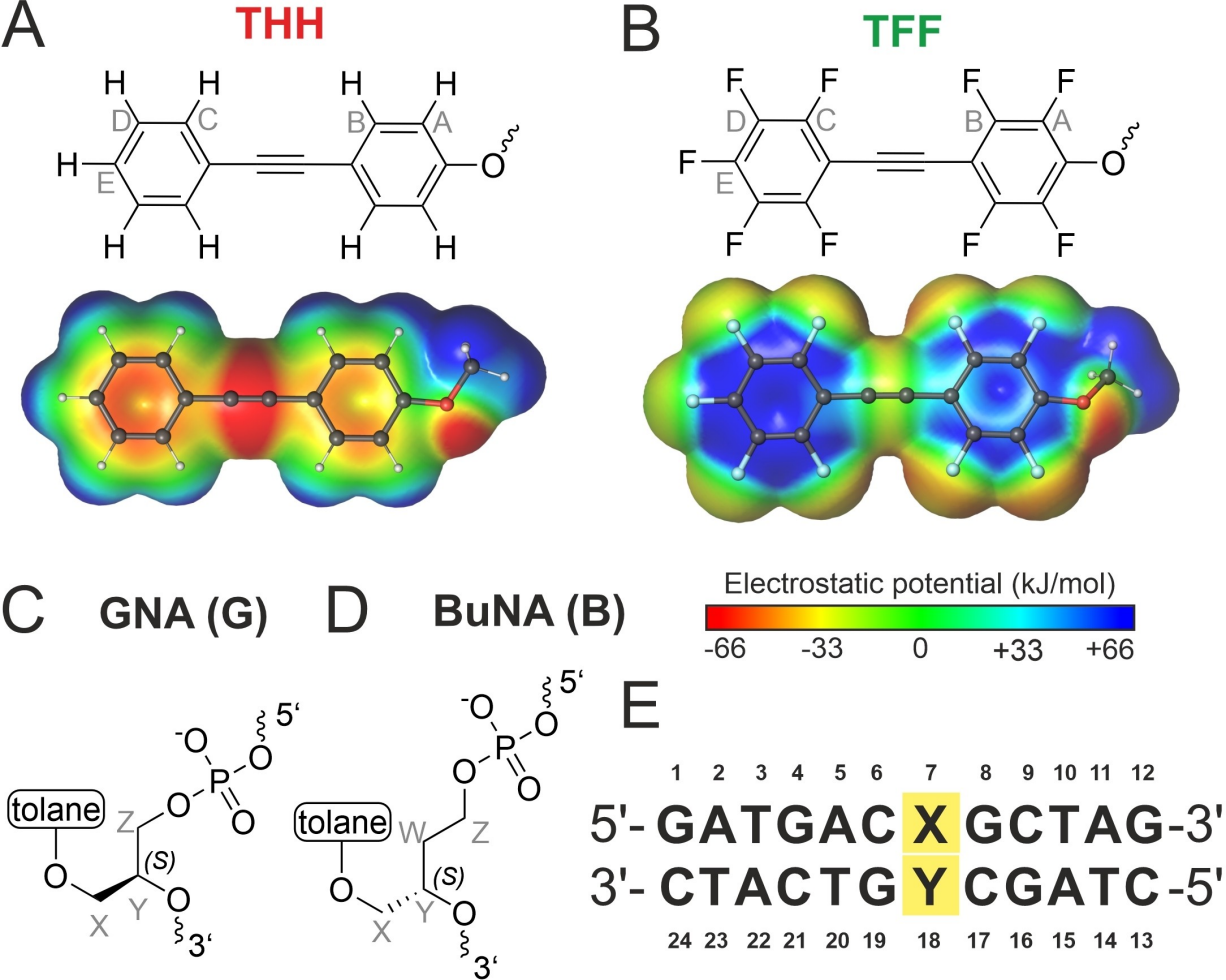
Structure of A) tolane (THH) and B) perfluorotolane (TFF) ether, with respective electrostatic potential surface. Structure of the acyclic backbones C) GNA and D) BuNA. Nomenclature for NMR assignment is given in grey letters. E) Sequence of the DNA duplex used in this study, modified position X7 and Y18 highlighted in yellow.

**Table 1:** Melting temperatures and thermodynamic parameters of DNA duplexes shown in Figure 1E containing THH and TFF homo‐ and hetero‐pair combinations.

Name^[a]^ X7/Y18	*T*_m_^[b]^ [°C] (Δ*T*_m_^[c]^)	Δ*H* ^0[d]^ [kcal mol^−1^]	Δ*S* ^0[d]^ [cal/(mol K)]	Δ*G* ^298[e]^ [kcal mol^−1^]
Ref DNA T/A	44.8 (0.0)	−92.9±1.5	−263±4.3	−14.5±2.0
GTHH/GTHH	40.7 (−4.1)	−74.5±0.6	−209±1.6	−12.2+0.7
GTHH/GTFF	42.4 (−2.4)	−69.6±0.4	−192±1.2	−12.3±0.6
GTFF/GTHH	43.5 (−1.3)	−73.3±0.5	−203±1.3	−12.9±0.6
GTFF/GTFF	44.8 (0.0)	−68.5±0.5	−187±1.6	−12.8±0.8
BTHH/BTHH	42.0 (−2.8)	−76.7±0.7	−214±2.1	−12.8±1.0
BTHH/BTFF	46.9 (+2.1)	−81.8±1.1	−227±3.2	−14.1±1.5
BTFF/BTHH	48.1 (+3.3)	−83.3±1.2	−231±3.3	−14.6±1.5
BTFF/BTFF	45.2 (+0.4)	−71.7±0.8	−197±2.3	−13.1±1.1

[a] G (GNA), B (BuNA), [b] *T*_m_ at 1 μM DNA, in 100 mM NaCl, 10 mM phosphate buffer, pH 7.0. [c] Difference to *T*_m_ of reference. [d] Derived from van't Hoff analyses with five concentrations (1, 2, 5, 10, 20 μM; Table S2). [e] Δ*G* ^298^=Δ*H* ^0^−*T*Δ*S* ^0^. Calculated at 25 °C.

Phosphoramidites (PA) of the THH building blocks were prepared from 4‐(2‐phenylethynyl)phenol (**1**) (Scheme 1). The GNA analogue GTHH was synthesized via a base‐catalyzed ring‐opening reaction of DMT‐protected (*R*)‐glycidol (**2**). The BuNA variant BTHH was obtained via a Mitsunobu reaction with 4‐(*S*)‐hydroxymethyl‐2‐phenyl‐1,3‐dioxan (**3**). The syntheses of the fluorotolanes BTFF and GTFF started with the nucleophilic aromatic substitution of iodopentafluorobenzene with **3** or D‐((+))‐solketal (**4**), followed by two Sonogashira reactions to install the acetylene unit and attach the second pentafluorophenyl ring. The building blocks were transformed to the DMT‐protected 2‐cyanoethyl *N*,*N*‐diisopropyl phosphoramidites, which were used for solid‐phase DNA synthesis (analytical anion exchange HPLC and HR‐ESI data in Supporting Information).

**Scheme 1:**
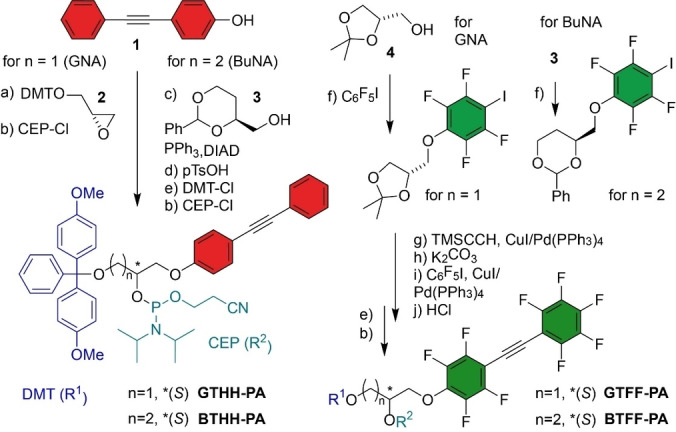
Synthesis of phosphoramidite building blocks. DIAD=diisopropyl azodicarboxylate, DMT‐Cl=4,4′‐dimethoxytrityl chloride, CEP‐Cl=2‐cyanoethyl *N*,*N*‐diisopropyl chlorophosphoramidite. For details see Schemes S1 and S2.

The thermodynamic stability of the tolane‐containing DNA duplexes was characterized by concentration‐dependent UV thermal melting experiments (Figure 2A–B and Table 1). The tolane homopair GTHH/GTHH destabilized the duplex by 4.1 °C compared to a T/A base pair at positions X7/Y18. In contrast, the duplex containing the perfluorinated homopair GTFF/GTFF had the same melting temperature (*T*_m_) as the Watson–Crick reference duplex. Surprisingly, the *T*_m_ of the duplexes containing GNA heteropairs were in between the *T*_m_ of the two homopairs, suggesting that the connection to the GNA backbone was suboptimal and did not allow favorable arene‐perfluoroarene interactions to occur. Within the BuNA series, the *T_m_* values were higher than for the corresponding GNA‐containing analogs. For the homopairs BTHH/BTHH and BTFF/BTFF, the relative trends in enthalpy and entropy were similar as with the GNA backbone. However, upon incorporation of the arene‐fluoroarene heteropairs the expected stabilization was observed: BTHH/BTFF and BTFF/BTHH showed the highest *T*_m_ in the series with 46.9 °C and 48.1 °C, respectively. This corresponds to a thermal stabilization of >3 °C compared to the Watson–Crick reference duplex, which is also reflected in the enthalpy and entropy values. With Δ*H* ^0^ of −81.8 kcal mol^−1^ for BTHH/BTFF and −83.3 kcal mol^−1^ for BTFF/BTHH, Δ*H* ^0^ was circa 10 kcal mol^−1^ more favorable in the BuNA heteropair series, while the entropic stabilization decreased. As expected, incorporation of a single BTHH or BTFF opposite to a nucleobase showed a destabilization of the duplex (Figure S1).

**Figure 2:**
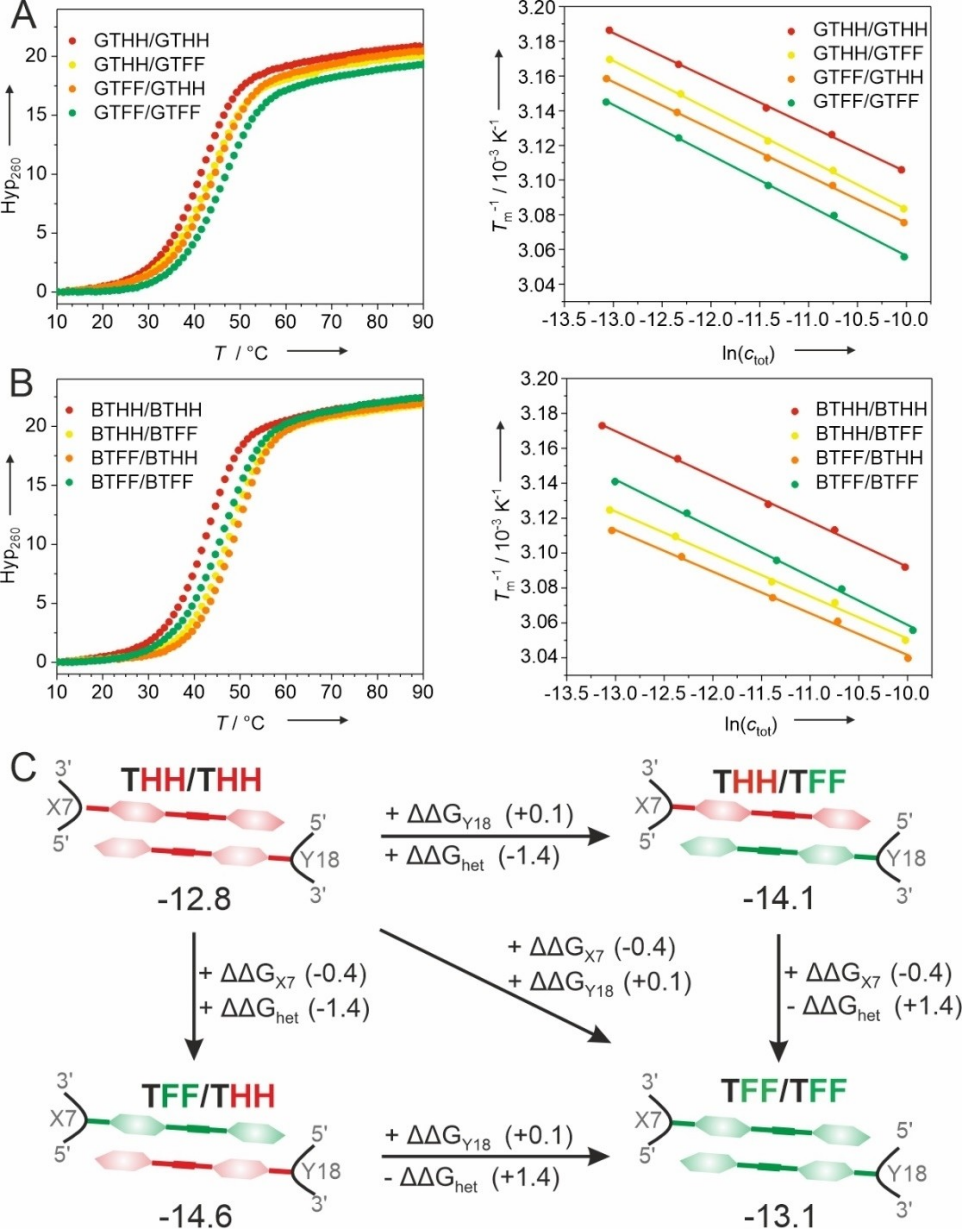
UV‐melting curves (left) and van't Hoff analysis (right) for DNA duplexes containing A) GNA and B) BuNA tolane homopairs and heteropairs. Hyperchromicity at 260 nm, 1 μM DNA in 10 mM sodium phosphate pH 7.0, 100 mM NaCl. C) Schematic representation of the double mutant cycle. ΔΔG values are given for the BuNA series.

Using these data in a chemical double mutant cycle^[24]^ allowed us to disentangle the thermodynamic contributions of the interactions between the tolane units and the neighboring DNA (Figure 2C). Starting from the THH/THH homopair, we focused on the changes of free energy upon fluorination and defined ΔΔG for the tolane units in the heteropair as ΔΔG_het_, and for each fluorinated tolane unit with the neighboring DNA as ΔΔG_X7_ and ΔΔG_Y18_, respectively. The fluorinated tolane at position X7 had a stabilizing effect on the DNA, but not at position Y18: ΔΔG_X7_ (−0.6 kcal mol^−1^ for GNA and −0.4 kcal mol^−1^ for BuNA) in comparison to ΔΔG_Y18_ (0.0 kcal mol^−1^ for GNA or +0.1 kcal mol^−1^ for BuNA). For the GNA variant a negligible ΔΔG_het_ of −0.1 kcal mol^−1^ was found (Figure S2). The gain in stability upon introduction of the BuNA heteropair was significantly larger, with ΔΔG_het_ of −1.4 kcal mol^−1^ (Figure 2C). A similar stabilization was observed in a duplex containing a neighboring A/T base pair instead of G/C (Figure S3). These values are on the same order of magnitude as previously estimated for the contribution of a phenylalanine‐pentafluorophenylalanine interaction in a peptide‐based system.^[20]^ Thus, the thermodynamic analysis shows that the BuNA backbone is the favored connection for the tolane‐fluorotolane heteropair.

FRET‐based DNA strand displacement experiments^[25]^ were designed to investigate the preference for heteropairing in competition with homopairing (Figure 3A, Figure S4,S5 and Table S3). First, an unlabeled strand was added to a Cy3/Cy5 labeled homopair‐containing duplex, resulting in a partial strand displacement. The displacement was tracked by monitoring the increase of the Cy3 fluorescence emission intensity, caused by the reduced FRET to Cy5. Then, an excess of a DNA strand with an unsubstituted propyl linker (C_3_) was added, allowing the determination of the maximal Cy3 fluorescence and the fraction of total displacement (*F*_norm_). The duplexes containing a THH/THH homopair reached a stronger displacement upon addition of a TFF strand than with a THH strand (Figure 3B–C). The BuNA variant showed a higher strand displacement (67.2±4.6 %) than the GNA analog (55.5±2.4 %). The experiments that were performed in the opposite direction, i.e. started with a labeled duplex containing the TFF/TFF homodimer, confirmed this observation. The results in Figure S5 show that in GNA the displacement with a TFF strand was slightly more effective; however, changing to BuNA led to a higher exchange with THH (70.5±2.4 %) than with TFF (55.1±2.2 %). This again confirms that the BuNA backbone is more favorable for the heteropair formation.

**Figure 3:**
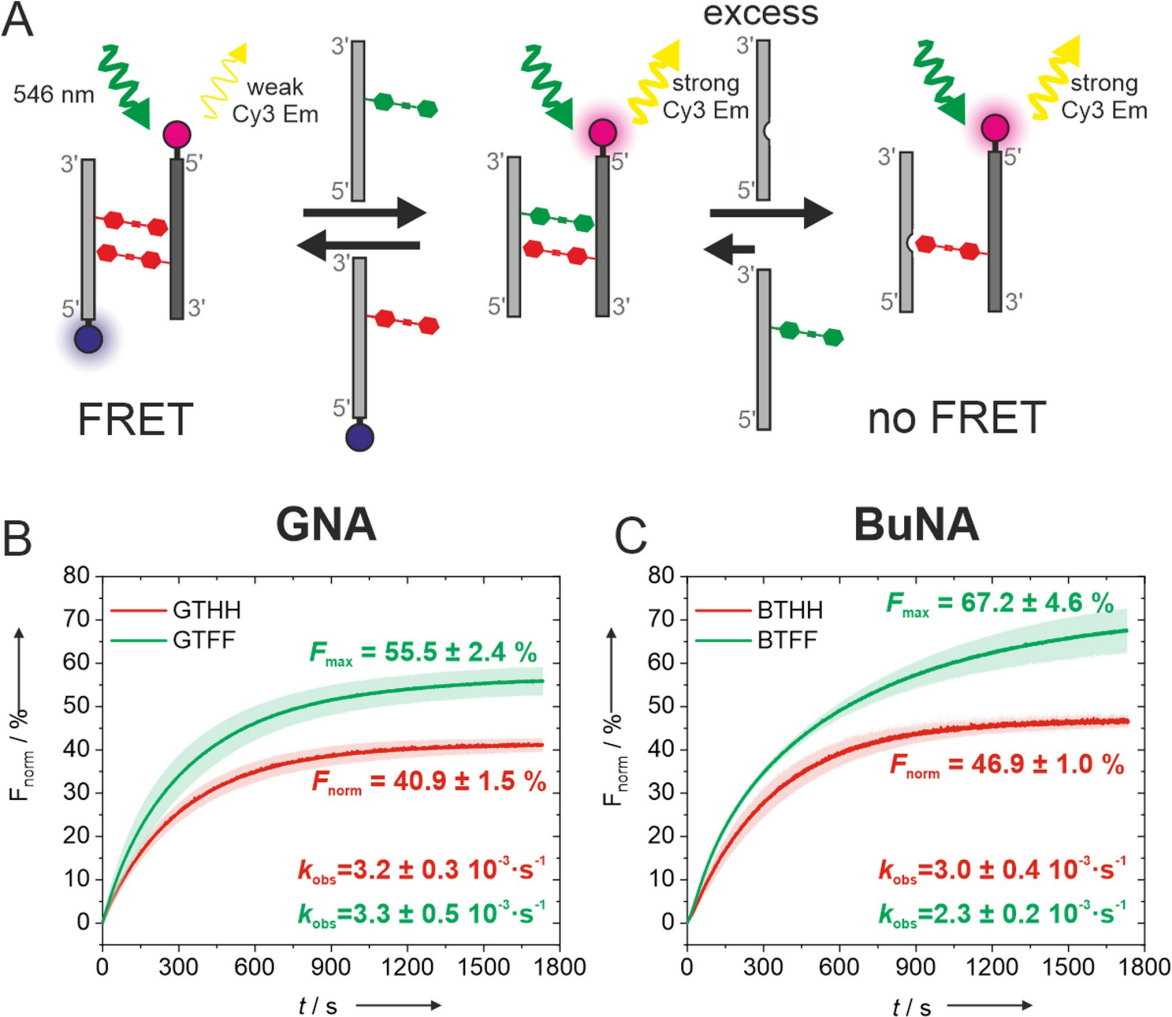
A) Schematic representation of a strand displacement experiment with a THH/THH (red) homopair and an unlabelled strand containing TFF (green). Strand 1 (light grey) is labelled with Cy5 (blue) and strand 2 (dark grey) is labelled with Cy3 (magenta). DNA strand containing C3 linker is represented with a gap. B,C) Fraction of total displacement (*F*_norm_) as a function of time, upon addition of an unlabelled THH (red curve) or TFF (green curve) containing strand to THH/THH homomopair in GNA (B) or BuNA (C).

A systematic NMR analysis of chemical shift perturbations (CSPs), imino‐water exchange rates (k_E*X*_) and NOESY cross peaks was conducted on the duplexes GTHH/GTHH, GTFF/GTHH, BTHH/BTHH and BTFF/BTHH, as well as on the reference T/A DNA duplex (Figure S6–S19). All the non‐terminal imino protons were observed in the ^1^H 1D NMR spectrum of the modified duplexes at 25 °C, and assigned as indicated in Figure 4A. For both GNA and BuNA backbones, THH/THH induced a downfield shift of G8 H1 (blue arrow, Figure 4A) and an upfield shift of G19 H1 (violet arrow, Figure 4A) compared to the reference duplex. In contrast, G8 H1 and G19 H1 were both upfield shifted for the TFF/THH heteropairs in both GNA and BuNA. Importantly, all imino signals near the modification site were detected as sharp signals even at 45 °C (Figure S7), suggesting that no major disruption of the base pairs is required to accommodate the tolane units. This is in strong contrast to the results observed by Christensen et al. for a DNA duplex containing pyrenes linked to a GNA backbone, where the modification induced an overall severe perturbation of the DNA duplex, including base pair disruption.^[26]^ Also comprehensive CSP analyses of aromatic base protons and sugar protons suggest only local perturbation of the duplex (Figure S12). Analysis of the tolane resonances revealed a single peak for pairs even at 10 °C of rotationally symmetric tolane ring protons, which showed that the tolane moieties in the modification site are flipping fast on the NMR time scale (Figure S13A–D). This fast rotation has been reported also for two biphenyl moieties embedded as C‐nucleosides in a similar DNA duplex.^[27]^ The combined ^1^H/^13^C CSPs for THH18 next to THH7 or TFF7 were analyzed (Figure S13E–F), and showed comparable trends for changing from a homo‐ to a heteropair within the GNA and BuNA backbones.

**Figure 4:**
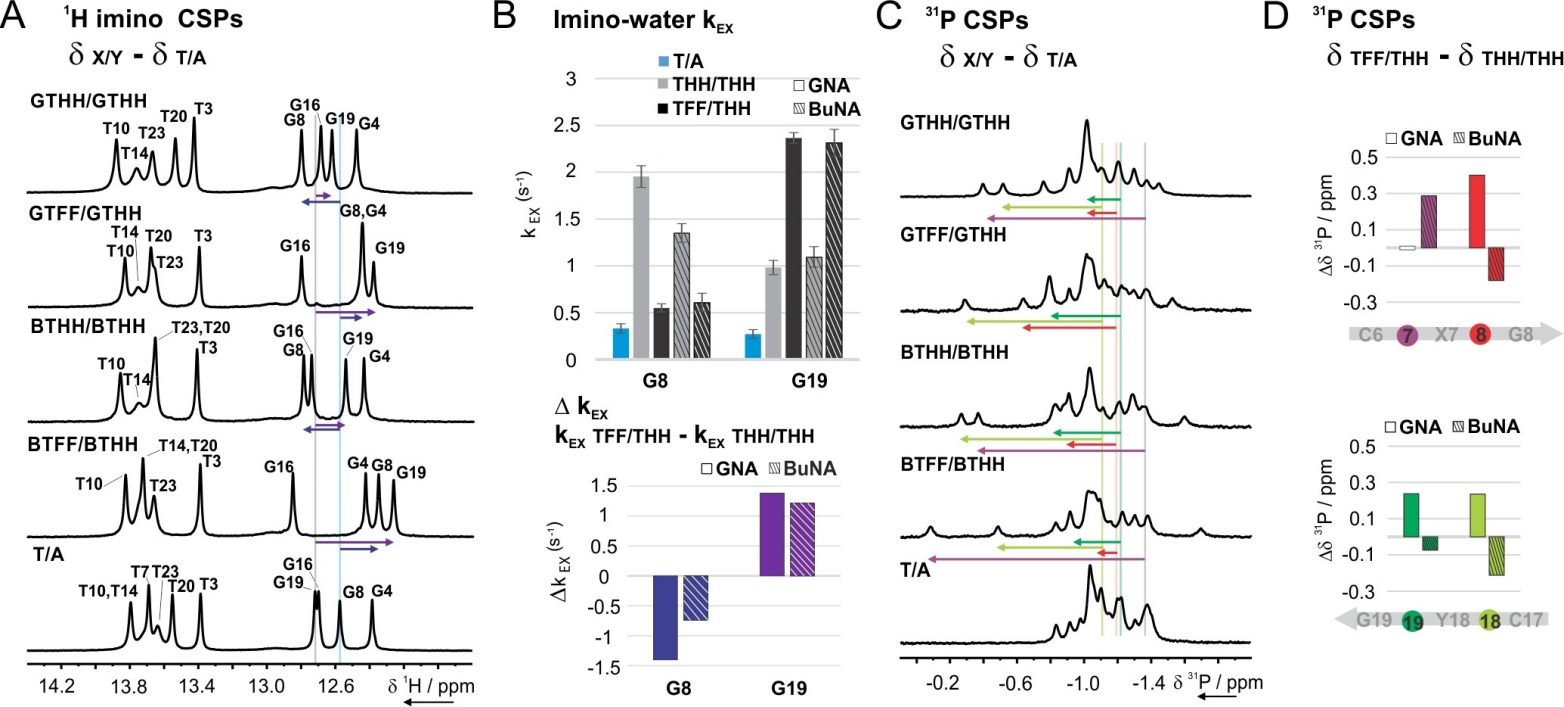
A) Imino region of the 1D ^1^H NMR spectrum at 25 °C of the duplexes indicated on the left with assignment. Chemical shift perturbation (CSP) of imino protons belonging to residues G8 and G19 is indicated with blue and violet arrows, respectively. B) Imino‐water exchange rates (k_E*X*_) of imino protons belonging to residues G8 and G19, as determined by CLEANEX‐PM experiments at 25 °C (B, top). Comparison of G8 and G19 k_E*X*_ for homo‐ and heteropairs embedded in GNA (full bars) or BuNA (dashed bars) backbone (B, bottom). C) 1D ^31^P NMR spectra of the DNA duplexes with CSP (vs reference T/A duplex) of selected phosphate units indicated with arrows (see panel D for color code and numbering). D) ^31^P CSP (TFF/THH vs THH/THH containing duplexes) of phosphate units belonging to residues adjacent to the modification site in GNA (full bars) and BuNA (dashed bars).

CLEANEX‐PM experiments were performed to obtain imino‐water exchange rates,^[28]^ in order to analyze the duplex stability at the base pair level (Figure S14). Focusing on the C6‐G19 and G8‐C17 Watson–Crick base pairs flanking the tolane pairs, comparable effects were observed for both GNA and BuNA backbones (Figure 4B). However, the relative influences of THH and TFF were quite distinct. A THH/THH homopair destabilizes both neighboring G−C base pairs to a similar extent, while the TFF/THH heteropair has an asymmetric effect. A reduced exchange rate of the G8 stacking on TFF comes along with a stronger disturbance (enhanced exchange rate) of G19 flanking the THH moiety.

Next, we evaluated the impact of the tolane modifications on the phosphate backbone by analyzing the ^31^P chemical shifts (Figure 4C–D and Figure S16). The phosphate groups connected to the Z‐end of the tolane units (P7 and P18, violet and light green, respectively, in Figure 4C–D) showed the largest ^31^P CSP compared to the reference T/A in all the duplexes (Figure 4C). Upon replacing THH7 with TFF7, the ^31^P CSPs of the phosphate groups P8, P18 and P19 showed opposite trends depending on the length of the acyclic backbone (Figure 4D): downfield shifts with GNA and upfield shifts with BuNA. Since downfield shifts can be interpreted as an increase of BII/BI population ratio,^[29]^ we can conclude that a THH/THH to TFF/THH substitution results in an increased backbone distortion in presence of GNA, but not in presence of BuNA. Thus, the ^31^P CSP data are in line with the lower global thermal stability observed for the GTFF/GTHH containing duplex, compared to the BTFF/BTHH.

Further insights into the local architecture were obtained by analysis of NOESY cross peaks for the samples BTHH/BTHH and BTFF/BTHH (Figure 5, S17–S19). The dense network of ^1^H,^1^H homonuclear and ^19^F,^1^H heteronuclear NOE interactions supports a head‐to‐tail arrangement of the tolane units in both homo‐ and heteropair combinations. Inter‐tolane NOESY cross peaks were detected between the outer ring (C/D/E) of one unit and the inner ring (A/B) of the other unit. Furthermore, the outer ring of each tolane unit showed several NOE contacts to the butyl linker of the opposite strand as well as to the sugar unit Z‐end of the opposite tolane unit, indicating that the tolane moiety spans the complete neighboring base pair. Interestingly, the NOESY spectra of the BTHH/BTHH duplex revealed that each tolane unit had several cross peaks with both GC base pairs flanking the modification site, rather than to only one expected preferred neighboring base pair (Figure S17A,18). As example, Figure 5A,C shows NOE contacts between each tolane unit and the imino protons of both G8 and G19. In contrast, such imino‐tolane inter‐strand cross peaks were not detected for the heteroduplex BTFF/BTHH (Figure 5B,D). Consistent with a polar‐π‐stacked orientation of the arene‐fluoroarene heteropair, medium to strong NOE contacts with only one base pair were observed (Figure S17B,19). Taken together, the NOE contact map as well as the intensities of the NOESY cross peaks (Figure S17–19) are consistent with the interpretation that a TFF/THH duplex forms a stacked heteropair, while the THH/THH homopair is not arranged in a preferred π‐stacked orientation but may experience a more flexible environment (sketch in Figure 5E). The combined data for the BTFF/BTHH pair suggest that the inner ring of each tolane unit is stacked to the neighboring guanine base and the outer ring is stacked mostly to the corresponding base paired cytosine (sketch in Figure 5F).

**Figure 5:**
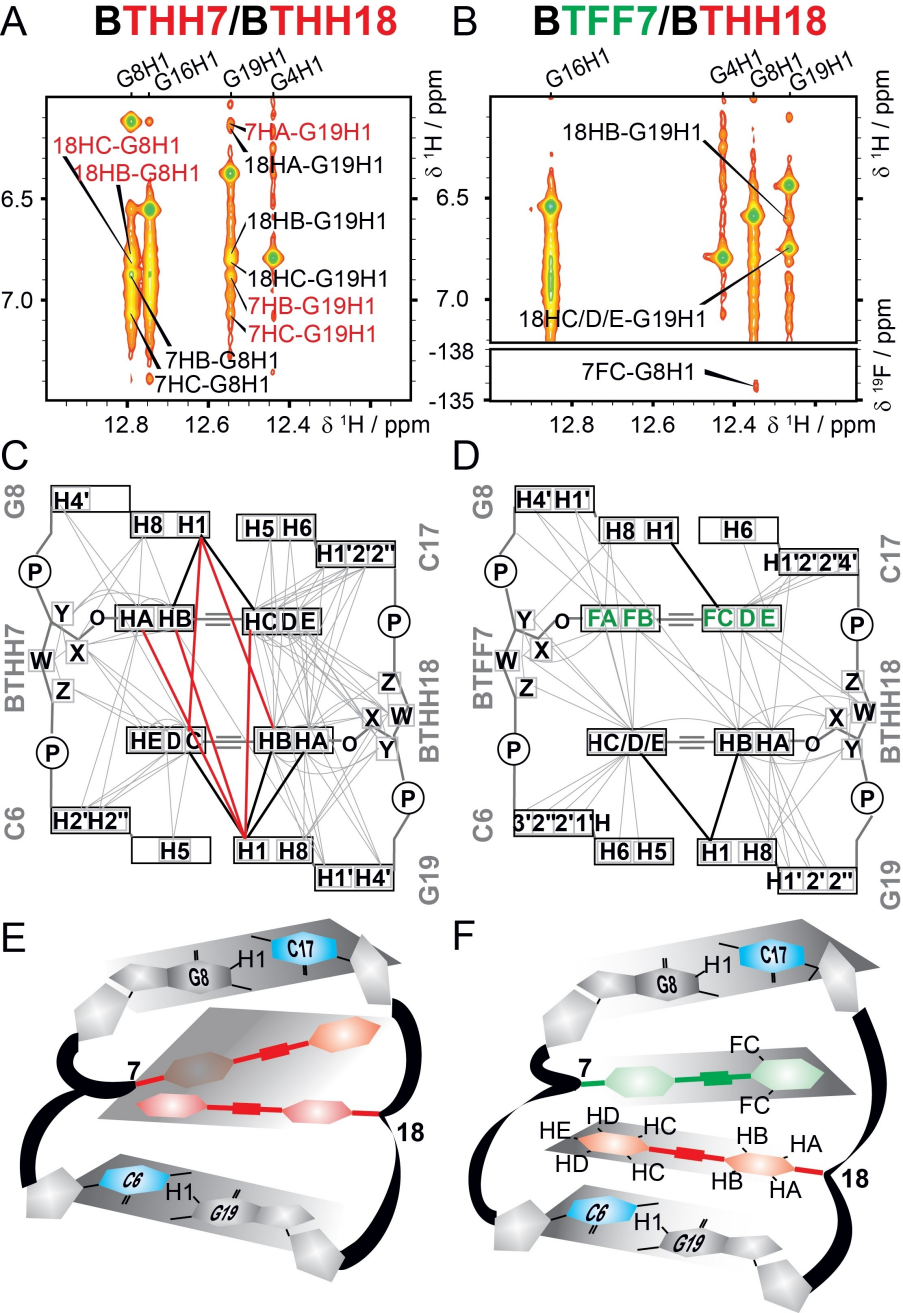
A,B) Imino/aromatic region of ^1^H,^1^H NOESY spectrum of BTHH/BTHH (A) and BTFF/BTHH (B, top) and ^1^H,^19^F HOESY (B, bottom), with cross peaks between the tolane units and the imino protons of G8 and G19. C,D) Schematic representation of all the homo‐ and heteronuclear NOE correlations with the tolane groups observed in ^1^H,^19^F HOESY and ^1^H,^1^H NOESY spectra. Imino‐tolane NOE contacts assigned in panels A–B are shown as bold lines, inter‐strand contacts are bold red. Further details are given in Figure S17. E,F) Sketches of the corresponding homopair (E) and heteropair (F) regions.

In summary, we introduced a bioorthogonal supramolecular recognition motif based on the tolane moiety, which is driven by polar π‐stacking and serves as a base pair replacement in DNA. The interaction energy of a TFF/THH heteropair contributes 1.4 kcal mol^−1^ to DNA duplex stabilization when it is incorporated via a BuNA backbone, but not when it is attached to a GNA backbone. Analysis of a double mutant cycle and imino exchange rate analyses allowed us to disentangle the directional stacking contributions of the fluorotolanes to the neighboring Watson–Crick base pairs. Comprehensive NMR CSP analyses together with NOESY data support the model, in which the tolane moieties of a heteropair are engaged in π‐stacking onto each other in a head to tail fashion. Consistent with the thermodynamic data, the NMR ^31^P CSP data revealed that a stronger perturbation of the DNA backbone is required to accommodate a GNA heteropair than for the BuNA heteropair. Thus, even an apparently small modification of the backbone constitution has a large impact on the stacking geometry and the overall duplex stability. While a continuous BuNA backbone is too flexible for stable Watson–Crick base pairing,^[23]^ it is clearly a privileged scaffold for exploiting the supramolecular tolane‐fluorotolane interaction in a DNA duplex. Further modifications of the tolane moiety may be used to refine the modes of aromatic interactions, including complementary partially fluorinated tolanes, that could lead to arene‐fluoroarene programmed assembly of DNA structures in the future.

## Conflict of interest

The authors declare no conflict of interest.

## Supporting information

As a service to our authors and readers, this journal provides supporting information supplied by the authors. Such materials are peer reviewed and may be re‐organized for online delivery, but are not copy‐edited or typeset. Technical support issues arising from supporting information (other than missing files) should be addressed to the authors.

Supporting Information

## Data Availability

The data that support the findings of this study are available in the supplementary material of this article.
