# Supplementary material for: Tailored Tolane‐Perfluorotolane Assembly as Supramolecular Base Pair Replacement in DNA
Source: Angew Chem Int Ed Engl. 2022 Dec 1;62(1):e202214456. doi: 10.1002/anie.202214456 (PMC10107946; doi:10.1002/anie.202214456)
Supplement: Supplementary file 1 — Supporting Information [file ANIE-62-0-s001.pdf]

Angewandte Chemie
Eine Zeitschrift der Gesellschaft Deutscher Chemiker

Supporting Information

Tailored Tolane-Perfluorotolane Assembly as Supramolecular Base Pair Replacement in DNA

*H. Neitz, I. Bessi, V. Kachler, M. Michel, C. Höbartner**

Supporting Information for

Tailored Tolane-Perfluorotolane Assembly as Supramolecular Base Pair Replacement in DNA

Hermann Neitz, Irene Bessi, Valentin Kachler, Manuela Michel and Claudia Höbartner*

Institute of Organic Chemistry, University of Würzburg, Am Hubland, 97074 Würzburg, Germany

Table of Contents

Supporting Schemes, Tables and Figures

		page
Scheme S1	Synthesis of GTHH and BTHH phosphoramidites	2
Scheme S2	Synthesis of GTFF and BTFF phosphoramidites	2
Table S1	Sequences and ESI-MS data of DNA oligonucleotides	3
Table S2	Thermodynamic data from melting curves	3
Table S3	Kinetic data from strand displacement FRET assay	4
Figure S1	Thermal stability of the tolane moiety and a natural nucleobase	5
Figure S2	Double mutant cycle for GNA series	5
Figure S3	Replacement of neighboring base-pair	6
Figure S4	Schematic representation and explanation of FRET strand displacement experiment	7
Figure S5	Strand displacement experiment with a TFF/TFF homopair	7
Figure S6	Assignment of the imino regions of the 1D ^{1}H NMR spectra and of the 1D ^{19}F NMR spectra of the duplexes at 25 °C	8
Figure S7	Imino regions of the 1D ^{1}H NMR spectra at various temperatures	9
Figure S8	Assignment of the imino regions of the 2D ^{1}H,^{1}H NOESY NMR spectra	10
Figure S9	Assignment of the aromatic-anomeric region of the 2D ^{1}H,^{1}H NOESY NMR spectrum for the modified duplexes	11
Figure S10	Assignment of the aromatic-anomeric region of the 2D ^{1}H,^{1}H NOESY NMR spectrum for the reference duplex	12
Figure S11	Assignment of the 2D ^{1}H,^{19}F HOESY spectrum of BTFF/BTHH duplex	13
Figure S12	Chemical shift perturbation analysis of the DNA base and sugar protons	14
Figure S13	Tolane region of the ^{1}H,^{13}C HSQC spectra of the modified duplexes at 10 °C and CSP analysis	15
Figure S14	Imino-water exchange rates derived by CLEANEX-PM experiments	16
Figure S15	Acyclic backbone region of ^{1}H,^{1}H DQF-COSY and ^{1}H,^{1}H NOESY spectra for the modified duplexes	17
Figure S16	Assignment of the ^{31}P NMR spectra	18
Figure S17	Detailed schematics of the tolane NOE contacts	19
Figure S18	Assignment of the aromatic region of the NOESY spectrum of BTHH/BTHH	20
Figure S19	Assignment of the aromatic region of the NOESY spectrum of BTFF/BTHH	21

Experimental Procedures

General Materials and Methods	22
Synthetic procedures and NMR spectra for compounds S1-S20	22-63
DNA oligonucleotide synthesis	64
5'-Labeling with click chemistry	64
UV/VIS spectroscopy and thermal melting	64
Calculation of double mutant cycle	65
Fluorescence spectroscopy and FRET displacement assay	65
NMR spectroscopy of DNA duplexes	66
Computational methods	67

Supporting / raw data

HPLC chromatograms	68-70
Melting curves and van't Hoff plots	71-75
FRET displacement curves	76-83

Cartesian coordinates 84

References 85

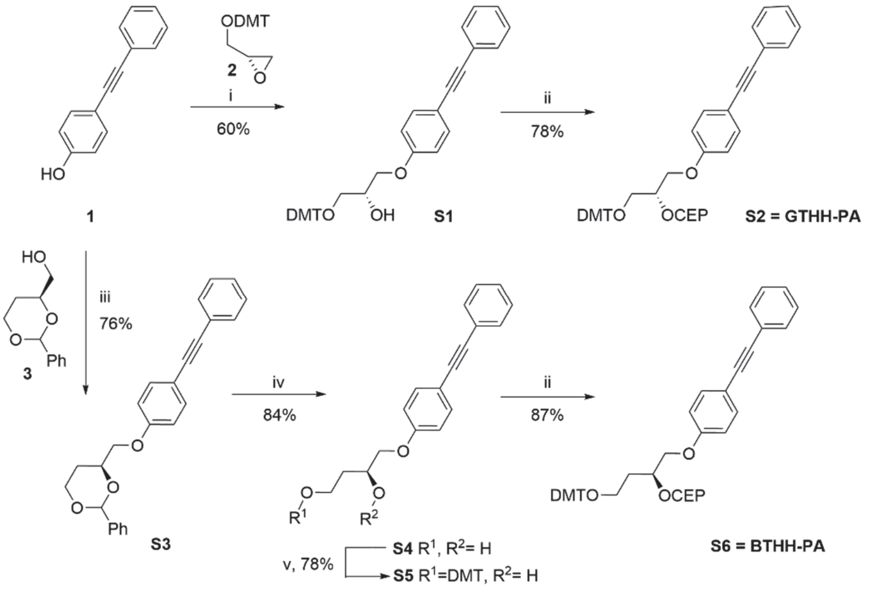

Scheme S1. Synthesis of the GTHH and BTHH phosphoramidite. i) NaH, DMF, 110°C, 1 d; ii) CEP-Cl, DIPEA, DCM, r.t., 6 h; iii) PPh_3, DIAD, THF, r.t., 30 min; iv) *p*-toluenesulfonic acid, MeOH, r.t., 40 h; v.) DMT-Cl, pyridine, r.t.; 3 h.

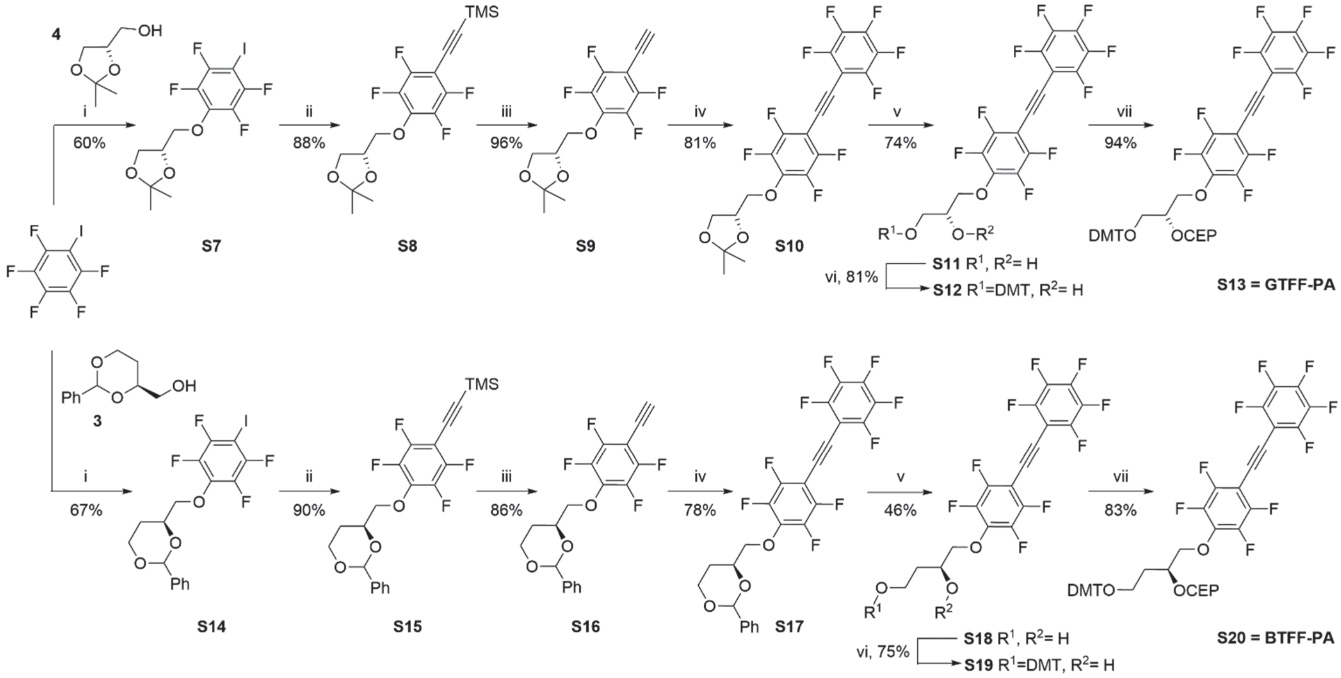

Scheme S2. Synthesis of the GTFF and BTFF phosphoramidites. i) NaH, THF, r.t., 3 h; ii) trimethylsilylacetylene, CuI, $Pd(PPh_3)_4$, 60°C, 3 h for S8; 80°C, 20 h for S15; iii) K_2CO_3, THF/MeOH, r.t., 2 h; iv) C_6F_5I, CuI, $Pd(PPh_3)_4$, 80°C, 3 h for S10; 80°C, 20 h for S17; v) 2 M HCl, THF, r.t., 4-5 d; vi) DMT-Cl, pyridine, r.t.; 23 h; vii) CEP-Cl, DIPEA, DCM, r.t., 2 h.

Table S1. Sequences and ESI-MS results of the used DNA oligonucleotides.

Oligo	5'-Sequence-3'	Chemical formula	Calc. Mass	Measured Mass
Reference1	GATGACTGCTAG	$C_{118}H_{148}N_{47}O_{70}P_{11}$	3683.65798	3683.63696
Reference2	CTAGCAGTCATC	$C_{116}H_{148}N_{43}O_{70}P_{11}$	3603.64569	3603.63199
AbasicLinker1	GATGAC(C3)GCTAG	$C_{111}H_{142}N_{45}O_{67}P_{11}$	3517.62069	3517.57859
AbasicLinker2	CTAGC(C3)GTCATC	$C_{111}H_{142}N_{45}O_{67}P_{11}$	3428.59683	3428.57390
GTHH1	GATGAC(GTHH)GCTAG	$C_{125}H_{150}N_{45}O_{68}P_{11}$	3709.67766	3709.68018
Alk-GTHH1	Alk-GATGAC(GTHH)GCTAG	$C_{131}H_{159}N_{45}O_{71}P_{12}$	3869.70659	3869.72739
GTHH2	CTAGC(GTHH)GTCATC	$C_{123}H_{151}N_{38}O_{70}P_{11}$	3620.65434	3620.67986
Alk-GTHH2	Alk-CTAGC(GTHH)GTCATC	$C_{129}H_{160}N_{38}O_{73}P_{12}$	3780.68272	3780.69911
GTFF1	GATGAC(GTFF)GCTAG	$C_{125}H_{141}F_9N_{45}O_{68}P_{11}$	3871.59286	3871.59038
Alk-GTFF1	Alk-GATGAC(GTFF)GCTAG	$C_{131}H_{150}F_9N_{45}O_{71}P_{12}$	4031.62234	4031.62269
GTFF2	CTAGC(GTFF)GTCATC	$C_{123}H_{142}F_9N_{38}O_{70}P_{11}$	3782.56900	3782.58448
Alk-GTFF2	Alk-CTAGC(GTFF)GTCATC	$C_{129}H_{151}F_9N_{38}O_{73}P_{12}$	3942.59848	3942.61320
BTHH1	GATGAC(BTHH)GCTAG	$C_{126}H_{152}N_{45}O_{68}P_{11}$	3723.69331	3723.70657
Alk-BTHH1	Alk-GATGAC(BTHH)GCTAG	$C_{132}H_{161}N_{45}O_{71}P_{12}$	3883.72224	3883.71342
BTHH2	CTAGC(BTHH)GTCATC	$C_{124}H_{153}N_{38}O_{70}P_{11}$	3634.66944	3634.66984
Alk-BTHH2	Alk-CTAGC(BTHH)GTCATC	$C_{130}H_{162}N_{38}O_{73}P_{12}$	3794.69837	3794.68923
BTFF1	GATGAC(BTFF)GCTAG	$C_{126}H_{143}F_9N_{45}O_{68}P_{11}$	3885.60851	3885.61827
Alk-BTFF1	Alk-GATGAC(BTFF)GCTAG	$C_{132}H_{152}F_9N_{45}O_{71}P_{12}$	4045.63799	4045.63546
BTFF2	CTAGC(BTFF)GTCATC	$C_{124}H_{144}F_9N_{38}O_{70}P_{11}$	3796.58520	3796.62324
Alk-BTFF2	Alk-CTAGC(BTFF)GTCATC	$C_{130}H_{153}F_9N_{38}O_{73}P_{12}$	3956.61413	3956.62462
T_BTHH	GATGAT(BTHH)GCTAG	$C_{127}H_{153}N_{44}O_{69}P_{11}$	3738.69297	3738.71257
BTHH_A	CTAGC(BTHH)ATCATC	$C_{124}H_{153}N_{38}O_{69}P_{11}$	3618.67453	3618.67141
T_BTFF	GATGAT(BTFF)GCTAG	$C_{127}H_{144}N_{44}O_{69}P_{11}F_9$	3900.60873	3900.62418
BTFF_A	CTAGC(BTFF)ATCATC	$C_{124}H_{144}N_{38}O_{69}P_{11}F_9$	3780.59028	3780.60927

Table S2. Thermodynamic data for DNA duplexes in phosphate buffer (100 mM NaCl, 10 mM sodium phosphate, pH 7.0).

Duplex	Oligo	Sequence	c_{total}[a] [µM]	T_m [°C]	ΔH^0 [kcal mol^{-1}]	ΔS^0 [cal mol^{-1}]	ΔG^{298}[b] [kcal mol^{-1}]
T/A	Reference1<br>Reference2	5'…CTG…3'<br>3'…GAC…5'	2.0<br>4.0<br>10.0<br>19.7<br>40.2	44.8<br>46.3<br>48.5<br>49.6<br>51.5	-92.9±1.5	-263±4.3	-14.5±2.0
GTHH/GTHH	GTHH1<br>GTHH2	5'…C(GTHH)G…3'<br>3'…G(GTHH)C…5'	2.2<br>4.4<br>10.8<br>21.2<br>43.2	40.7<br>42.6<br>45.2<br>46.7<br>48.8	-74.5±0.6	-209±1.6	-12.2±0.7
GTHH/GTFF	GTHH1<br>GTFF2	5'…C(GTHH)G…3'<br>3'…G(GTFF)C…5'	2.2<br>4.5<br>11.0<br>21.4	42.4<br>44.3<br>47.1<br>48.9	-69.6±0.4	-192±1.2	-12.3±0.6

			44.2	51.2			
GTFF/GTHH	GTFF1<br>GTHH2	5'…C(GTFF)G…3'<br>3'…G(GTHH)C…5'	2.1<br>4.3<br>11.0<br>21.4<br>44.3	43.5<br>45.4<br>48.1<br>49.8<br>52.0	-73.3±0.5	-203±1.3	-12.9±0.6
GTFF/GTFF	GTFF1<br>GTFF2	5'…C(GTFF)G…3'<br>3'…G(GTFF)C…5'	2.1<br>4.4<br>11.0<br>21.7<br>44.4	44.8<br>46.9<br>49.8<br>51.6<br>54.1	-68.5±0.6	-187±1.6	-12.8±0.8
BTHH/BTHH	BTHH1<br>BTHH2	5'…C(BTHH)G…3'<br>3'…G(BTHH)C…5'	2.0<br>4.3<br>10.9<br>21.4<br>44.3	42.0<br>43.9<br>46.5<br>48.1<br>50.3	-76.7±0.7	-214±2.1	-12.8±1.0
BTHH/BTFF	BTHH1<br>BTFF2	5'…C(BTHH)G…3'<br>3'…G(BTFF)C…5'	2.1<br>4.2<br>11.3<br>21.5<br>44.4	46.9<br>48.4<br>51.1<br>52.4<br>54.7	-81.8±1.1	-227±3.2	-14.1±1.5
BTFF/BTHH	BTFF1<br>BTHH2	5'…C(BTFF)G…3'<br>3'…G(BTHH)C…5'	2.2<br>4.5<br>11.4<br>22.2<br>45.6	48.1<br>49.6<br>52.1<br>53.6<br>55.8	-83.3±1.2	-231±3.3	-14.6±1.5
BTFF/BTFF	BTFF1<br>BTFF2	5'…C(BTFF)G…3'<br>3'…G(BTFF)C…5'	2.3<br>4.7<br>11.8<br>23.1<br>48.0	45.2<br>47.1<br>49.9<br>51.6<br>54.1	-71.7±0.8	-197±2.3	-13.1±1.1
T_BTHH/<br>BTHH_A	T_BTHH<br>BTHH_A	5'…T(BTHH)G…3'<br>3'…A(BTHH)C…5'	2.0<br>4.1<br>10.5<br>20.1<br>41.7	40.2<br>42.0<br>44.5<br>45.9<br>48.0	-78.5±0.5	-222±1.5	-12.4±0.7
T_BTHH/<br>BTFF_A	T_BTHH<br>BTFF_A	5'…T(BTHH)G…3'<br>3'…A(BTFF)C…5'	2.1<br>4.4<br>11.3<br>21.4<br>44.2	41.2<br>43.2<br>45.6<br>47.7<br>49.6	-72.6±0.6	-202±1.8	-12.4±0.8
T_BTFF/<br>BTHH_A	T_BTFF<br>BTHH_A	5'…T(BTFF)G…3'<br>3'…A(BTHH)C…5'	2.2<br>4.4<br>11.2<br>21.7<br>44.9	44.1<br>45.8<br>48.3<br>50.1<br>52.1	-77.1±0.3	-215±0.8	-13.1±0.4
T_BTFF/<br>BTFF_A	T_BTFF<br>BTFF_A	5'…T(BTFF)G…3'<br>3'…A(BTFF)C…5'	2.0<br>4.4<br>11.4<br>22.3<br>45.8	39.3<br>41.5<br>44.3<br>46.3<br>48.5	-67.3±0.1	-186±0.2	-11.7±0.1

[a] Total concentration of DNA strands. Values were calculated with the absorption at 260 nm of the melted duplex [b] Calculated for T = 25 °C.

Table S3. Total strand displacement and displacement rate of the FRET exchange experiments. Values were obtained from a fit to first-order reaction kinetics.

Labeled duplex	Unlabeled strand	F_{max} [%]	k_{obs} [10^{-3} s^{-1}]
GTHH/GTHH	GTHH	40.9 ± 1.5	3.2 ± 0.3
GTHH/GTHH	GTFF	55.5 ± 2.4	3.3 ± 0.5
GTFF/GTFF	GTHH	49.5 ± 4.5	1.2 ± 0.1
GTFF/GTFF	GTFF	60.1 ± 4.1	0.8 ± 0.1
BTHH/BTHH	BTHH	46.9 ± 1.0	3.0 ± 0.4
BTHH/BTHH	BTFF	67.2 ± 4.6	2.3 ± 0.2
BTFF/BTFF	BTHH	70.5 ± 2.4	0.7 ± 0.1
BTFF/BTFF	BTFF	55.1 ± 2.2	1.2 ± 0.1

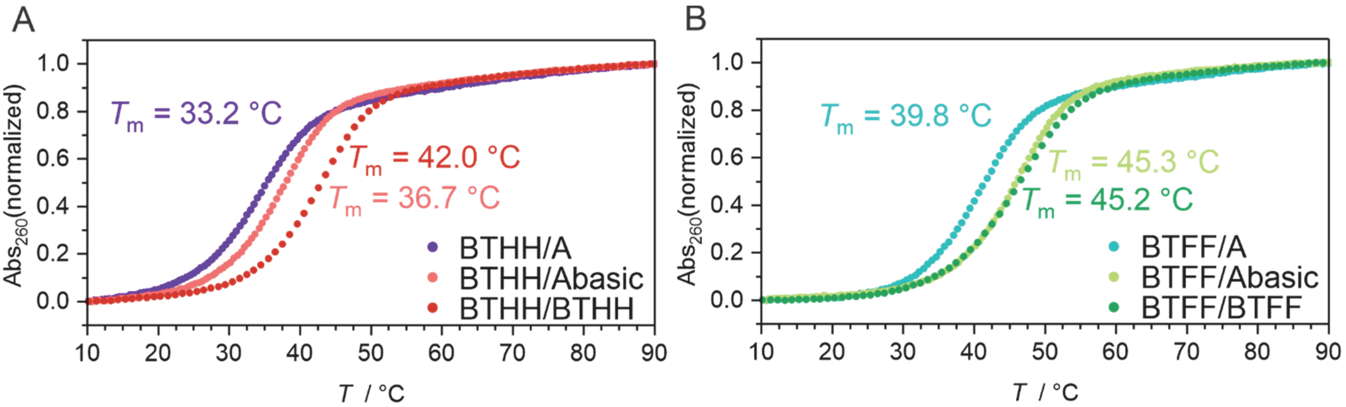

Figure S1 UV-melting curves of a DNA duplex with a A) BTHH or B) BTFF unit opposite to the nucleobase A, an abasic linker and another tolane unit.

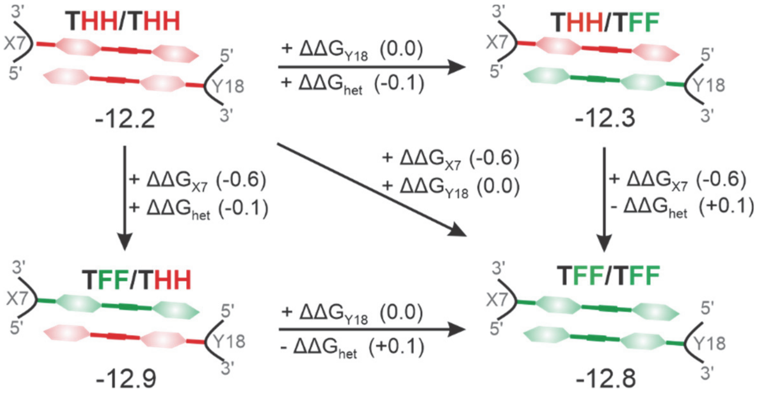

Figure S2 Double mutant cycle for GNA series. See Fig 2C in the manuscript for BuNA data.

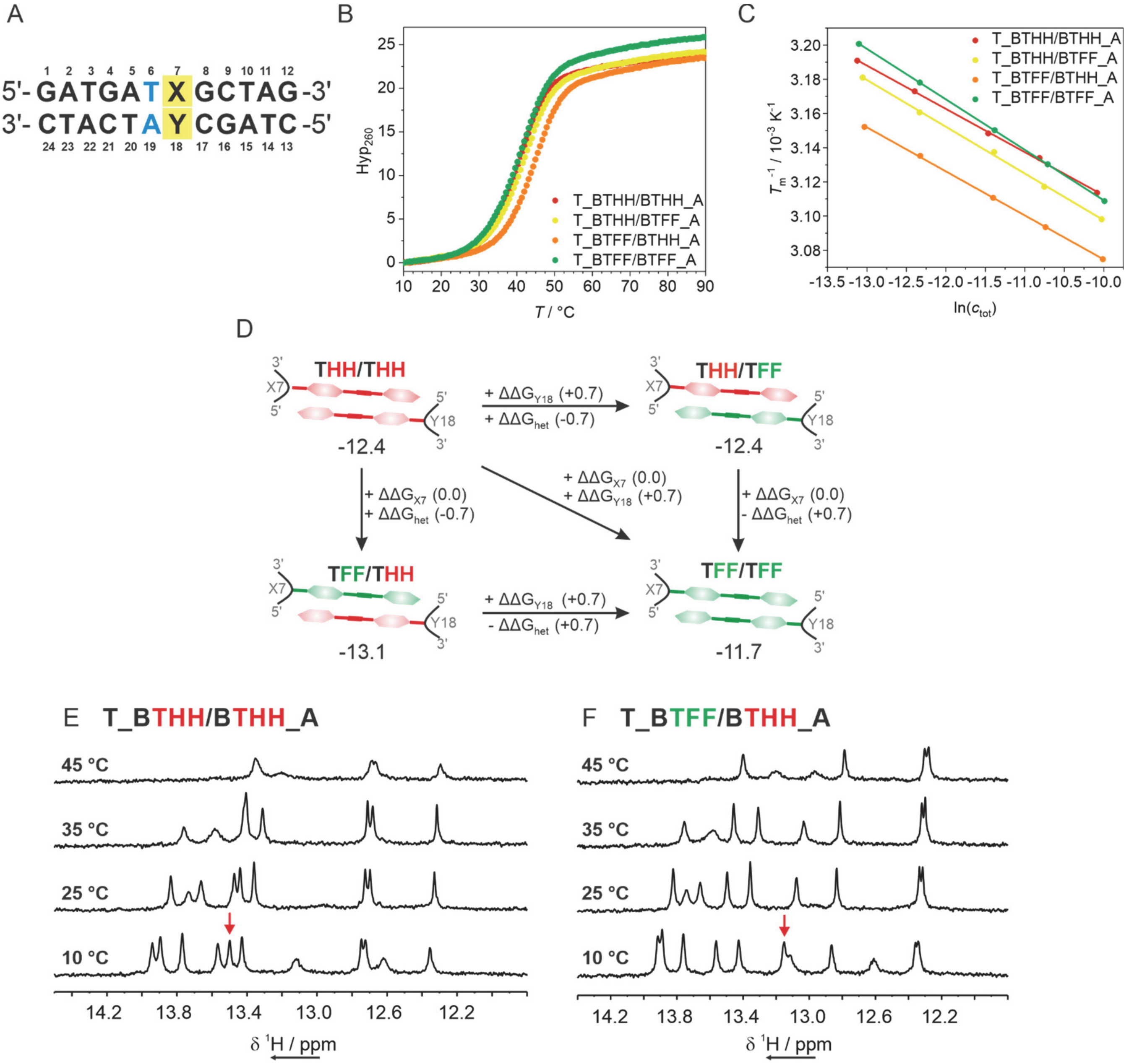

Figure S3 A) Sequence of the DNA duplex with a A/T-base pair next to the modification site (blue letters), modified position X7 and Y18 highlighted in yellow. UV-melting curves at 1 µM duplex concentration (B) and van't Hoff analysis (C) for DNA duplexes containing BuNA tolane homopairs and heteropairs. D) Double mutant cycle for the series with an A/T-base pair replacement. Imino region of the ^{1}H NMR spectrum of T_BTHH/BTHH_A (E) and T_BTFF/BTHH_A (F) at various temperatures. Spectra recorded on 50 µM DNA duplex sample in NMR buffer, 90% H_2O, 10% D_2O, 600 MHz. Red arrow indicates tentative assignment of T6H1 on the spectra recorded at 10 °C.

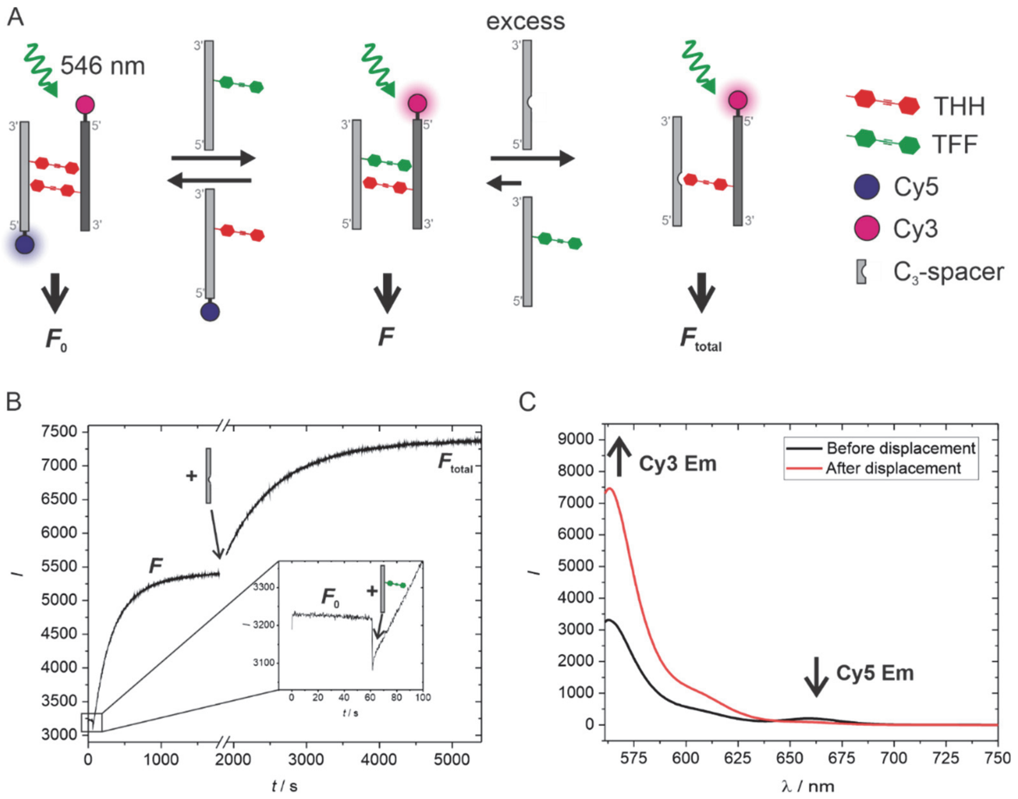

Figure S4 A) Schematic representation of a FRET strand displacement experiment starting with a THH homopair and adding an unlabeled TFF containing single strand. All variations of starting homopairs (THH/THH with TFF/TFF) with different unlabeled single strands (TFF/THH) were tested for both backbones. B) Change in Cy3 fluorescence during the FRET exchange experiment. Injection points of the single strands with TFF or THH modification and C_3-spacer and regions for the estimation of F_0, F and F_{total} are highlighted in the graph. F was normalized according to equation (10) and fitted with equation (11) to obtain the kinetic parameters F_{max} and k_{obs}. C) Emission spectra before and after complete strand displacement. Change of Cy3 and Cy5 Emission is highlighted.

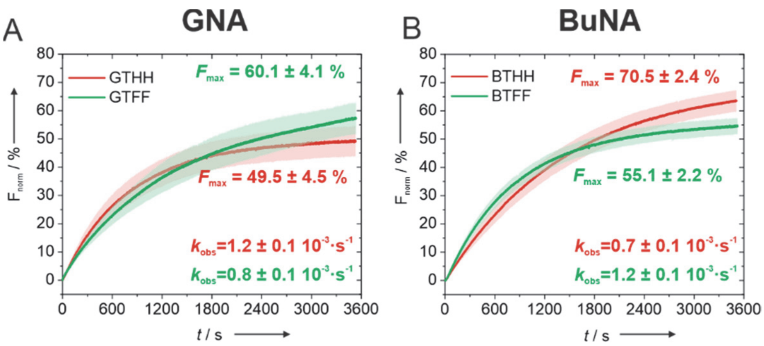

Figure S5 Fraction of total displacement (F_{norm}) as a function of time, upon addition of an unlabelled THH (red curve) or TFF (green curve) containing strand to TFF/TFF homomopair in GNA (A) or BuNA (B). See Fig 3B,C in the manuscript for TFF/TFF homomopair data.

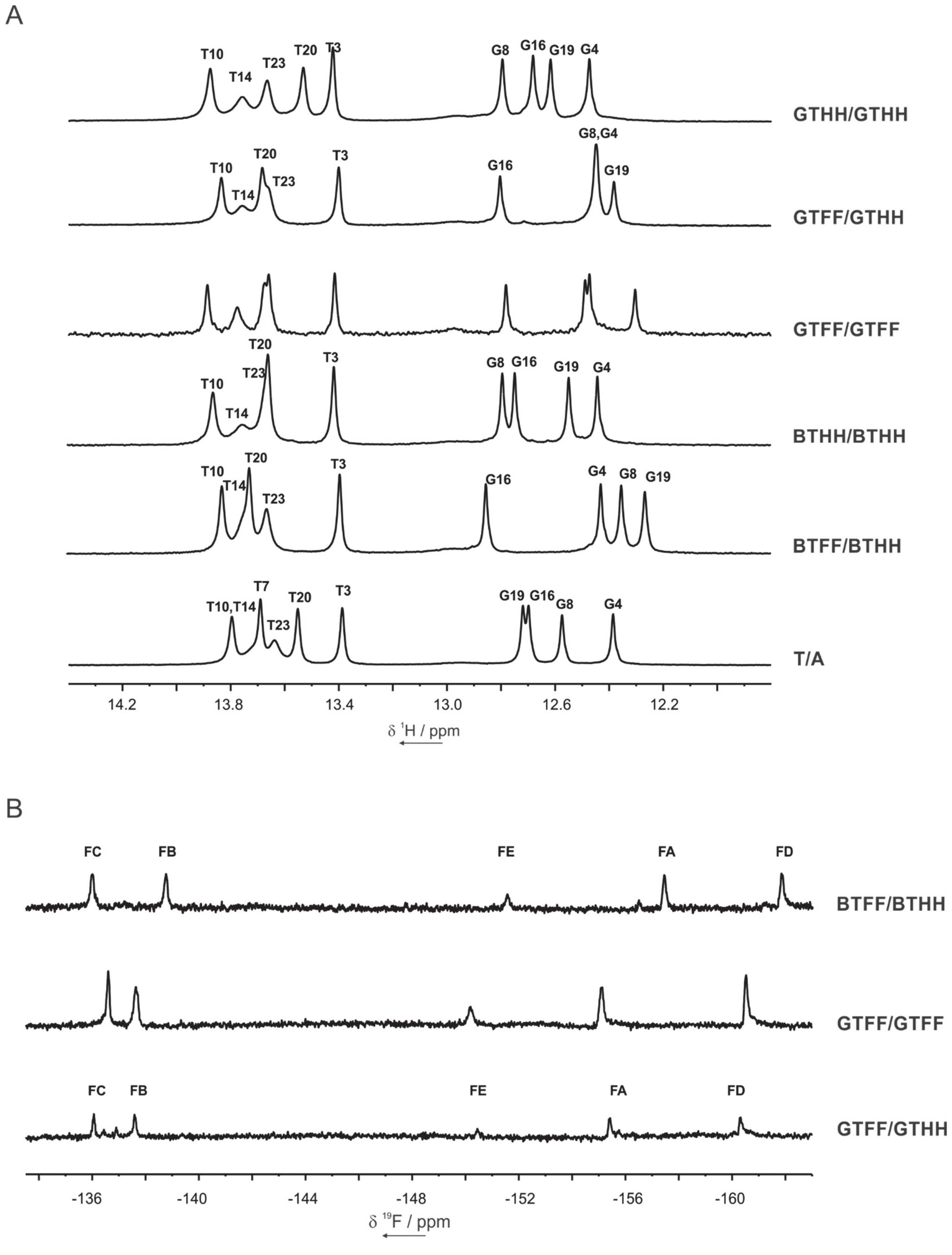

Figure S6 A) Imino region of the 1D ^{1}H NMR spectrum and B) 1D ^{19}F NMR spectrum of the duplexes indicated on the right. Assignment confirmed by 2D NMR experiment is shown (not available for GTFF/GTFF). Spectra recorded on 0.2-1.0 mM DNA duplex in NMR buffer, 90%H_2O/10%D_2O, 600 MHz, 25 °C.

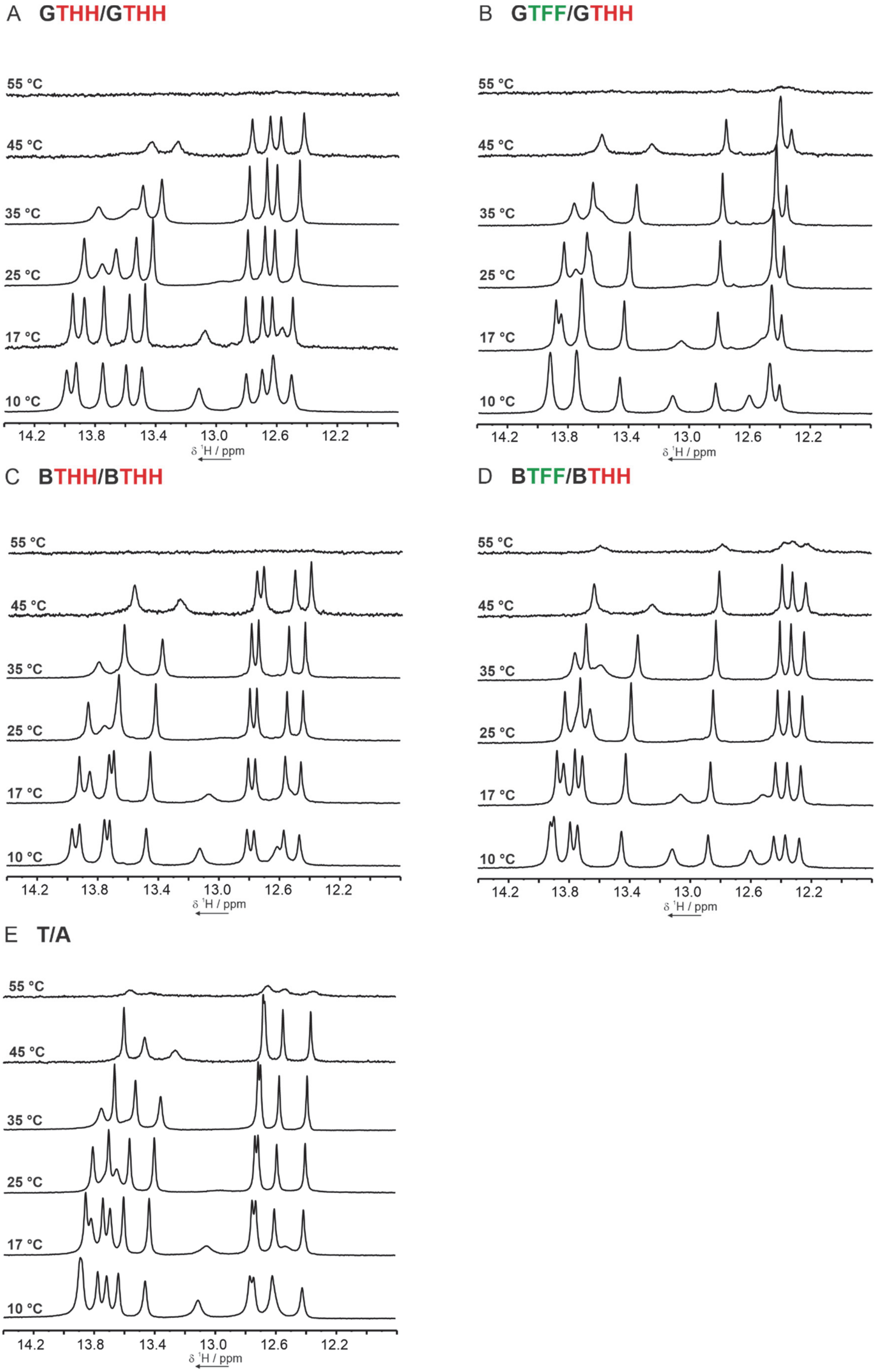

Figure S7 Imino region of the 1D ^{1}H NMR spectrum of the duplexes A) GTHH/GTHH, B) GTFF/GTHH, C) BTHH/BTHH, D) BTFF/BTHH and E) T/A at various temperatures. Spectra recorded on 0.2-1.0 mM DNA duplex in NMR buffer, 90%H_2O/10%D_2O, 600 MHz.

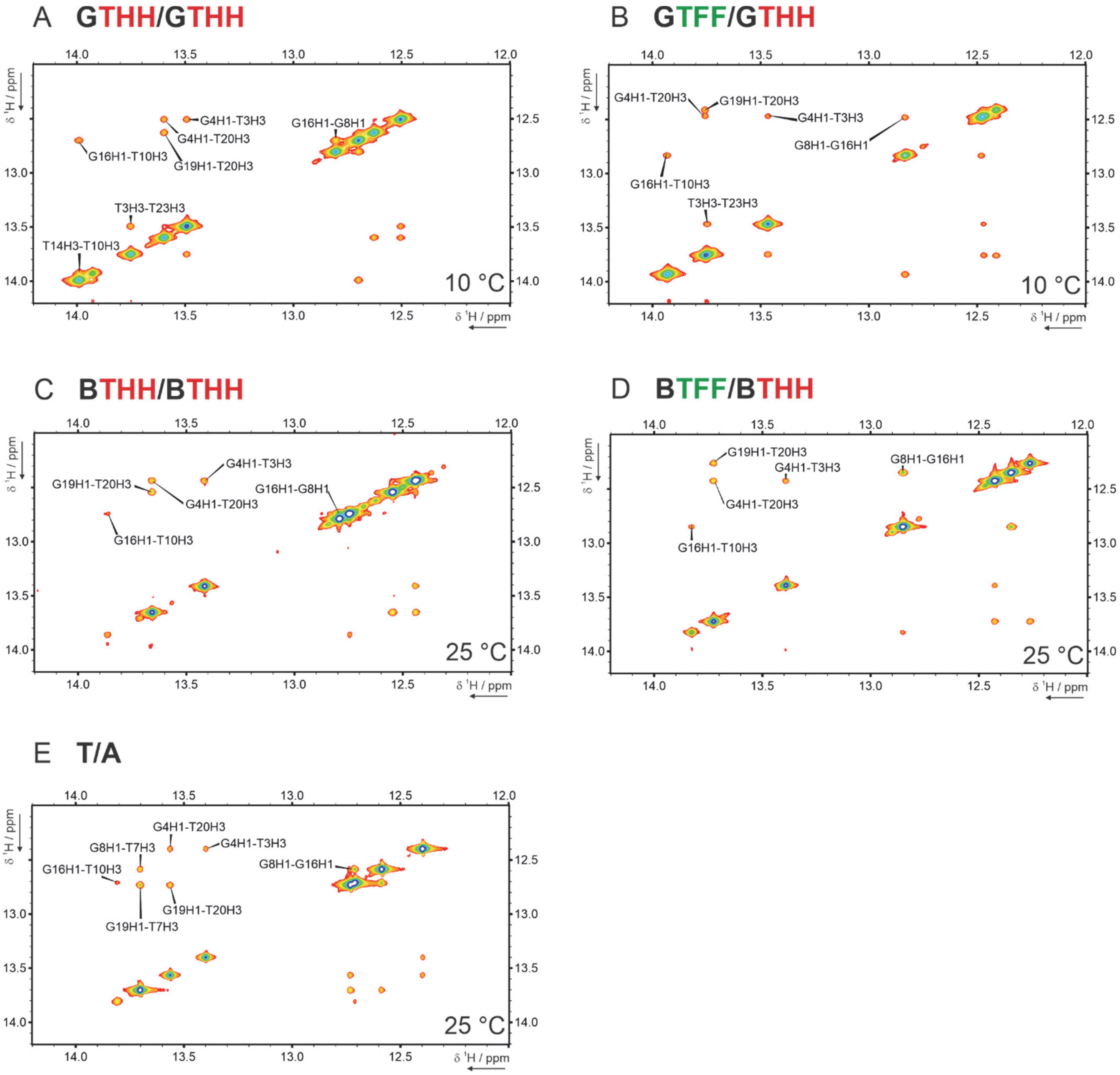

Figure S8 Imino region of the 2D $^1H,^1H$ NOESY NMR spectrum of the duplexes A) GTHH/GTHH, B) GTFF/GTHH, C) BTHH/BTHH, D) BTFF/BTHH and E) T/A at the indicated temperature. Assignment of the imino-imino connectivities is shown. Spectra recorded on 1.0 mM DNA duplex in NMR buffer, 90%H_2O/10%D_2O, 600 MHz.

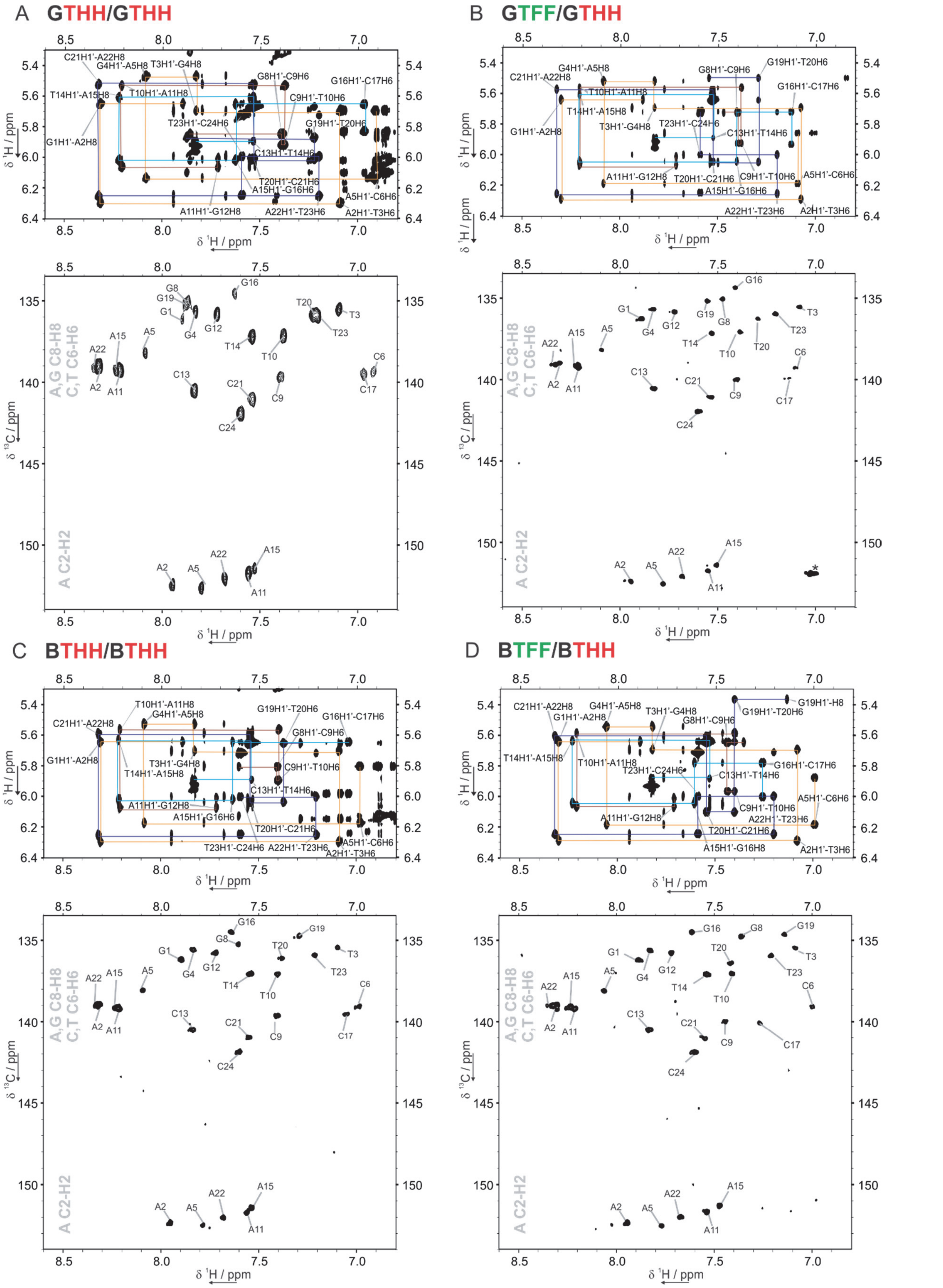

Figure S9 Aromatic-anomeric region of the 2D $^{1}H,^{1}H$ NOESY (top) spectrum and aromatic region of the $^{1}H,^{13}C$ HSQC (bottom) spectrum of the duplexes A) GTHH/GTHH, B) GTFF/GTHH, C) BTHH/BTHH, D) BTFF/BTHH. Assignment on the NOESY spectra is reported for clarity only for the sequential step 5'-H1'(n)-H6/H8(n+1)-3'. Sequential walk is indicated and color coded as follows: G1-C6 orange, G8-G12 brown, C13-C17 cyan, G19-C24 blue. Spectra recorded on 1.0 mM DNA duplex in NMR buffer, 100%D_2O, 600 MHz, 25 °C.

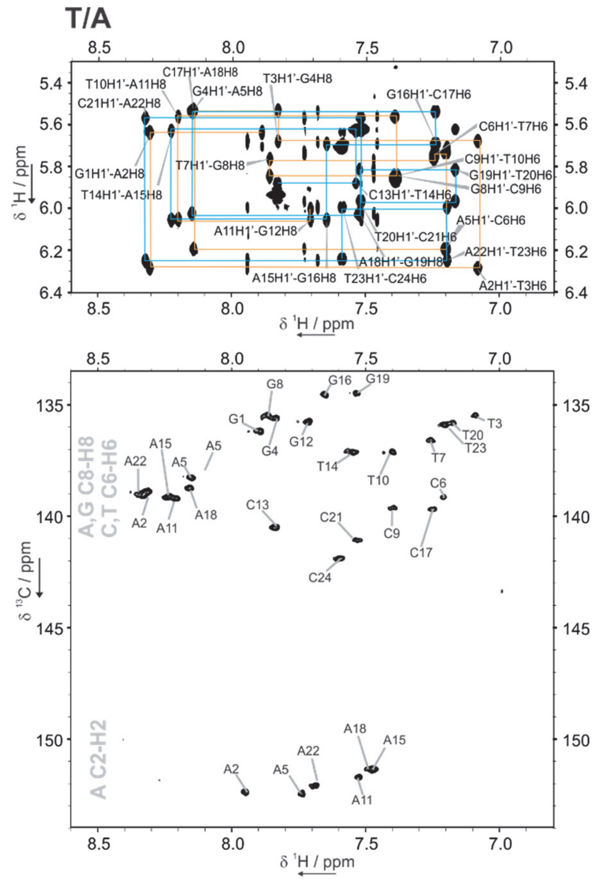

Figure S10 Aromatic-anomeric region of the 2D $^1H,^1H$ NOESY (top) spectrum and aromatic region of the $^1H,^{13}C$ HSQC (bottom) spectrum of the T/A duplex. Assignment on the NOESY spectra is reported for clarity only for the sequential step 5'-H1'(n)-H6/H8(n+1)-3'. Sequential walk is indicated and color coded as follows: G1-G12 orange, C13-C24 cyan. Spectra recorded on 1.0 mM DNA duplex in NMR buffer, 100%D_2O, 600 MHz, 25 °C.

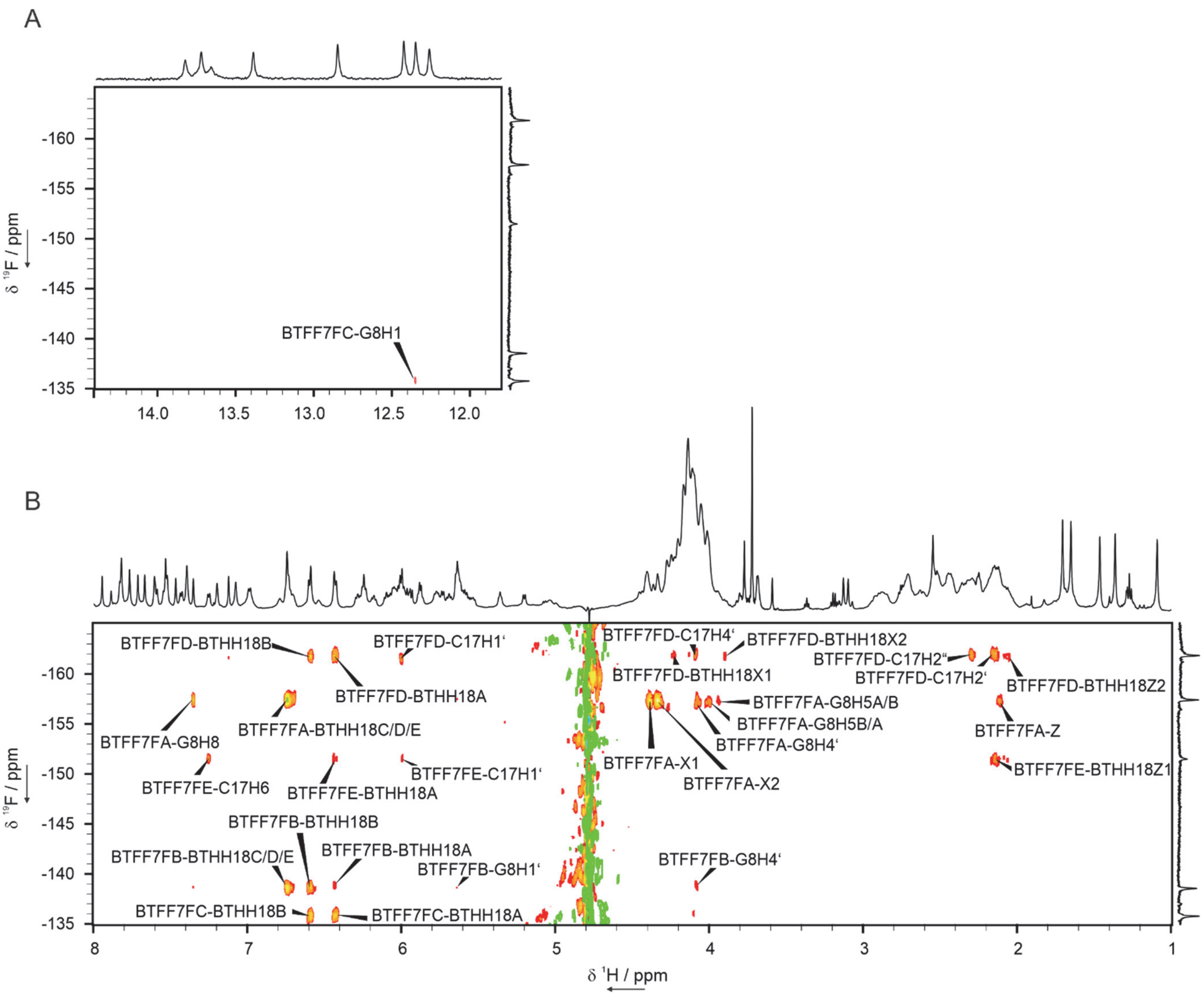

Figure S11 Imino (A) and aromatic/sugar region (B) of the 2D $^{1}H,^{19}F$ HOESY spectrum of BTFF/BTHH duplex with assignment. Spectra recorded on 1.0 mM DNA duplex in NMR buffer, 90%H_2O/10%D_2O, 600 MHz, 25 °C.

^{1}H CSPs DNA

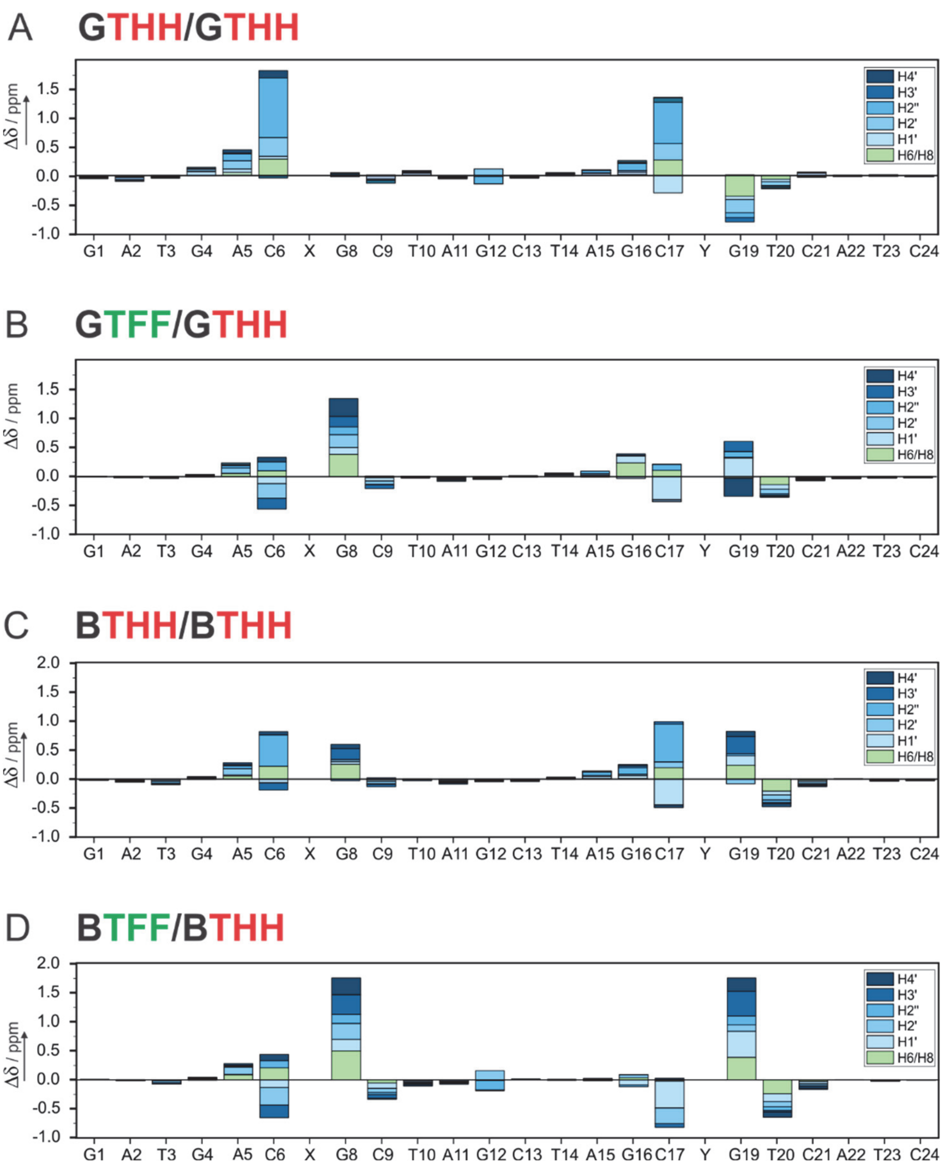

Figure S12 Chemical shift perturbation (CSP, Δδ) analysis of base and sugar ^{1}H for the duplexes A) GTHH/GTHH, B) GTFF/GTHH, C) BTHH/BTHH, D) BTFF/BTHH. $\Delta\delta = (^1\text{H c.s.}_{X/Y}) - (^1\text{H c.s.}_{T/A})$.

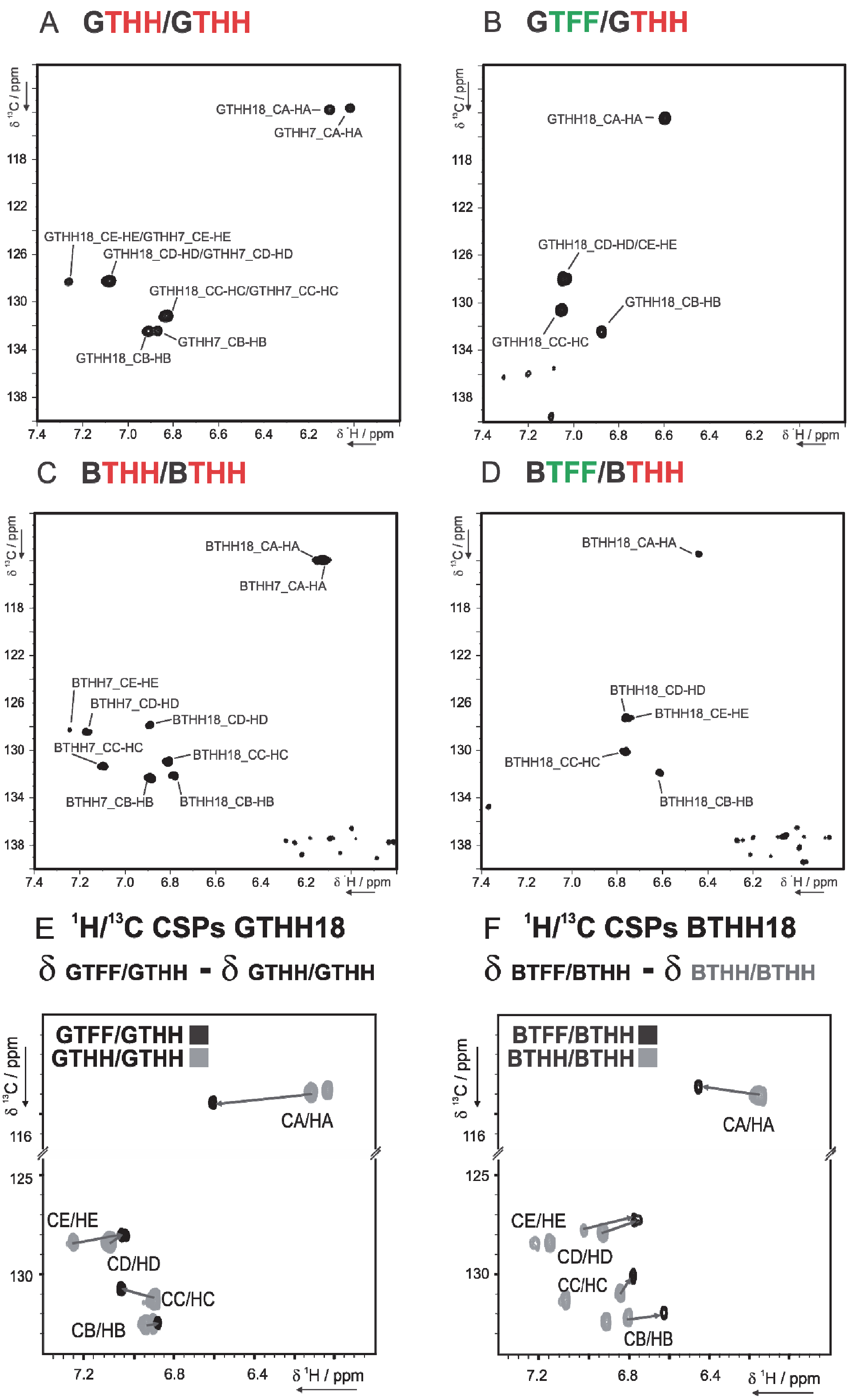

Figure S13 Tolane region of the $^{1}H,^{13}C$ HSQC spectrum recorded on the duplexes A) GTHH/GTHH, B) GTFF/GTHH, C) BTHH/BTHH, D) BTFF/BTHH with assignment. Spectra recorded on 1.0 mM DNA duplex in NMR buffer, 90%H_2O/10%D_2O (A,B) or 100%D_2O (C,D), 600 MHz, 10 °C. Spectra A,B were recorded with a F1 FID resolution of 178Hz without folding of other signals; spectra C,D were recorded with a F1 FID resolution of 115 Hz and contain sugar C1'-H1' folded signals. E) Combined $^{1}H/^{13}C$ CSP of the tolane unit THH18 in GTFF/GTHH compared to GTHH/GTHH, represented as dark gray arrows on the overlay of the aromatic region of the $^{1}H,^{13}C$ HSQC of GTHH/GTHH (gray spectrum) and GTFF/GTHH (black spectrum). F) Same as in E) but with BuNA backbone.

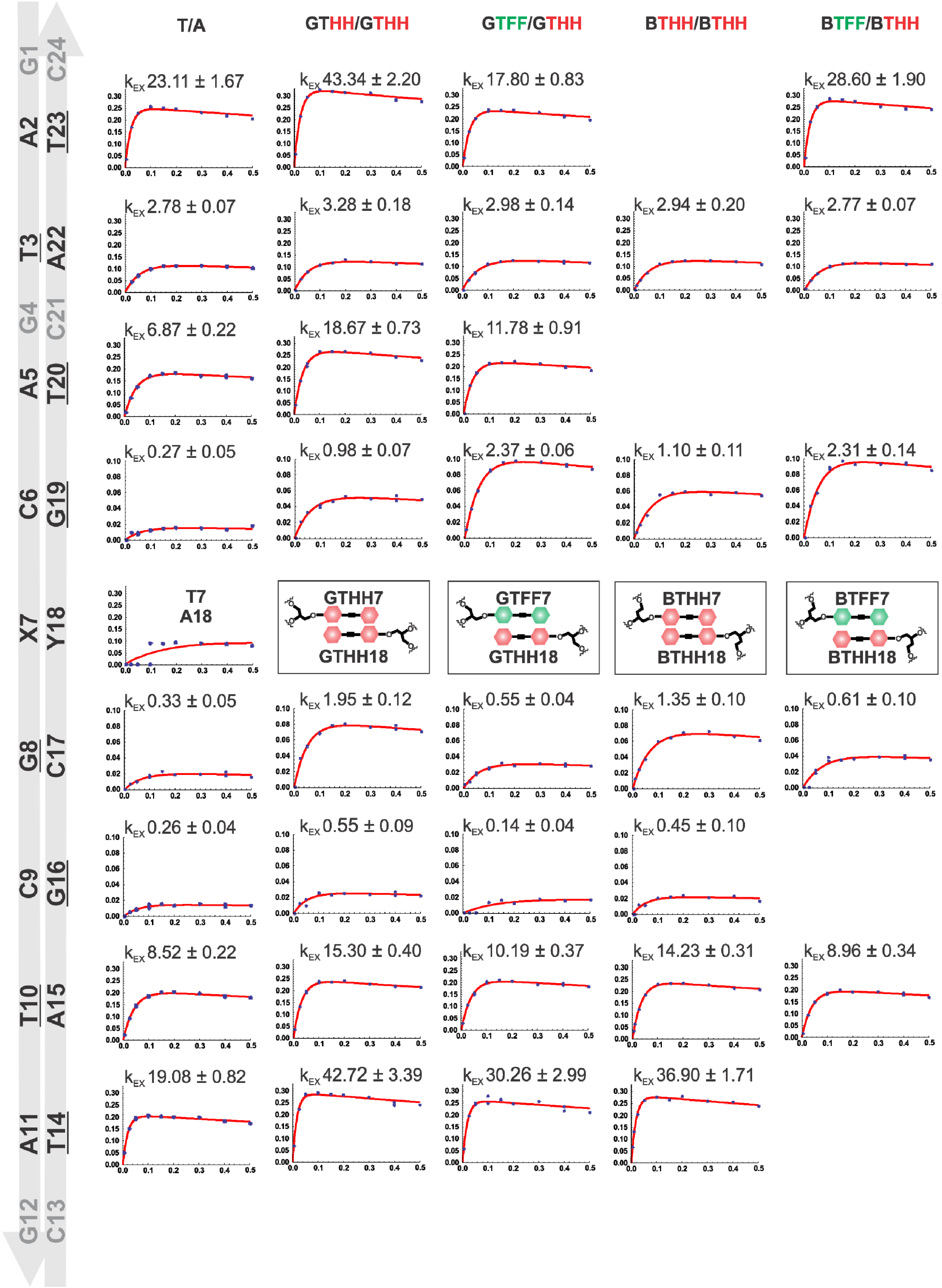

Figure S14 Relative peak intensities of the imino proton NMR signals as a function of the mixing time after water inversion in the CLEANEX-PM experiments. Plots are arranged in rows, as a function of the imino-containing residue position along the duplex, and in columns, according to the different duplex (label on top). The sequence is reported on the left. The residues whose imino proton-water k_{EX} could not be determined due to too slow exchange (G4 in all the duplexes; G16 in BTFF7/BTHH18) or to the peak broadening beyond detection at 25 °C (G1 and G12 in all the duplexes) are gray. The imino proton-water k_{EX} of residue T20 and T23 in BTHH7/BTHH18 as well as T20 and T14 in BTFF7/BTHH18 could not be determined due to peak overlap. The best fit to Eq. 12 is depicted as a solid red line. Results of the fitting are reported on top of each graph (error from the fitting).

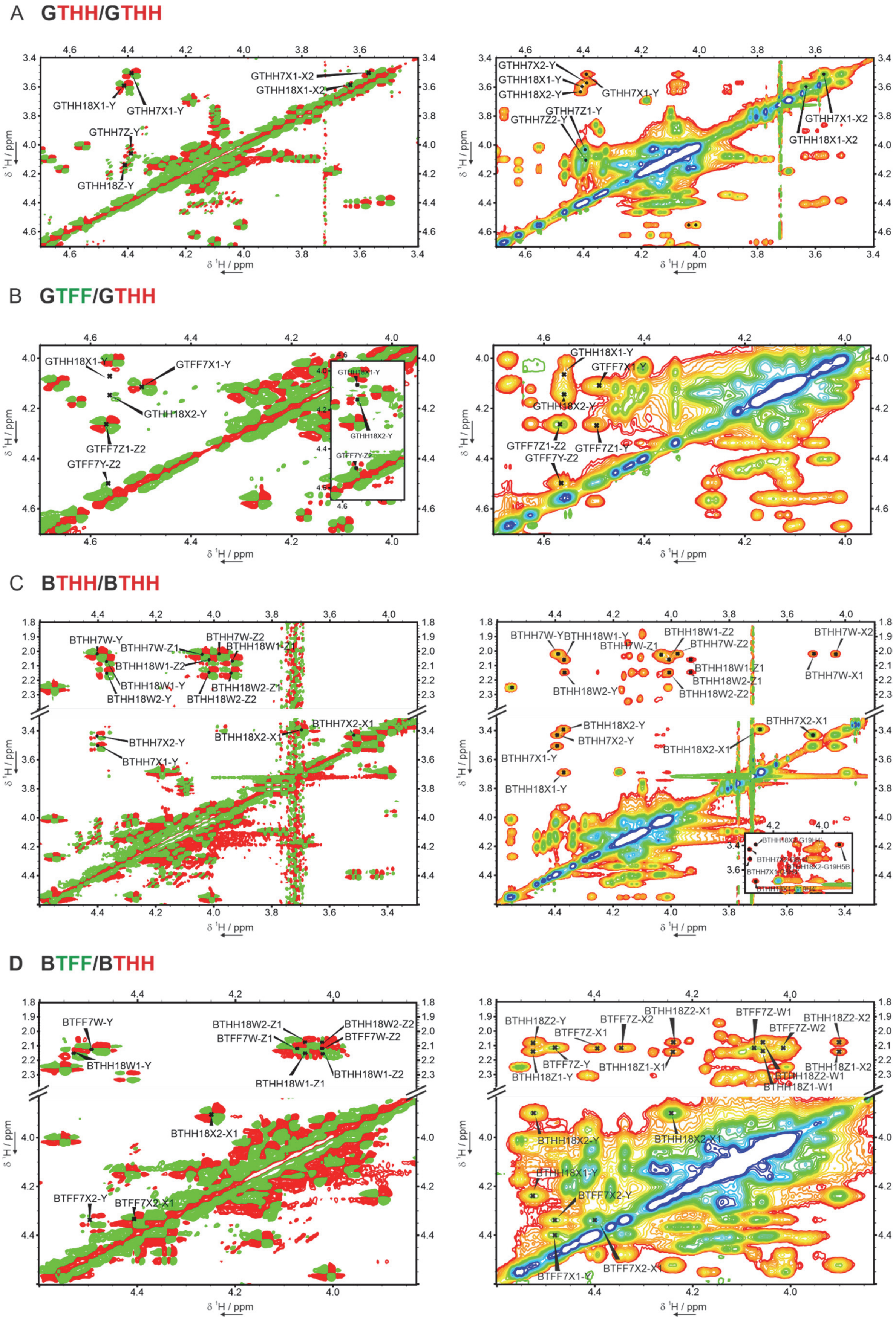

Figure S15 Acyclic backbone region of ^{1}H,^{1}H DQF-COSY (left) and ^{1}H,^{1}H NOESY (right) spectra recorded on the duplexes A) GTHH/GTHH, B) GTFF/GTHH, C) BTHH/BTHH, D) BTFF/BTHH. Spectra recorded on 1.0 mM DNA duplex in NMR buffer, 100%D_2O, 600 MHz, 25 °C.

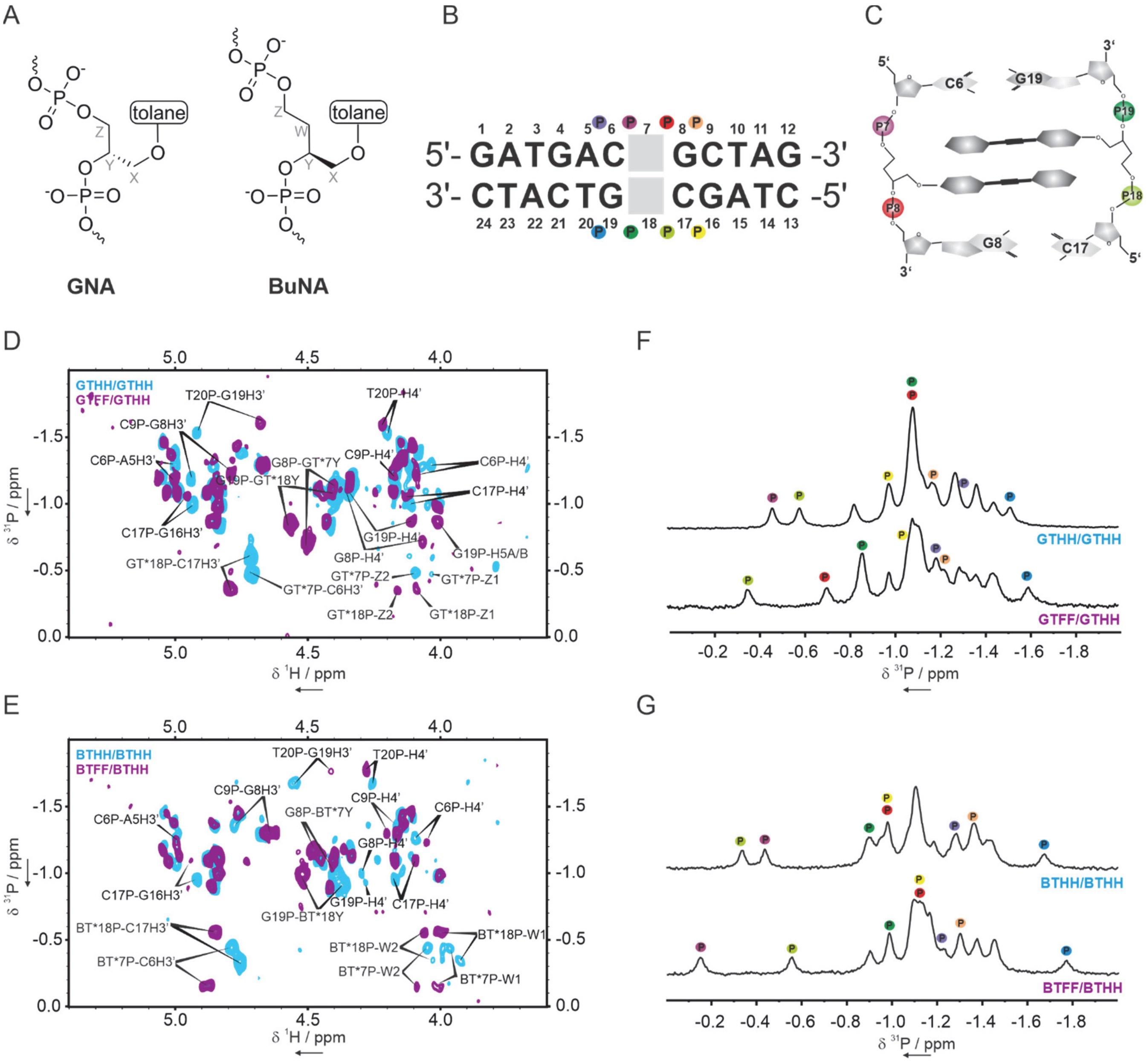

Figure S16 Assignment of the ^{31}P NMR spectra. Letter code, color code and numbering used for labeling acyclic backbone (A) and phosphate groups (B-C). Overlay of the ^{1}H,^{31}P HSQC of D) GTHH/GTHH (cyan) and GTFF/GTHH (purple) duplex and E) BTHH/BTHH (cyan) and BTFF/BTHH (purple) duplex, with H3'(n-1)-P(n) and H4'(n)-P(n) assignment indicated only for phosphate residues 6-9 and 17-20. Overlay of the 1D ^{31}P NMR spectrum of F) GTHH/GTHH (top) and GTFF/GTHH (bottom), and F) BTHH/BTHH (top) and BTFF/BTHH (bottom), with assignment of the phosphate residues 6-9 and 17-20. Spectra recorded on 1.0 mM DNA duplex in NMR buffer, 100%D_2O, 600 MHz, 25 °C. We note that P7 in GTFF/GTHH could not be assigned because the correlations to the glycerol backbone Z protons and to C6 H3' in the 1H,31P HSQC are missing. This can be due either to a particularly small ^{3}JHP or to a highly dynamic backbone. In either case, GTFF/GTHH P7 has a peculiar behavior. Overall, the GTFF/GTHH combination produced the most significant perturbation on the phosphate backbone compared to the reference duplex.

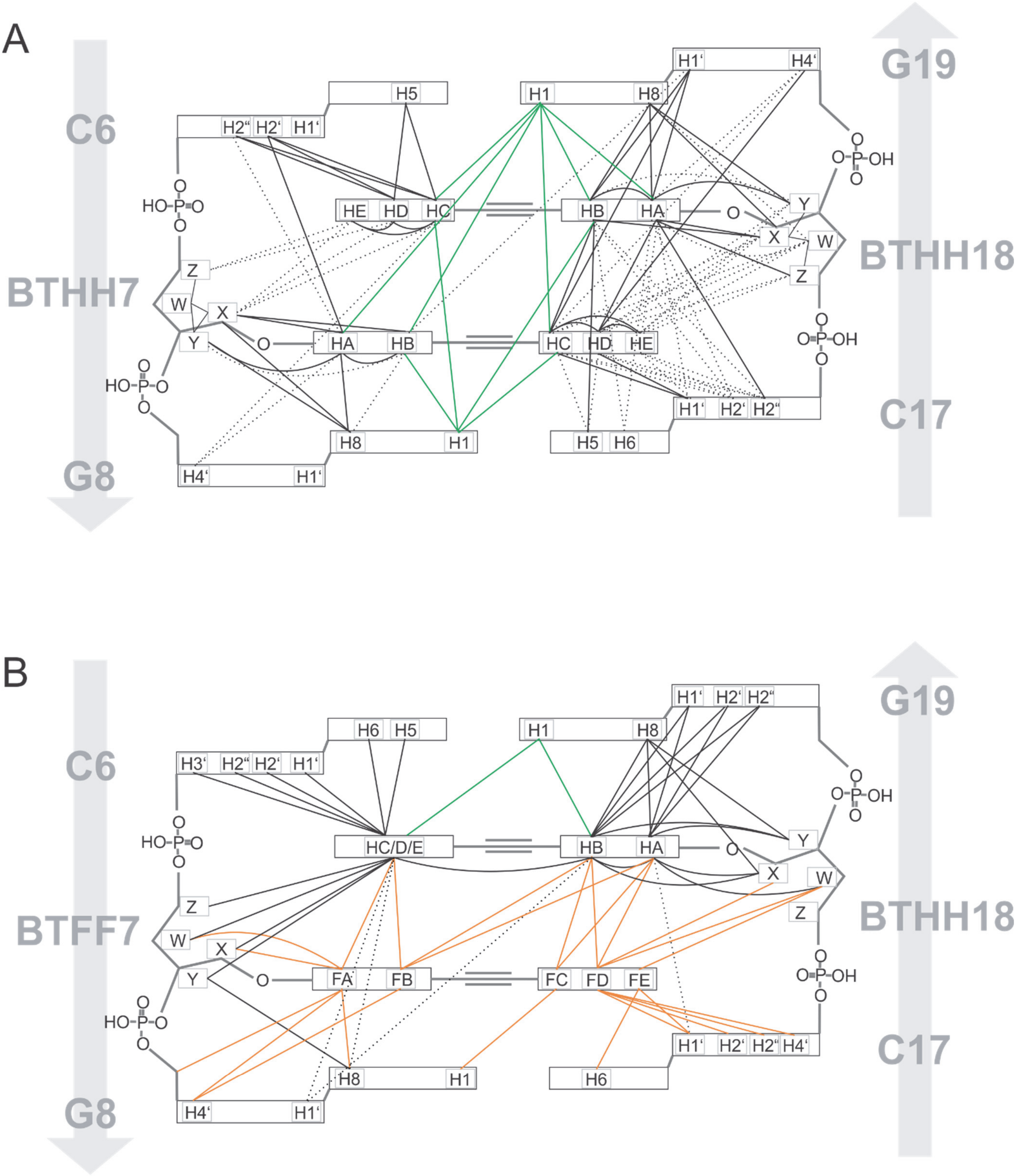

— contacts from NOESY in 10%H_2O/90%D_2O (irrespective of intensity)

— contacts from NOESY in 100%D_2O (full line → medium/strong, dotted line → weak)

— contacts from ^{19}F,^{1}H HOESY in 10%H_2O/90%D_2O (irrespective of intensity)

Figure S17 A) Schematics of the tolane NOE contacts detected for BTHH/BTHH. B) Schematics of the tolane NOE and hetero-NOE contacts detected for BTFF/BTHH. Color code explained in the legend on the bottom. C6 H1' and G8 H1' resonances in BTHH/BTHH are overlapped and the ambiguous cross peaks observed to BTHH7 HA and BTHH18 HC/D are not indicated. NOESY spectra recorded with 200 ms mixing time on 1.0 mM DNA duplex in NMR buffer, 100%D_2O, 600 MHz, 25 °C.

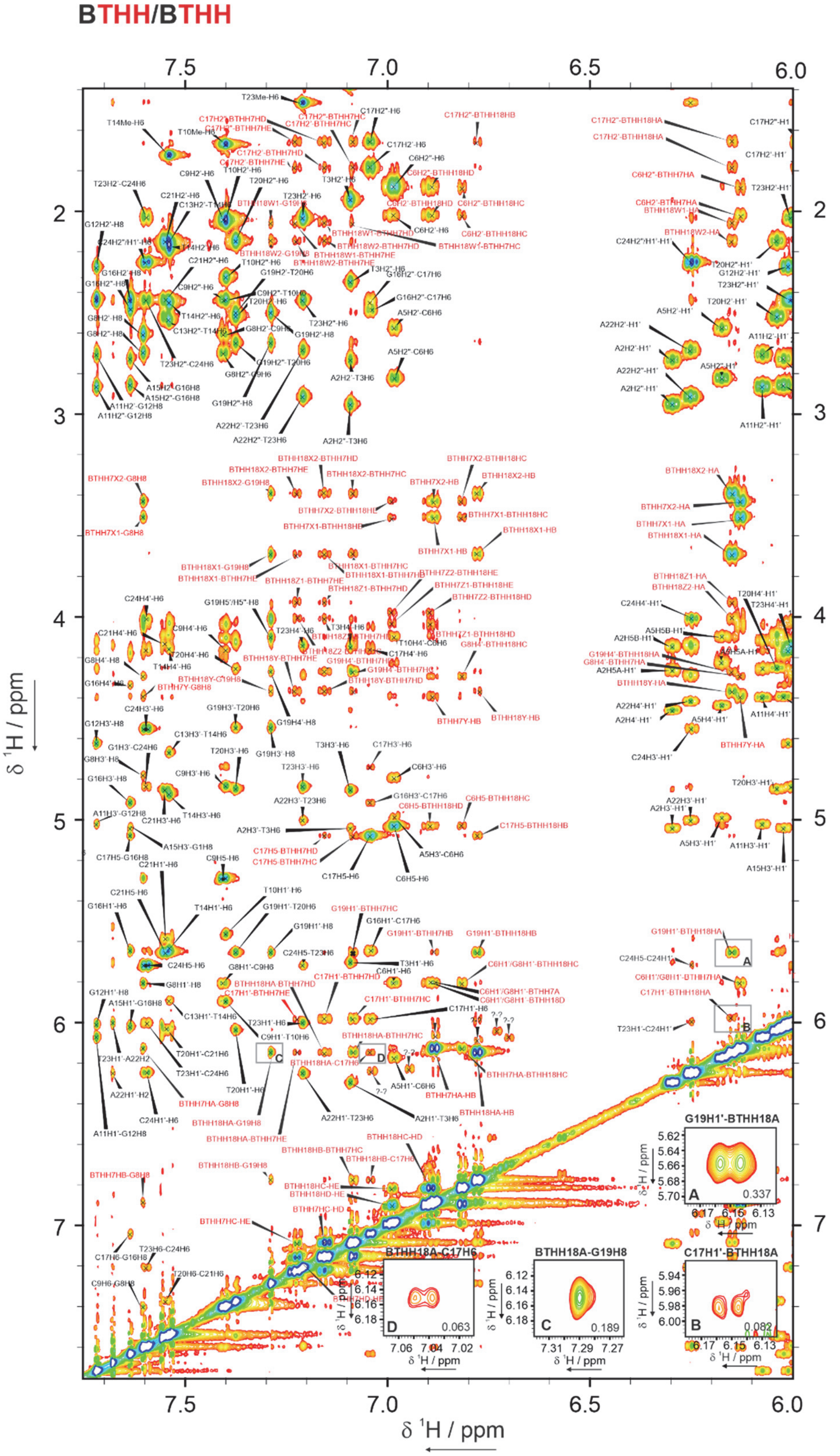

Figure S18
Aromatic region of the NOESY spectrum of BTHH/BTHH 1 mM in 100% D_2O (600 MHz, 25 °C) with assignment. Cross peaks with the tolane aromatic protons are highlighted in red. Spectrum shown at lowest positive contour level adjusted to be comparable with spectrum shown in Figure S17. The cross peaks boxed in gray, corresponding to NOE interaction of BTHH18 HA with aromatic and anomeric protons of its adjacent residues (i.e., C17 and G19), did not suffer from overlap with other peaks and were suitable for integration. A zoom on the selected cross peaks is shown on the bottom right corner of the spectrum. The integral of each peak, normalized to the integral of peak C9_H5-H6, is shown in the bottom right corner of the corresponding spectrum excerpt. See for comparison the corresponding cross peaks in BTFF/BTHH (Figure S19).

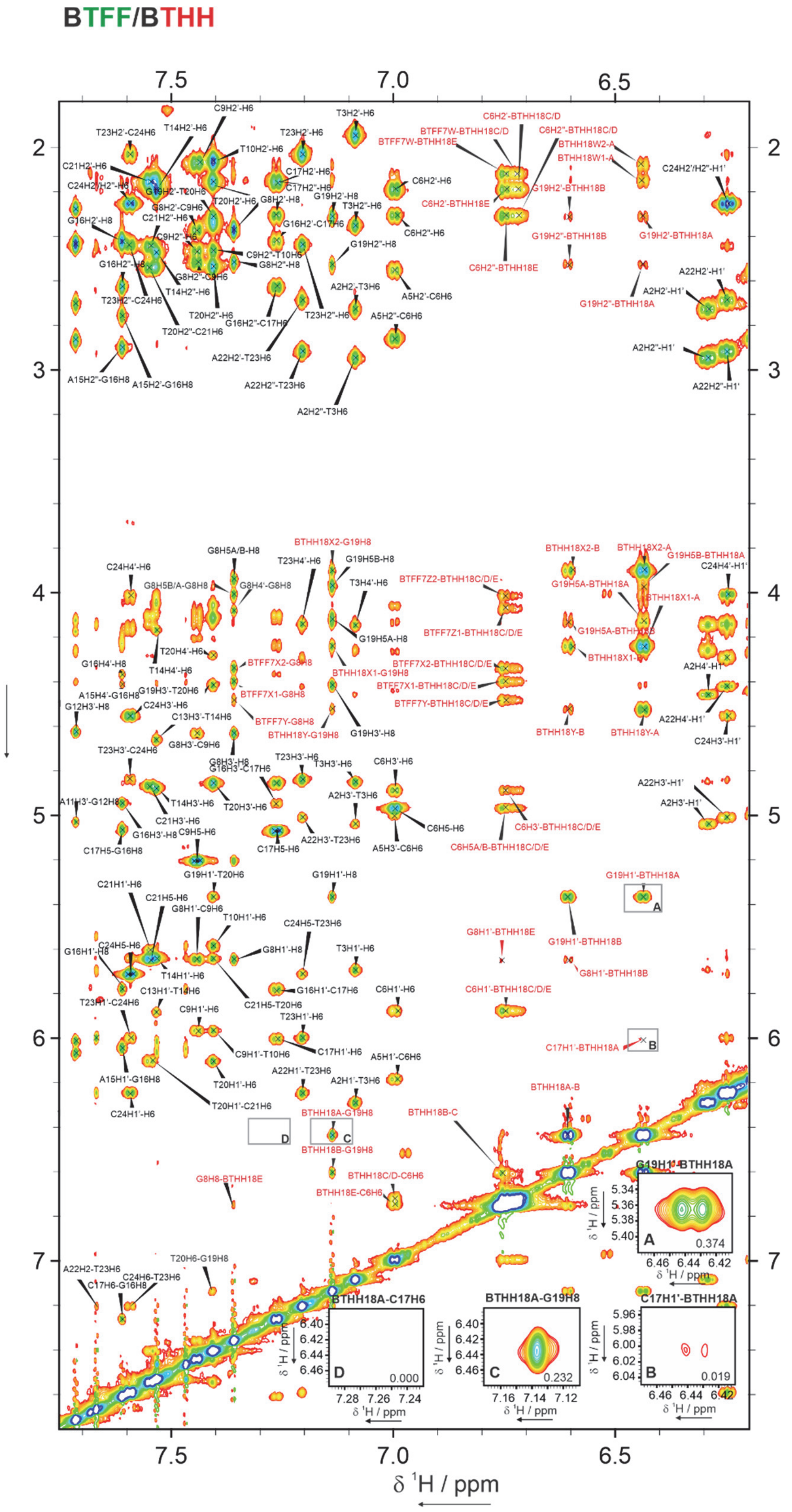

Figure S19
Aromatic region of the NOESY spectrum of BTFF/BTHH 1 mM in 100% D_2O (600 MHz, 25 °C) with assignment. Cross peaks with the tolane aromatic protons are highlighted in red. Spectrum shown at lowest positive contour level adjusted to be comparable with spectrum shown in Figure S16. The cross peaks boxed in gray, corresponding to NOE interaction of BTHH18 HA with aromatic and anomeric protons of its adjacent residues (i.e., C17 and G19), did not suffer from overlap with other peaks and were suitable for integration. A zoom on the selected cross peaks is shown on the bottom right corner of the spectrum. The integral of each peak, normalized to the integral of peak C9_H5-H6, is shown in the bottom right corner of the corresponding spectrum excerpt. See for comparison the corresponding cross peaks in BTHH/BTHH (Figure S18).

Experimental Procedures

General Material and Methods

All standard chemicals were purchased from commercial suppliers. D-(+)-solketal = (*S*)-(+)-2,2-dimethyl-1,3-dioxolane-4-methanol, trimethylsilylacetylene, (*R*)-(+)-glycidol were obtained from Tokyo Chemical Industry. Iodopentafluorobenzene, 4-iodophenol, L-malic acid, borane dimethyl sulfide complex solution (2 M in THF), trimethyl borate, benzaldehyde dimethyl acetal and tetrakis(triphenylphosphine)palladium(0) were purchased from Sigma Aldrich. 4,4'-Dimethoxytrityl chloride and 2-cyanoethyl *N,N*-diisopropylchlorophosphoramidite were obtained from Chem Genes Corporation. Triphenylphosphine was obtained from Acros Organics and diisopropyl azodicarboxylate was purchased from Alfa Aesar.

4-(2-Phenylethynyl)phenol (**1**)[1] as well as the backbone precursors (*S*)-DMT-*O*-glycidol (**2**)[2] and 4-(*S*)-hydroxymethyl-2-phenyl-1,3-dioxan (**3**)[3] were synthesized according to previously published protocols.

Dry solvents DCM and THF were obtained via a solvent purification system (SPS) from Inert Corporation. Pyridine for DMT-protection was obtained from Acros Organics and dried over activated molecular sieves. Solvents for column chromatography were used in technical quality and distilled prior to use. All other organic solvents were used in pro analysis or for synthesis quality without further purification.

Column chromatography was carried out on silica gel (Kieselgel 60, Merck, 0.063 – 0.200 mm).Thin layer chromatography (TLC) was performed on aluminum-backed plates coated with silica gel and a fluorescent indicator (Alugram SIL G/UV254, Macherey-Nagel, UV visualization, 254 nm). For UV-inactive substances a cerium molybdate stain was used.

NMR-Spectroscopy and mass spectrometry

^{1}H-, ^{13}C-, ^{19}F- and ^{31}P-NMR spectra were recorded on a Bruker Avance HD III spectrometer at 400 MHz. Spectra were calibrated to the residual solvent peak from $CDCl_3$ (δ = 7.26 (^{1}H) and δ = 77.16 (^{13}C)) and DMSO-d_6 (δ = 2.50 (^{1}H) and δ = 39.52 (^{13}C)). Other nuclei were referenced on the unified scale. Chemical shifts δ are reported in ppm and coupling constants *J* are given in Hz. Multiplicities are denoted as follows: s (singlet), d (doublet), t (triplet), q (quartet), p (pentet), dd (doublet of doublet), td (triplet of doublet), m (multiplet), br (broad).

NMR spectra of building blocks were evaluated with MestReNova v12.0.4.

High resolution ESI mass spectra were measured on a Bruker micrOTOF-Q III spectrometer.

Synthetic procedures

Compound **S1**

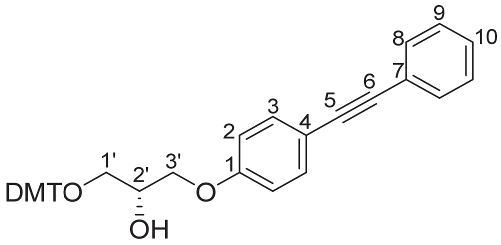

Under nitrogen atmosphere, 4-(2-phenylethynyl)phenol (**1**) (500 mg, 2.57 mmol, 1.12 eq.) and sodium hydride (60% oil dispersion, 20.6 mg, 515 µmol, 0.20 eq.) were dissolved in anhydrous DMF (6 mL) and stirred at ambient temperature for 2 h. A solution of (*S*)-DMT-*O*-glycidol (**2**) (864 mg, 2.29 mmol. 1.00 eq.) in anhydrous DMF (8 mL) was added and the mixture was stirred for 1 d at 110°C. The solvent was removed under reduced pressure and the residue was purified by column chromatography (hexane/EtOAc 2:1 + 3% Et_3N –1:1 + 1% Et_3N) to afford compound **S1** (863 mg, 9.01 mmol, 60%) as a yellow foam.

^{1}H NMR (400 MHz, $CDCl_3$): *δ* (ppm) = 7.54 – 7.49 (m, 2H, 8-H), 7.48 – 7.44 (m, 2H, 3-H), 7.44 – 7.40 (m, 2H, DMT-H), 7.37 – 7.26 (m, 9H, 9-H, 10-H, DMT-H), 7.25 – 7.18 (m, 1H, DMT-H), 6.88 – 6.84 (m, 2H, 2-H), 6.84 – 6.79 (m, 4H, DMT-H), 4.18 – 4.10 (m, 1H, 2'-H), 4.11 – 4.01 (m, 2H, 3'-H), 3.79 (s, 6H, DMT-OMe-H), 3.40 – 3.30 (m, 2H, 1'-H), 2.44 (d, *J* = 5.3 Hz, 1H, 2'-OH);

^{13}C{^{1}H} NMR (100 MHz, $CDCl_3$): *δ* (ppm) = 158.69 (1-C), 158.68 (DMT-C), 144.81 (DMT-C), 135.93 (DMT-C), 135.91 (DMT-C), 133.19 (3-C), 131.60 (8-C), 130.17 (DMT-C), 128.46 (DMT-C), 128.20 (DMT-C), 128.13 (10-C), 128.04 (9-C), 127.02 (DMT-C), 123.65 (7-C), 115.91 (4-C), 114.74 (2-C), 113.31 (DMT-C), 89.38 (5-C), 88.35 (6-C), 86.45 (DMT-C), 69.58 (2'-C), 69.21 (3'C), 64.04 (1'-C), 55.37 (DMT-C);

HR-MS (ESI+): *m*/*z* calc. ($C_{38}H_{34}O_5Na$, $[M+Na]^+$): 593.22984, found: 593.23111

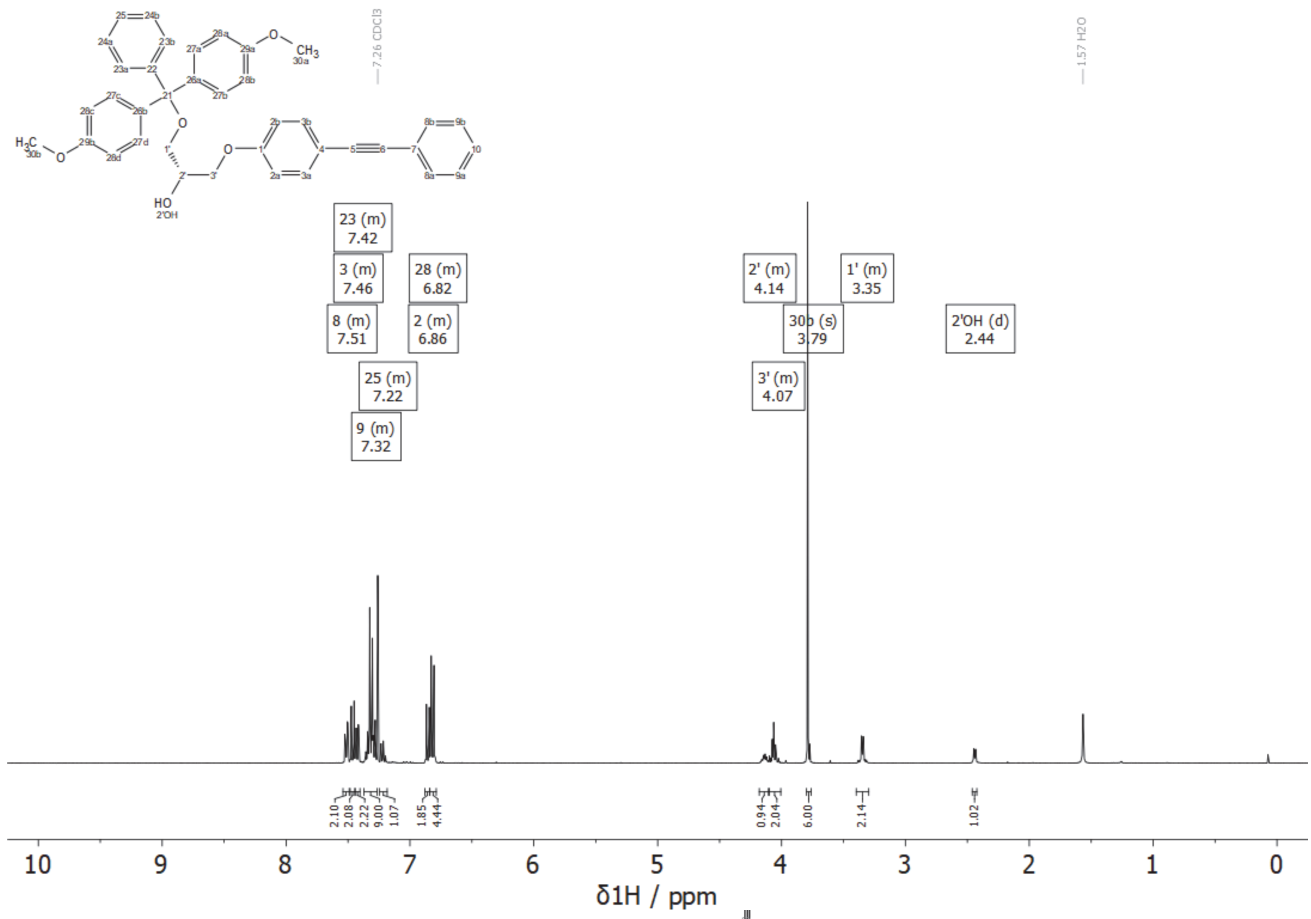
7.26 CDCl3
1.57 H2O
23 (m) 7.42
3 (m) 7.46
28 (m) 6.82
8 (m) 7.51
2 (m) 6.86
25 (m) 7.22
9 (m) 7.32
2' (m) 4.14
1' (m) 3.35
30b (s) 3.79
2'OH (d) 2.44
3' (m) 4.07
2.10
2.08
2.22
9.00
1.07
1.85
4.44
0.94
2.04
6.00
2.14
1.02
δ1H / ppm

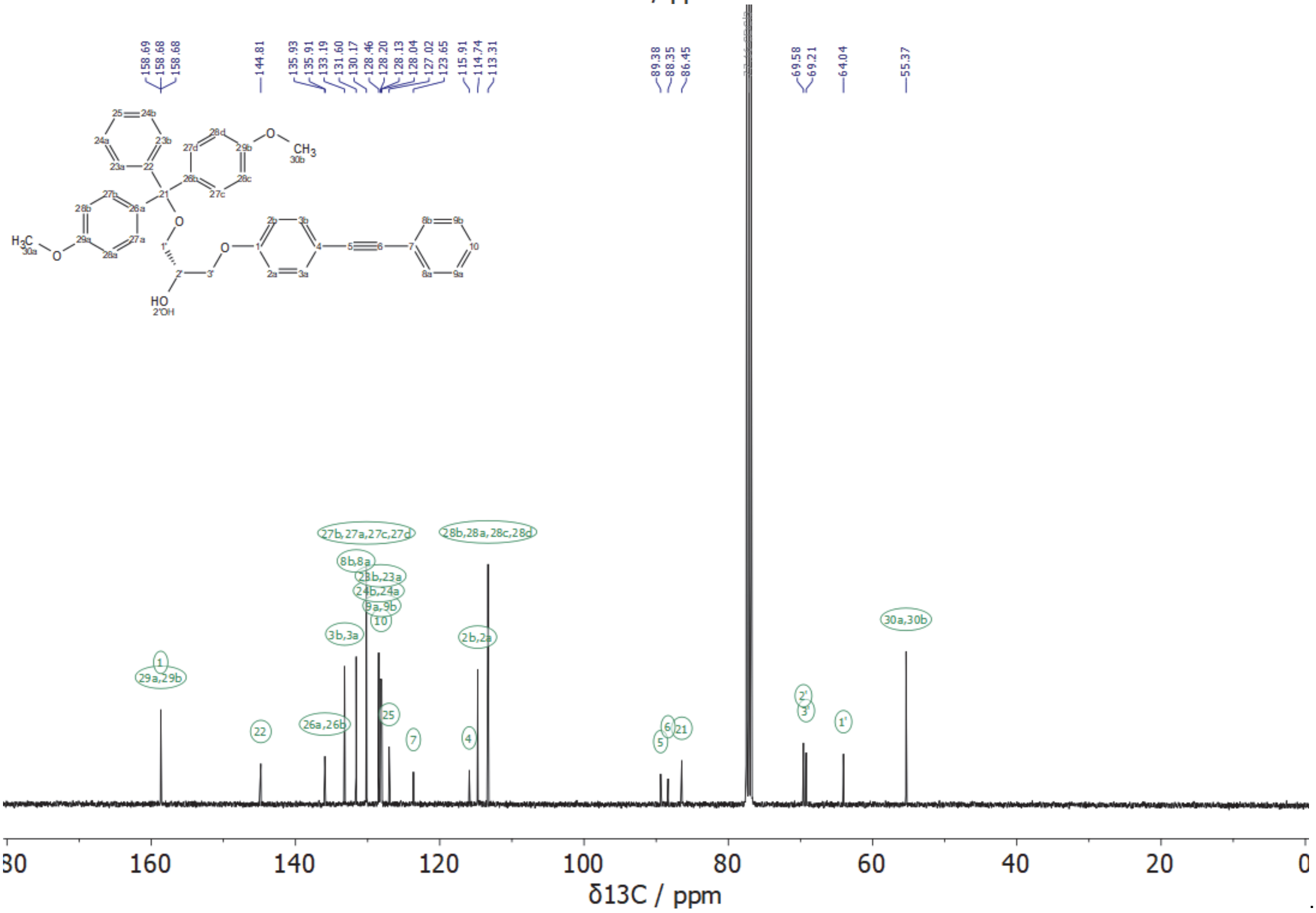
158.69
158.68
158.68
144.81
135.93
135.91
133.19
131.60
130.17
128.46
128.20
128.13
128.04
127.02
123.65
115.91
114.74
113.31
89.38
88.35
86.45
69.58
69.21
64.04
55.37
δ13C / ppm

Compound **S2** = GTHH-PA

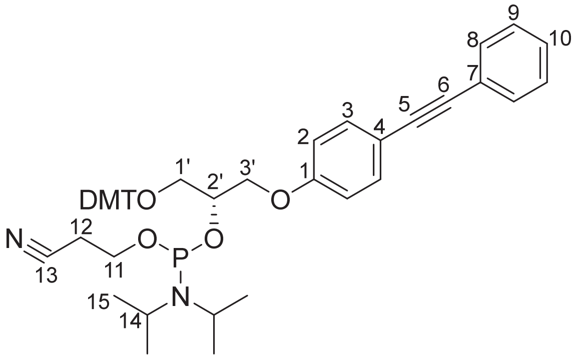

Compound **S1** (340 mg, 596 µmol, 1.00 eq.) was dissolved with DIPEA (596 µL, 442 mg, 3.42 mmol, 5.74 eq.) in anhydrous DCM (15 mL). After 10 min CEP-Cl (173 µL, 773 µmol, 1.30 eq.) was added. Additional CEP-Cl (17.0 µL, 18.3 mg, 77.3 µmol, 0.13 eq.) was added after 2 h stirring at room temperature. The reaction mixture was stirred at ambient temperature for additionally 4 h. The solvent was removed under reduced pressure and the residue was purified by column chromatography (DCM/hexane 3:1 + 1% Et_3N) to afford compound **S2** as a colorless foam (358 mg, 464 µmol, 78%).

^{1}H NMR (400 MHz, $CDCl_3$): *δ* (ppm) = 7.54 – 7.49 (m, 4H, 8-H), 7.49 – 7.38 (m, 8H, 3-H, DMT-H), 7.39 – 7.10 (m, 20H, 9-H, 10-H, DMT-H), 6.92 – 6.74 (m, 12H, 2-H, DMT-H), 4.39 – 4.23 (m, 3H, 2'-H, 3'-H), 4.19 – 4.03 (m, 3H, 3'-H), 3.91 – 3.67 (m, 16H, 11-H; DMT-H), 3.66 – 3.47 (m, 4H, 14-H), 3.42 – 3.20 (m, 4H, 1'-H), 2.72 – 2.37 (m, 4H, 12-H), 1.25 – 0.98 (m, 24H, 15-H);

^{13}C{^{1}H} NMR (100 MHz, $CDCl_3$): *δ* (ppm) = 158.89 (1-C), 158.85 (1-C), 158.60 (DMT-C), 158.59 (DMT-C), 144.92 (DMT-C), 144.90 (DMT-C), 136.12 (DMT-C), 136.09 (DMT-C), 136.07 (DMT-C), 133.19 (3-C), 133.13 (3-C), 131.59 (8-C), 130.25 (DMT-C), 130.20 (DMT-C), 130.17 (DMT-C), 128.45, 128.33, 128.26, 128.08, 127.93, 126.93 (DMT-C), 126.89 (DMT-C), 123.69 (7-C), 117.86 (13-C), 117.75 (13-C), 115.65 (4-C), 115.59 (4-C), 114.79 (2-C), 114.72 (2-C), 113.20 (DMT-C), 89.47 (5-C), 88.27 (6-C), 88.24 (6-C), 86.29 (DMT-C), 86.25 (DMT-C), 72.21 (3'-C), 72.03 (3'-C), 71.82 (3'-C), 71.65 (3'-C), 68.98 (2'-C), 68.95 (2'-C), 68.74 (2'-C), 68.70 (2'-C), 63.76 (1'-C), 63.72 (1'-C), 63.67 (1'-C), 63.64 (1'-C), 58.67 (11-C), 58.59 (11-C), 58.49 (11-C), 58.41 (11-C), 55.36 (DMT-C), 55.35 (DMT-C), 43.41 (14-C), 43.37 (14-C), 43.29 (14-C), 43.24 (14-C), 24.85 (15-C), 24.83 (15-C), 24.78 (15-C), 24.75 (15-C), 24.71 (15-C), 24.64 (15-C), 24.57 (15-C), 20.50 (12-C), 20.44 (12-C), 20.37 (12-C), 20.30 (12-C);

^{31}P NMR (162 MHz, $CDCl_3$): *δ* (ppm) = 149.87, 149.61;

HR-MS (ESI+): *m/z* calc. ($C_{47}H_{51}O_6N_2Na$, $[M+Na]^+$): 793.33769, found: 793.33746.

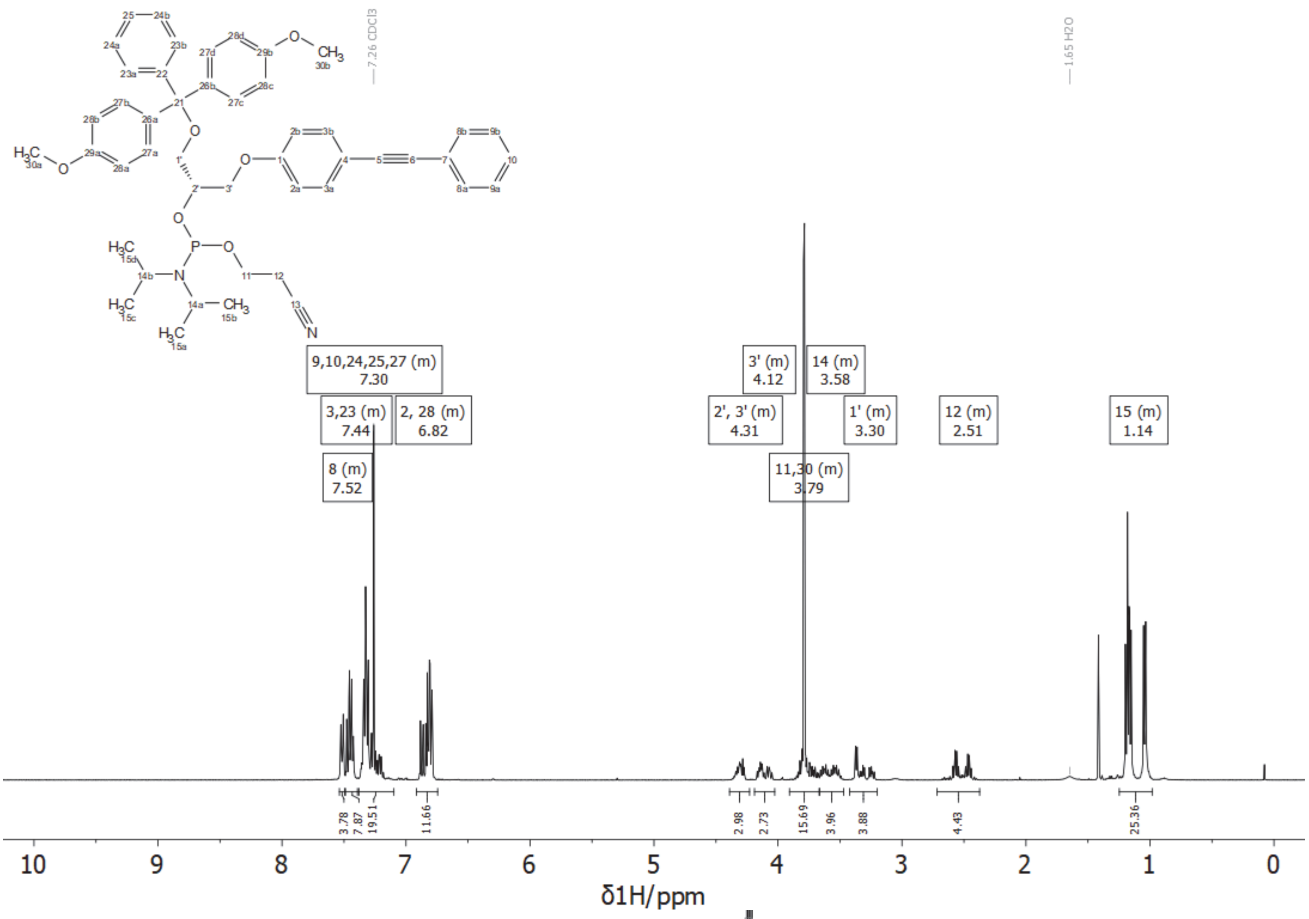
7.26 CDCl3
1.65 H2O
9,10,24,25,27 (m) 7.30
3,23 (m) 7.44
2, 28 (m) 6.82
8 (m) 7.52
3' (m) 4.12
14 (m) 3.58
2', 3' (m) 4.31
1' (m) 3.30
11,30 (m) 3.79
12 (m) 2.51
15 (m) 1.14
3.78
7.87
19.51
11.66
2.98
2.73
15.69
3.96
3.88
4.43
25.36
δ1H/ppm

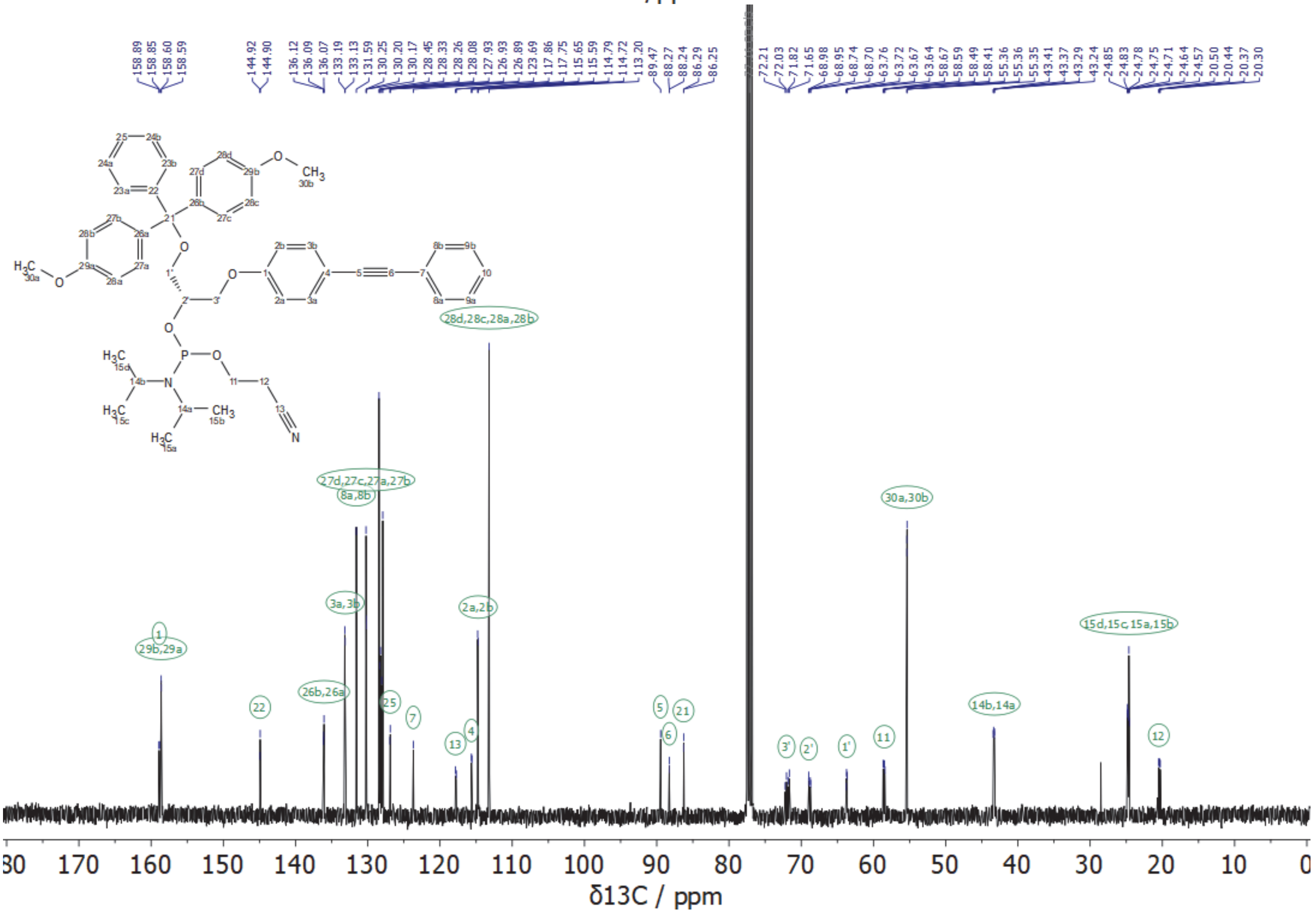
158.89
158.85
158.60
158.59
144.92
144.90
136.12
136.09
136.07
133.19
133.13
131.59
130.25
130.20
130.17
128.45
128.33
128.26
128.08
127.93
126.93
126.89
123.69
117.86
117.75
115.65
115.59
114.79
114.72
113.20
89.47
88.27
88.24
86.29
86.25
72.21
72.03
71.82
71.65
68.98
68.95
68.74
68.70
63.76
63.72
63.67
63.64
58.67
58.59
58.49
58.41
55.36
55.36
55.35
43.41
43.37
43.29
43.24
24.85
24.83
24.78
24.75
24.71
24.64
24.57
20.50
20.44
20.37
20.30
28d,28c,28a,28b
27d,27c,27a,27b
8a,8b
30a,30b
3a,3b
2a,2b
1
29b,29a
15d,15c,15a,15b
22
26b,26a
25
7
4
13
5
6
21
14b,14a
3'
2'
1'
11
12
δ13C / ppm

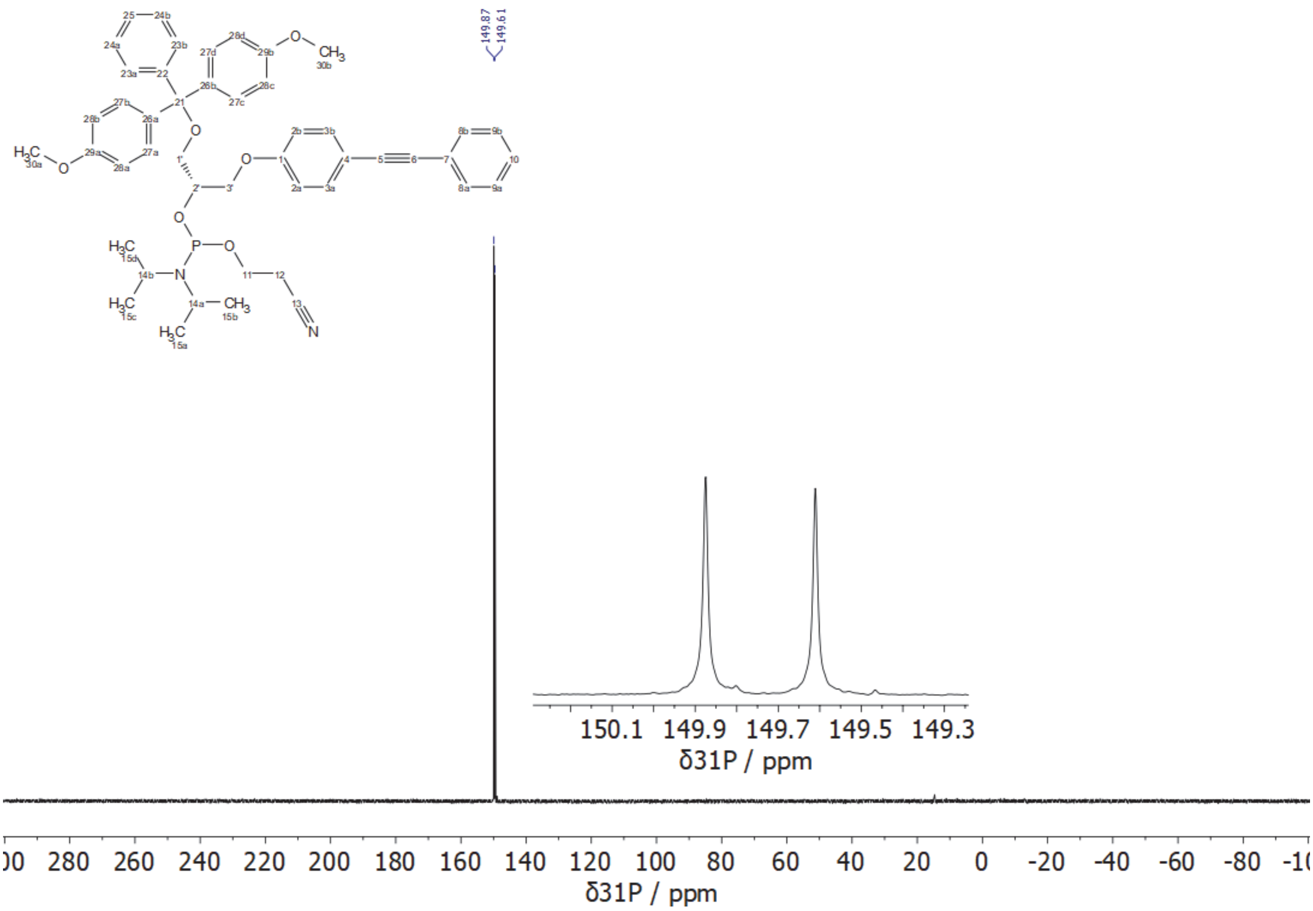

Compound **S3**

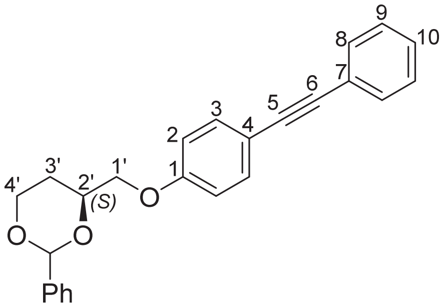

General procedure was adapted from He et. al.[4] 4-(2-Phenylethynyl)phenol (**1**) (500 mg, 2.57 mmol, 1.00 eq.), 4-(*S*)-hydroxmethyl-2-phenyl-1,3-dioxan (**3**) (649 mg, 3.34 mmol, 1.30 eq.) and triphenylphosphine (709 mg, 2.70 mmol, 1.05 eq.) were dissolved in THF (0.9 mL). Diisopropyl azodicarboxylate (530 µL, 2.70 mmol, 1.05 eq.) was added dropwise and the reaction mixture was sonicated for 30 min. Purification of the residue by column chromatography (hexane/DCM 3:2) afforded compound **S3** as a yellow solid (722 mg, 1.95 mmol, 76%).

^{1}H NMR (400 MHz, $CDCl_3$): *δ* (ppm) = 7.54 – 7.49 (m, 4H, 8-H, Ph-H), 7.49 – 7.44 (m, 2H, 3-H), 7.42 – 7.28 (m, 6H, 9-H, 10-H, Ph-H), 6.93 – 6.88 (m, 2H, 2-H), 5.60 (s, 1H, C*H*-Ph), 4.41 – 4.32 (m, 1H), 4.37 – 4.26 (m, 1H), 4.23 – 4.17 (m, 1H), 4.11 – 3.99 (m, 1H), 4.06 – 3.98 (m, 1H), 2.09 – 1.92 (m, 1H), 1.77 – 1.68 (m, 1H);

^{13}C{^{1}H} NMR (100 MHz, $CDCl_3$): *δ* (ppm) = 158.86 (C-1), 138.38 (C-12), 133.19 (C-3), 131.59 (C-8), 129.07, 128.45, 128.44, 128.11, 126.26 (13-C), 123.67 (C-7), 115.85 (C-4), 114.83 (C-2), 101.48(*C*H-Ph), 89.41 (C-5), 88.32 (C-6), 75.44 (C-2'), 70.77 (C-1'), 66.88 (C-4'), 28.31 (C-3');

HR-MS (ESI+): *m/z* calc. ($C_{25}H_{22}NaO_3$, $[M+Na]^+$): 393.14666, found: 393.14680.

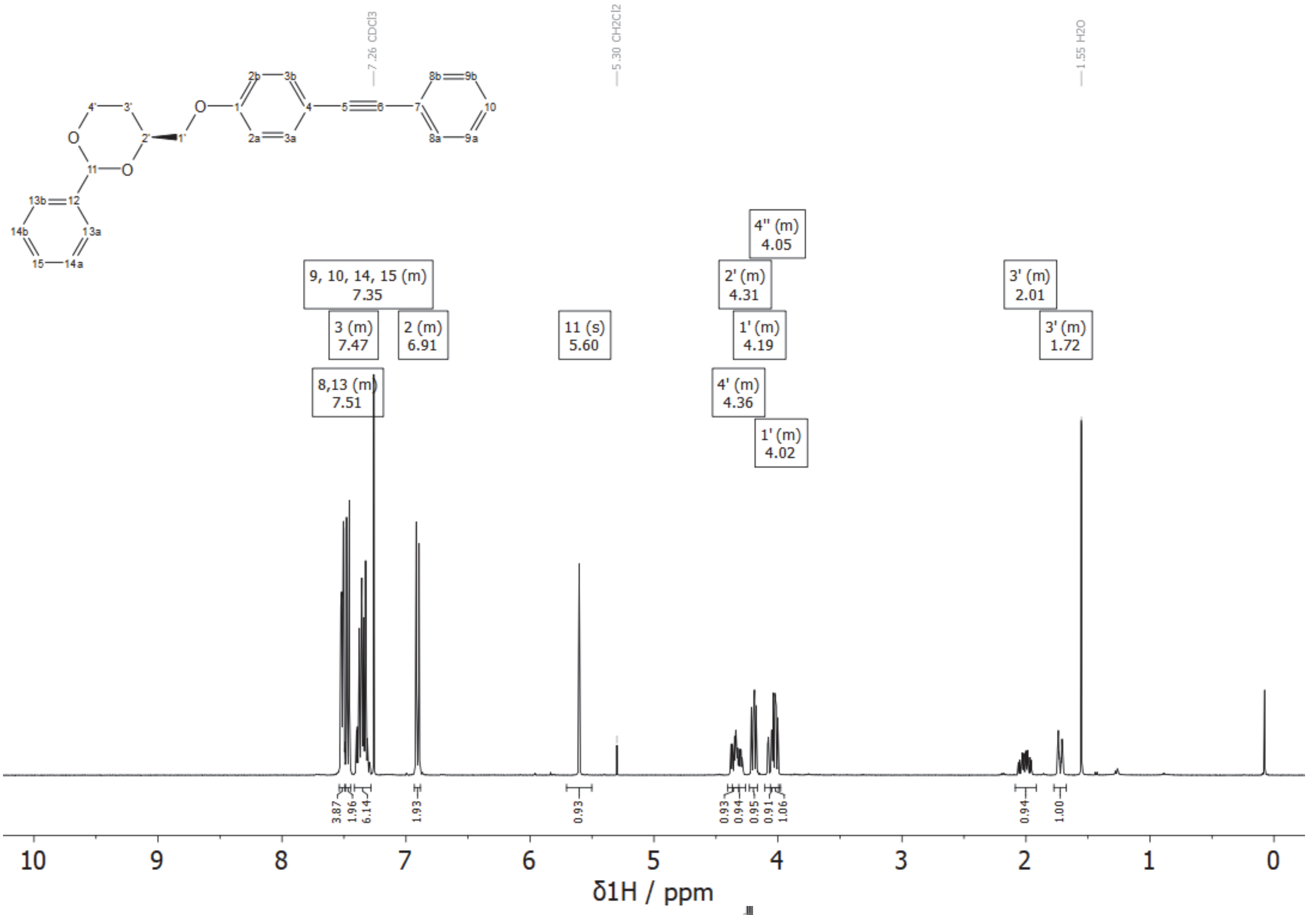

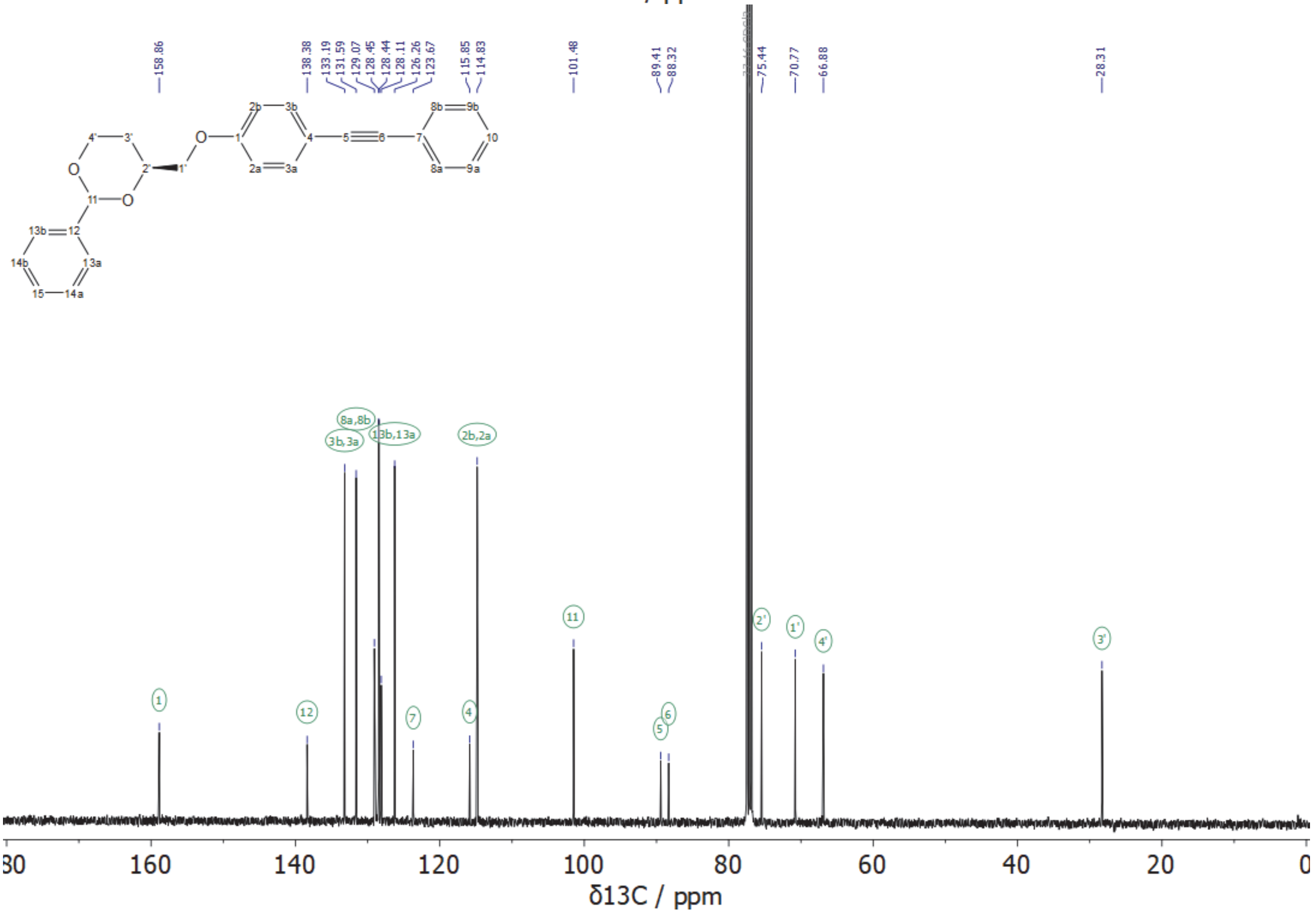

Compound **S4**

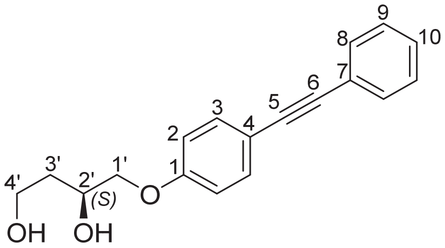

Compound **S3** (650 mg, 1.75 mmol, 1.00 eq.) was dissolved in methanol (24 mL). *p*-Toluenesulfonic acid monohydrate (501 mg, 2.63 mmol, 1.50 eq.) was added and the reaction mixture stirred at ambient temperature for 40 h. Afterwards, the reaction was diluted with a saturated potassium carbonate solution (2.5 mL) and extracted with DCM (4x50 mL). The combined organic layers were dried over Na_2SO_4, evaporated and the solid residue was purified by column chromatography (hexane/EtOAc 1:1–1:2) to afford compound **S4** as a colorless solid (416 mg, 1.47 mmol, 84%).

^{1}H NMR (400 MHz, DMSO-d$_6$): *δ* (ppm) = 7.55 – 7.50 (m, 2H, 8-H), 7.50 – 7.45 (m, 2H, 3-H), 7.44 – 7.36 (m, 3H, 9-H, 10-H), 7.01 – 6.95 (m, 2H, 2-H), 4.87 (d, *J* = 4.8 Hz, 1H, 2'-OH), 4.43 (t, *J* = 5.1 Hz, 1H, 4'-OH), 3.98 – 3.84 (m, 3H, 1'-H, 2'-H), 3.60 – 3.51 (m, 2H, 4'-H), 1.74 – 1.64 (m, 1H, 3'-H), 1.62 – 1.51 (m, 1H, 3'-H);

^{13}C{^{1}H} NMR (100 MHz, DMSO-d$_6$): *δ* (ppm) = 159.10 (1-C), 132.95 (3-C), 131.19 (8-C), 128.74 (9-C), 128.44 (10-C), 122.70 (7-C), 114.96 (2-C), 114.07 (4-C), 89.53 (5-C), 87.95 (6-C), 72.54 (1'-C), 65.85 (2'-C), 57.58 (4'-C), 36.74 (3'-C);

HR-MS (ESI+): *m*/*z* calc. ($C_{18}H_{18}NaO_3$, [M+Na]$^+$): 305.11482, found: 305.11540.

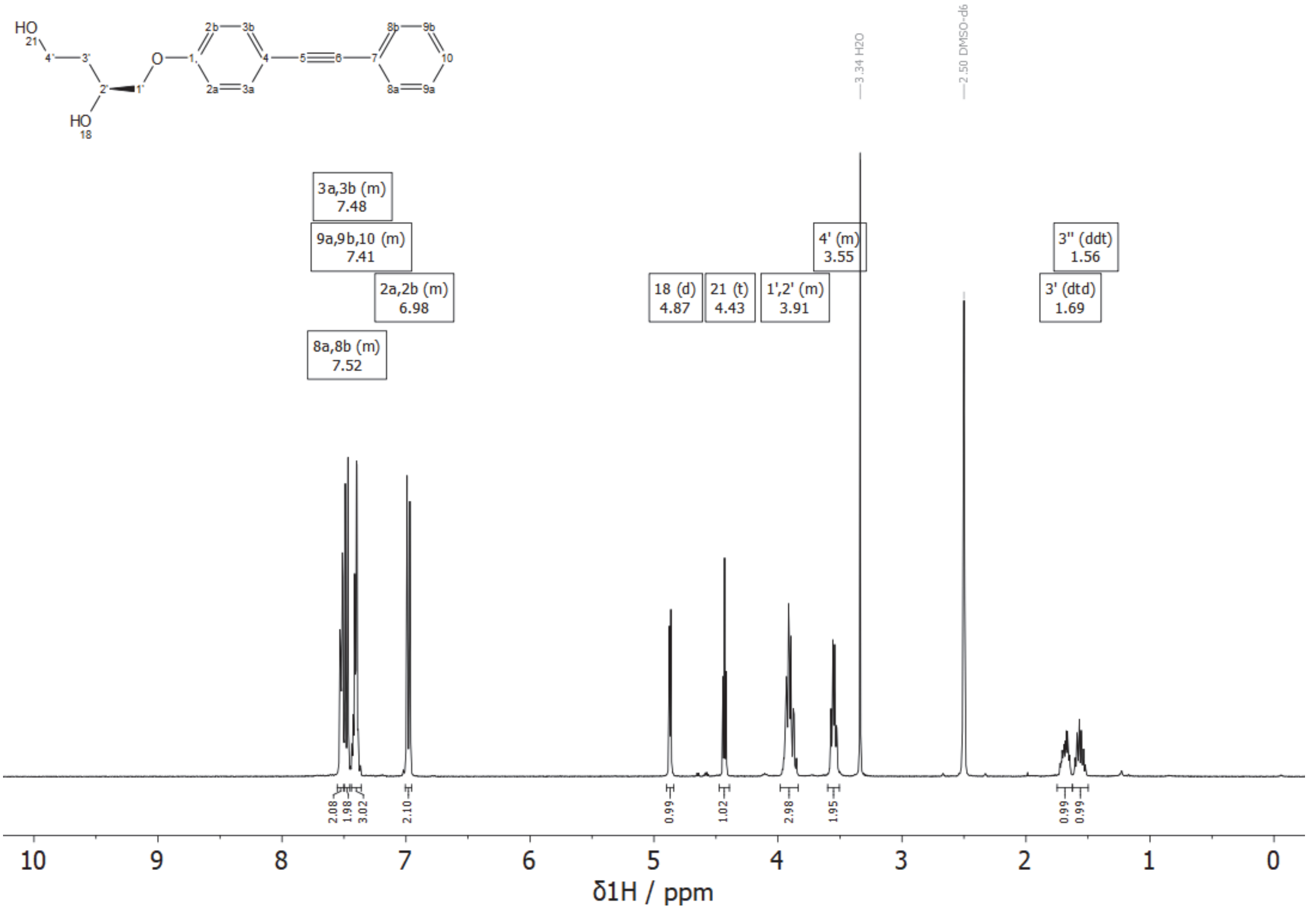

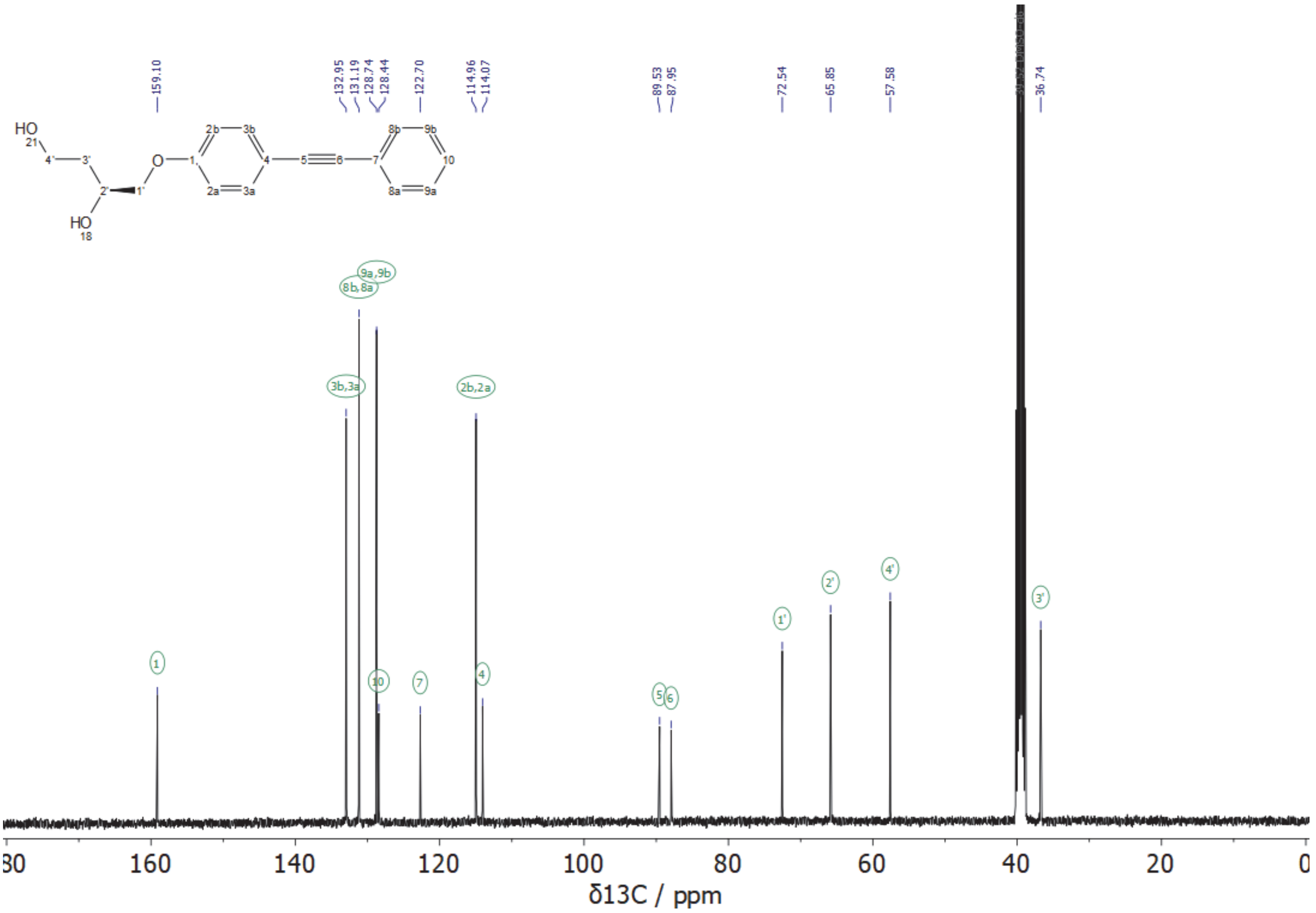

Compound **S5**

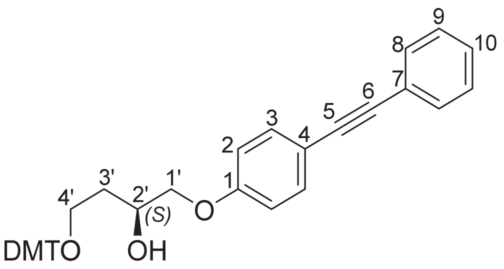

Compound **S4** (350 mg, 1.24 mmol, 1.00 eq.) was dissolved in anhydrous pyridine (19 mL). 4,4'-Dimethoxytrityl chloride (504 mg, 1.49 mmol, 1.20 eq.) was added in small portions over a time period of 25 min and the reaction mixture stirred at ambient temperature for 3 h. The solvent was removed under reduced pressure and the residue was purified by column chromatography (hexane/EtOAc 6:1–3:1 +1% Et_3N) to afford compound **S5** as a colorless foam (563 mg, 963 µmol, 78%).

^{1}H NMR (400 MHz, $CDCl_3$): *δ* (ppm) = 7.54 – 7.49 (m, 2H, 8-H), 7.47 – 7.42 (m, 4H, H-3, DMT-H), 7.38 – 7.27 (m, 9H, 9-H, 10-H, DMT-H), 7.24 – 7.19 (m, 1H, DMT-H), 6.88 – 6.79 (m, 6H, 2-H, DMT-H), 4.27 – 4.16 (m, 1H, 2'-H), 3.98 – 3.85 (m, 2H, 1'-H), 3.79 (s, 6H, DMT-H), 3.46 – 3.37 (m, 1H, 4'-H), 3.35 – 3.26 (m, 1H, 4'-H), 3.09 (d, *J* = 3.3 Hz, 1H, 2'-OH), 1.98 – 1.84 (m, 2H, 3'-H);

^{13}C{^{1}H} NMR (100 MHz, $CDCl_3$): *δ* (ppm) = 158.82 (C-1), 158.64 (DMT-C), 144.91 (DMT-C), 136.13 (DMT-C), 136.04 (DMT-C), 133.19 (8-C), 131.59 (3-C), 130.10, 128.15, 128.10, 128.06, 126.99, 123.67 (7-C), 115.79 (4-C), 114.72 (2-C), 113.32 (DMT-C), 89.42 (5-C), 88.30 (6-C), 86.80 (DMT-C), 71.89 (1'-C), 69.37 (2'-C), 61.38 (4'-C), 55.36 (DMT-C), 33.36 (3'-C);

HR-MS (ESI+): *m*/*z* calc. ($C_{39}H_{36}NaO_5$, $[M+Na]^+$): 607.24549, found: 607.24702.

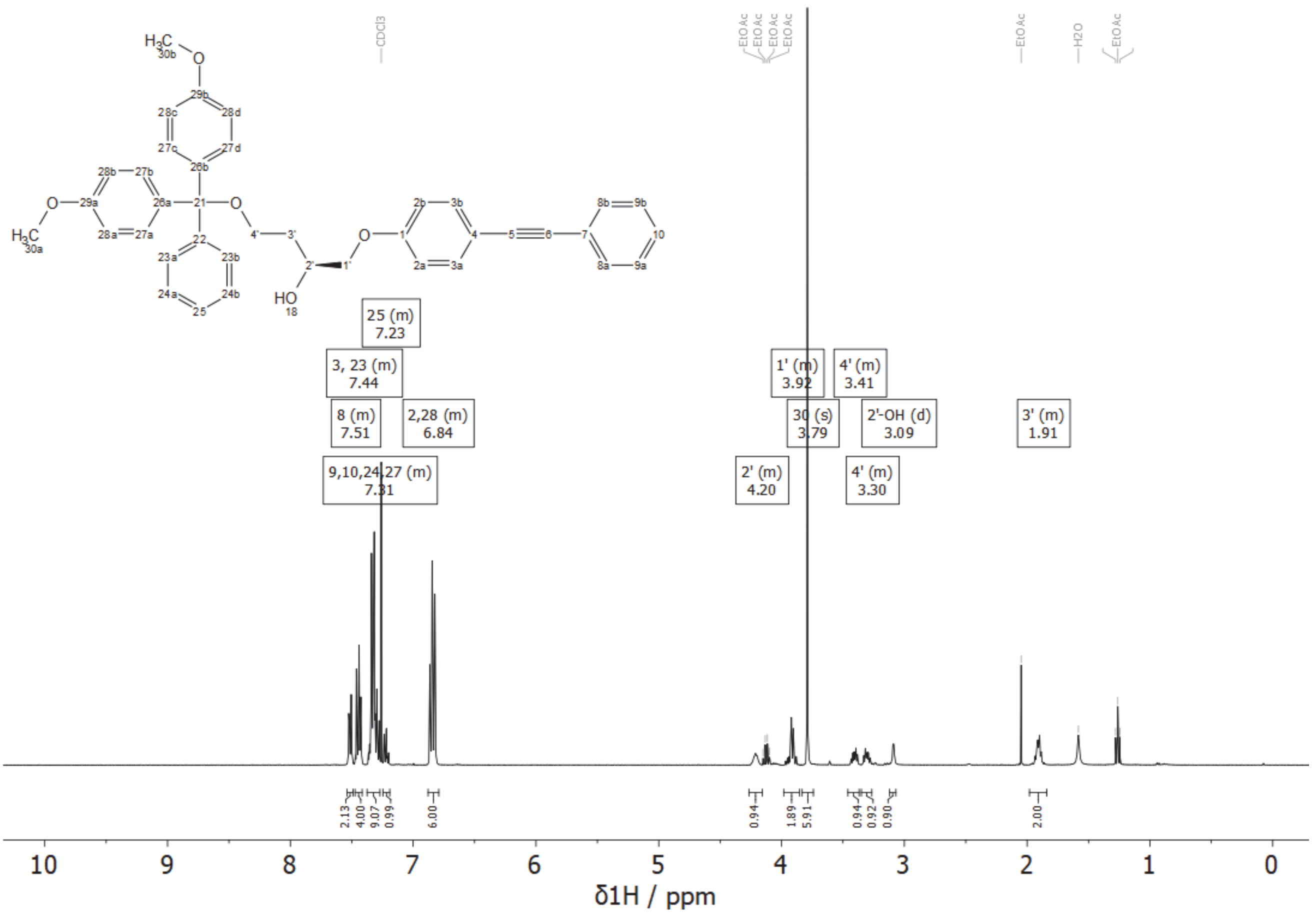

CDCl3
EtOAc
H2O
25 (m) 7.23
3, 23 (m) 7.44
8 (m) 7.51
2,28 (m) 6.84
9,10,24,27 (m) 7.31
1' (m) 3.92
4' (m) 3.41
30 (s) 3.79
2'-OH (d) 3.09
3' (m) 1.91
2' (m) 4.20
4' (m) 3.30
2.13
4.00
9.07
0.99
6.00
0.94
1.89
5.91
0.94
0.92
0.90
2.00
δ1H / ppm

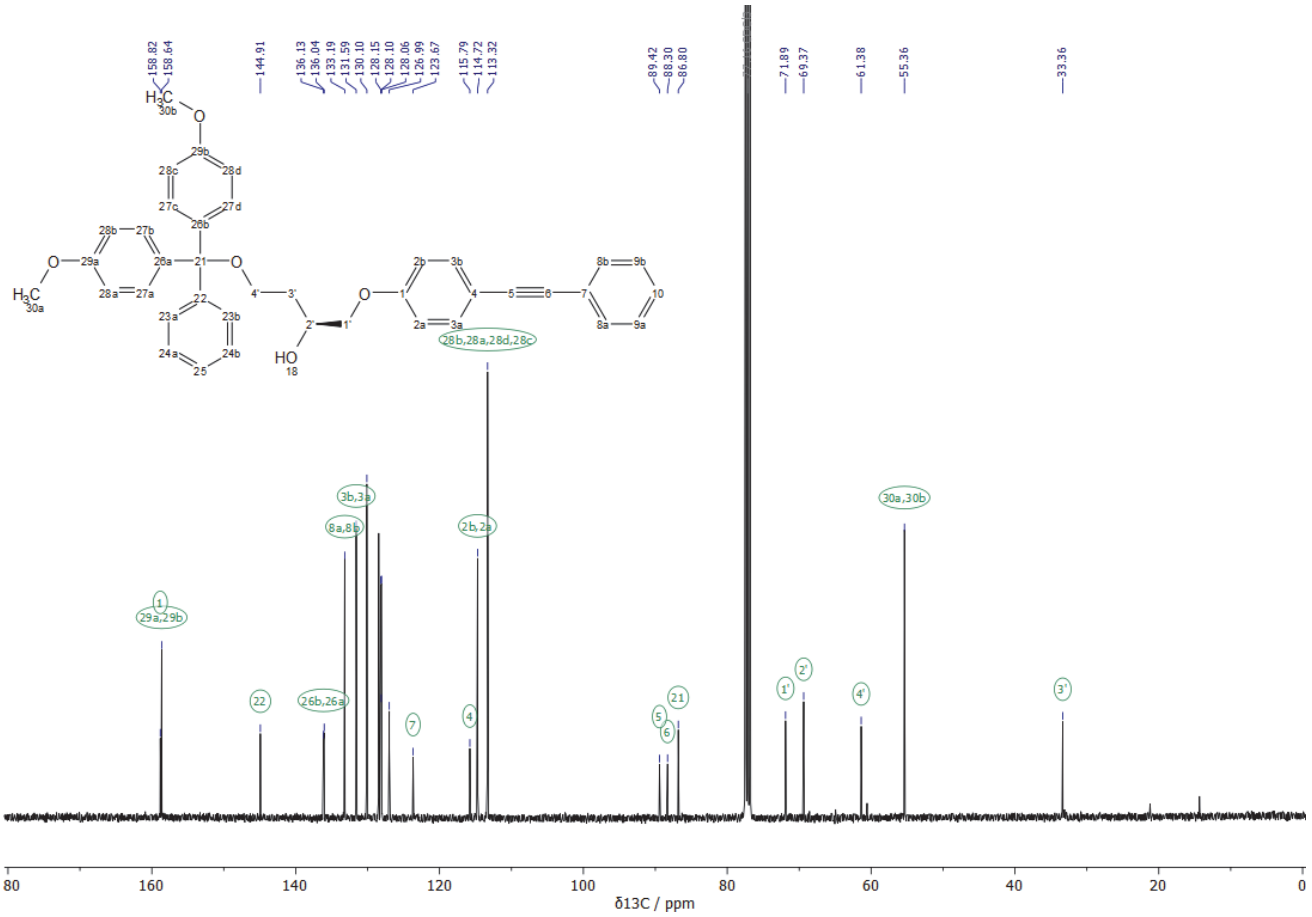

158.82
158.64
144.91
136.13
136.04
133.19
131.59
130.10
128.15
128.10
128.06
126.99
123.67
115.79
114.72
113.32
89.42
88.30
86.80
71.89
69.37
61.38
55.36
33.36
28b,28a,28d,28c
3b,3a
8a,8b
2b,2a
30a,30b
1
29a,29b
22
26b,26a
7
4
5
6
21
1'
2'
4'
3'
δ13C / ppm

Compound **S6** = BTHH-PA

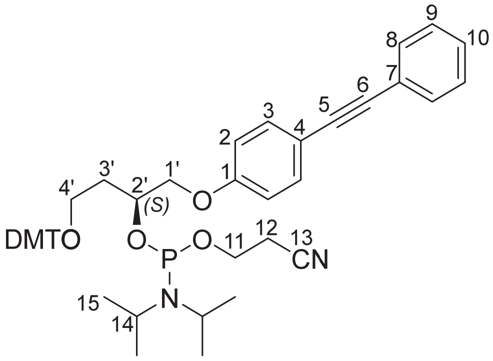

Compound **S5** (300 mg, 513 µmol, 1.00 eq.) was dissolved under nitrogen atmosphere with DIPEA (536 µL, 3.08 mmol, 6.00 eq.) in anhydrous DCM. After 10 min CEP-Cl (149 µL, 667 µmol, 1.30 eq.) was added. Additional CEP-Cl (11.5 µL, 51.3 µmol, 0.10 eq.) was added after 2 h stirring at room temperature. The reaction mixture was stirred additionally at ambient temperature for 1 h. The solvent was removed under reduced pressure and the residue was purified by column chromatography (hexane/EtOAc 6:1–5:1 + 1% Et_3N) to afford compound **S6** as a colorless foam (349 mg, 445 µmol, 87%).

^{1}H NMR (400 MHz, $CDCl_3$): *δ* (ppm) = δ 7.55 – 7.47 (m, 4H, 8-H), 7.48 – 7.38 (m, 8H, 3-H, DMT-H), 7.40 – 7.21 (m, 17H, 9-H, 10-H, DMT-H), 7.26 – 7.15 (m, 2H, DMT-H), 6.87 – 6.75 (m, 12H, 2-H, DMT-H), 4.41 – 4.30 (m, 2H, 2'-H), 4.06 – 3.95 (m, 4H, 1'-H), 3.88 – 3.67 (m, 14H, 11-H, DMT-H), 3.68 – 3.46 (m, 6H, 11-H, 14-H), 3.32 – 3.18 (m, 4H, 4'-H), 2.61 – 2.36 (m, 4H, 12-H), 2.11 – 1.94 (m, 4H, 3'-H), 1.20 – 1.00 (m, 24H, 15-H).;

^{13}C{^{1}H} NMR (100 MHz, $CDCl_3$): *δ* (ppm) = 158.98 (C-1), 158.92 (C-1), 158.51 (DMT-C), 145.23 (DMT-C), 145.22 (DMT-C), 136.53 (DMT-C), 136.50 (DMT-C), 136.45 (DMT-C), 136.40 (DMT-C), 133.19 (3-C), 133.14 (3-C), 131.58 (8-C), 130.18 (DMT-C), 130.12 (DMT-C), 130.11 (DMT-C), 128.45, 128.33, 128.23, 128.08, 127.92, 126.84 (DMT-C), 126.83 (DMT-C), 123.71 (7-C), 117.88 (13-C), 117.78 (13-C), 115.56 (4-C), 115.51 (4-C), 114.77 (2-C), 114.75 (2-C), 113.18 (DMT-C), 113.16 (DMT-C), 89.51 (6-C), 89.49 (6-C), 88.24 (5-C), 88.22 (5-C), 86.26 (DMT-C), 86.23 (DMT-C), 71.00 (1'-C), 70.98 (1'-C), 70.77 (2'-C), 70.59(2'-C), 70.52 (2'-C), 70.35 (2'-C), 59.90 (4'-C), 59.82 (4'-C), 58.51(11-C), 58.48 (11-C), 58.33 (11-C), 58.29 (11-C), 55.36 (DMT-C), 55.34 (DMT-C), 43.32 (14-C), 43.19 (14-C), 34.00 (3'-C), 33.96 (3'-C), 33.93 (3'-C), 33.89 (3'-C), 24.86 (15-C), 24.78 (15-C), 24.71 (15-C), 24.67 (15-C), 24.62 (15-C), 24.59 (15-C), 24.54 (15-C), 20.47 (12-C), 20.40 (12-C), 20.37 (12-C), 20.30 (12-C);

^{31}P{^{1}H} NMR (162 MHz, $CDCl_3$): *δ* (ppm) = 148.94, 148.61;

HR-MS (ESI+): *m/z* calc. ($C_{48}H_{53}N_2NaO_6P$, $[M+Na]^+$): 807.3533, found: 807.3523.

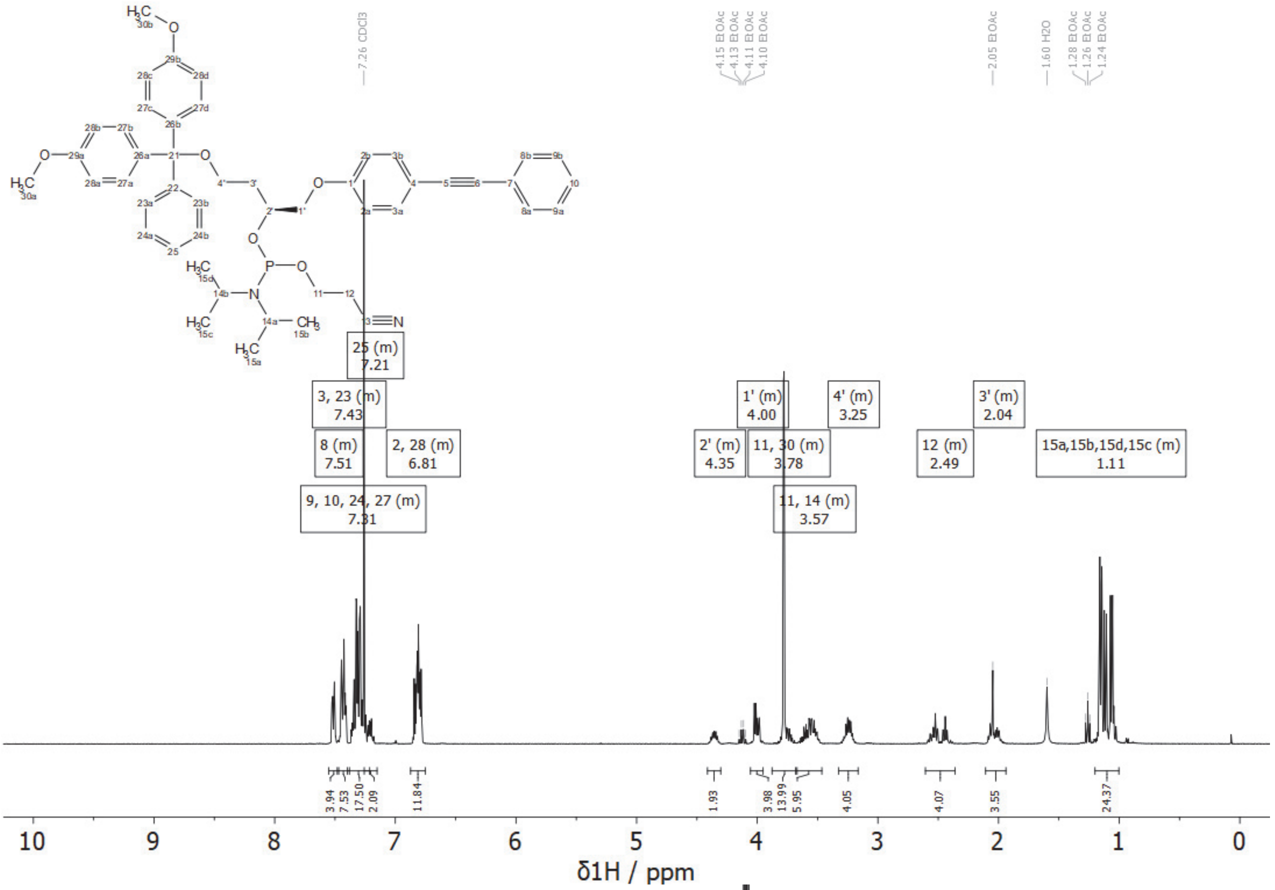
7.26 CDCl3
4.15 EtOAc
4.13 EtOAc
4.11 EtOAc
4.10 EtOAc
2.05 EtOAc
1.60 H2O
1.28 EtOAc
1.26 EtOAc
1.24 EtOAc
25 (m) 7.21
3, 23 (m) 7.43
8 (m) 7.51
2, 28 (m) 6.81
9, 10, 24, 27 (m) 7.31
1' (m) 4.00
4' (m) 3.25
2' (m) 4.35
11, 30 (m) 3.78
11, 14 (m) 3.57
3' (m) 2.04
12 (m) 2.49
15a,15b,15d,15c (m) 1.11
3.94
7.53
17.50
2.09
11.84
1.93
3.98
13.99
5.95
4.05
4.07
3.55
24.37
δ1H / ppm

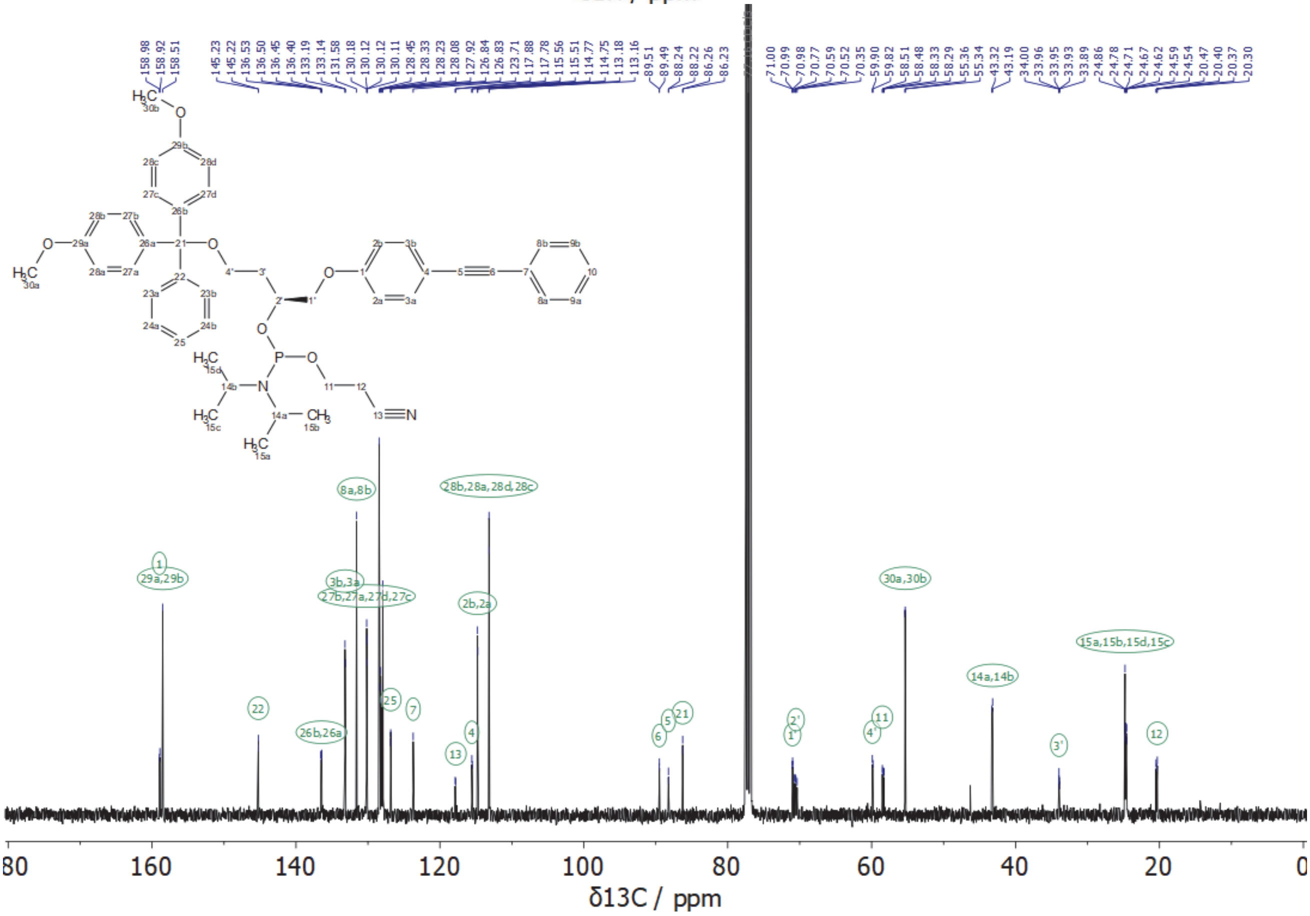
158.98
158.92
158.51
145.23
145.22
136.53
136.50
136.45
136.40
133.19
133.14
131.58
130.18
130.12
130.11
128.45
128.33
128.23
128.08
127.92
126.84
126.83
123.71
117.88
117.78
115.56
115.51
114.77
114.75
113.18
113.16
89.51
89.49
88.24
88.22
86.26
86.23
71.00
70.99
70.98
70.77
70.59
70.52
70.35
59.90
59.82
58.51
58.48
58.33
58.29
55.36
55.34
43.32
43.19
34.00
33.96
33.95
33.93
33.89
24.86
24.78
24.71
24.67
24.62
24.59
24.54
20.47
20.40
20.37
20.30
8a,8b
28b,28a,28d,28c
1
29a,29b
3b,3a
27b,27a,27d,27c
2b,2a
30a,30b
15a,15b,15d,15c
14a,14b
22
26b,26a
25
7
4
13
6
5
21
2'
1'
4'
11
3'
12
δ13C / ppm

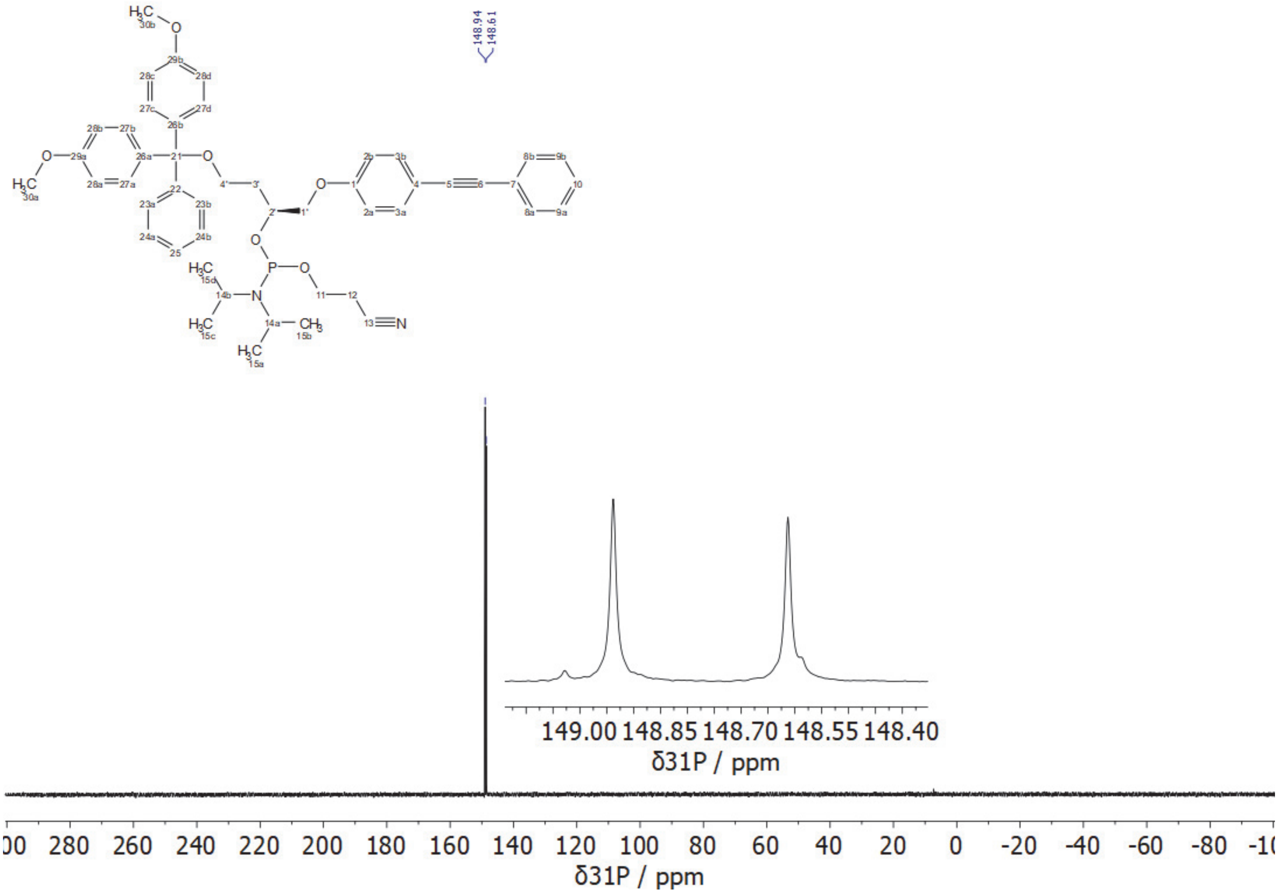

Compound **S7**

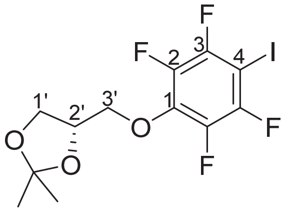

(*S*)-(+)-2,2-Dimethyl-1,3-dioxolane-4-methanol (**4**, also known as (*S*)-(+)-1,2-isopropylidenglycerin or D-(+)-Solketal) (1.87 mL, 2.00 g, 15.1 mmol, 1.00 eq.) and sodium hydride (60% oil dispersion, 605 mg, 15.1 mmol, 1.00 eq.) was dissolved in anhydrous THF (75 mL) and stirred under ambient temperature for 30 min. Iodopentafluorobenzene (2.02 mL, 4.45 g, 15.1 mmol, 1.00 eq.) was added dropwise to the mixture. After 3 h water (50 mL) was added. The mixture was extracted with Et_2O (3x100 mL). The combined organic phases were washed with brine (50 mL) and dried over Na_2SO_4, evaporated and the residue was purified by column chromatography (DCM/hexane 1:1) to afford compound **S7** (3.66 g, 9.01 mmol, 60%) as a colorless oil.

^{1}H NMR (400 MHz, $CDCl_3$): *δ* (ppm) = 4.44 (p, *J* = 5.6 Hz, 1H, 2'-H), 4.32 – 4.27 (m, 1H, 3'-H), 4.22 – 4.17 (m, 1H, 3'-H), 4.17 – 4.12 (m, 1H, 1'-H), 3.97 – 3.92 (m, 1H, 1'-H), 1.42 (q, *J* = 0.7 Hz, 3H, CH_3), 1.38 (q, *J* = 0.7 Hz, 3H, CH_3);

^{13}C{^{1}H} NMR (100 MHz, $CDCl_3$): *δ* (ppm) = 148.84 – 148.42 (m), 146.48 – 145.80 (m), 142.32 – 141.39 (m), 139.82 – 139.05 (m), 138.46 – 137.80 (m), 110.10 (*C*(CH_3)$_2$), 74.97 (t, *J* = 3.2 Hz, 3'-C), 74.13 (2'-C), 66.28 (1'-C), 64.53 (t, J = 28.2 Hz, 4-C), 26.78 (CH_3), 25.39 (CH_3);

^{19}F{^{1}H} NMR (376 MHz, $CDCl_3$): *δ* (ppm) = -120.74 – -121.37 (m, 2F, 3-F), -153.82 – -154.27 (m, 2F, 2-F);

HR-MS (ESI+): *m/z* calc. ($C_{12}H_{11}F_4IO_3Na$, $[M+Na]^+$): 428.95813, found: 428.95787.

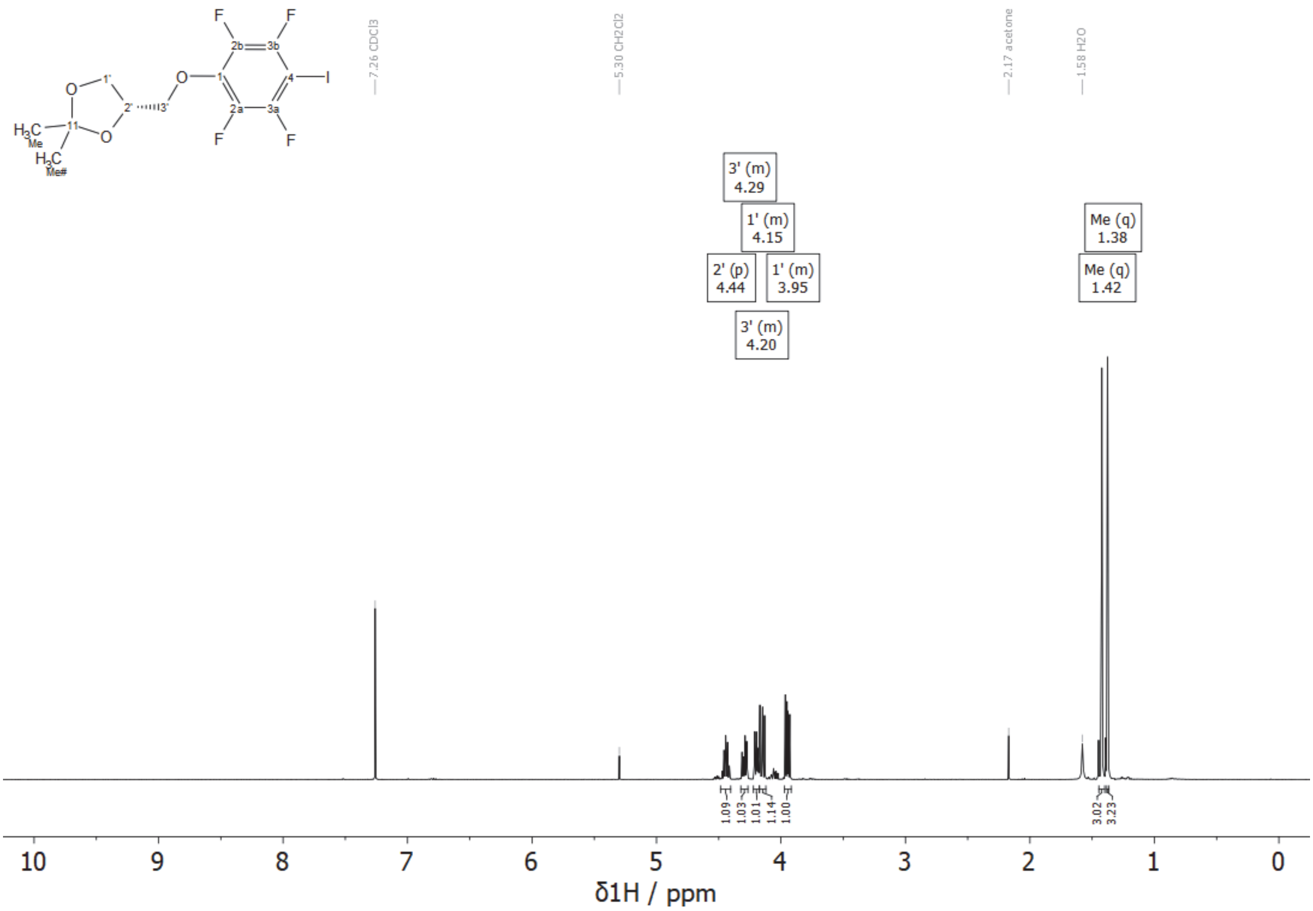

7.26 CDCl3
5.30 CH2Cl2
2.17 acetone
1.58 H2O
3' (m) 4.29
1' (m) 4.15
2' (p) 4.44
1' (m) 3.95
3' (m) 4.20
Me (q) 1.38
Me (q) 1.42
1.09
1.03
1.01
1.14
1.00
3.02
3.23
10
9
8
7
6
5
4
3
2
1
0
δ1H / ppm

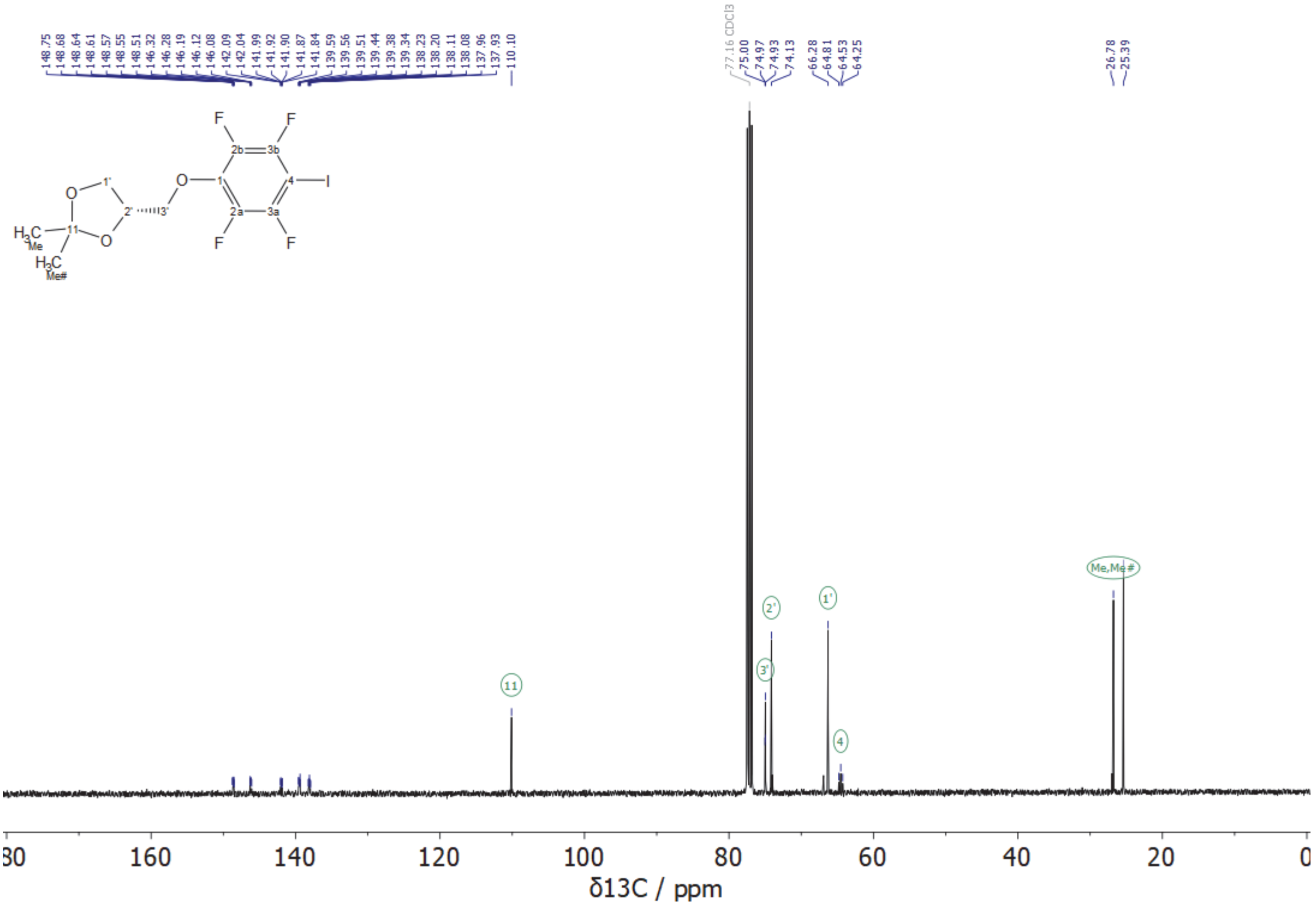
148.75
148.68
148.64
148.61
148.57
148.55
148.51
146.32
146.28
146.19
146.12
146.08
142.09
142.04
141.99
141.92
141.90
141.87
141.84
139.59
139.56
139.51
139.44
139.38
139.34
138.23
138.20
138.11
138.08
137.96
137.93
110.10
77.16 CDCl3
75.00
74.97
74.93
74.13
66.28
64.81
64.53
64.25
26.78
25.39
11
2'
3'
1'
4
Me,Me#
δ13C / ppm

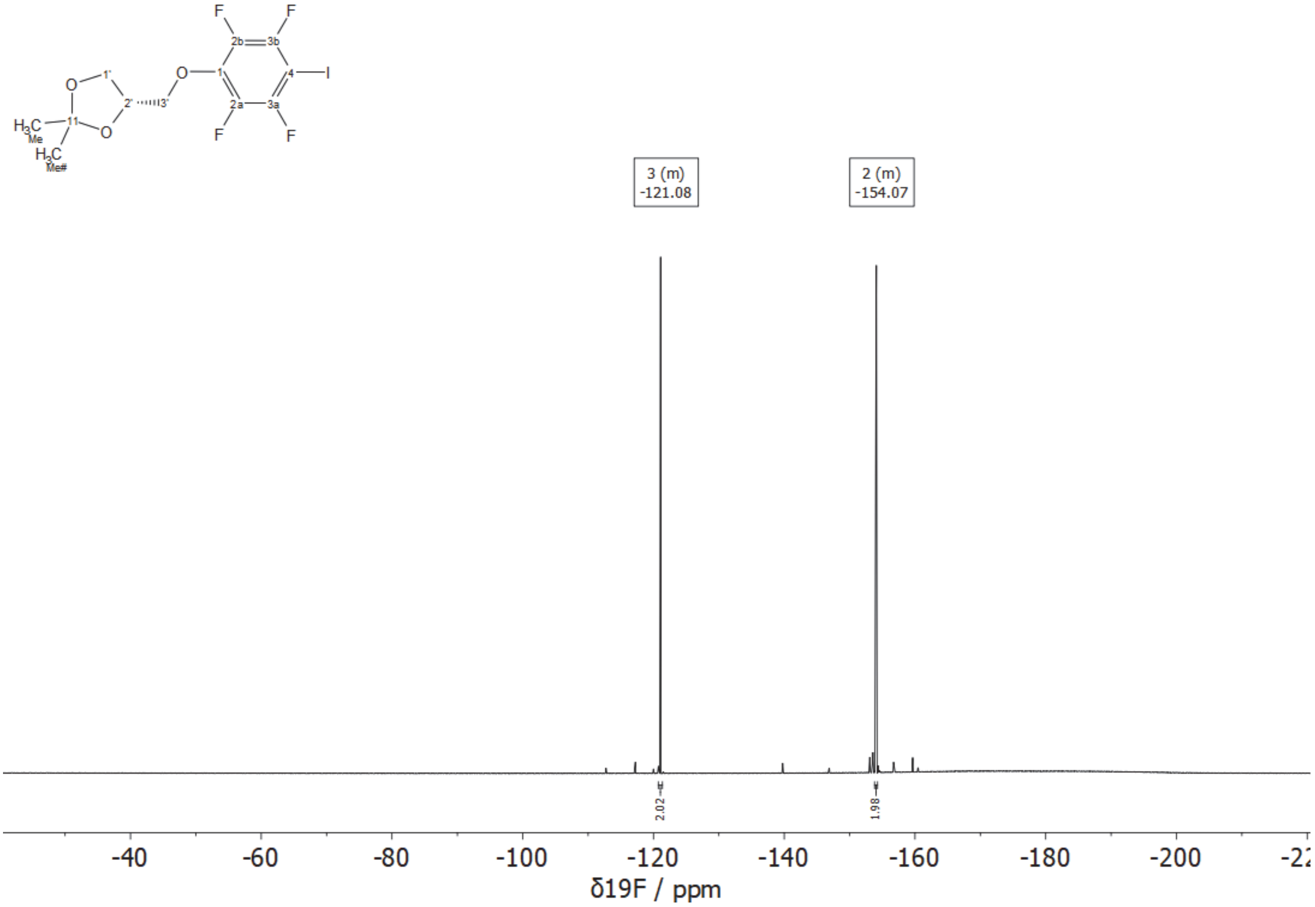
3 (m)
-121.08
2 (m)
-154.07
2.02
1.98
δ19F / ppm

Compound **S8**

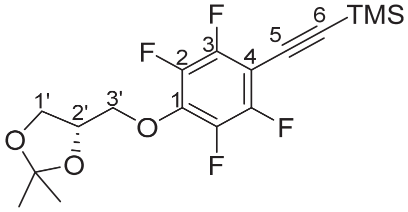

Compound **S8** was synthesized according to Tschierske et al.[5] Under nitrogen atmosphere, CuI (79.7 mg, 419 µmol, 10 mol%) and $Pd(PPh_3)_4$ (242 mg, 209 µmol, 5.0 mol%) was dissolved in argon-purged Et_3N (20 mL). A solution of compound **S7** (1.70 g, 4.19 mmol, 1.00 eq.) in argon-purged Et_3N (7 mL) was added and stirred at ambient temperature for 10 min. Then, trimethylsilylacetylene (1.16 mL, 822 mg, 8.37 mmol, 2.00 eq) was added and stirring was continued for 20 h at 80 °C. The precipitate mixture was filtered off and washed with Et_2O. The filtrate was washed with a saturated solution of ammonium chloride (2x50 mL). The aqueous phase was extracted with Et_2O (2x50 mL). The combined organic phases were washed with brine (50mL), dried over Na_2SO_4, evaporated and the residue was purified by column chromatography (DCM/hexane 1:1) to afford compound **S8** as a yellow oil (1.38 g, 3.67 mmol, 88%).

^{1}H NMR (400 MHz, $CDCl_3$): δ (ppm) = 4.48 – 4.39 (m, 1H, 2'-H), 4.33 – 4.27 (m, 1H, 3'-H), 4.24 – 4.18 (m, 1H, 3'-H), 4.17 – 4.11 (m, 1H, 2'-H), 3.97 – 3.91 (m, 1H, 2'-H), 1.42 (q, J = 0.8 Hz, 3H, CH_3), 1.37 (q, J = 0.7 Hz, 3H, CH_3), 0.27 (s, 9H, TMS);

^{13}C{^{1}H} NMR (100 MHz, $CDCl_3$): δ (ppm) = 149.24 – 148.35 (m), 146.92 – 145.96 (m), 142.82 – 141.39 (m), 140.15 – 139.37 (m), 138.50 – 137.67 (m, 1-C), 110.11 ($C(CH_3)_2$), 107.93 (t, J = 3.6 Hz, C-6), 98.65 (C-4), 88.49 (t, J = 3.7 Hz, C-5), 74.93 (t, J = 3.4 Hz, C-3'), 74.17 (C-2'), 66.24 (C-1'), 26.76 (CH_3), 25.40 (CH_3), -0.24 (TMS).;

^{19}F{^{1}H} NMR (376 MHz, $CDCl_3$): δ (ppm) = -134.68 – -140.68 (m, 2F, 2-F), -154.78 – -159.33 (m, 2F, 3-F);

HR-MS (ESI+): m/z calc. ($C_{17}H_{20}F_4O_3SiNa$, $[M+Na]^+$): 399.10100, found: 399.10083.

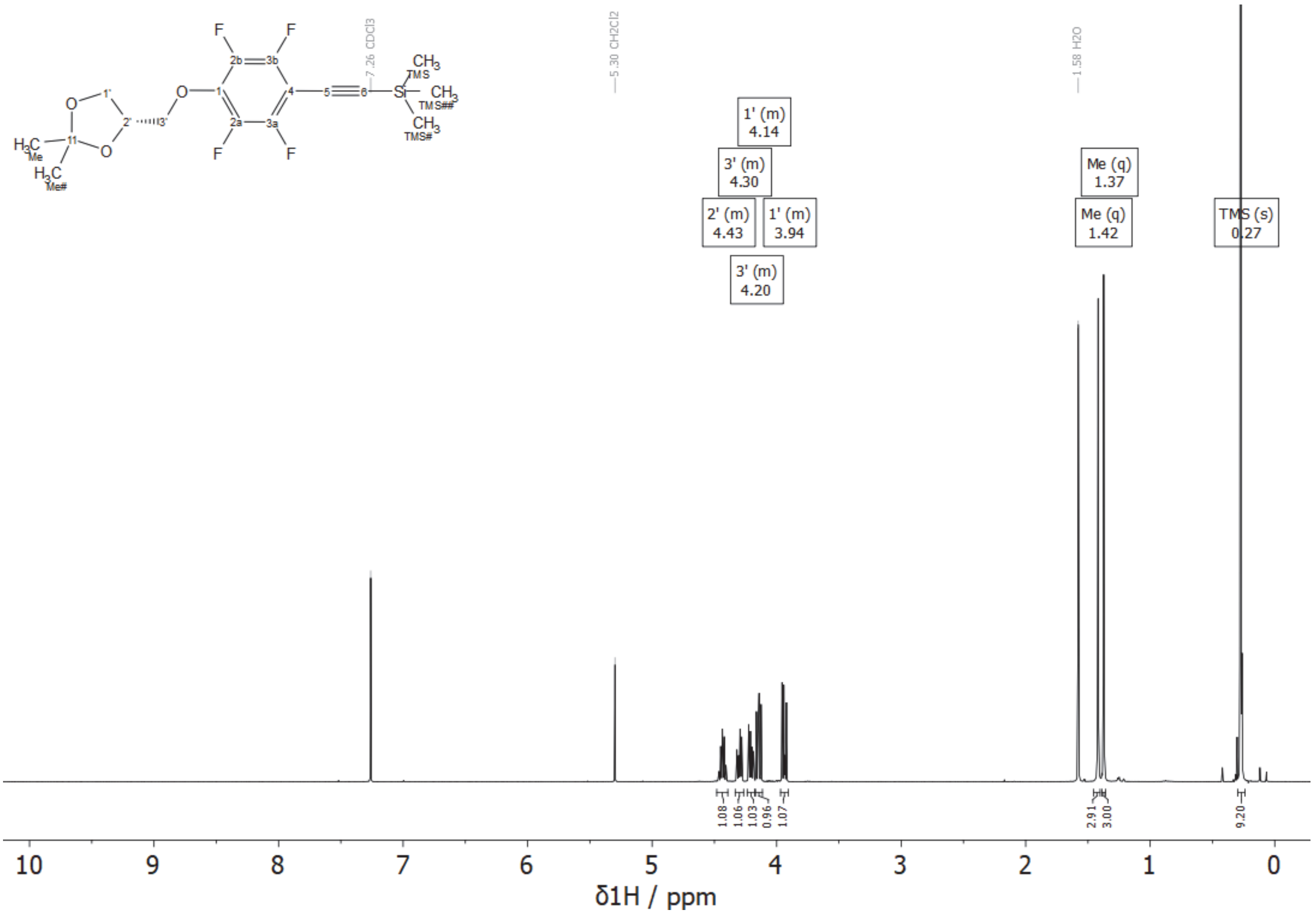

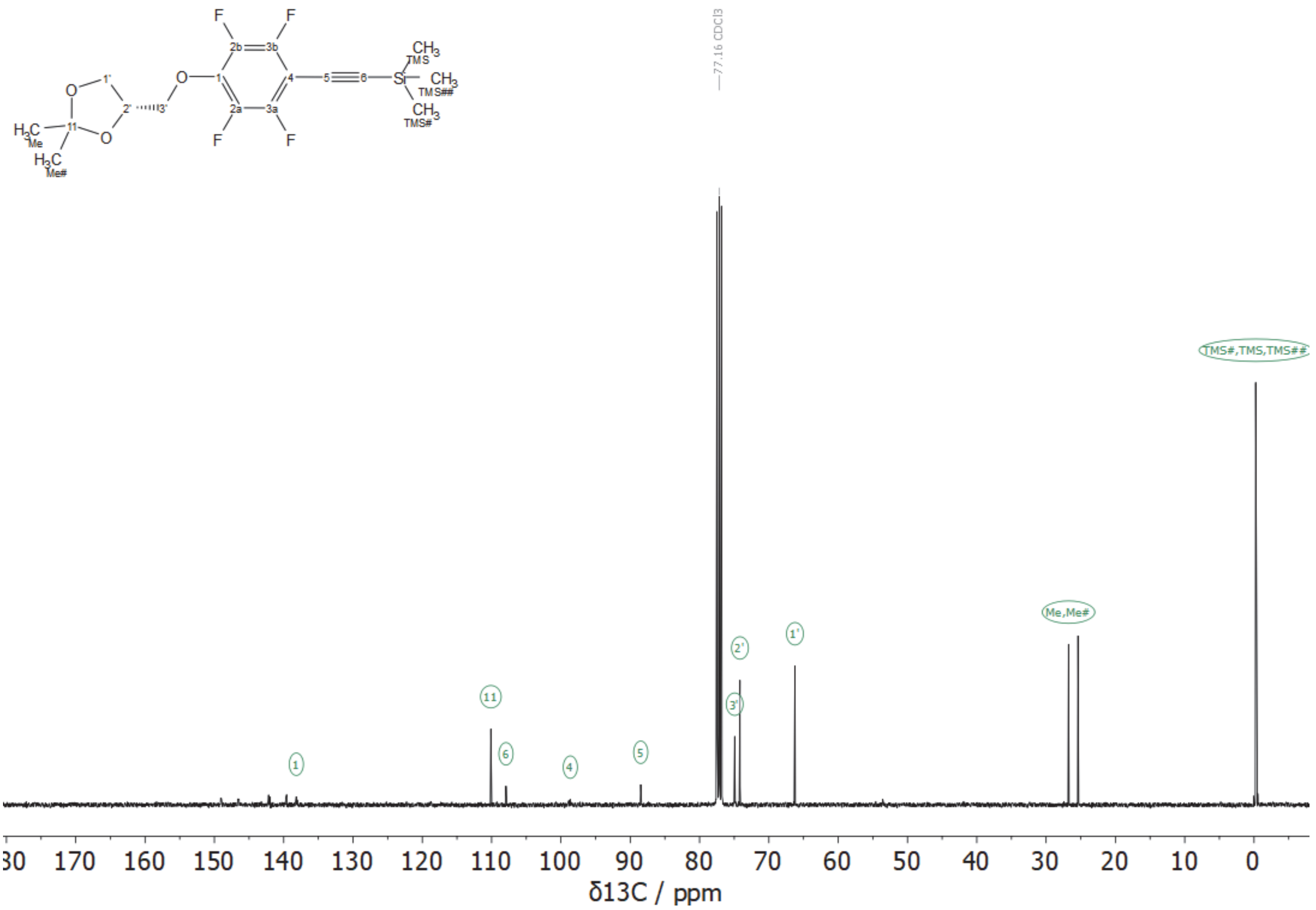
77.16 CDCl3
TMS#,TMS,TMS##
Me,Me#
δ13C / ppm

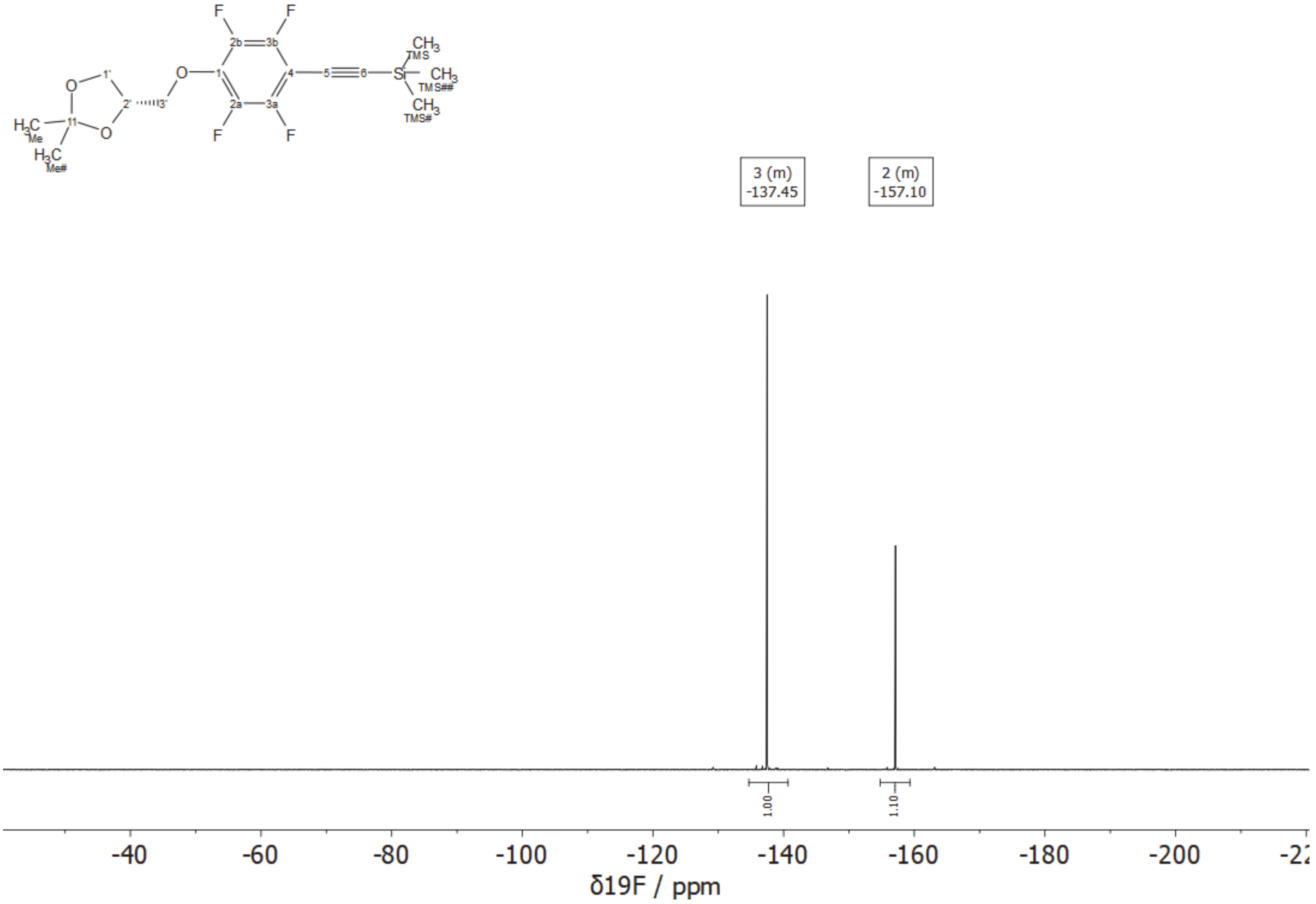
3 (m)
-137.45
2 (m)
-157.10
1.00
1.10
δ19F / ppm

Compound **S9**

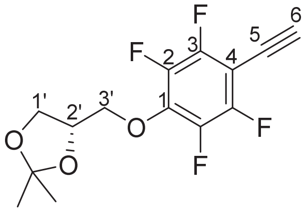

Compound **S8** (1,43 mg, 3.52 µmol, 1.00 eq.) was dissolved in THF/MeOH (1:1, 10 mL). K_2CO_3 (147 mg, 1.06 mmol, 1.00 eq.) was added and the mixture was stirred for 2 h at room temperature. The suspension was diluted with water (30 mL), extracted with Et_2O (3x50 mL) and washed with brine (2x30 mL). The organic phase was dried over Na_2SO_4, evaporated and the residue was purified by column chromatography (DCM/hexane 1:1) to afford compound **S9** as a light brown oil (312 mg, 1.03 mmol, 96%).

^{1}H NMR (400 MHz, $CDCl_3$): δ (ppm) = 4.48 – 4.40 (m, 1H, H-2'), 4.36 – 4.28 (m, 1H, H-3'), 4.27 – 4.19 (m, 1H, H-3'), 4.18 – 4.12 (m, 1H, H-3'), 3.99 – 3.91 (m, 1H, H-3'), 3.56 (q, J = 0.8 Hz, 1H, H-6), 1.42 (q, J = 0.8 Hz, 3H, CH_3), 1.38 (q, J = 0.7 Hz, 3H, CH_3).;

^{13}C{^{1}H} NMR (100 MHz, $CDCl_3$): δ (ppm) = 149.96 – 148.75 (m), 147.36 – 146.35 (m), 142.65 – 141.50 (m), 140.05 – 139.24 (m), 139.04 – 138.20 (m), 110.13 ($C(CH_3)_2$), 97.33 (t, J = 18.1 Hz, C-4), 88.85 (t, J = 3.8 Hz, C-5), 74.94 (t, J = 3.4 Hz, C-3'), 74.15 (C-2'), 68.69 (t, J = 4.0 Hz, C-6), 66.20, 26.75, 25.38;

^{19}F{^{1}H} NMR (376 MHz, $CDCl_3$): δ (ppm) = -136.42 – -138.69 (m, 2F, 2-F), -155.34 – -158.80 (m, 2F, 3-F);

HR-MS (ESI+): m/z calc. ($C_{14}H_{12}F_4O_3Na$, $[M+Na]^+$): 327.06148, found: 327.06102.

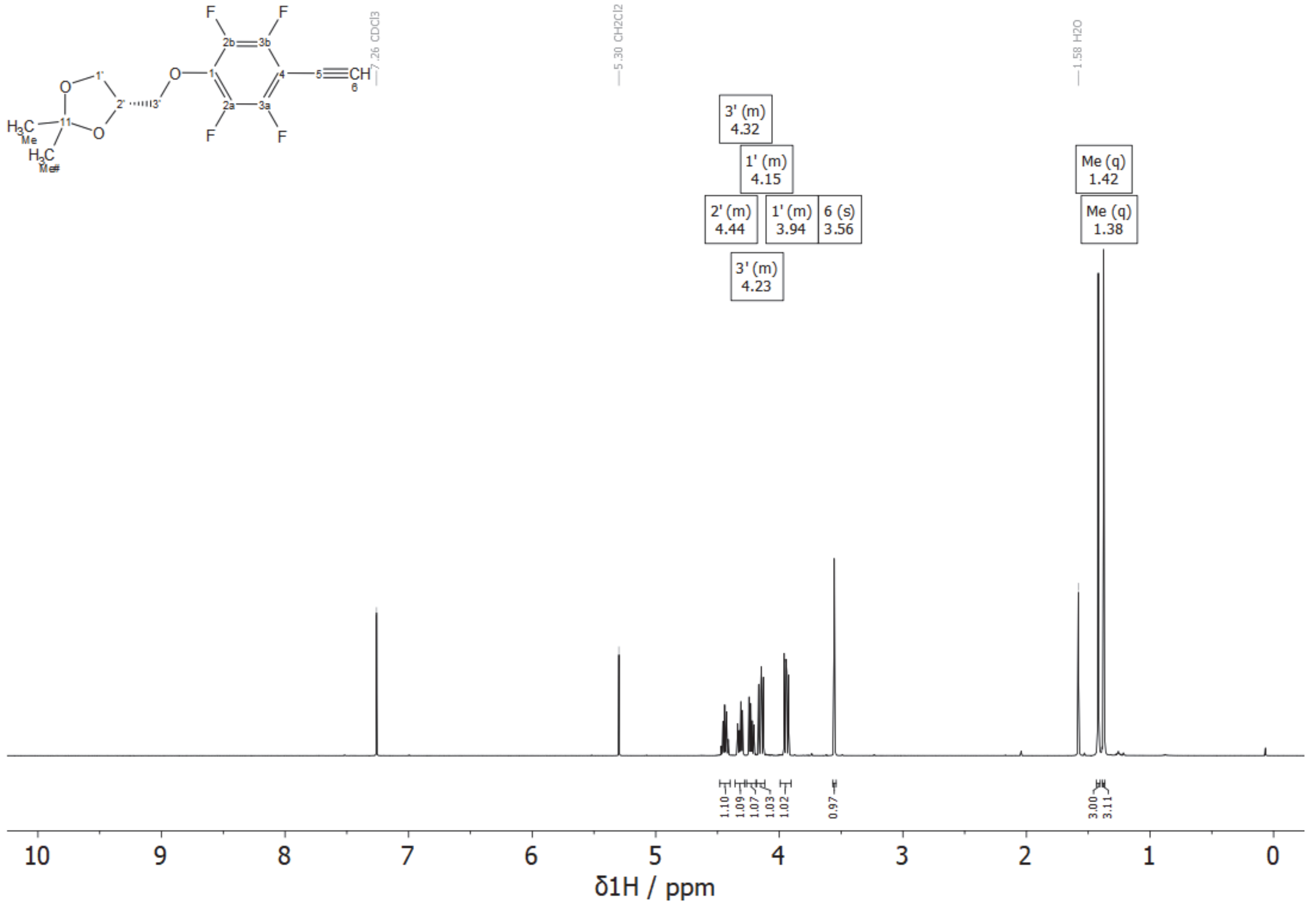

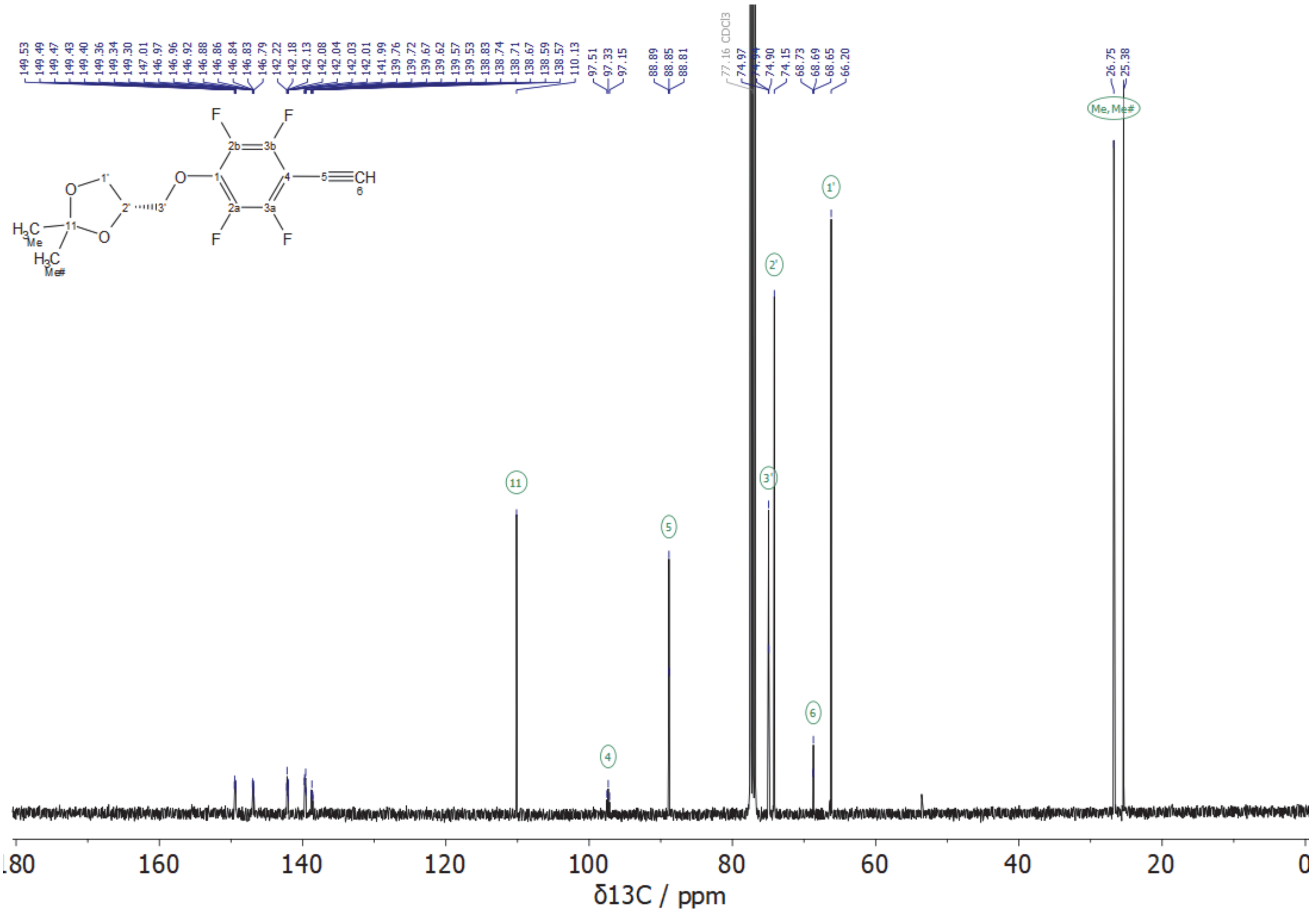

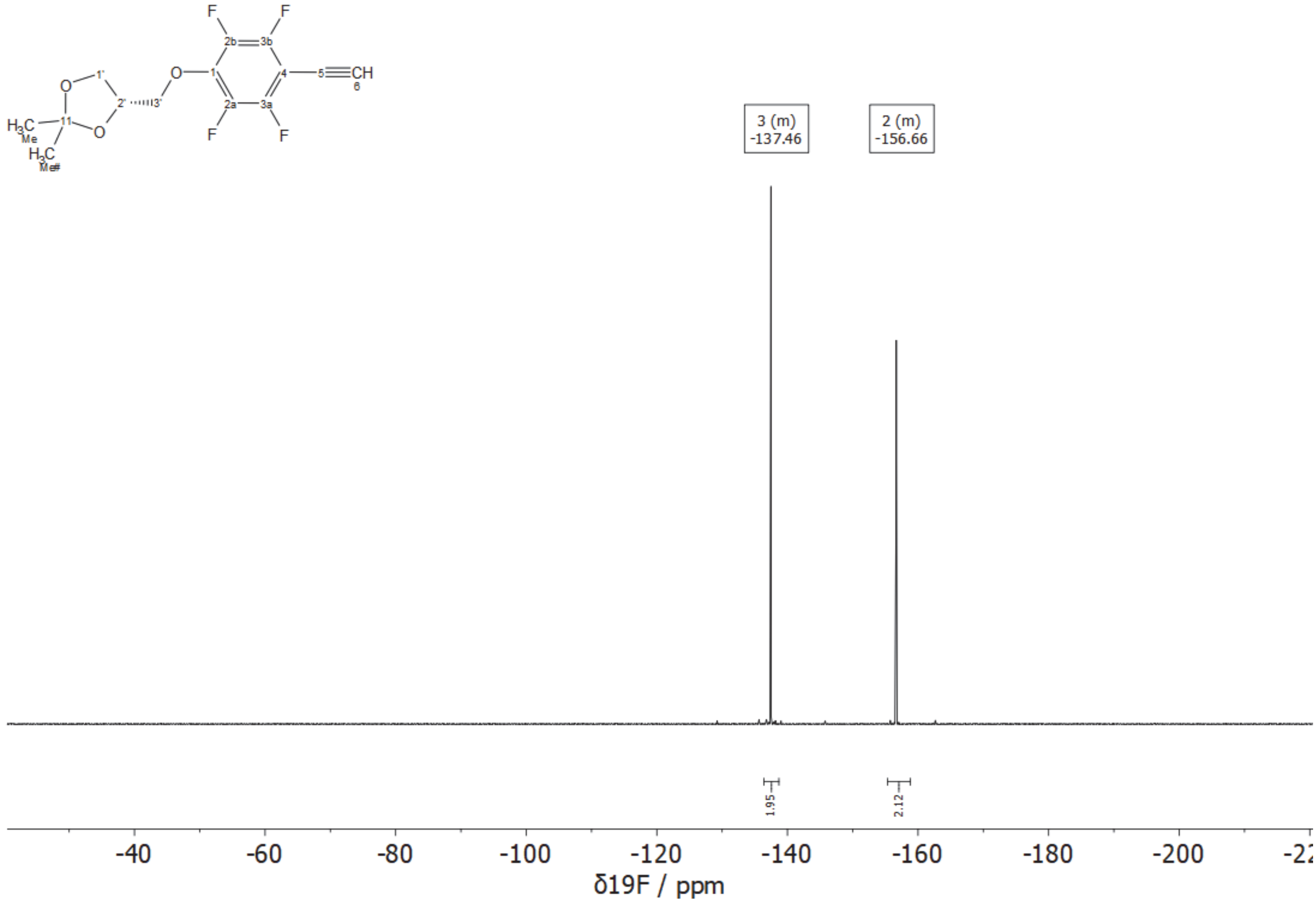

Compound **S10**

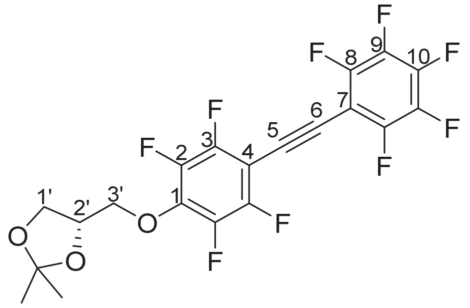

Under nitrogen atmosphere, CuI (17.2 mg, 90.3 µmol, 10 mol%), $Pd(PPh_3)_4$ (52.0 mg, 45.0 µmol, 5.0 mol%) and iodopentafluorobenzene (180 µL, 397 mg, 1.35 mmol, 1.50 eq.) were dissolved in argon-purged Et_3N (8.7 mL). A solution of compound **S9** (274 mg, 900 µmol, 1.00 eq.) in argon-purged Et_3N (2.4 mL) was added and stirring was continued for 20 h at 60°C. The precipitate mixture was filtered off and washed with Et_2O. The filtrate was washed with a saturated solution of ammonium chloride (2x30 mL). The aqueous phase was extracted with Et_2O (2 x 30 mL). The combined organic phases were washed with brine (30mL), dried over Na_2SO_4, evaporated and the residue was purified by column chromatography (DCM/hexane 1:1) to afford compound **S10** as a pale yellow solid (868 g, 1.84 mmol, 81%).

^{1}H NMR (400 MHz, $CDCl_3$): *δ* (ppm) = 4.53 – 4.41 (m, 1H, 2'-H), 4.40 – 4.32 (m, 1H, 3'-H), 4.32 – 4.24 (m, 1H, 3'-H), 4.21 – 4.12 (m, 1H, 1'-H), 4.01 – 3.90 (m, 1H, 1'-H), 1.43 (q, *J* = 0.7 Hz, 3H, CH_3), 1.38 (q, *J* = 0.7 Hz, 3H, CH_3);

^{13}C{^{1}H} NMR (100 MHz, $CDCl_3$): *δ* (ppm) = 148.99 – 148.16 (m), 146.70 – 145.67 (m), 142.53 – 141.72 (m), 139.95 – 138.69 (m), 136.77 – 136.14 (m), 110.19 (*C*(CH_3)$_2$), 99.29, 96.99, 87.74 – 85.67 (m), 85.00 – 81.68 (m), 74.97 (t, *J* = 3.5 Hz, 3'-C), 74.17 (2'-C), 66.17 (1'-C), 26.75 (CH_3), 25.38 (CH_3);

^{19}F{^{1}H} NMR (376 MHz, $CDCl_3$): *δ* (ppm) = -133.73 – -135.61 (m, 2F), -135.92 – -136.54 (m, 2F), -150.24 (tt, *J* = 20.7, 2.4 Hz, 1F), -156.04 – -156.61 (m, 2F), -160.85 – -161.32 (m, 2F);

HR-MS (ESI+): *m/z* calc. ($C_{20}H_{11}F_9O_3Na$, $[M+Na]^+$): 493.04567 , found:. 493.04563

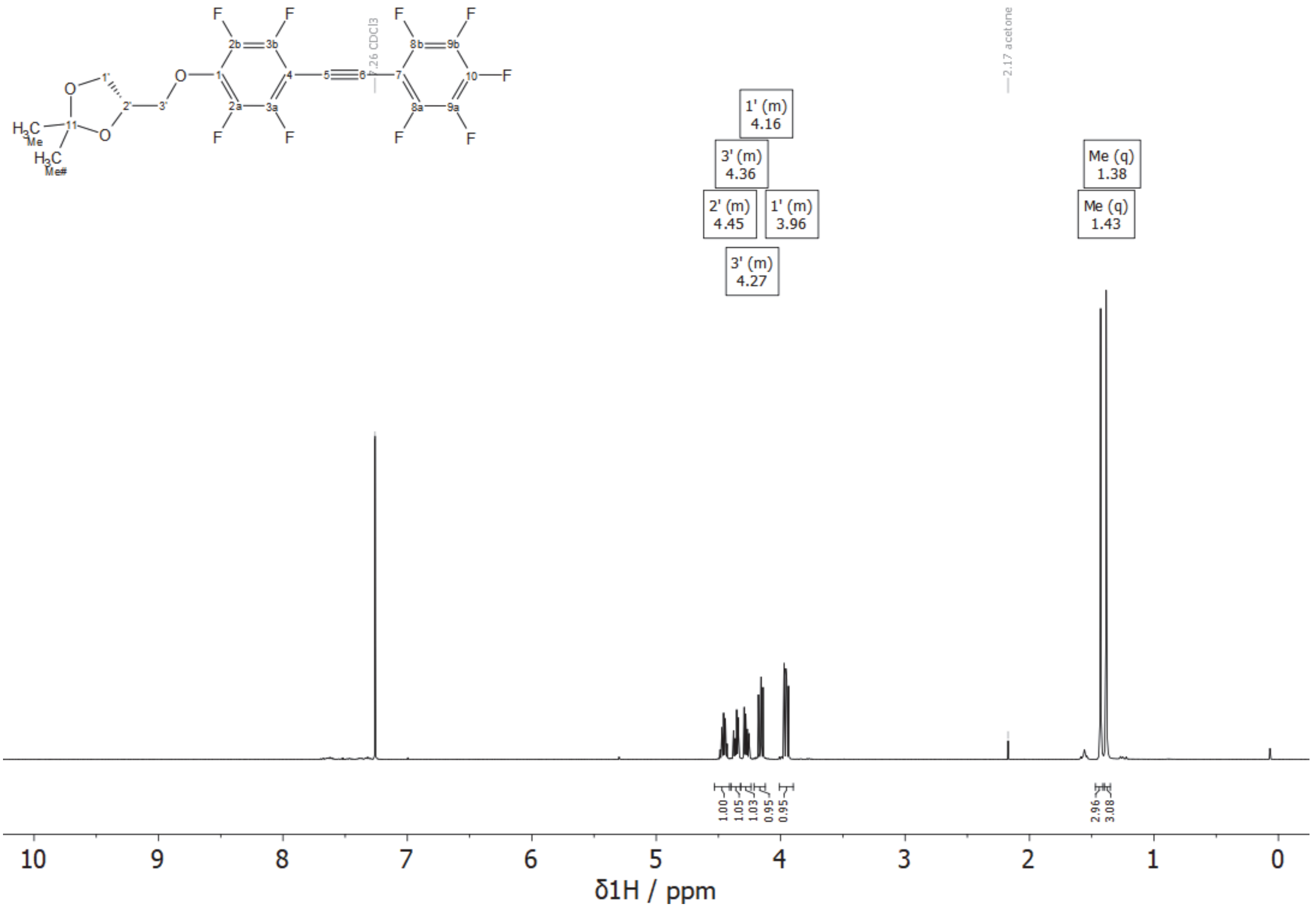

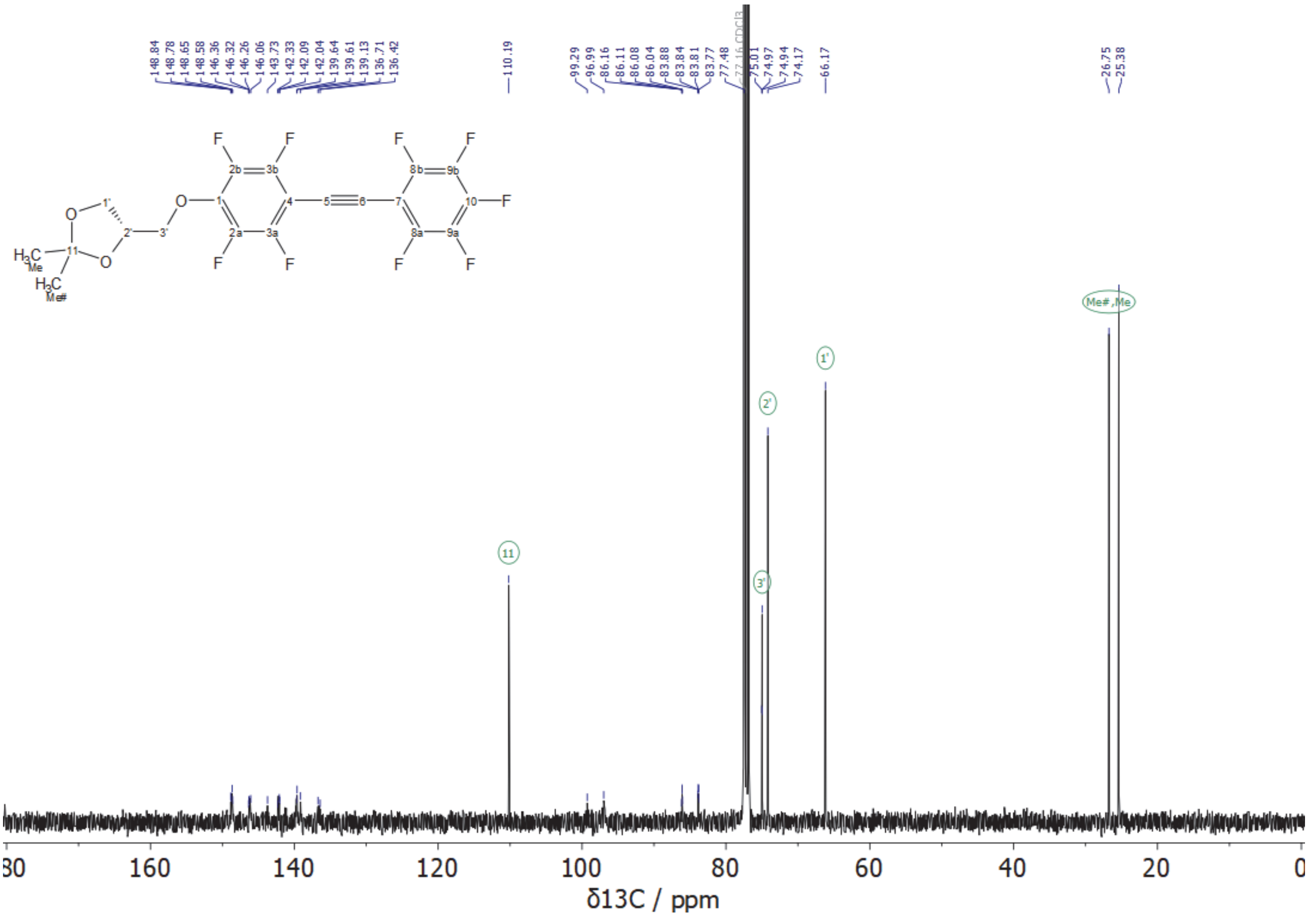
148.84
148.78
148.65
148.58
146.36
146.32
146.26
146.06
143.73
142.33
142.09
142.04
139.64
139.61
139.13
136.71
136.42
110.19
99.29
96.99
86.16
86.11
86.08
86.04
83.88
83.84
83.81
83.77
77.48
77.16 CDCl3
75.01
74.97
74.94
74.17
66.17
26.75
25.38
11
3'
2'
1'
Me#,Me
δ13C / ppm

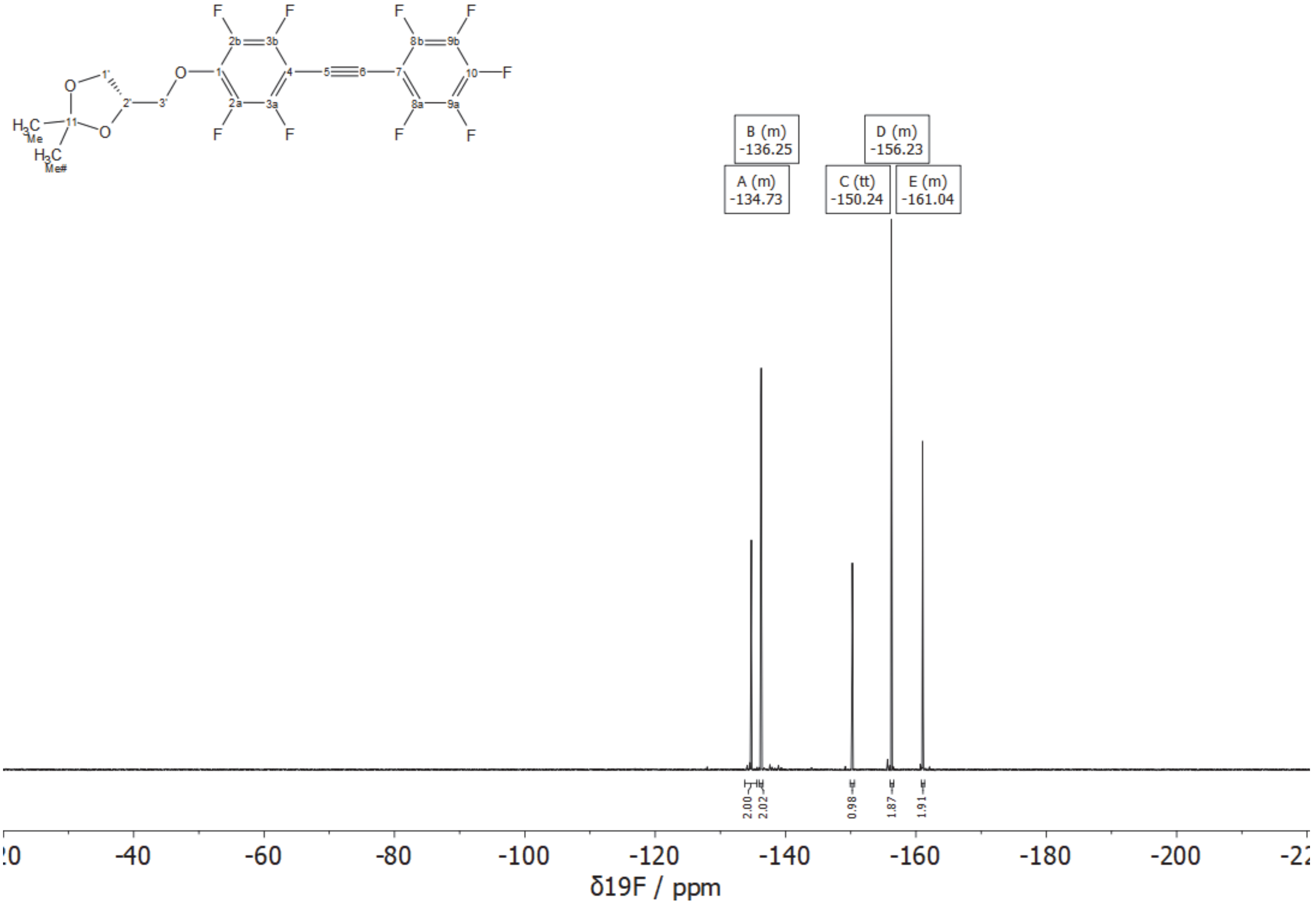
B (m)
-136.25
A (m)
-134.73
D (m)
-156.23
C (tt)
-150.24
E (m)
-161.04
2.00
2.02
0.98
1.87
1.91
δ19F / ppm

Compound **S11**

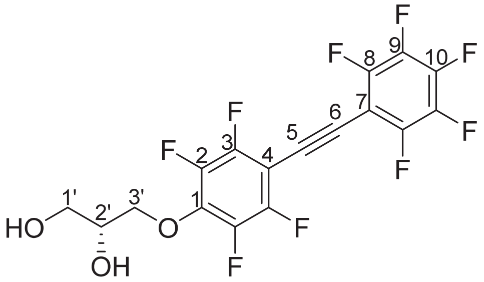

Compound **S10** (395 mg, 841 µmol, 1.00 eq.) was dissolved in THF (7 mL). 2 M Hydrochloric acid (7 mL) was added and the reaction mixture was stirred for 4 d at room temperature. The mixture was diluted with water (20 mL) and extracted with EtOAc (3x30 mL). The combined organic phases were washed with brine (20 mL) and dried over Na_2SO_4. The solvent was removed under reduced pressure and the residue was purified by column chromatography (hexane/EtOAc 3:2) to afford compound **S11** as a colorless solid (268 mg, 623 µmol, 74%).

^{1}H NMR (400 MHz, $CDCl_3$): *δ* (ppm) = 4.49 – 4.27 (m, 2H, 3'-H), 4.12 (p, *J* = 4.4 Hz, 1H, 2'-H), 3.93 – 3.69 (m, 2H, 1'-H), 2.58 (s_{br}, 1H, OH), 1.91 (s_{br}, 1H, OH);

^{13}C{^{1}H} NMR (100 MHz, $CDCl_3$): *δ* (ppm) = 86.11 – 85.84 (m), 84.12 – 83.71 (m), 76.35 (t, *J* = 3.4 Hz, 3'-C), 70.69 (2'-C), 63.10 (1'-C);

^{19}F{^{1}H} NMR (376 MHz, $CDCl_3$): *δ* (ppm) = -134.44 – -134.94 (m, 2F), -135.73 – -136.17 (m, 2F), -150.14 (tt, *J* = 20.8, 2.5 Hz, 1F), -156.27 – -156.68 (m, 2F), -160.85 – -161.20 (m, 2F);

HR-MS (ESI+): *m*/*z* calc. ($C_{17}H_7F_9O_3Na$, $[M+Na]^+$): 453.01437 , found: 453.01494.

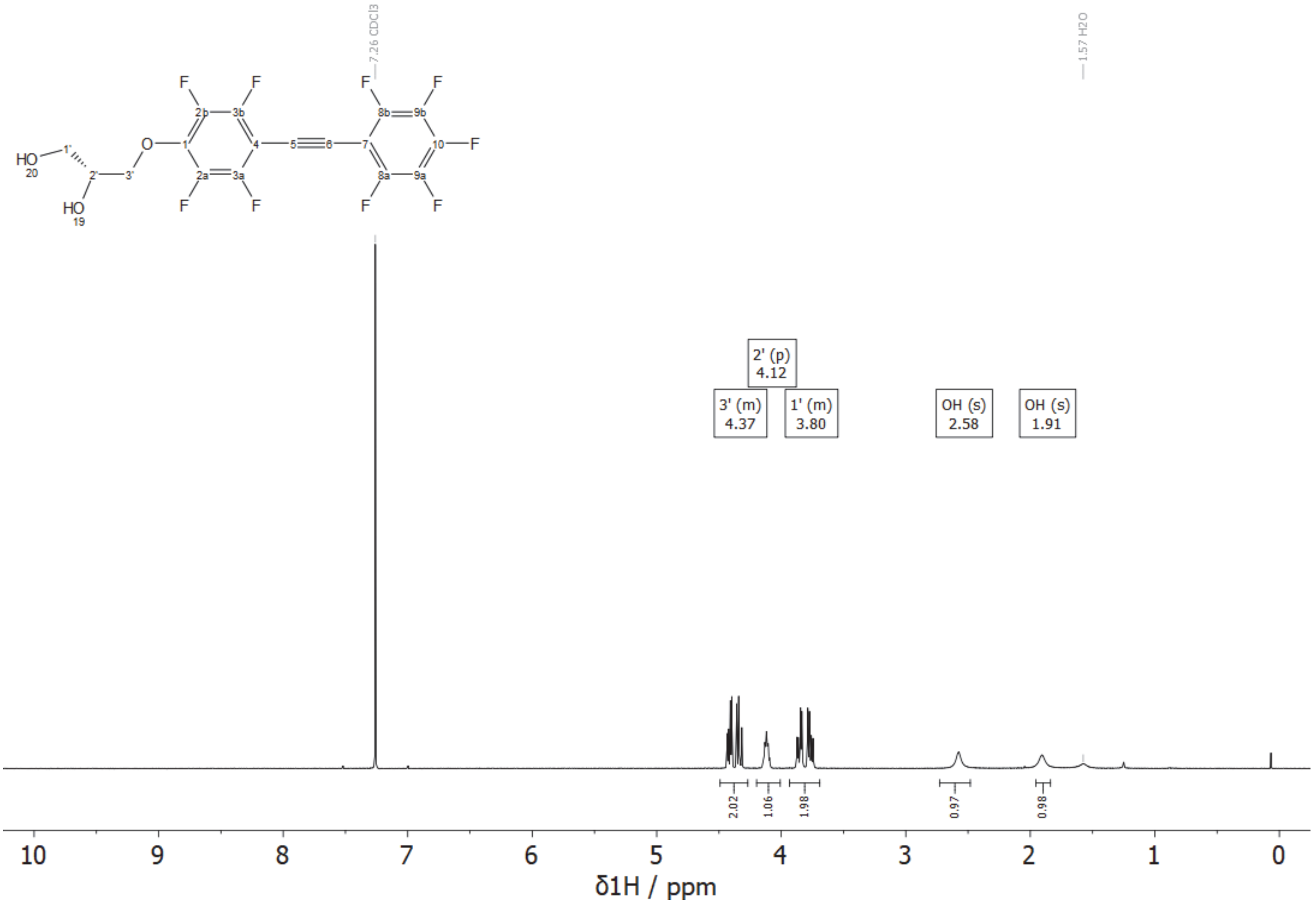

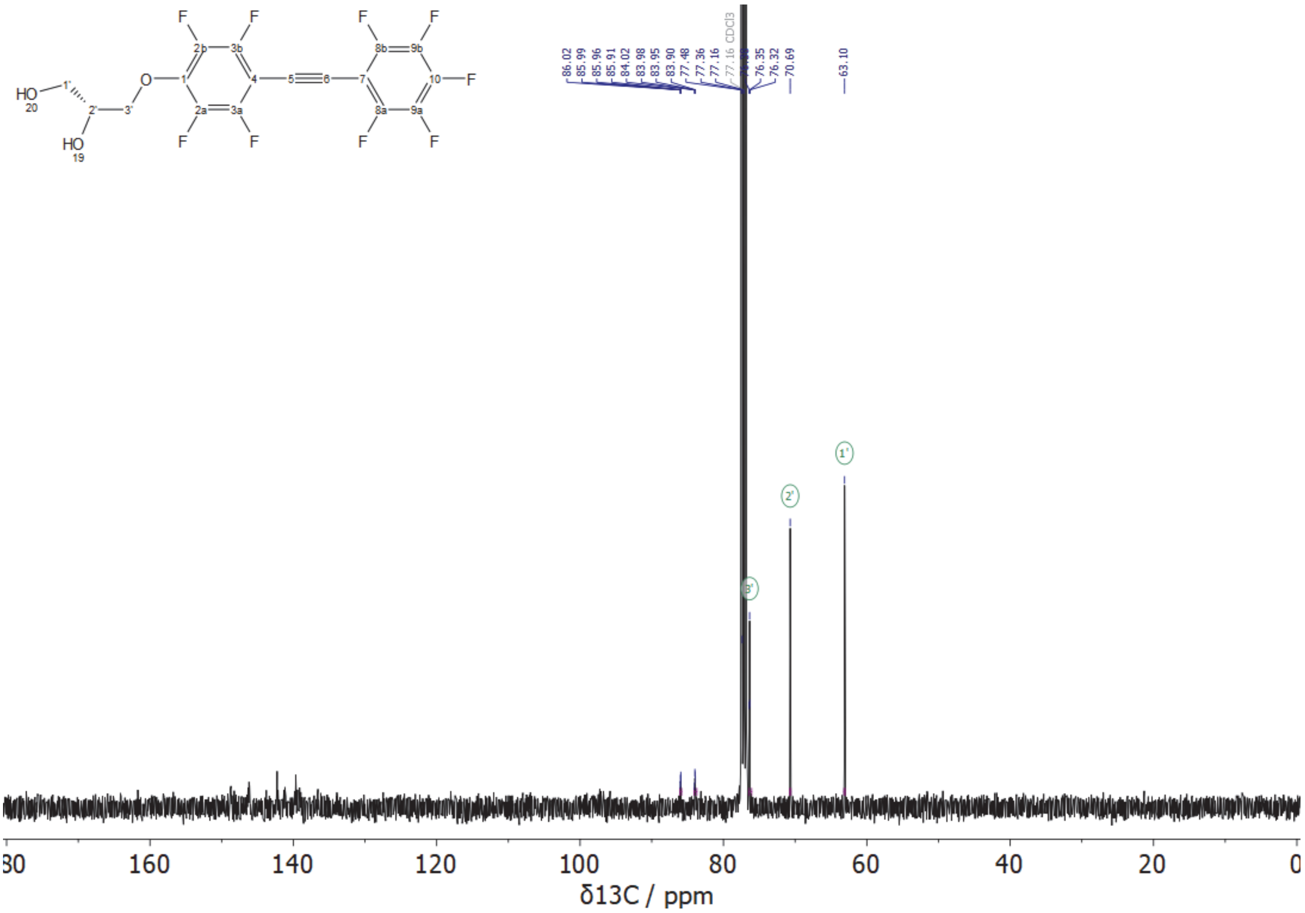
86.02
85.99
85.96
85.91
84.02
83.98
83.95
83.90
77.48
77.36
77.16
77.16 CDCl3
76.35
76.32
70.69
63.10
1'
2'
3'
δ13C / ppm

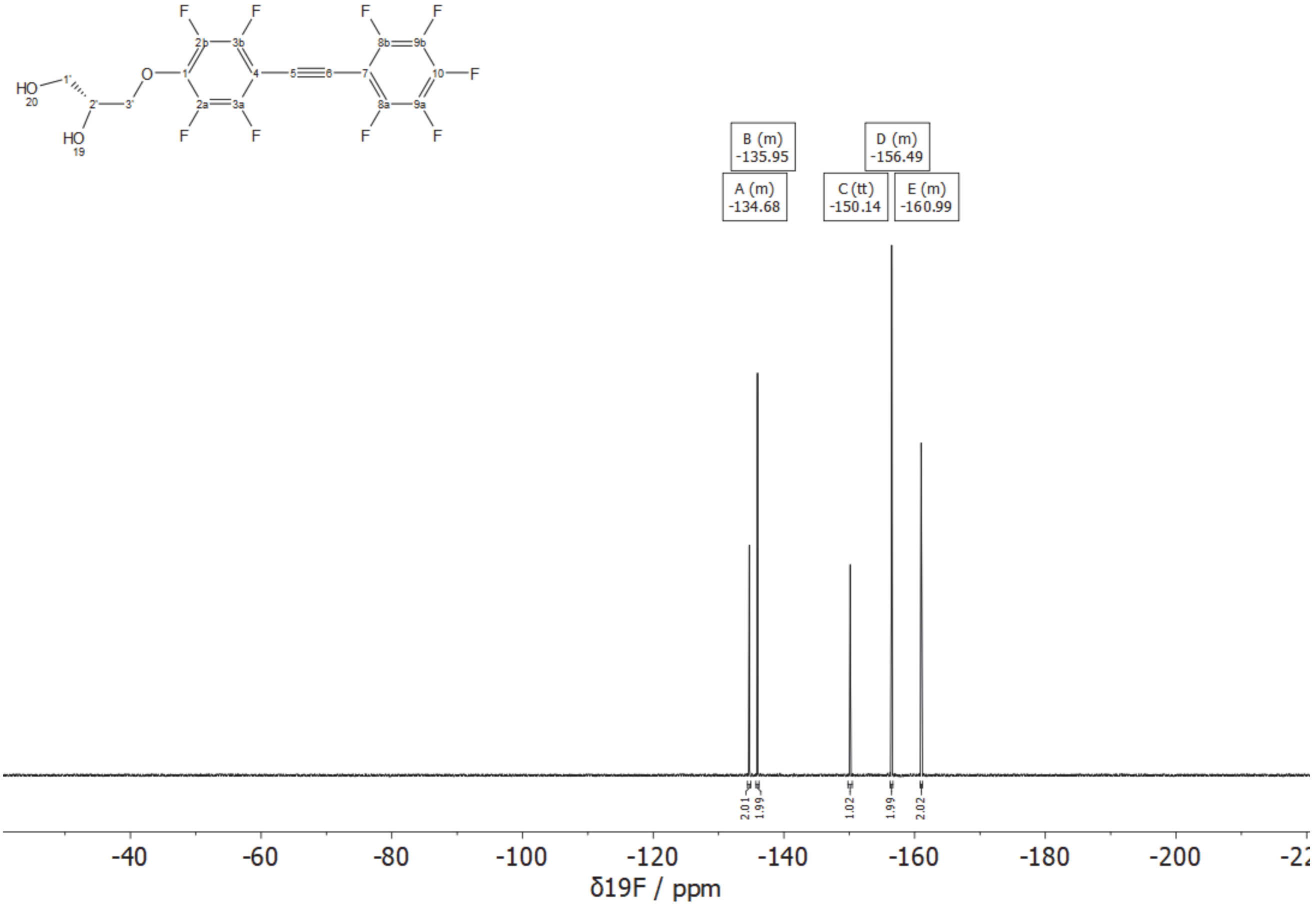
B (m)
-135.95
A (m)
-134.68
C (tt)
-150.14
D (m)
-156.49
E (m)
-160.99
2.01
1.99
1.02
1.99
2.02
δ19F / ppm

Compound **S12**

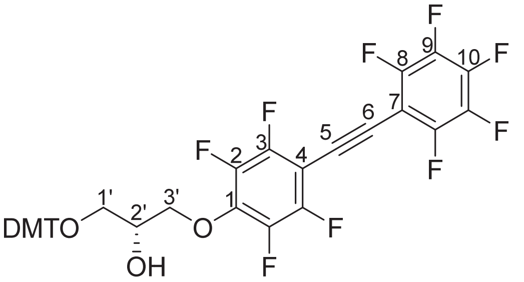

Under nitrogen atmosphere, compound **S11** (200 mg, 465 µmol, 1.00 eq.) was dissolved in anhydrous pyridine (6.5 mL). 4,4'-dimethoxytrityl chloride (205 mg, 604 mmol, 1.30 eq.) was added in small portions over a time period of 30 min and the reaction mixture was stirred at ambient temperature for 4 h. The mixture was diluted with DCM (50 mL), washed with sodium hydrogen carbonate solution (5%, 3x30 mL) and dried over Na_2SO_4.The solvent was removed under reduced pressure and the residue was purified twice by column chromatography (hexane/EtOAc 4:1+1% Et_3N and DCM/hexane 1:1+1% Et_3N) to afford compound **S12** as a colorless foam (277 mg, 378 µmol, 81%).

^{1}H NMR (400 MHz, $CDCl_3$): *δ* (ppm) = 7.43 – 7.38 (m, 2H, DMT-H), 7.34 – 7.25 (m, 6H, DMT-H), 7.25 – 7.20 (m, 1H, DMT-H), 6.86 – 6.80 (m, 4H, DMT-H), 4.45 – 4.30 (m, 2H, 3'-H), 4.15 – 4.05 (m, 1H, 2'H), 3.79 (s, 6H, DMT-H), 3.40 – 3.28 (m, 2H, 1'-H), 2.42 (d, *J* = 5.4 Hz, 1H, 2'-OH);

^{13}C{^{1}H} NMR (100 MHz, $CDCl_3$): *δ* (ppm) = 158.74 (DMT-C), 144.65 (DMT-C), 135.75 (DMT-C), 130.12 (DMT-C), 128.14 (DMT-C), 128.06 (DMT-C), 127.09 (DMT-C), 113.33 (DMT-C), 86.57 (DMT-C), 86.20 – 86.06 (m), 83.80 – 83.73 (m), 76.28 (t, *J* = 3.5 Hz, 3'-C), 70.01 (2'-C), 63.63 (1'-C), 55.36 (DMT-C).;

^{19}F{^{1}H} NMR (376 MHz, $CDCl_3$): *δ* (ppm) = -134.53 – -134.96 (m, 2F), -135.98 – -136.53 (m, 2F), -150.27 (tt, *J* = 20.8, 2.4 Hz, 1F), -155.97 – -156.83 (m, 2F), -160.68 – -161.51 (m, 2F);

HR-MS (ESI+): *m/z* calc. ($C_{38}H_{25}F_9O_5Na$, $[M+Na]^+$): 755.14505, found: 755.14427.

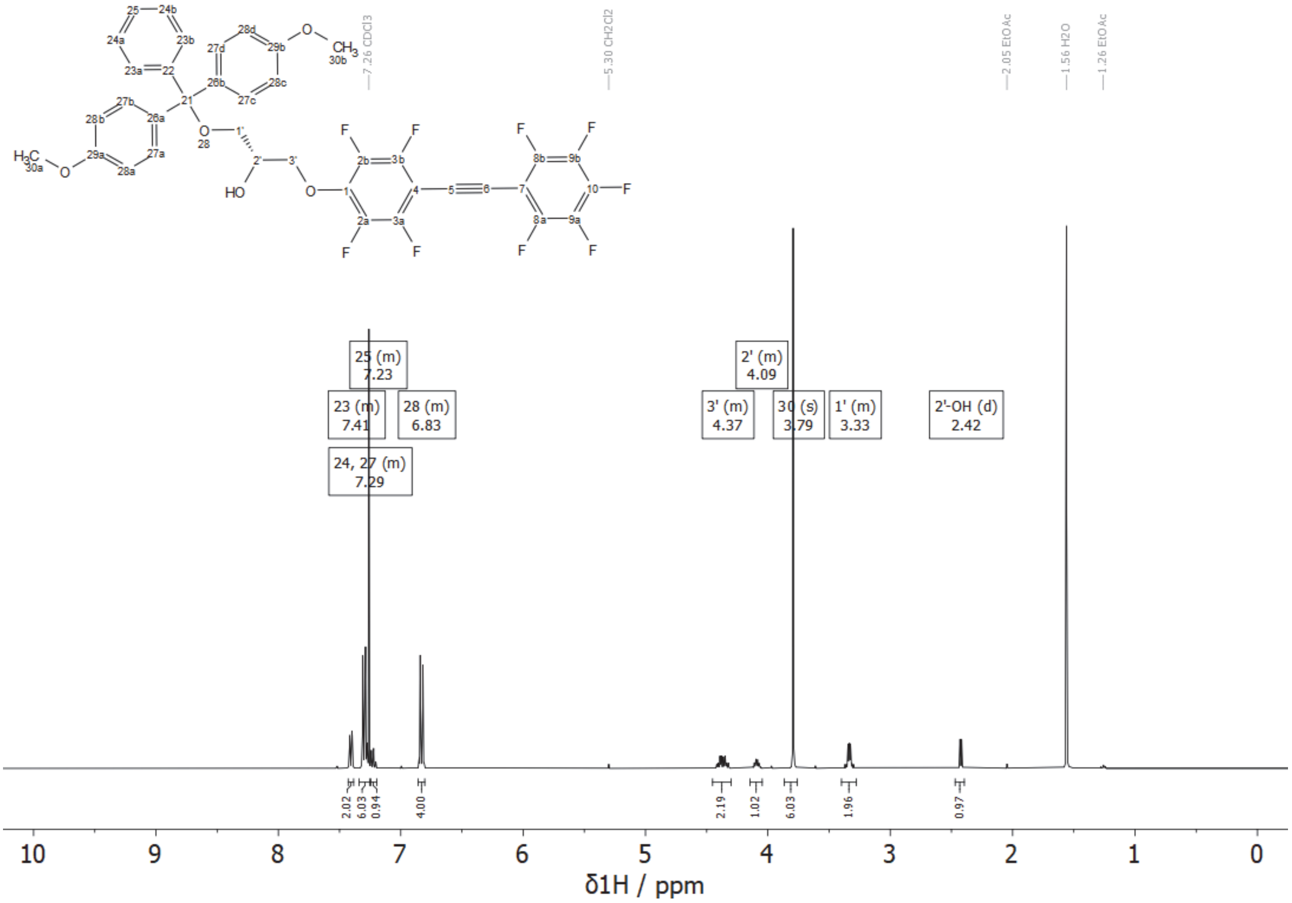

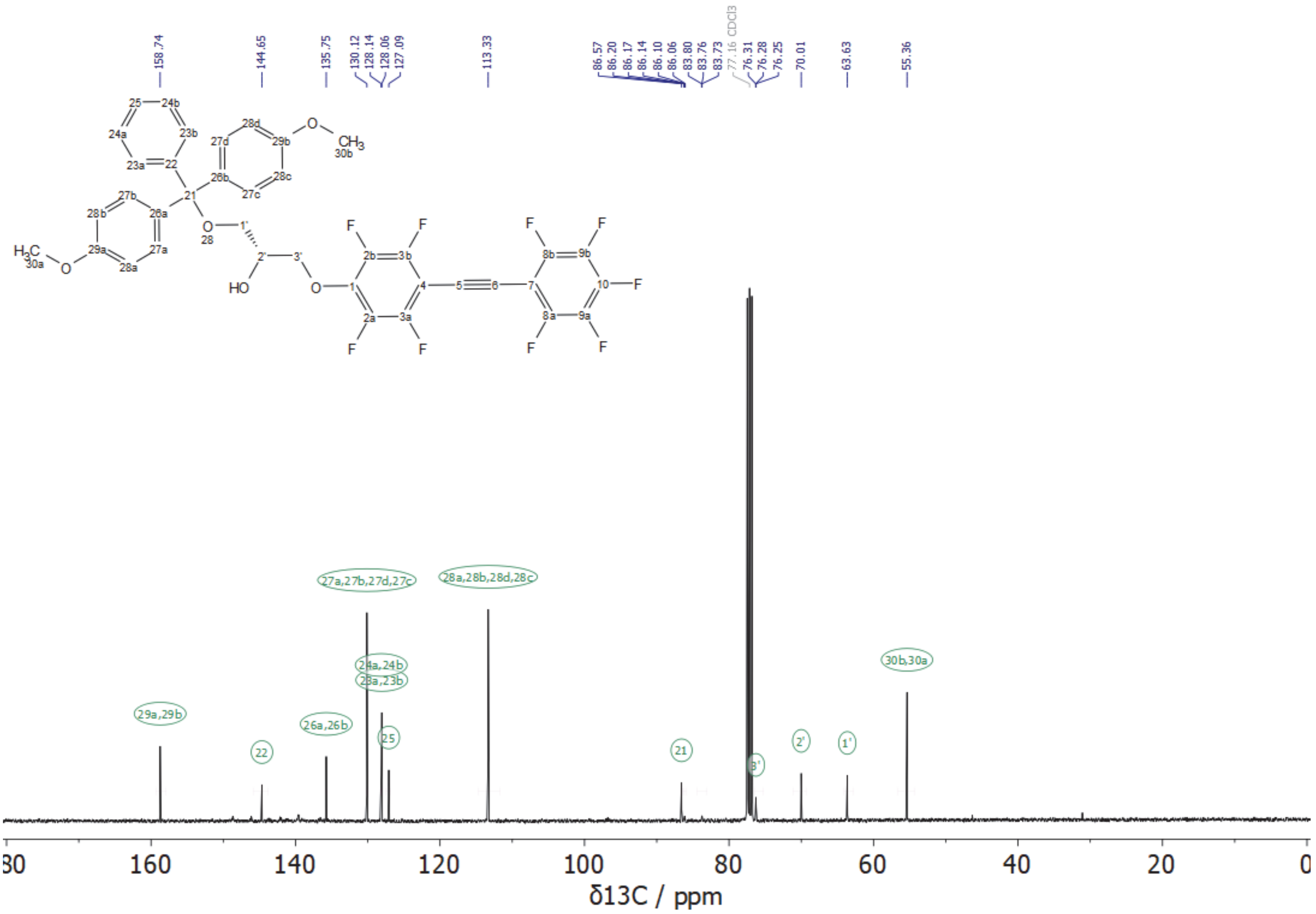
158.74
144.65
135.75
130.12
128.14
128.06
127.09
113.33
86.57
86.20
86.17
86.14
86.10
86.06
83.80
83.76
83.73
77.16 CDCl3
76.31
76.28
76.25
70.01
63.63
55.36
HO
29a,29b
22
26a,26b
27a,27b,27d,27c
24a,24b
23a,23b
25
28a,28b,28d,28c
21
3'
2'
1'
30b,30a
δ13C / ppm

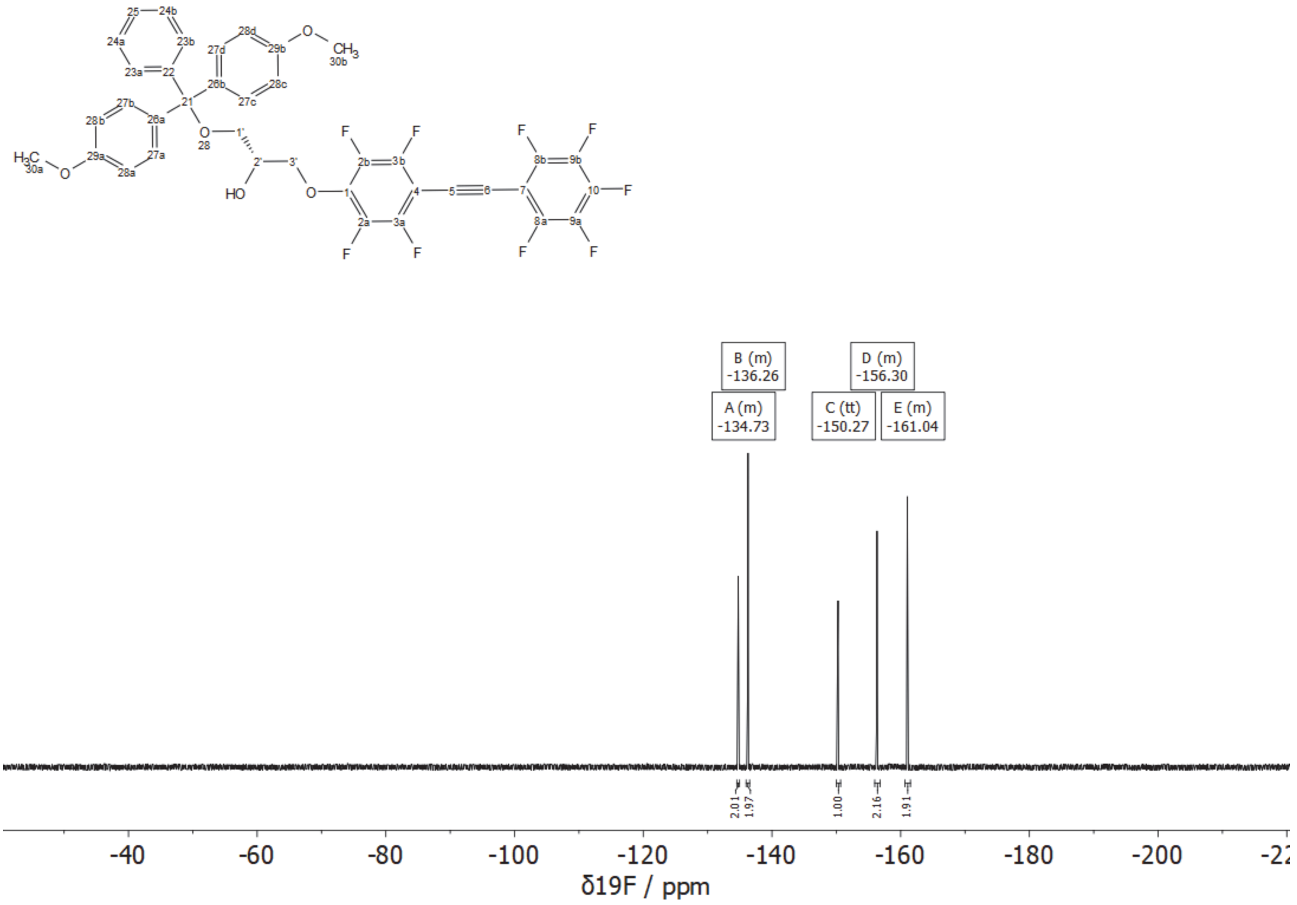
HO
B (m)
-136.26
A (m)
-134.73
D (m)
-156.30
C (tt)
-150.27
E (m)
-161.04
2.01
1.97
1.00
2.16
1.91
δ19F / ppm

Compound **S13** = GTFF-PA

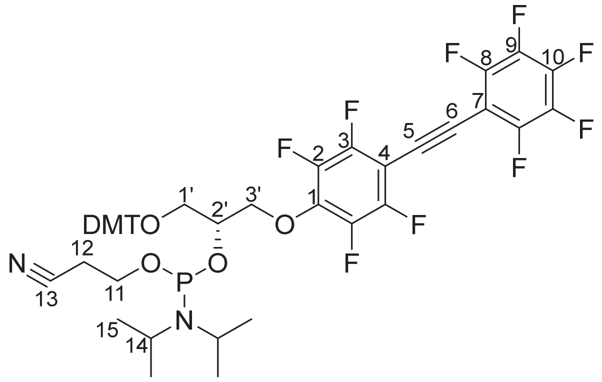

Compound **S12** (254 mg, 347 µmol, 1.00 eq.) was dissolved with DIPEA (354 µL, 269 mg, 2.08 mmol, 6.00 eq.) in anhydrous DCM (7 mL). After 20 min CEP-Cl (101 µL, 106 mg, 451 µmol, 1.30 eq.) was added. The reaction mixture was stirred additionally at ambient temperature for 3 h. The solvent was removed under reduced pressure and the residue was purified by column chromatography (hexane/EtOAc 5:1 + 1% Et_3N) to afford compound **S13** as a colorless foam (304 mg, 326 µmol, 94%).

^{1}H NMR (400 MHz, $CDCl_3$): *δ* (ppm) = 7.47 – 7.38 (m, 4H, DMT-H), 7.36 – 7.22 (m, 12H, DMT-H), 7.26 – 7.16 (m, 2H, DMT-H), 6.88 – 6.75 (m, 8H, DMT-H), 4.71 – 4.57 (m, 1H, 3'-H), 4.56 – 4.40 (m, 3H, 3'-H), 4.32 – 4.14 (m, 2H, 2'-H), 3.90 – 3.65 (m, 16H, DMT-H, 11-H), 3.64 – 3.52 (m, 2H, 14-H), 3.48 – 3.32 (m, 5H, 1'-H, 14-H), 3.25 (dd, *J* = 9.5, 6.6 Hz, 1H, 1'-H), 2.61 (t, *J* = 6.4 Hz, 2H, 12-H), 2.53 – 2.40 (m, 2H, 12-H), 1.28 – 0.99 (m, 24H, 15-H).;

^{13}C{^{1}H} NMR (100 MHz, $CDCl_3$): *δ* (ppm) = 158.65 (DMT-C), 158.63 (DMT-C), 144.83 (DMT-C), 136.06 (DMT-C), 136.00 (DMT-C), 135.97 (DMT-C), 135.95 (DMT-C), 130.19 (DMT-C), 130.16 (DMT-C), 130.13 (DMT-C), 128.26 (DMT-C), 128.21 (DMT-C), 127.95 (DMT-C), 126.98 (DMT-C), 126.93 (DMT-C), 117.75 (13-C), 117.67(13-C), 113.22 (DMT-C), 86.39 (DMT-C), 86.34 (DMT-C), 75.49 (3'-C), 72.77 (2'-C), 72.60 (2'-C), 72.41 (2'-C), 72.25 (2'-C), 63.14 (1'-C), 63.13 (1'-C), 63.10 (1'-C), 58.52 (11-C), 58.39 (11-C), 58.33 (11-C), 58.21 (11-C), 55.36 (DMT-C), 55.35 (DMT-C), 43.41 (14-C), 43.32 (14-C), 43.29 (14-C), 43.19 (14-C), 24.82 (15-C), 24.80 (15-C), 24.75 (15-C), 24.73 (15-C), 24.56 (15-C), 24.54 (15-C), 24.49 (15-C), 24.47 (15-C), 20.45 (12-C), 20.39 (12-C), 20.34 (12-C), 20.27 (12-C);

^{19}F{^{1}H} NMR (376 MHz, $CDCl_3$): *δ* (ppm) = -134.66 – -134.90 (m, 4F), -136.45 – -136.83 (m, 4F), -150.07 – -150.61 (m, 2F), -155.59 – -156.33 (m, 4F), -160.87 – -161.25 (m, 4F);

^{31}P{^{1}H} NMR (162 MHz, $CDCl_3$): *δ* (ppm) = 150.04 (t, *J* = 2.2 Hz, 1P), 149.72 (t, *J* = 2.1 Hz, 1P);

HR-MS (ESI+): *m/z* calc. ($C_{47}H_{42}F_9O_6N_2PNa$, $[M+Na]^+$): 955.25290, found: 955.25188.

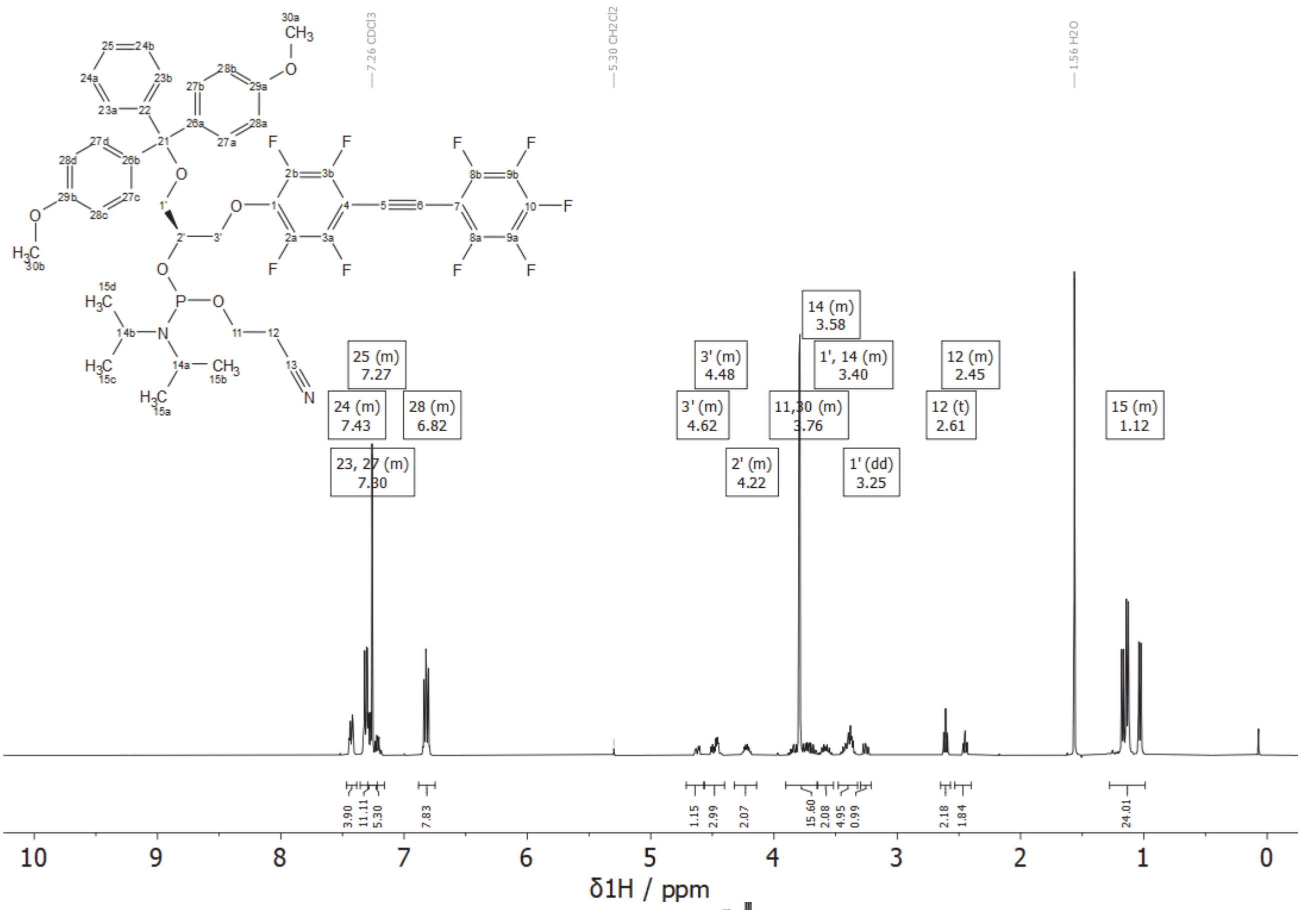
7.26 CDCl3
5.30 CH2Cl2
1.56 H2O
25 (m) 7.27
24 (m) 7.43
28 (m) 6.82
23, 27 (m) 7.30
3' (m) 4.48
3' (m) 4.62
2' (m) 4.22
14 (m) 3.58
1', 14 (m) 3.40
11,30 (m) 3.76
1' (dd) 3.25
12 (m) 2.45
12 (t) 2.61
15 (m) 1.12
δ1H / ppm

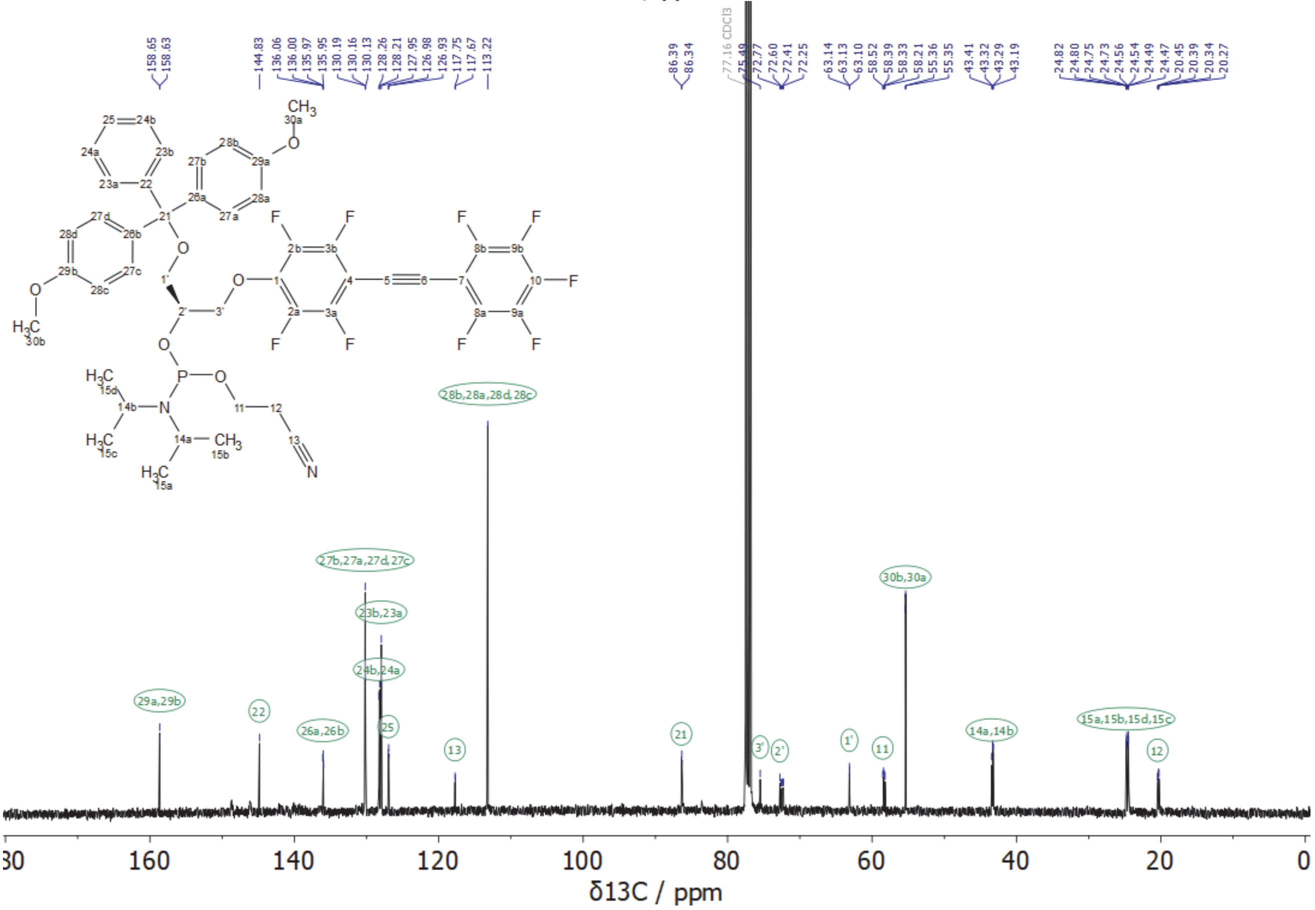
77.16 CDCl3
29a,29b
22
26a,26b
27b,27a,27d,27c
23b,23a
24b,24a
25
13
28b,28a,28d,28c
21
3'
2'
1'
11
30b,30a
14a,14b
15a,15b,15d,15c
12
δ13C / ppm

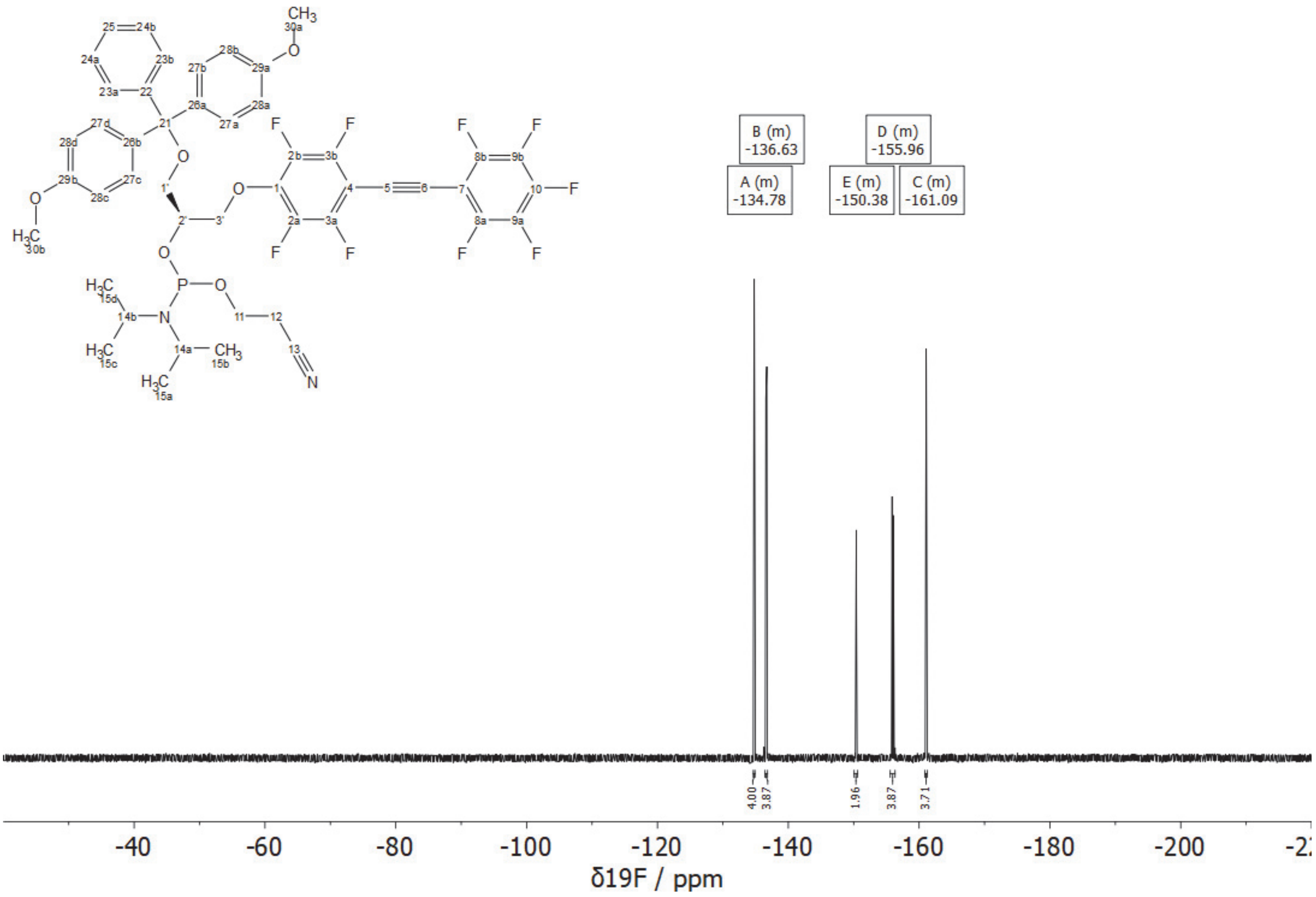
B (m)
-136.63
A (m)
-134.78
D (m)
-155.96
E (m)
-150.38
C (m)
-161.09
4.00
3.87
1.96
3.87
3.71
δ19F / ppm

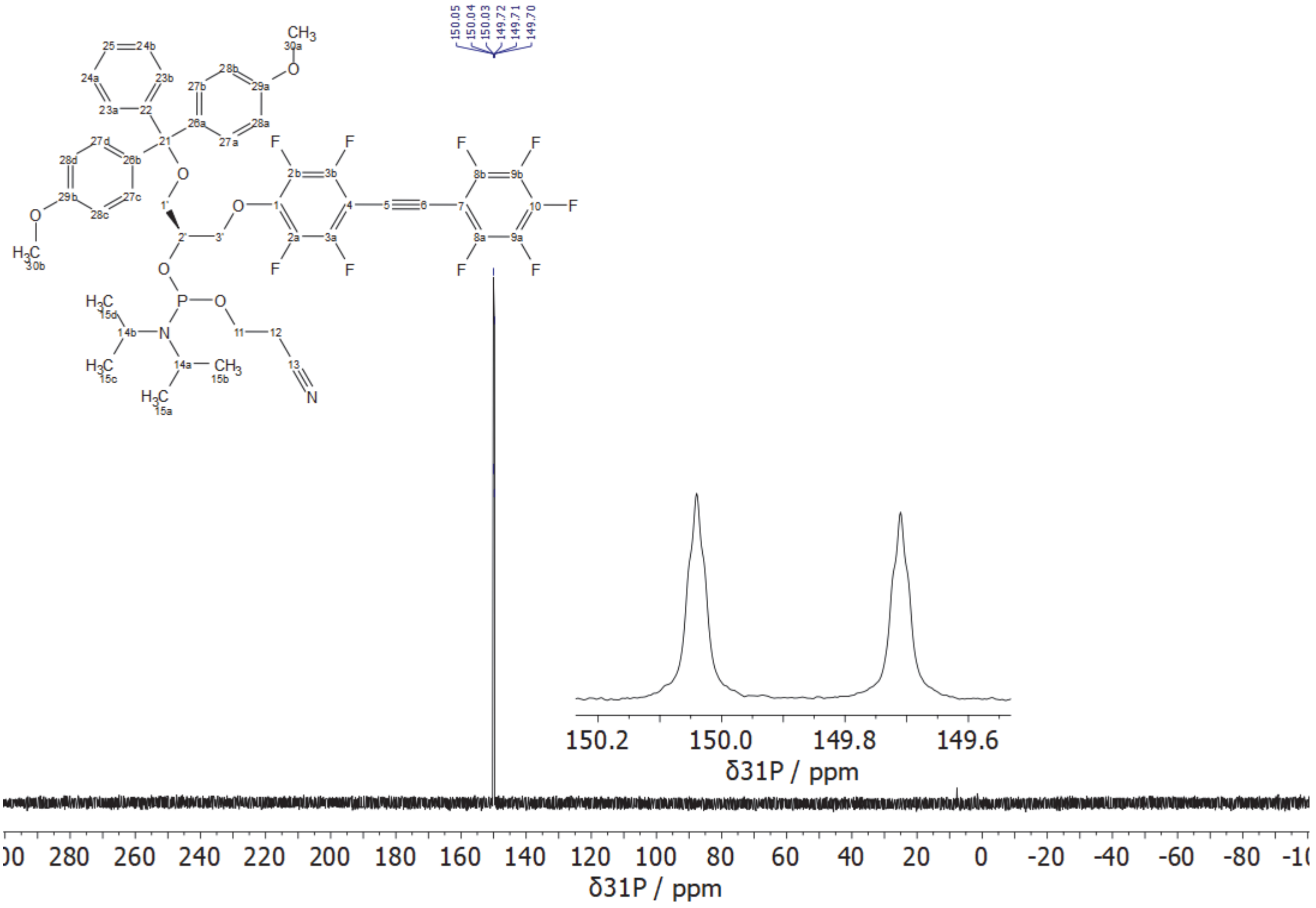
150.05
150.04
150.03
149.72
149.71
149.70
δ31P / ppm
δ31P / ppm

Compound **S14**

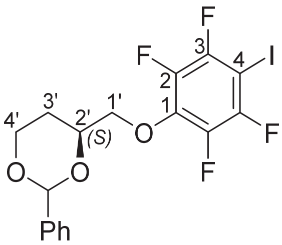

4-(*S*)- Hydroxmethyl-2-phenyl-1,3-dioxan (**3**) (1.00 g, 5.15 mmol, 1.00 eq.) and sodium hydride (60% oil dispersion, 206 mg, 5.15 mmol, 1.00 eq.) were dissolved in anhydrous THF (25 mL) and stirred at ambient temperature. Iodopentafluorobenzene (687 µL, 1.51 g, 5.15 mmol, 1.00 eq.) was added dropwise to the mixture. After 3 h additional iodopentafluorobenzene (138 µL, 303 mg, 1.03 mmol, 0.20 eq.) was added. The solvent was evaporated after 30 min and the residue was purified by column chromatography (hexane/EtOAc 9:1) to afford compound **S14** (1.61 mg, 3.43 mmol, 67%) as a colorless solid.

^{1}H NMR (400 MHz, $CDCl_3$): *δ* (ppm) = 7.42 – 7.29 (m, 5H, Ph-H), 5.54 (s, 1H, C*H*-Ph), 4.44 – 4.23 (m, 4H, 1'-H, 2'-H, 4'-H), 4.17 – 3.92 (m, 1H, 4'-H), 2.12 – 1.91 (m, 1H, 2'-H), 1.66 – 1.59 (m, 1H, 2'-H);

^{13}C{^{1}H} NMR (100 MHz, $CDCl_3$): *δ* (ppm) = 138.12 (Ph-C), 129.03 (Ph-C), 128.36 (Ph-C), 125.97 (Ph-C), 101.18 (*C*H-Ph), 76.66 (t, *J* = 3.1 Hz, 1'-C), 76.03 (2'-C), 66.65 (4'-C), 64.23 (t, *J* = 28.1 Hz, 4-C), 27.15 (3'-C);

^{19}F{^{1}H} NMR (376 MHz, $CDCl_3$): *δ* (ppm) = -121.33 – -121.66 (m, 2F, 3-F), -153.54 – -154.31 (m, 2F, 2-F);

HR-MS (ESI+): *m*/*z* calc. ($C_{17}H_{13}F_4O_3NaI$, $[M+Na]^+$): 490.97378, found: 490.97517.

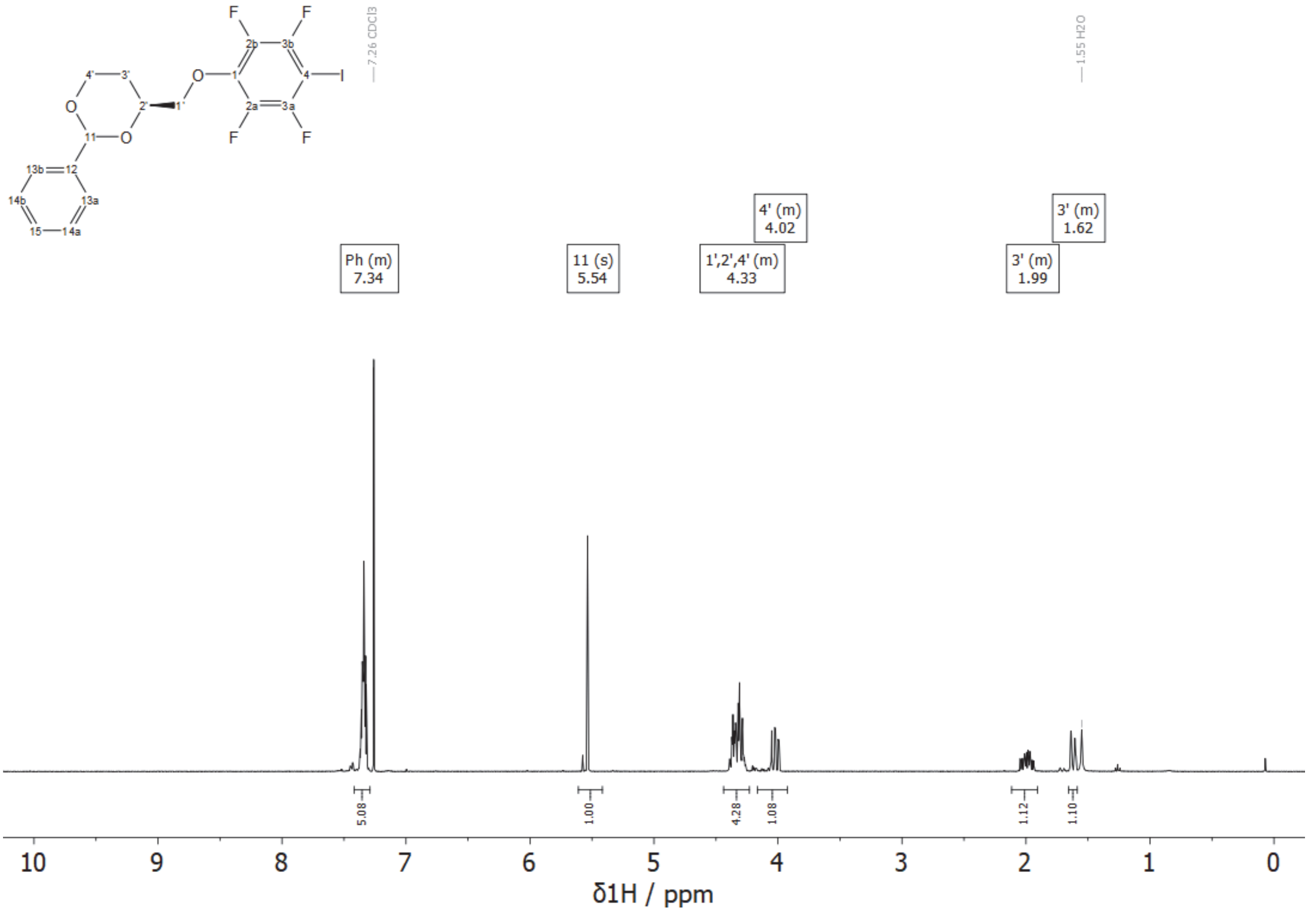

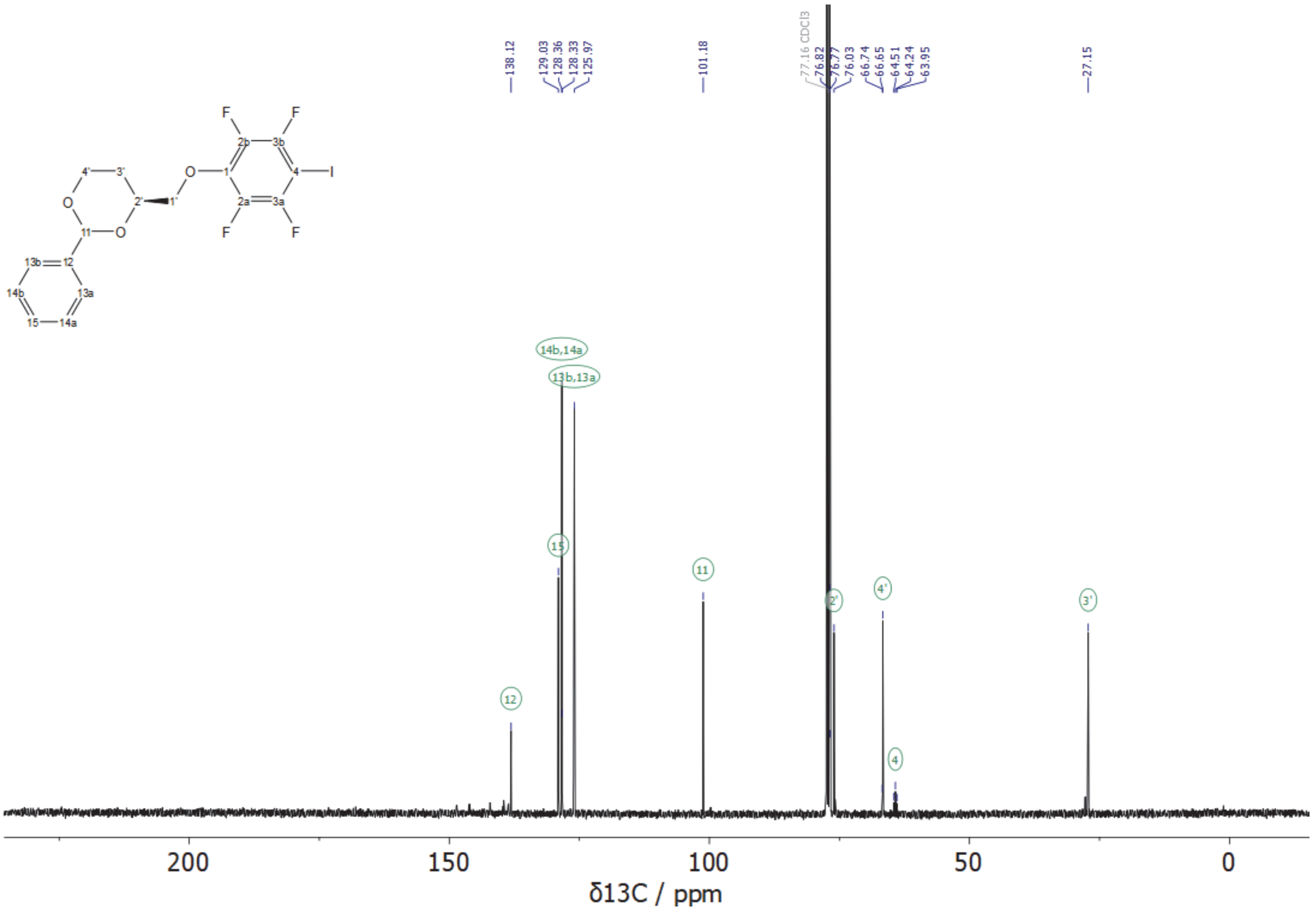
138.12
129.03
128.36
128.33
125.97
101.18
77.16 CDCl3
76.82
76.77
76.03
66.74
66.65
64.51
64.24
63.95
27.15
14b,14a
13b,13a
15
11
12
2'
4'
4
3'
200
150
100
50
0
δ13C / ppm

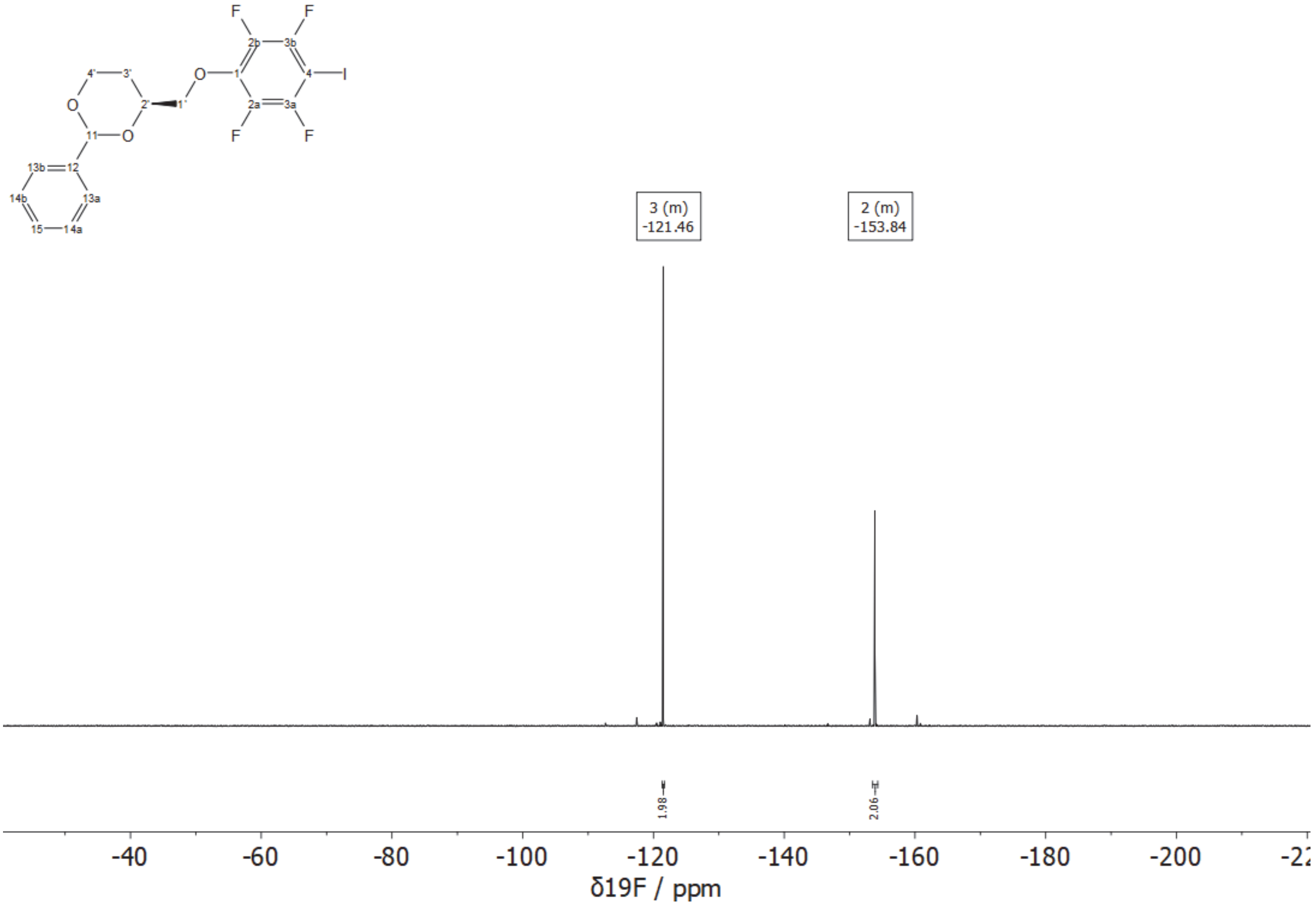
3 (m)
-121.46
2 (m)
-153.84
1.98
2.06
-40
-60
-80
-100
-120
-140
-160
-180
-200
δ19F / ppm

Compound **S15**

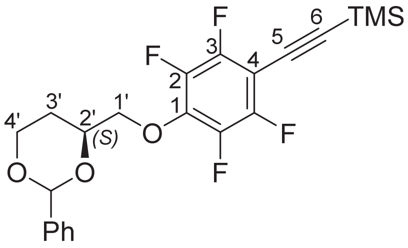

Under nitrogen atmosphere, CuI (51.5 mg, 271 µmol, 10 mol%) and $Pd(PPh_3)_4$ (93.8 mg, 81.1 µmol, 3.0 mol%) were dissolved in argon-purged Et_3N (14 mL). A solution of compound **S14** (1.27 g, 2.71 mmol, 1 eq.) in argon-purged Et_3N (11 mL) was added and stirred at ambient temperature for 10 min. Then, trimethylsilylacetylene (487 µL, 345 mg, 3.52 mmol, 1.3 eq) was added and stirring was continued for 3 h at 60°C. The precipitate was filtered off and washed with Et_2O. The filtrate was washed with a saturated solution of ammonium chloride (2x50 mL) and brine (2x50 mL). The organic phases were dried over Na_2SO_4, evaporated and the residue was purified by column chromatography (hexane/EtOAc 20:1–15:1) to afford compound **S15** as a brown oil (1.07 g, 2.44 mmol, 90%).

^{1}H NMR (400 MHz, $CDCl_3$): *δ* (ppm) = 7.46 – 7.28 (m, 5H, Ph-H), 5.54 (s, 1H, C*H*-Ph), 4.43 – 4.24 (m, 4H, 1'-H, 2'-H, 4'H), 4.10 – 3.95 (m, 1H, 4'-H), 2.07 – 1.91 (m, 1H, 3'-H), 1.67 – 1.58 (m, 1H, 3'-H), 0.28 (s, 9H, TMS);

^{13}C{^{1}H} NMR (100 MHz, $CDCl_3$): *δ* (ppm) = 149.21 – 148.81 (m), 146.71 – 146.31 (m), 142.44 – 142.02 (m), 140.01 – 139.56 (m), 138.97 – 138.42 (m), 138.15 (Ph-C), 129.02 (Ph-C), 128.36 (Ph-C), 126.01 (Ph-C), 107.73 (t, *J* = 3.7 Hz, 6-C), 101.21 (*C*H-Ph), 98.47 (t, *J* = 18.6 Hz), 88.58 (t, *J* = 3.7 Hz, 5-C), 76.87 (1'-C), 75.89 (2'-C), 66.66 (4'-C), 27.22 (3'-C), -0.23 (TMS).

^{19}F{^{1}H} NMR (376 MHz, $CDCl_3$): *δ* (ppm) = -134.91 – -140.25 (m, 2F, 3-F), -155.45 – -158.93 (m, 2F, 2-F);

HR-MS (ESI+): *m*/*z* calc. ($C_{22}H_{22}F_4O_3NaSi$, $[M+Na]^+$): 461.11665, found: 461.11709.

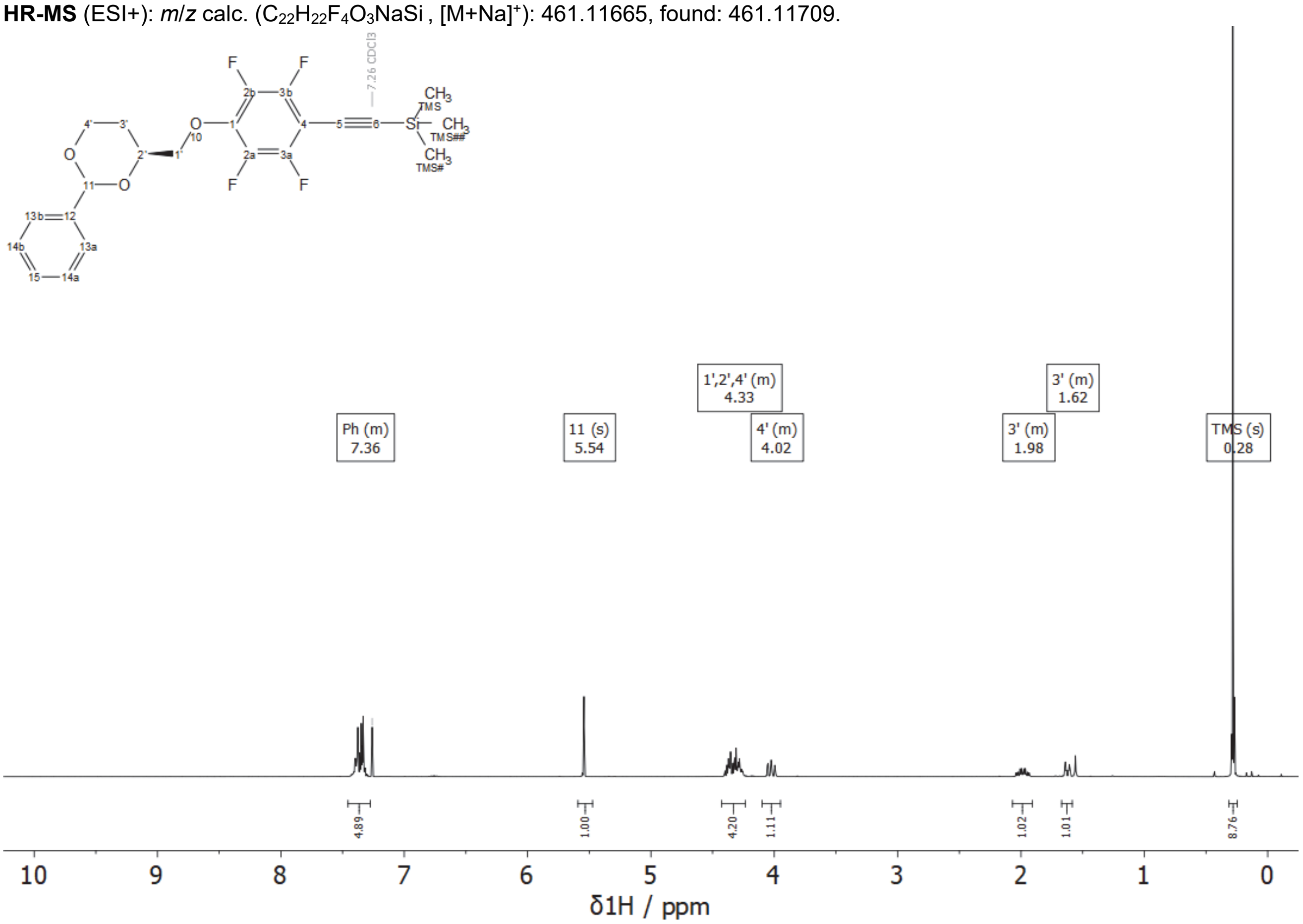

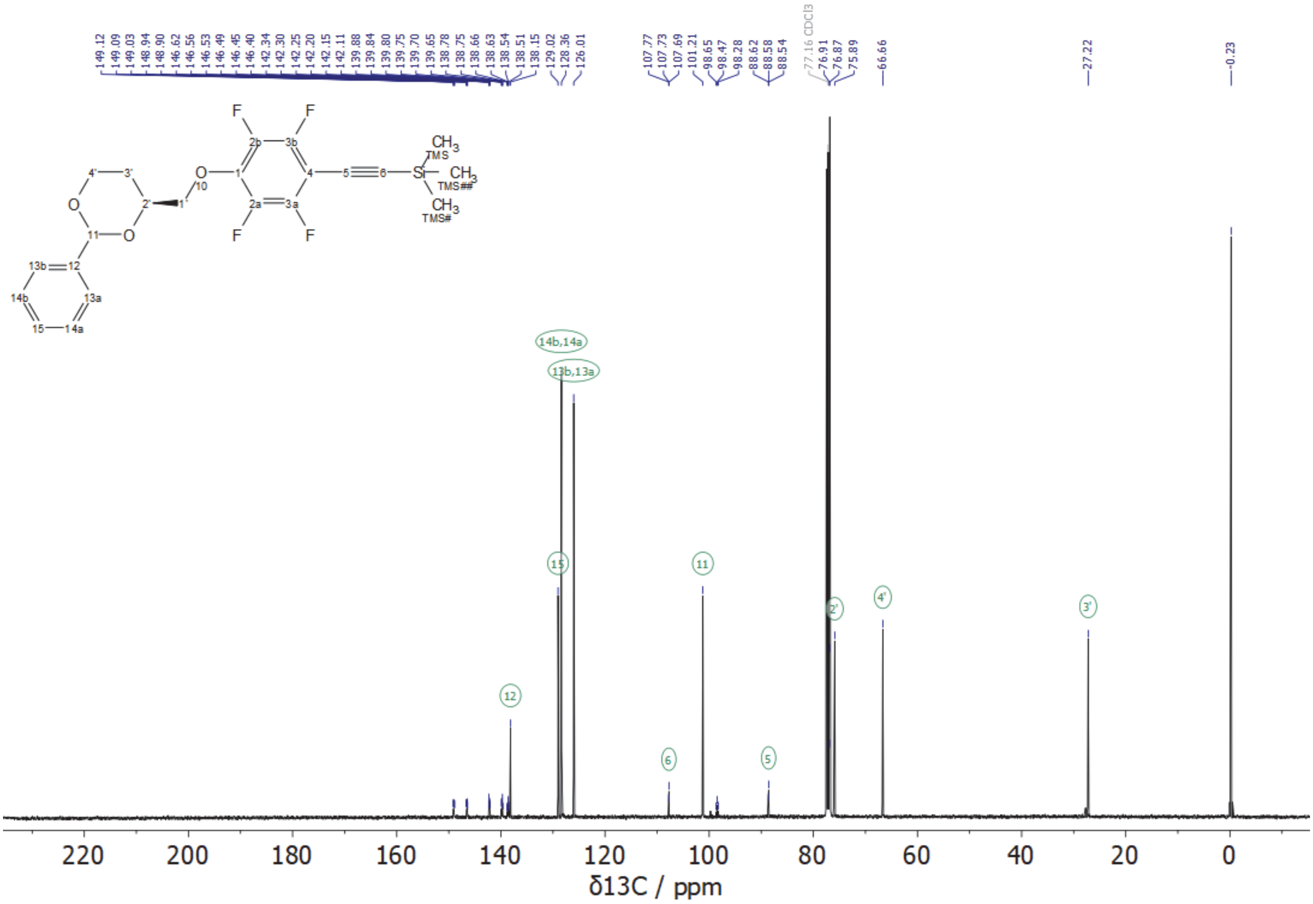
149.12
149.09
149.03
148.94
148.90
146.62
146.56
146.53
146.49
146.45
146.40
142.34
142.30
142.25
142.20
142.15
142.11
139.88
139.84
139.80
139.75
139.70
139.65
138.78
138.75
138.66
138.63
138.54
138.51
138.15
129.02
128.36
126.01
107.77
107.73
107.69
101.21
98.65
98.47
98.28
88.62
88.58
88.54
77.16 CDCl3
76.91
76.87
75.89
66.66
27.22
-0.23
14b,14a
13b,13a
15
11
12
6
5
2'
4'
3'
δ13C / ppm

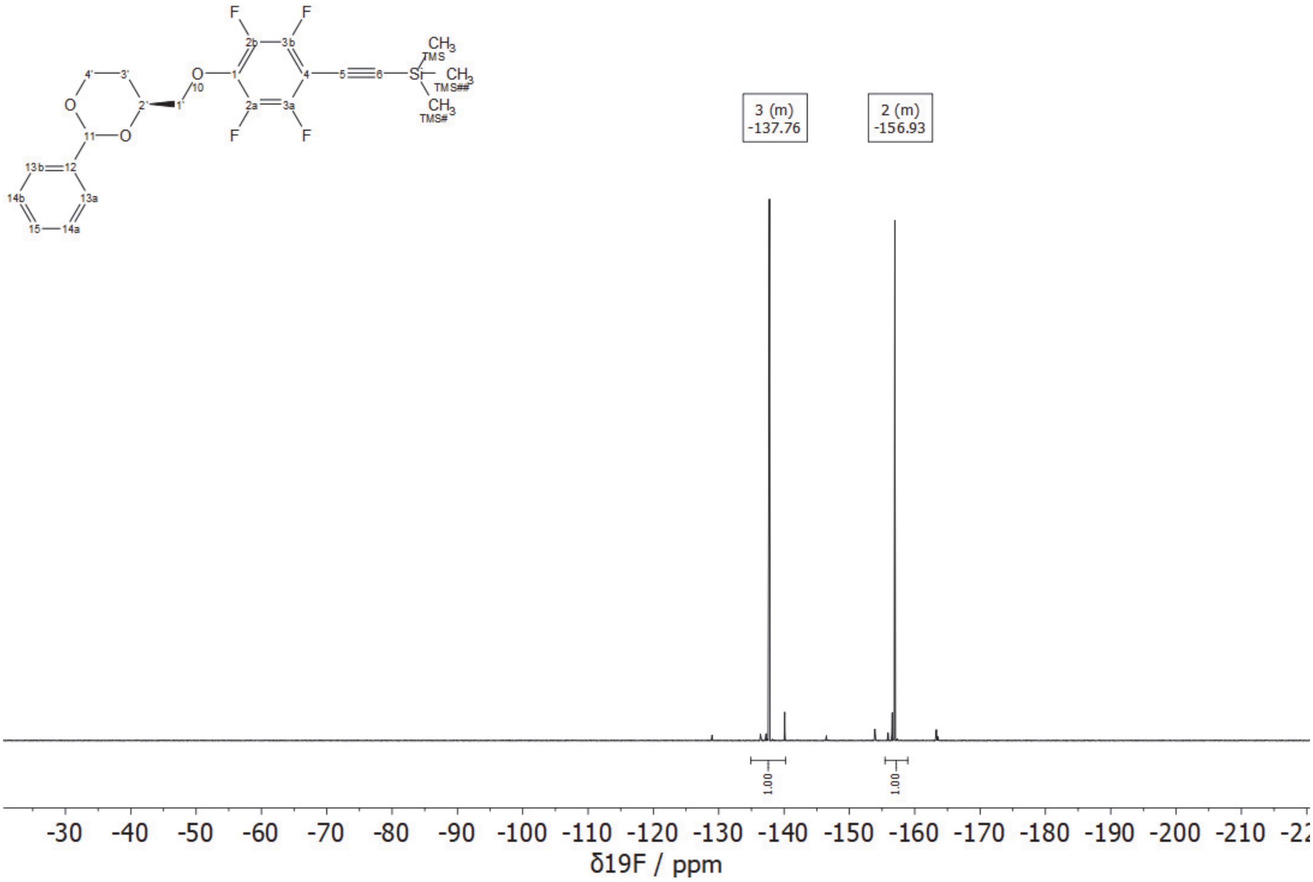
3 (m)
-137.76
2 (m)
-156.93
1.00
1.00
δ19F / ppm

Compound **S16**

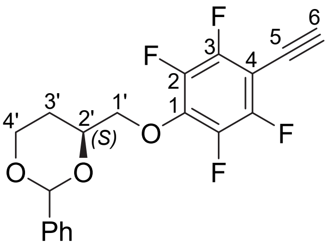

Compound **S15** (371 mg, 846 µmol, 1 eq.) was dissolved in THF/MeOH (1:1, 8 mL). K_2CO_3 (234 mg, 1.69 mmol, 2.00 eq.) was added and the mixture was stirred for 2.5 h at room temperature. The suspension was diluted with water (30 mL), extracted with Et_2O (3x50 mL) and washed with brine (2x30 mL). The organic phases were dried over $MgSO_4$, evaporated and the residue was purified by column chromatography (hexane/EtOAc 20:1–15:1) to afford compound **S16** as a brown solid (267 mg, 729 µmol, 86%).

^{1}H NMR (400 MHz, $CDCl_3$): *δ* (ppm) = 7.41 – 7.29 (m, 5H, Ph-H), 5.54 (s, 1H, C*H*-Ph), 4.46 – 4.24 (m, 4H, 1'-H, 2'-H, 4'-H), 4.02 (ddd, J = 12.3, 11.5, 2.6 Hz, 1H, 4'-H), 3.55 (t, *J* = 0.8 Hz, 1H, 6-H), 2.05 – 1.91 (m, 1H, 3'-H), 1.66 – 1.58 (m, 1H, 3'-H);

^{13}C{^{1}H} NMR (100 MHz, $CDCl_3$): *δ* (ppm) = 149.60 – 149.10 (m), 147.08 – 146.65 (m), 142.55 – 141.88 (m), 140.00 – 139.42 (m), 139.48 – 138.87 (m), 138.13 (Ph-C), 129.06 (Ph-C), 128.37 (Ph-C), 125.99 (Ph-C), 101.21 (*C*H-Ph), 97.40 – 96.84 (m, 4-C), 88.68 (t, *J* = 3.8 Hz, 6-C), 76.89 (1'-C), 75.96 (2'-C), 68.77 (t, *J* = 4.0 Hz, 5-C), 66.65 (4'-C), 27.16 (3'-C);

^{19}F{^{1}H} NMR (376 MHz, $CDCl_3$): *δ* (ppm) = -134.91 – -140.00 (m, 2F, 3-F), -154.94 – -160.80 (m, 2F, 2-F);

HR-MS (ESI+): *m*/*z* calc. ($C_{19}H_{14}F_4O_3Na$, $[M+Na]^+$):389.07713, found:389.07619.

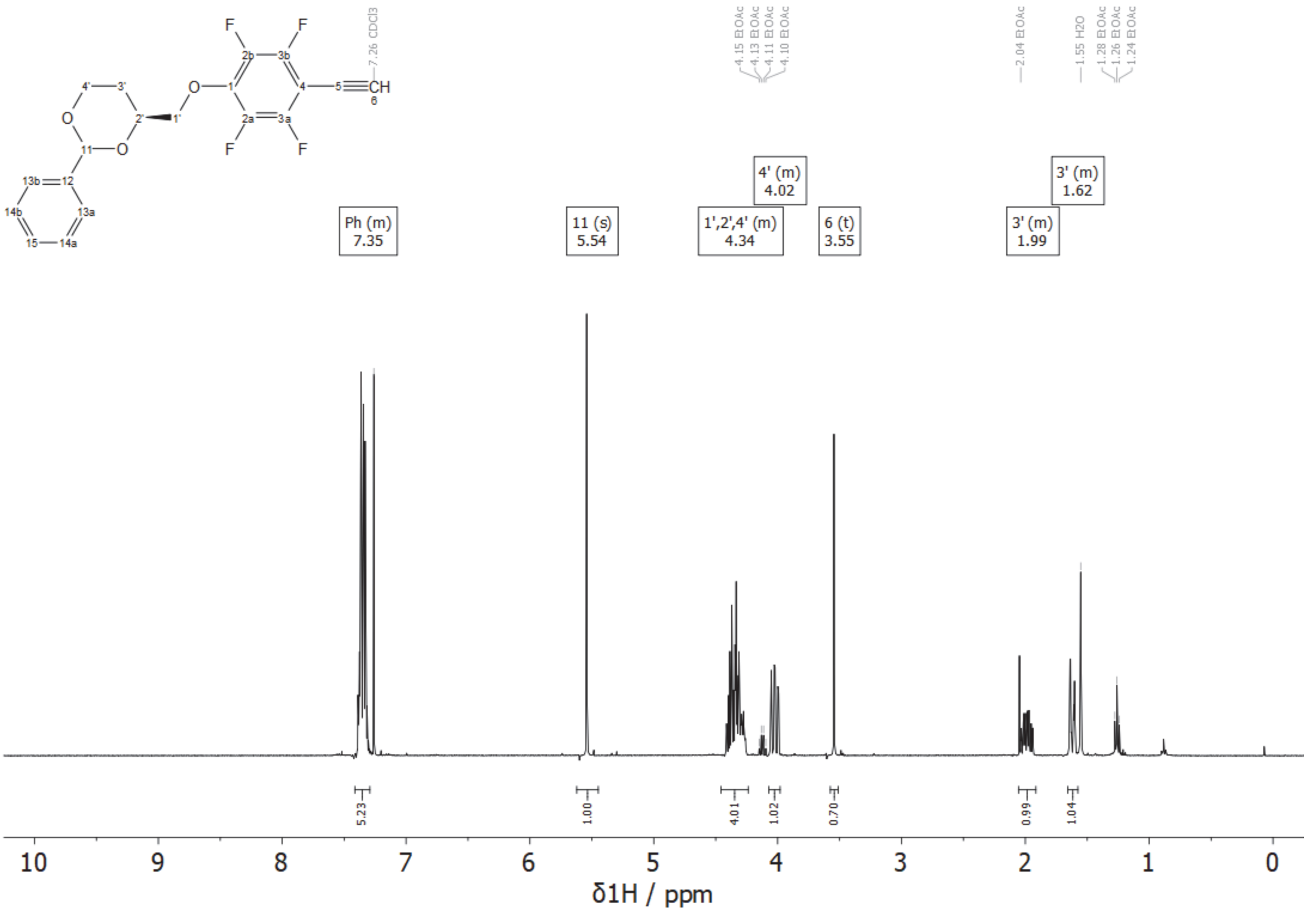

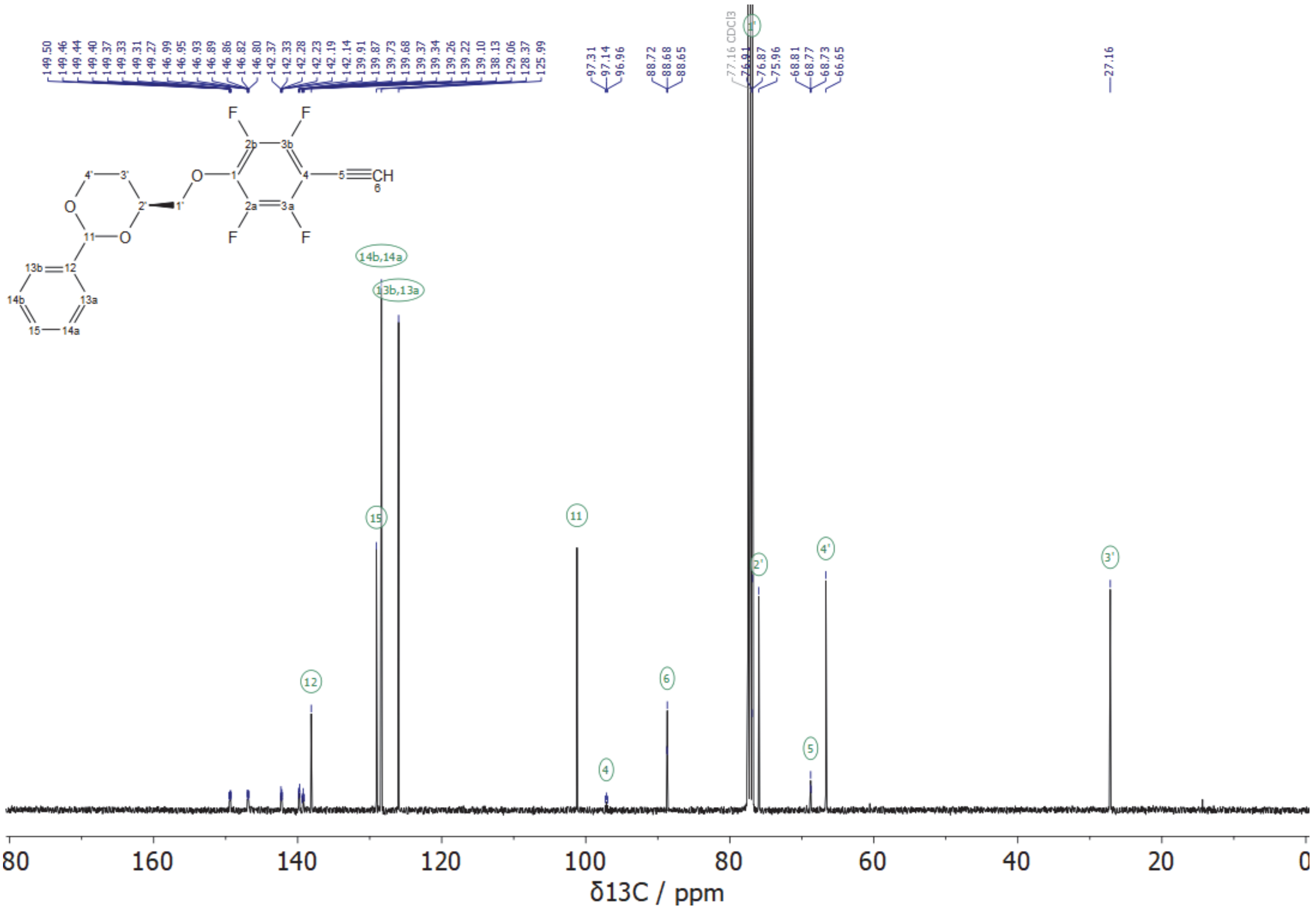
δ13C / ppm

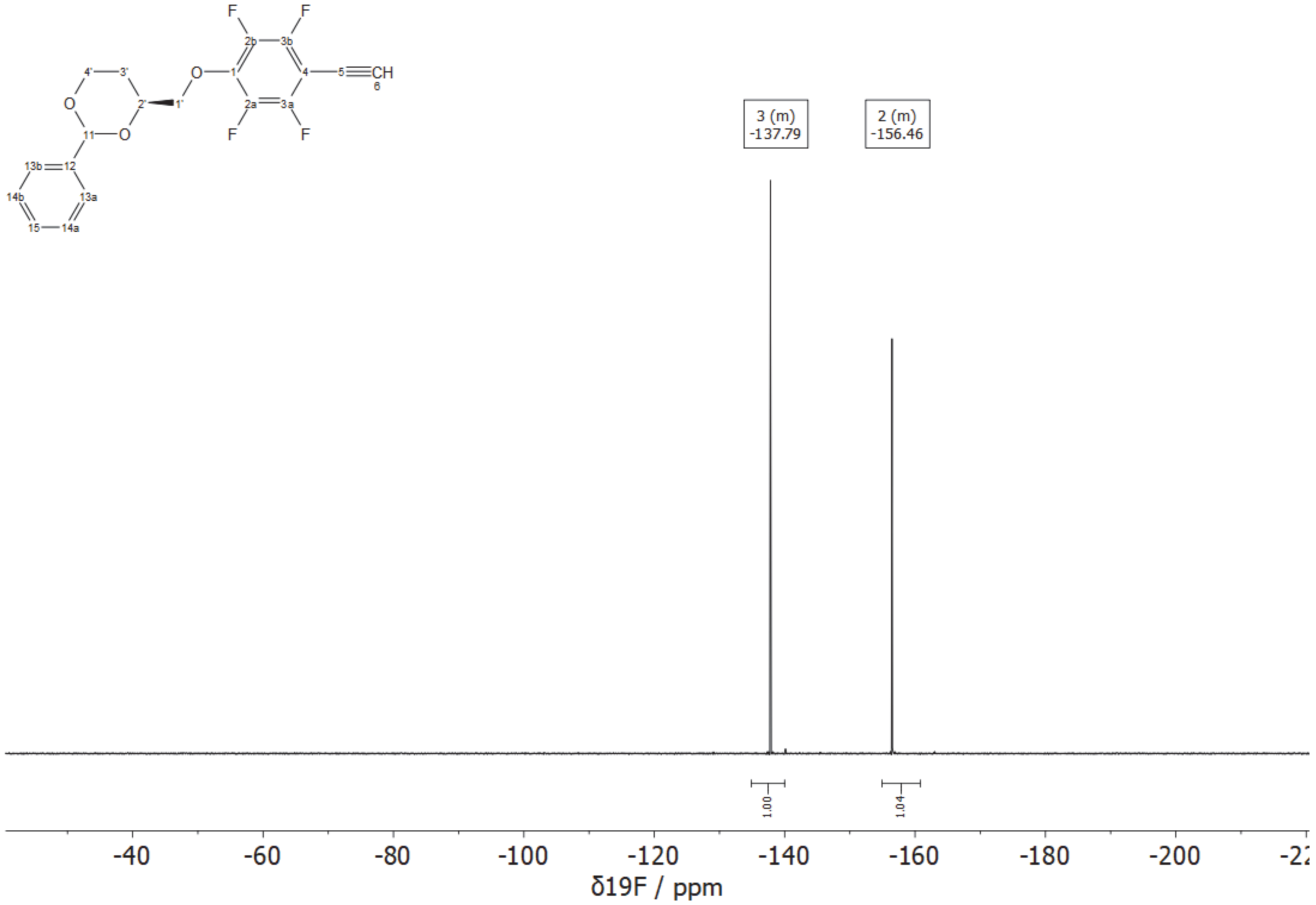
3 (m)
-137.79
2 (m)
-156.46
δ19F / ppm

Compound **S17**

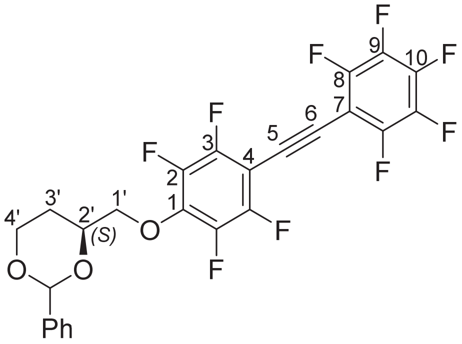

Under nitrogen atmosphere, CuI (34.0 mg, 179 µmol, 10 mol%) and $Pd(PPh_3)_4$ (62.2 mg, 53.6 µmol, 3.0 mol%) were dissolved in argon-purged Et_3N (13 mL). A solution of compound **16** (654 mg, 1.79 mmol, 1.00 eq.) in argon-purged Et_3N (7.5 mL) was added and stirred at ambient temperature for 10 min. Then, iodopentafluorobenzene (262 µL, 578 mg, 1.96 mmol, 1.10 eq) was added and stirring was continued for 3 h at 80°C. The mixture was diluted with Et_2O (50 mL) and washed with a saturated solution of ammonium chloride (2x30 mL). The aqueous phase was extracted again with Et_2O (2x30 mL). The organic phases were dried over Na_2SO_4, evaporated and the residue was purified by column chromatography (hexane/EtOAc 20:1) to afford compound **S17** as a yellow solid (744 g, 1.40 mmol, 78%).

^{1}H NMR (400 MHz, $CDCl_3$): *δ* (ppm) = 7.40 – 7.31 (m, 5H, Ph-H), 5.54 (s, 1H, C*H*-Ph), 4.50 – 4.25 (m, 4H, 1'-H, 2'-H, 4'-H), 4.09 – 3.96 (m, 1H, 4'-H), 2.08 – 1.92 (m, 1H, 3'H), 1.70 – 1.57 (m, 1H, 3'-H);

^{13}C{^{1}H} NMR (100 MHz, $CDCl_3$): *δ* (ppm) = 138.09 (12-C), 129.08 (15-C), 128.36 (14-C), 125.97 (13-C), 101.22 (11-C), 99.28, 96.75, 86.17, 83.63, 76.88 (1'-C), 75.98 (2'-C), 66.63 (4'-C), 27.08 (3'-C);

^{19}F{^{1}H} NMR (376 MHz, $CDCl_3$): *δ* (ppm) = -134.10 – -135.15 (m, 2F), -136.31 – -136.87 (m, 2F), -150.36 (tt, *J* = 20.9, 2.4 Hz, 1F), -155.66 – -156.33 (m, 2F), -160.76 – -161.37 (m, 2F);

HR-MS (ESI+): *m/z* calc. ($C_{25}H_{13}F_9O_3Na$, $[M+Na]^+$): 555.06187, found: 555.06280.

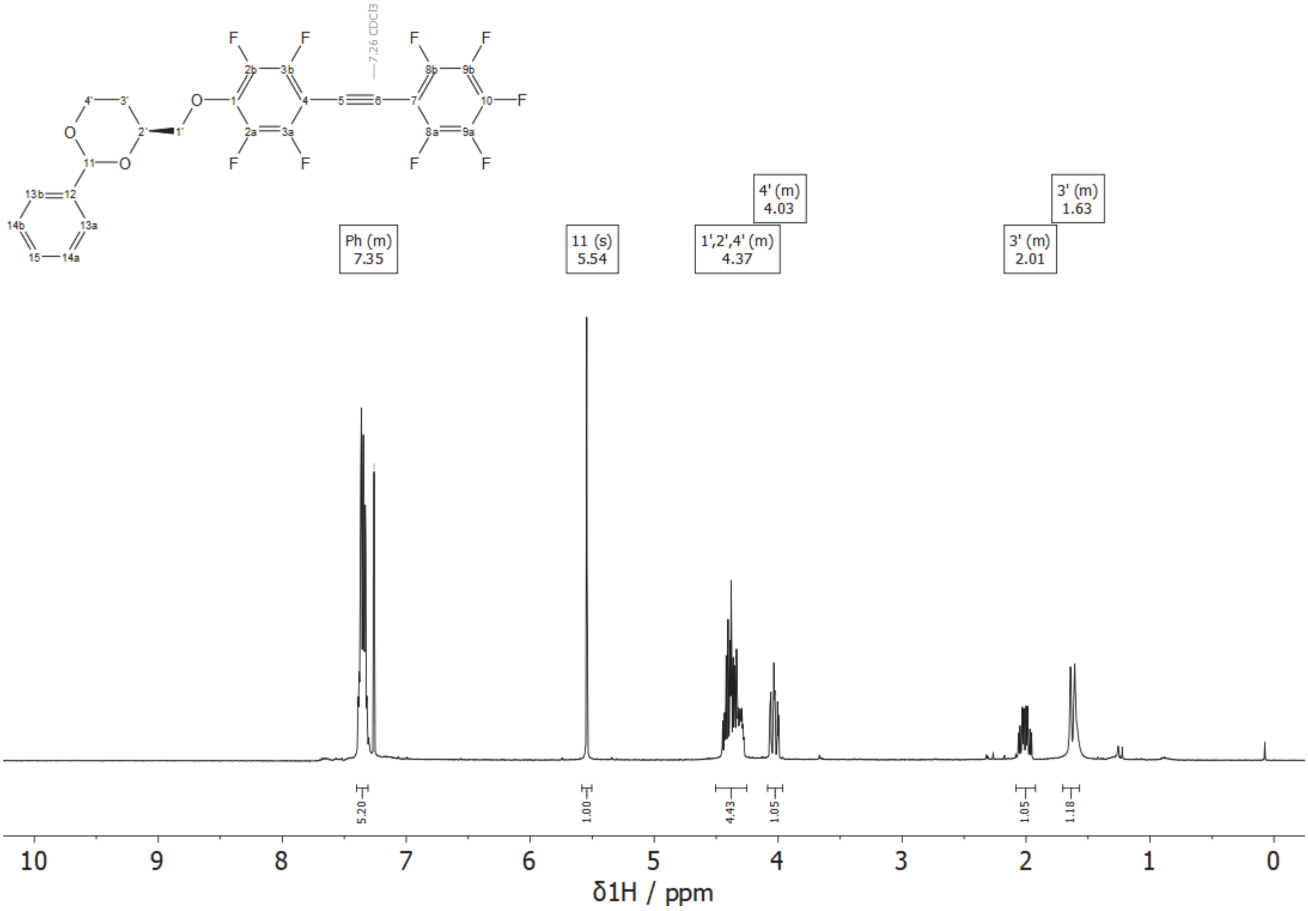

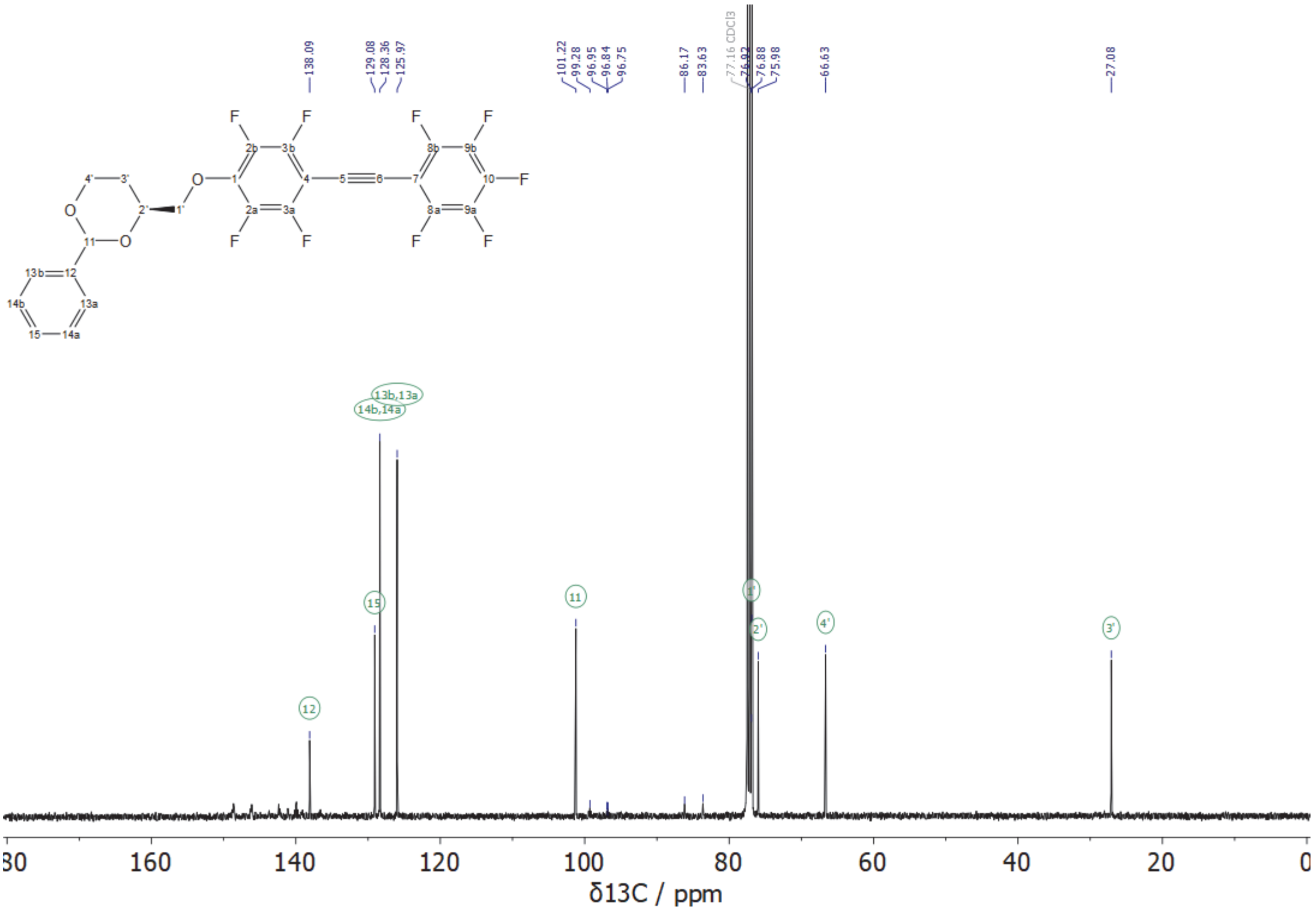
138.09
129.08
128.36
125.97
101.22
99.28
96.95
96.84
96.75
86.17
83.63
77.16 CDCl3
76.92
76.88
75.98
66.63
27.08
13b,13a
14b,14a
12
15
11
1'
2'
4'
3'
δ13C / ppm

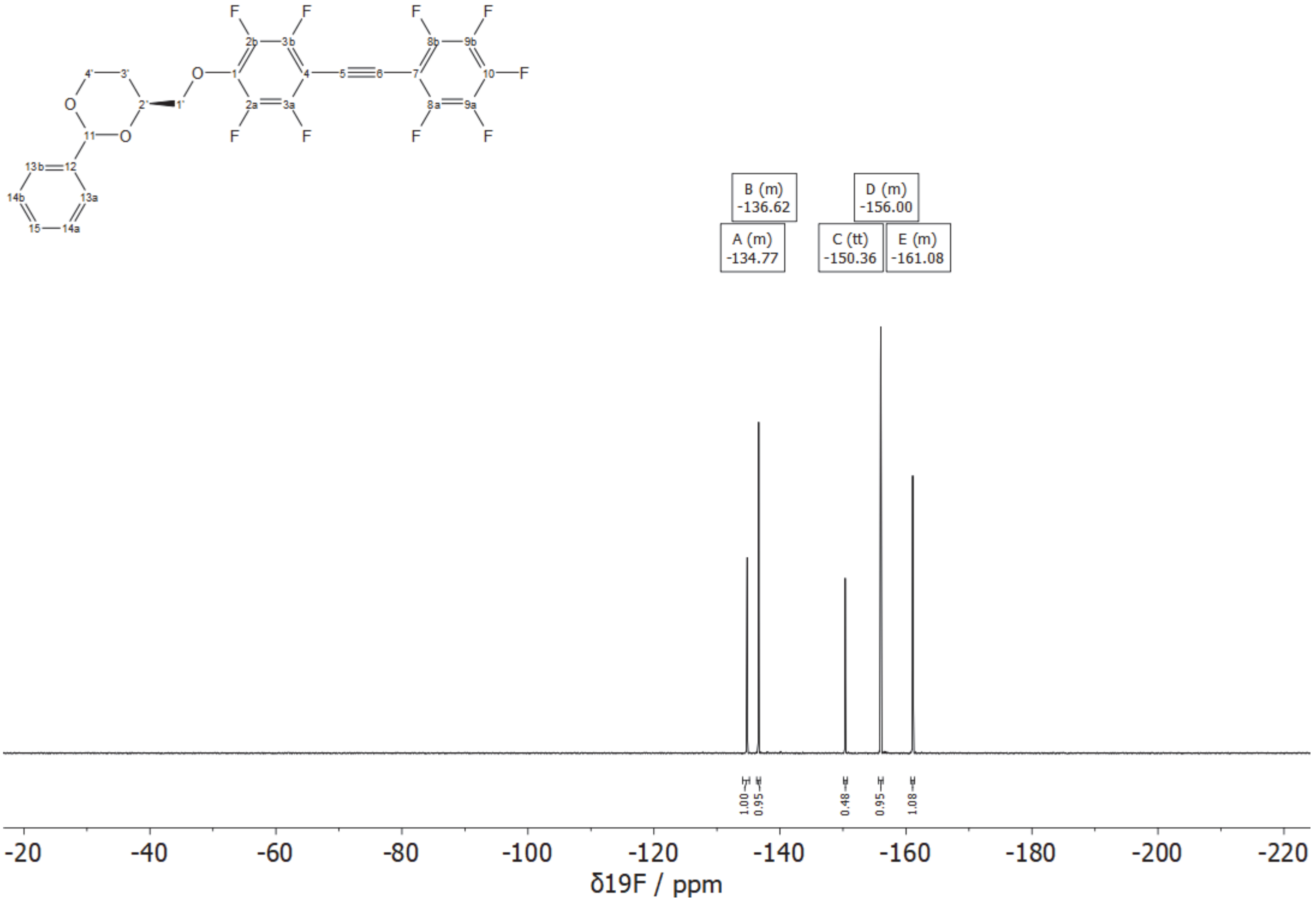
B (m)
-136.62
D (m)
-156.00
A (m)
-134.77
C (tt)
-150.36
E (m)
-161.08
1.00
0.95
0.48
0.95
1.08
δ19F / ppm

Compound **S18**

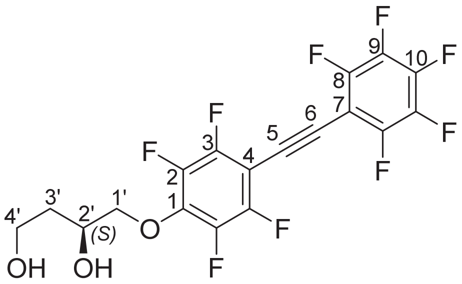

Compound **S17** (455 mg, 854 µmol, 1.00 eq.) was dissolved in THF (8.6 mL). Then 2 M hydrochloric acid (8.6 mL) was added and the reaction mixture was stirred for 5 d at room temperature. The mixture was diluted with water (20 mL) and extracted with EtOAc (3x30 mL). The combined organic phases were washed with brine (20 mL). The solvent was removed under reduced pressure and the residue was purified by column chromatography (hexane/EtOAc 1:1 + 1% Et_3N) to afford compound **S18** as a colorless solid (164 mg, 369 µmol, 43%). Compound **S17** (208 mg. 391 µmol, 46%) could be recovered for further reactions.

^{1}H NMR (400 MHz, $CDCl_3$): δ (ppm) = 4.37 – 4.18 (m, 3H, 1'-H, 2'-H), 3.99 – 3.87 (m, 2H, 4'-H), 2.97 (s_{br}, 1H, OH), 2.08 (s_{br}, 1H, OH), 1.90 – 1.75 (m, 2H, 3'-H);

^{13}C{^{1}H} NMR (100 MHz, $CDCl_3$): δ (ppm) = 79.11 (t, J = 3.3 Hz, 1'C), 70.25 (2'-C), 60.95 (4'-C), 34.21 (3'-C);

^{19}F{^{1}H} NMR (376 MHz, $CDCl_3$): (ppm) = -134.45 – -135.10 (m, 2F), -135.77 – -136.55 (m, 2F), -150.19 (tt, J = 20.8, 2.5 Hz, 1F), -156.11 – -156.67 (m, 2F), -160.73 – -161.59 (m, 2F);

HR-MS (ESI+): m/z calc. ($C_{18}H_9F_9O_3Na$, $[M+Na]^+$): 467.03002, found: 467.03038.

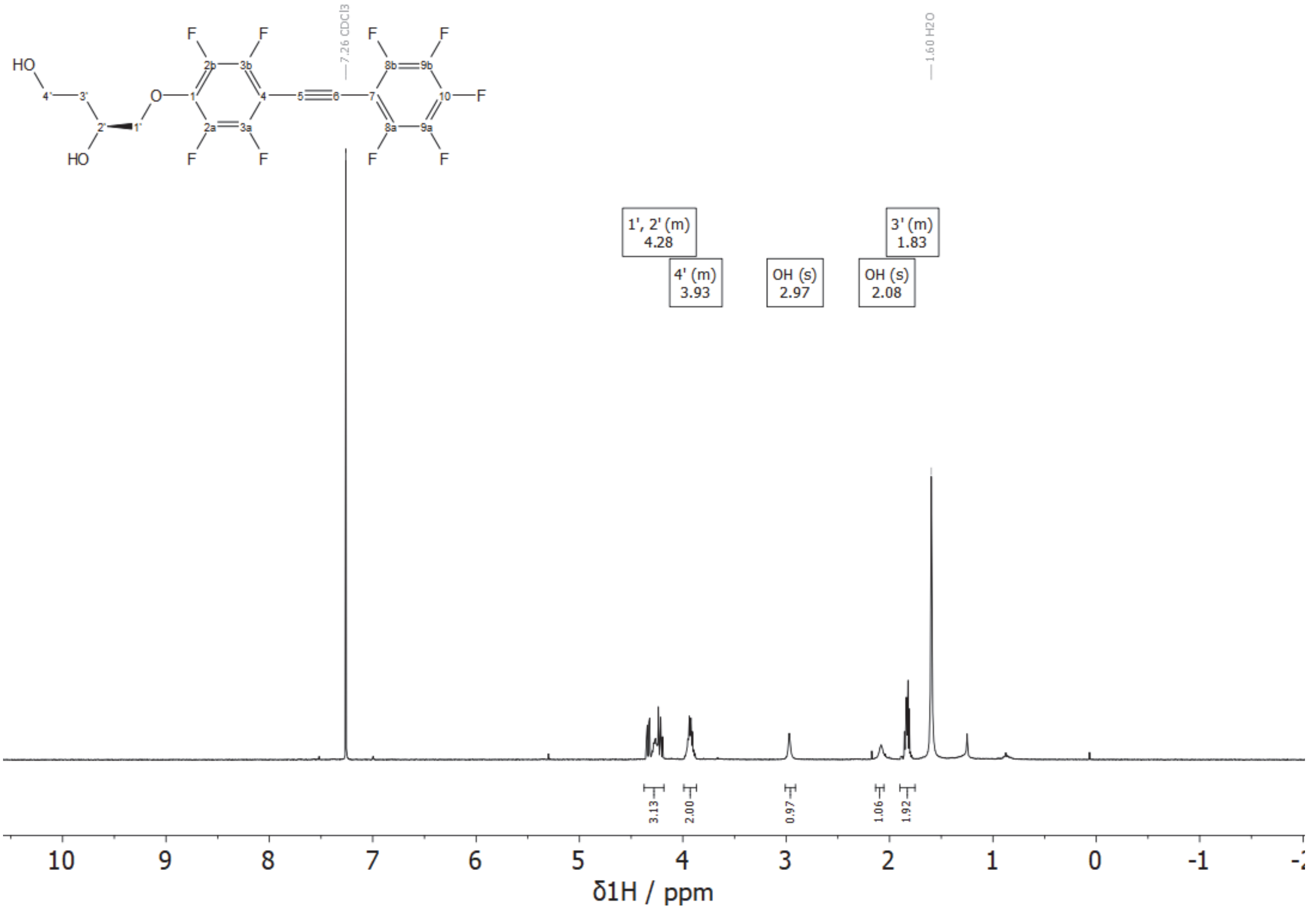

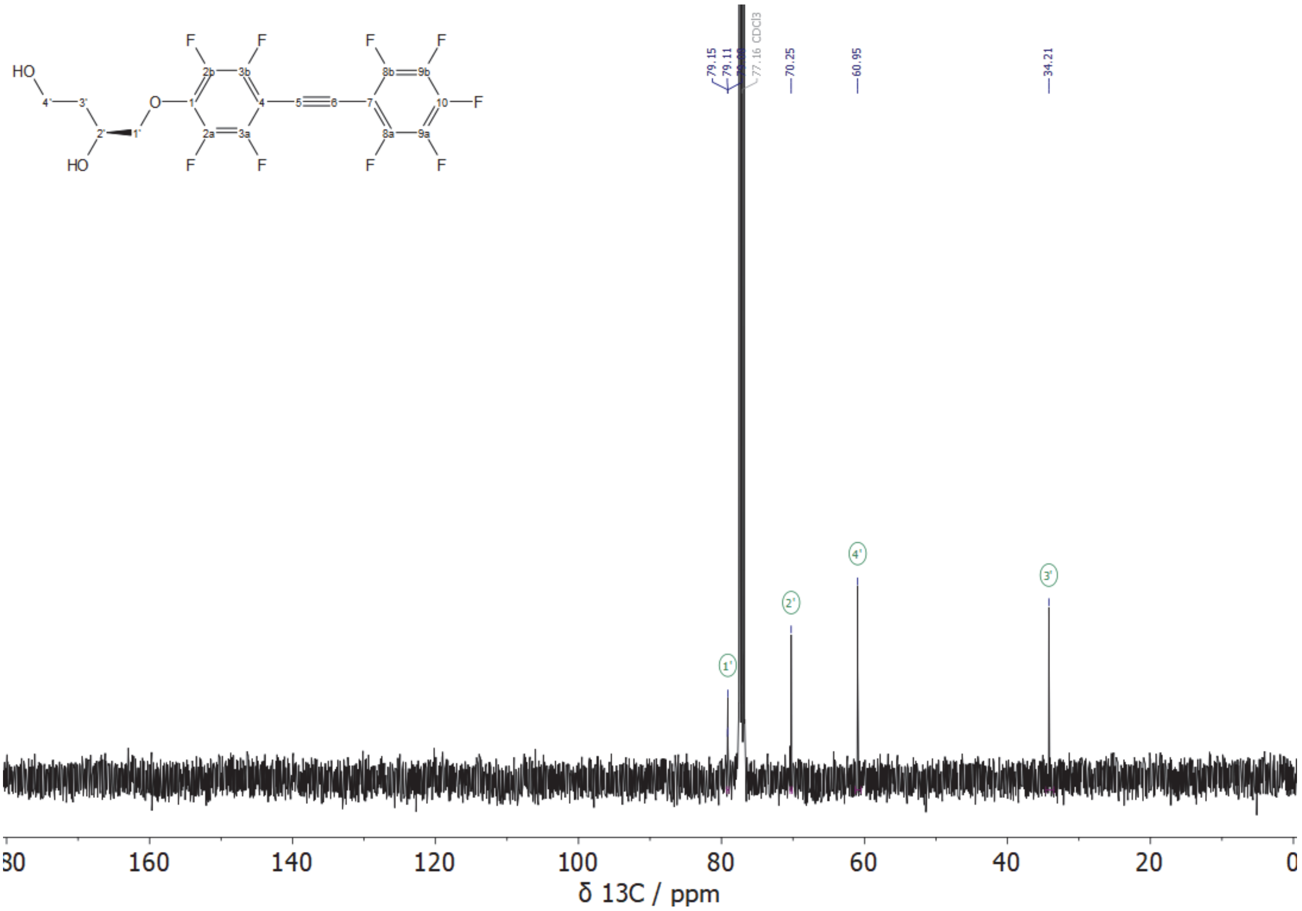
79.15
79.11
77.16 CDCl3
70.25
60.95
34.21
1'
2'
4'
3'
δ 13C / ppm

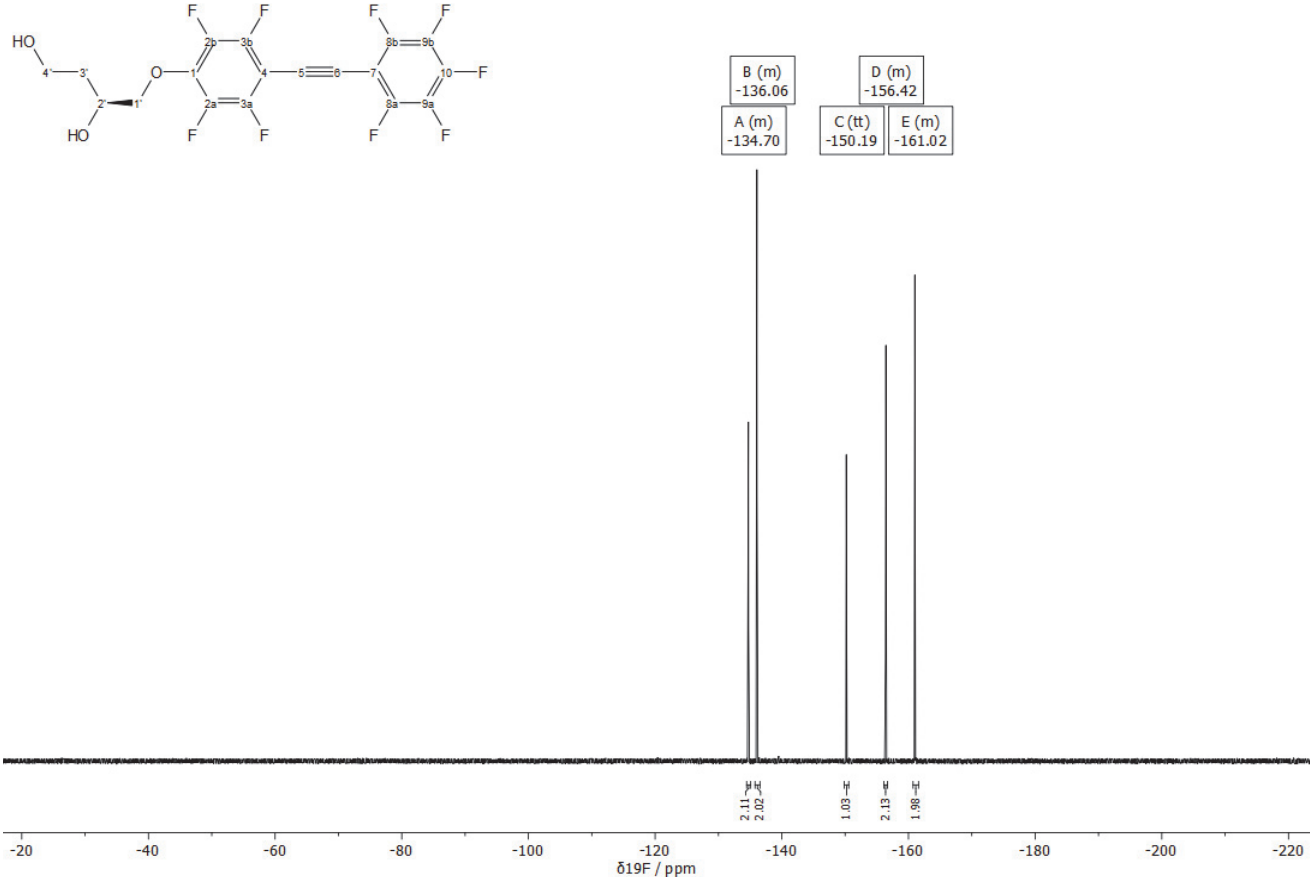
B (m)
-136.06
A (m)
-134.70
D (m)
-156.42
C (tt)
-150.19
E (m)
-161.02
2.11
2.02
1.03
2.13
1.98
δ19F / ppm

Compound **S19**

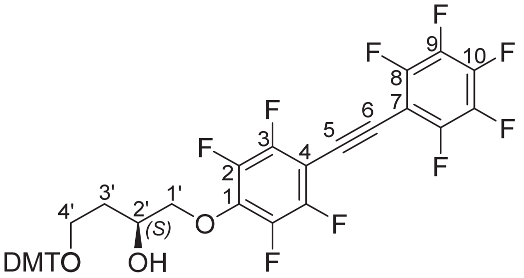

Under nitrogen atmosphere compound **S18** (160 mg, 361 µmol, 1.00 eq.) was dissolved in anhydrous pyridine (15 mL). 4,4'-dimethoxytrityl chloride (147 mg, 433 µmol, 1.20 eq.) was added in small portions over a time period of 10 min and the reaction mixture was stirred at ambient temperature for 23 h. The solvent was removed under reduced pressure and the residue was purified by column chromatography (hexane/EtOAc 7:1–6:1 +1% Et_3N) to afford compound **S19** as a colorless foam (203 mg, 272 µmol, 75%).

^{1}H NMR (400 MHz, $CDCl_3$): *δ* (ppm) = 7.43 – 7.39 (m, 2H, DMT-H), 7.34 – 7.27 (m, 6H, DMT-H), 7.24 – 7.19 (m, 1H, DMT-H), 6.86 – 6.81 (m, 4H, DMT-H), 4.31 – 4.12 (m, 3H, 1'-H, 2'-H), 3.79 (s, 6H, DMT-H), 3.45 – 3.25 (m, 2H, 4'-H), 3.09 (d, *J* = 3.2 Hz, 1H, 2'-OH), 1.98 – 1.81 (m, 2H, 3'-OH);

^{13}C{^{1}H} NMR (100 MHz, $CDCl_3$): *δ* (ppm) = 158.67 (DMT-C), 144.78 (DMT-C), 136.00 (DMT-C), 135.92 (DMT-C), 130.06 (DMT-C), 128.10 (DMT-C), 128.08 (DMT-C), 127.02 (DMT-C), 113.32 (DMT-C), 86.90 (DMT-C), 78.69 (t, *J* = 3.4 Hz, 1'-C), 69.83 (2'-C), 61.22 (4'-C), 55.35 (DMT-C), 32.74 (3'-C);

^{19}F{^{1}H} NMR (376 MHz, $CDCl_3$): *δ* (ppm) = -134.69 – -134.83 (m, 2F), -135.86 – -136.94 (m, 2F), -150.30 (tt, *J* = 20.7, 2.3 Hz, 1F), -155.72 – -157.26 (m, 2F), -160.63 – -161.59 (m, 2F);

HR-MS (ESI+): *m/z* calc. ($C_{39}H_{27}F_9NaO_5$, $[M+Na]^+$): 769.16070, found: 769.16267.

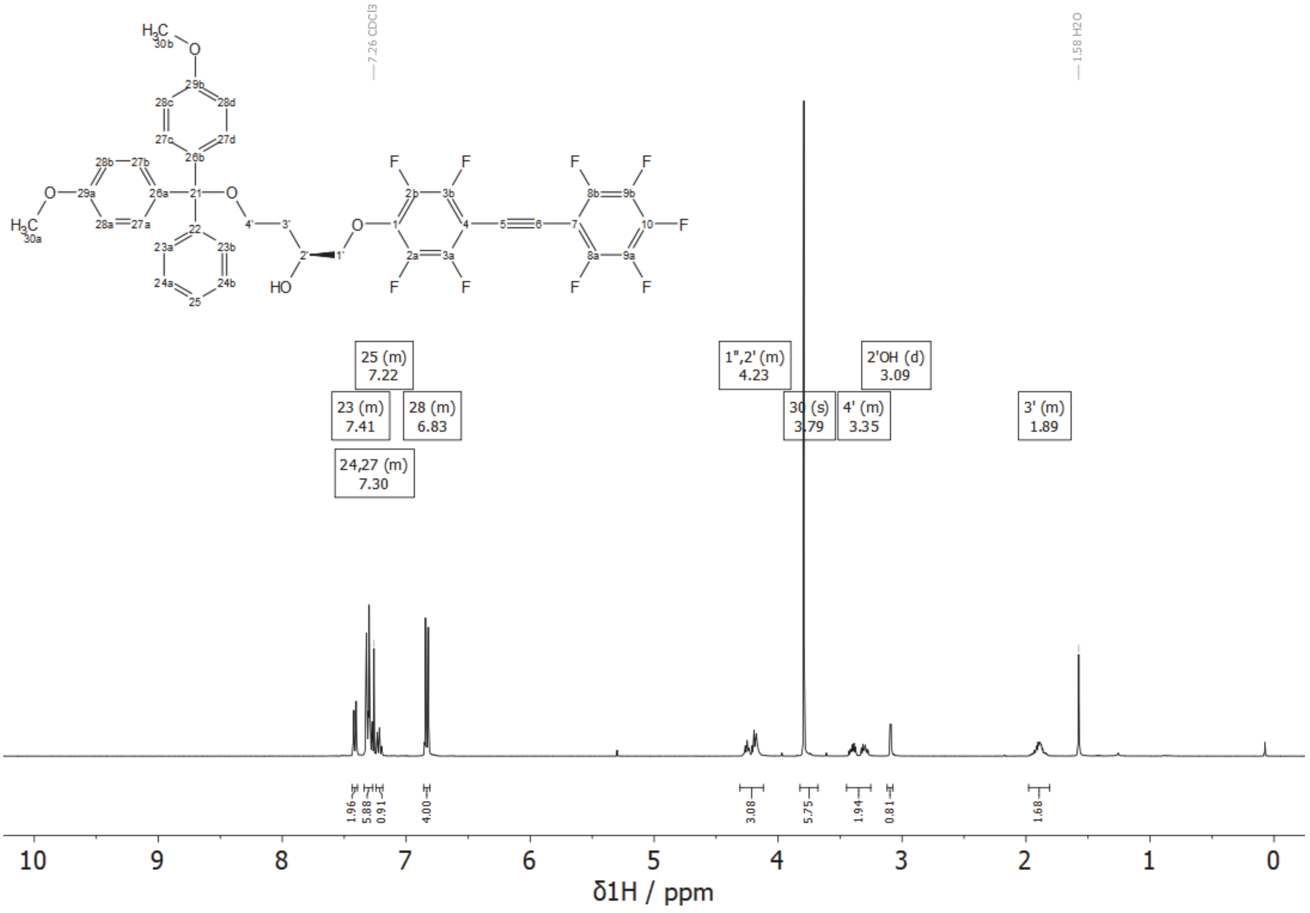

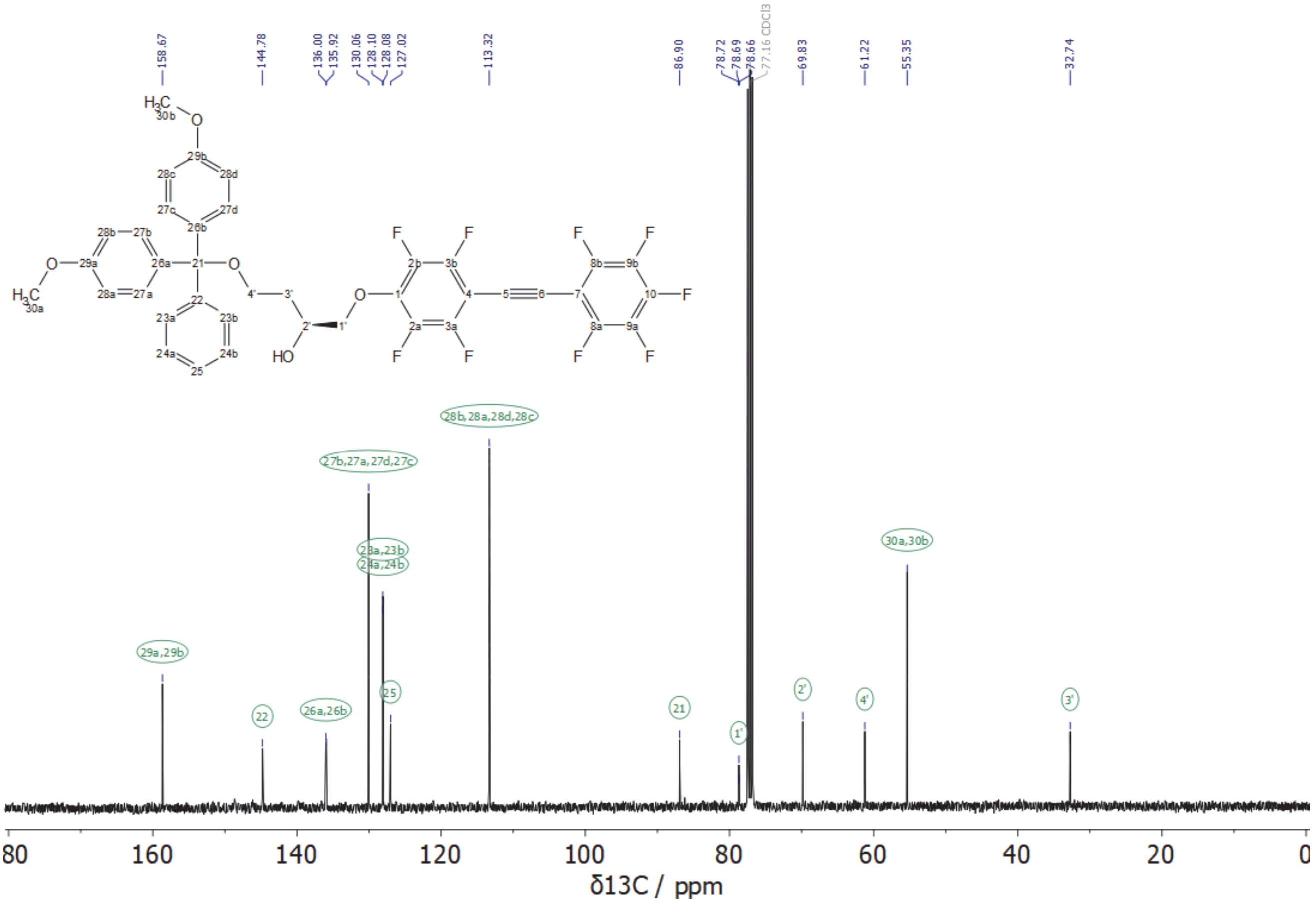
158.67
144.78
136.00
135.92
130.06
128.10
128.08
127.02
113.32
86.90
78.72
78.69
78.66
77.16 CDCl3
69.83
61.22
55.35
32.74
δ13C / ppm

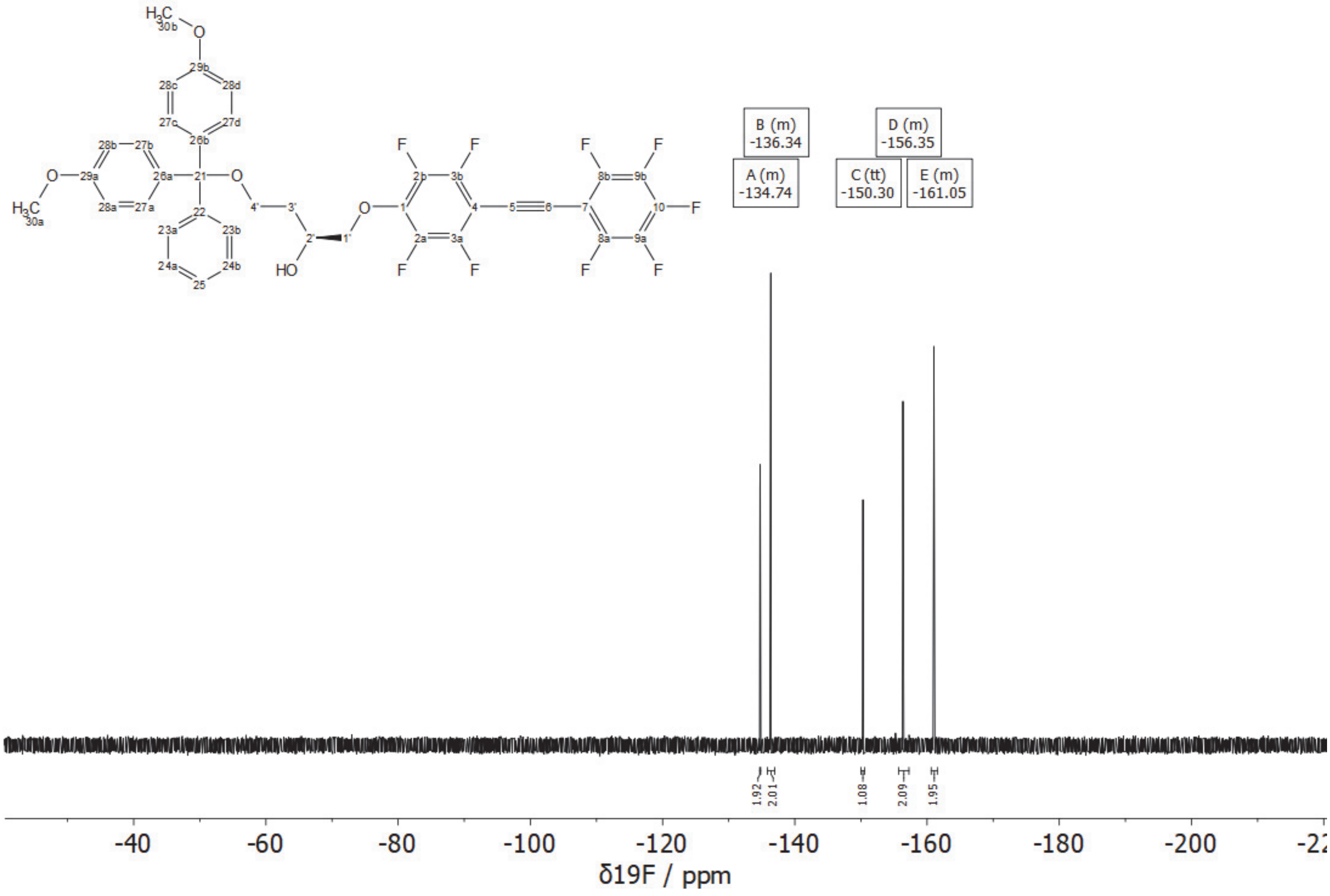
B (m)
-136.34
A (m)
-134.74
D (m)
-156.35
C (tt)
-150.30
E (m)
-161.05
δ19F / ppm

Compound **S20** = BTFF-PA

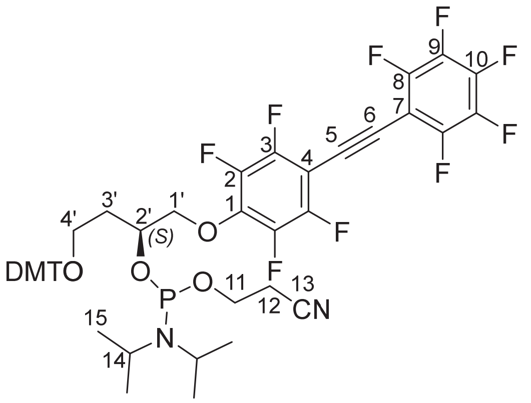

Under nitrogen atmosphere, compound **S19** (148 mg, 198 µmol, 1.00 eq.) was dissolved with DIPEA (202 µL, 153 mg 1.19 mmol, 6.00 eq.) in anhydrous DCM (6 mL). After 10 min CEP-Cl (60.8 mg, 257 µmol, 1.30 eq.) was added. The reaction mixture was stirred at ambient temperature for 2 h. The solvent was removed under reduced pressure and the residue was purified by column chromatography (hexane/EtOAc 7:1 + 1% Et_3N) to afford compound **S20** as a colorless foam (156 mg, 165 µmol, 83%).

^{1}H NMR (400 MHz, $CDCl_3$): *δ* (ppm) = 7.52 – 7.37 (m, 4H, DMT-H), 7.34 – 7.22 (m, 12H, DMT-H), 7.24 – 7.17 (m, 2H, DMT-H), 6.84 – 6.78 (m, 8H, DMT-H), 4.53 – 4.42 (m, 1H, 1'-H), 4.39 – 4.24 (m, 5H, 1'-H, 2'-H), 3.87 – 3.70 (m, 13H, 11-H, DMT-H), 3.70 – 3.53 (m, 3H, 11-H), 3.56 – 3.28 (m, 4H, 14-H), 3.31 – 3.17 (m, 4H, 4'-H), 2.63 – 2.55 (m, 2H, 12-H), 2.51 – 2.42 (m, 2H, 12-H), 2.16 – 1.96 (m, 4H, 3'-H), 1.19 – 0.98 (m, 24H, 15-H);

^{13}C{^{1}H} NMR (100 MHz, $CDCl_3$): *δ* (ppm) = 158.54 (DMT-C), 158.53 (DMT-C), 145.19 (DMT-C), 145.14 (DMT-C), 136.45 (DMT-C), 136.36 (DMT-C), 136.34 (DMT-C), 130.16 (DMT-C), 130.13 (DMT-C), 130.11 (DMT-C), 130.09 (DMT-C), 128.27 (DMT-C), 128.19 (DMT-C), 127.91 (DMT-C), 127.90 (DMT-C), 126.86 (DMT-C), 126.83 (DMT-C), 117.73 (13-C), 113.16 (DMT-C), 113.13 (DMT-C), 86.30 (DMT-C), 86.28 (DMT-C), 71.80 (2'-C), 71.63 (2'-C), 70.75 (2'-C), 70.60 (2'-C), 59.83 (4'-C), 59.67 (4'-C), 58.46 (11-C), 58.27 (11-C), 58.15 (11-C), 57.96 (11-C), 55.34 (DMT-C), 55.32 (DMT-C), 43.31 (14-C), 43.21 (14-C), 43.19 (14-C), 43.09 (14-C), 33.27(3'-C), 33.22 (3'-C), 33.18 (3'-C), 24.82 (15-C), 24.75 (15-C), 24.68 (15-C), 24.62 (15-C), 24.55 (15-C), 24.47 (15-C), 24.46 (15-C), 24.38 (15-C), 20.38 (12-C), 20.31 (12-C);

^{19}F{^{1}H} NMR (376 MHz, $CDCl_3$): *δ* (ppm) = -134.64 – -135.02 (m, 4F), -136.41 – -137.10 (m, 4F), -150.17 – -150.76 (m, 2F), -155.51 – -156.18 (m, 4F), -160.87 – -161.52 (m, 4F);

^{31}P{^{1}H} NMR (162 MHz, $CDCl_3$): *δ* (ppm) = 149.16 (t, *J* = 2.0 Hz, 1P), 148.63 (t, *J* = 2.7 Hz, 1P);

HR-MS (ESI+): *m/z* calc. ($C_{48}H_{44}F_9N_2NaO_6P$, $[M+Na]^+$): 969.26855, found: 969.26957.

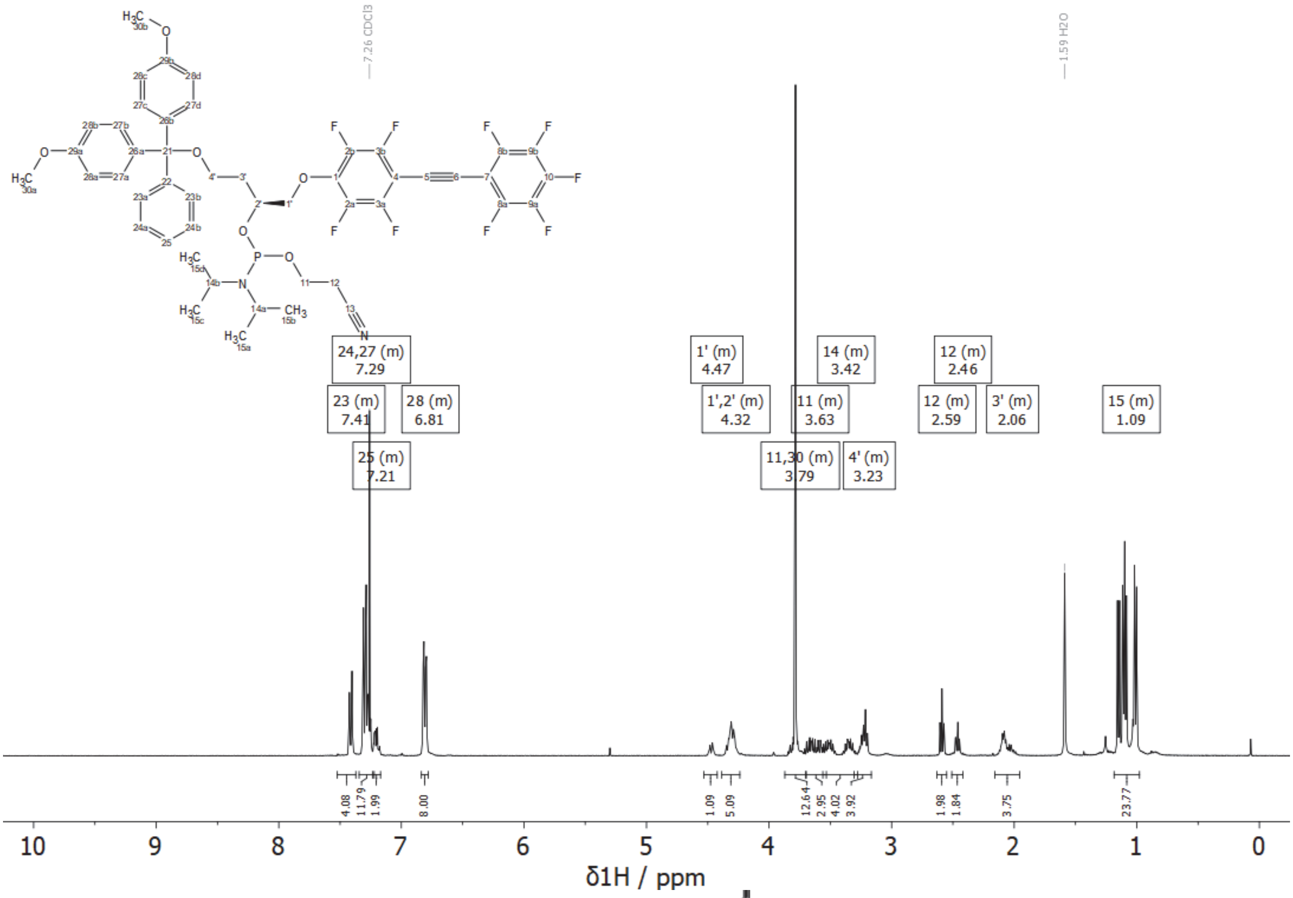

7.26 CDCl3
1.59 H2O
24,27 (m) 7.29
23 (m) 7.41
28 (m) 6.81
25 (m) 7.21
1' (m) 4.47
1',2' (m) 4.32
14 (m) 3.42
11 (m) 3.63
11,30 (m) 3.79
4' (m) 3.23
12 (m) 2.46
12 (m) 2.59
3' (m) 2.06
15 (m) 1.09
4.08
11.79
1.99
8.00
1.09
5.09
12.64
2.95
4.02
3.92
1.98
1.84
3.75
23.77
δ1H / ppm

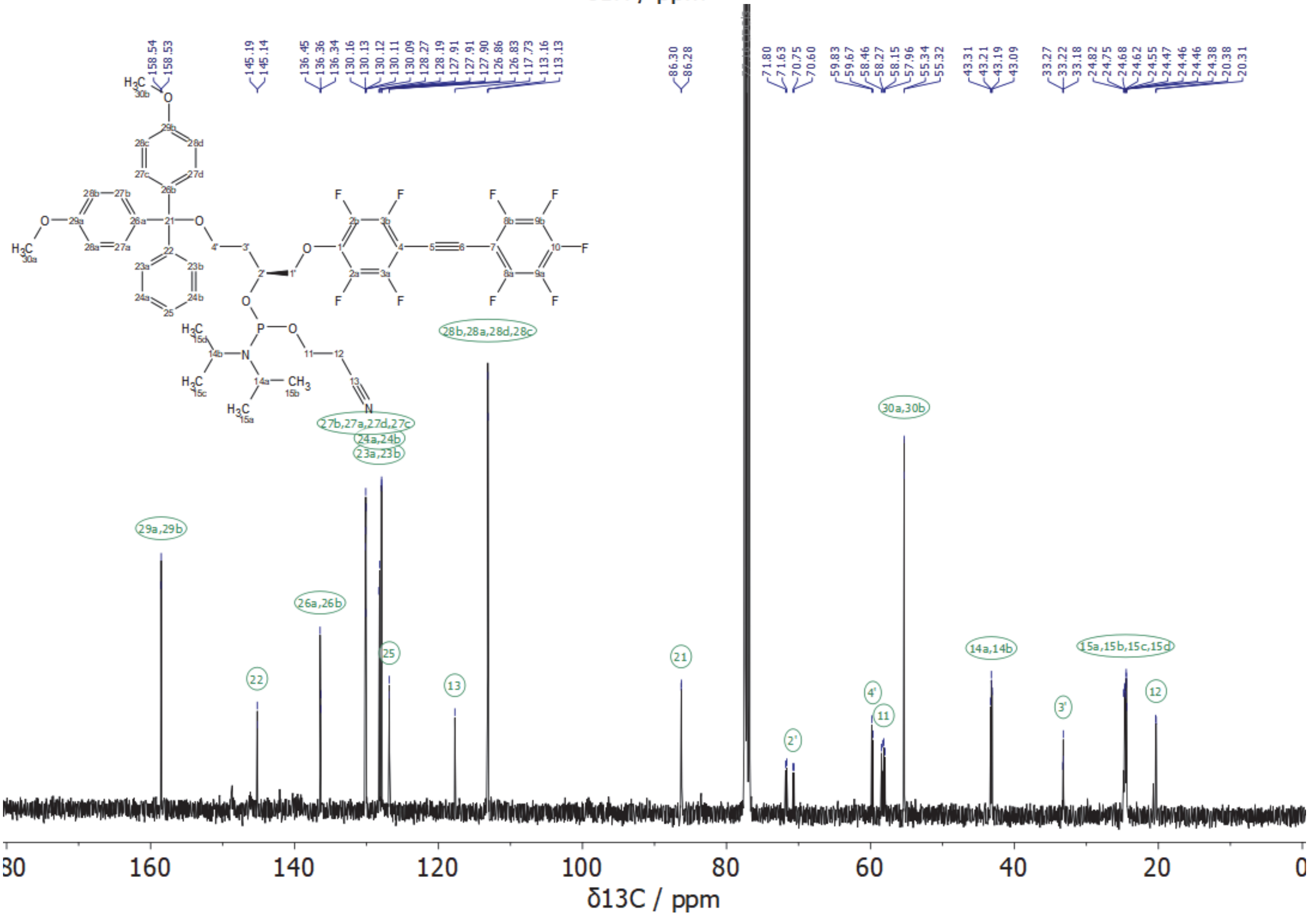

158.54
158.53
145.19
145.14
136.45
136.36
136.34
130.16
130.13
130.12
130.11
130.09
128.27
128.19
127.91
127.90
126.86
126.83
117.73
113.16
113.13
86.30
86.28
71.80
71.63
70.75
70.60
59.83
59.67
58.46
58.27
58.15
57.96
55.34
55.32
43.31
43.21
43.19
43.09
33.27
33.22
33.18
24.82
24.75
24.68
24.62
24.55
24.47
24.46
24.38
20.38
20.31
28b,28a,28d,28c
27b,27a,27d,27c
24a,24b
23a,23b
29a,29b
26a,26b
22
25
13
21
2'
4'
11
30a,30b
14a,14b
3'
15a,15b,15c,15d
12
δ13C / ppm

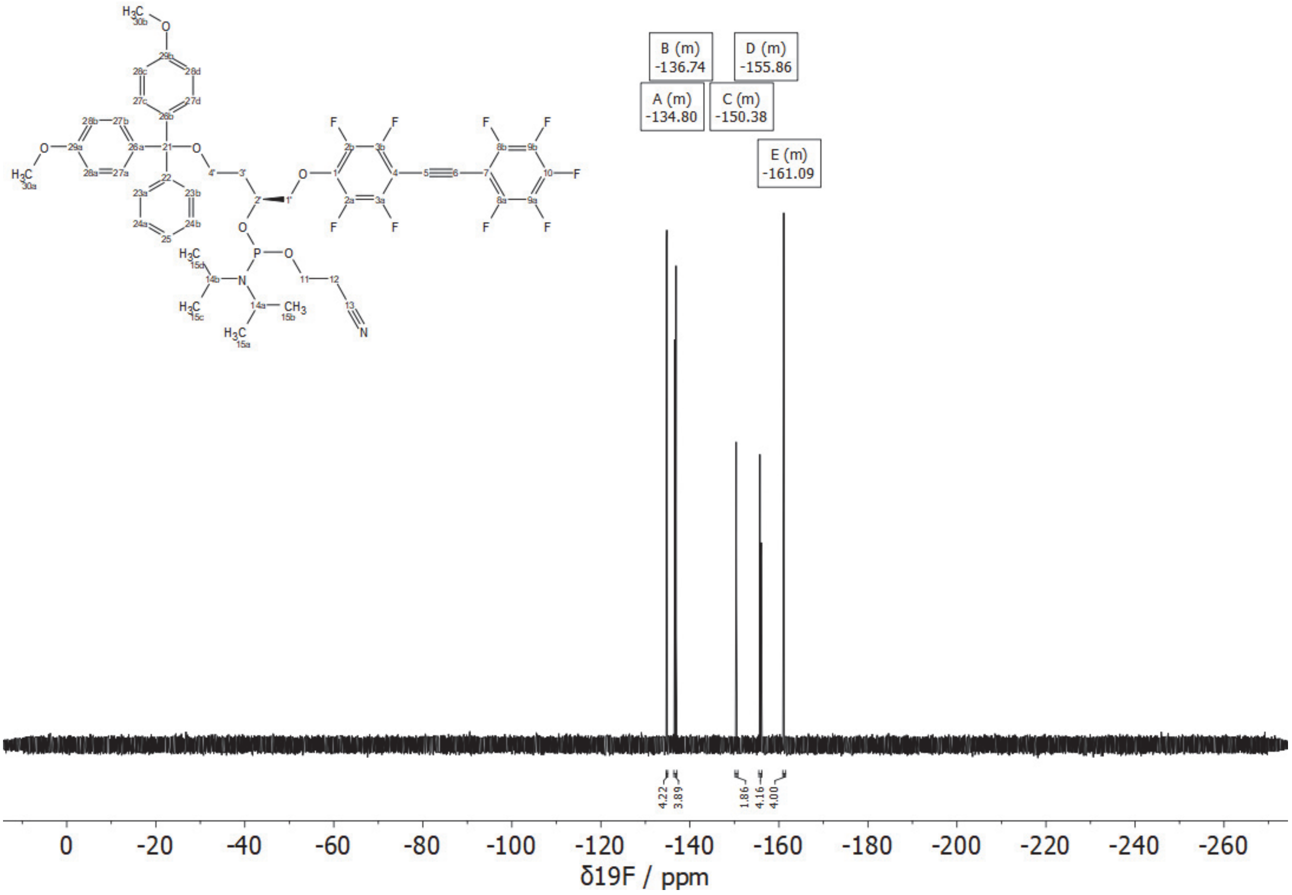
B (m)
-136.74
D (m)
-155.86
A (m)
-134.80
C (m)
-150.38
E (m)
-161.09
4.22
3.89
1.86
4.16
4.00
δ19F / ppm

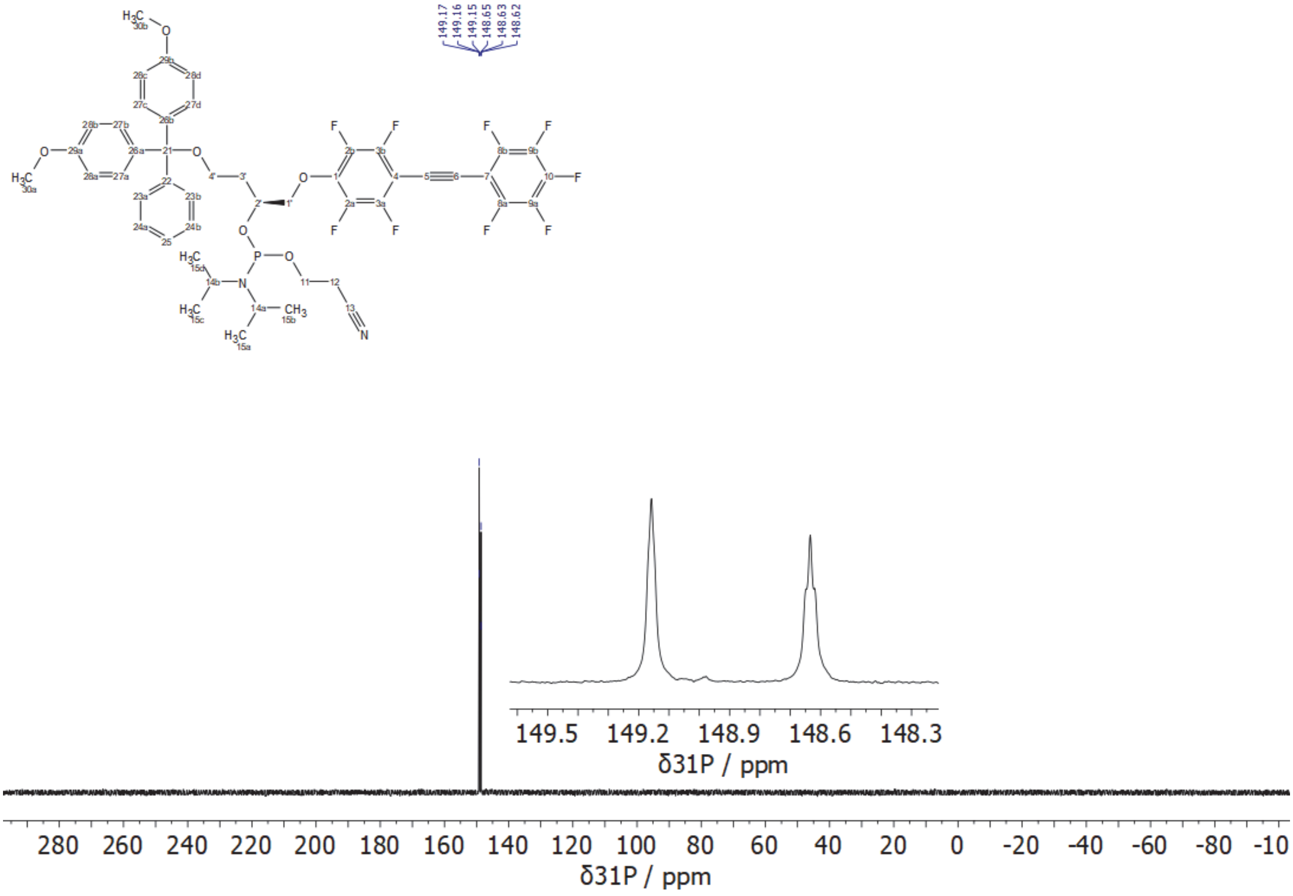
149.17
149.16
149.15
148.65
148.63
148.62
149.5
149.2
148.9
148.6
148.3
δ31P / ppm
δ31P / ppm

Oligonucleotide synthesis

Oligonucleotides were prepared on an Applied Biosystems ABI 392 DNA/RNA synthesizer on a 0.6 µmol scale using standard phosphoramidite chemistry. DMT-dC(ac)-CPG and DMT-dG(dmf)-CPG with a pore size of 1000 Å and loading density of 25 – 35 µmol/g were used as solid supports and were obtained from Sigma Aldrich. DMT-dA(bz), DMT-dC(ac), DMT-dG(dmf), DMT-dT were purchased from Chem Genes Corporation. Hex-5-yn-1-*O*-(2-cyanoethyl-*N*,*N*-diisopropyl)phosphoramidite[6] and 3-(4,4'-dimethoxytrityloxy)propyl-1-(2-cyanoethyl-*N*,*N*-diisopropyl)phosphoramidite[7] were synthesized as previously described. Solutions of standard phosphoramidites (70 mM) and synthesized phosphoramidites (80 mM) were prepared in anhydrous MeCN. Following solutions were used during synthesis:

Activator: 5 M ethylthiotetrazole (ETT) in anhydrous acetonitrile,
Oxidation: 20 mM iodine in THF/water/pyridine (66:12:22, *v*/*v*/*v*)
Cap A: pyridine/acetic anhydride/THF (10/10/80, *v*/*v*/*v*)
Cap B: NMI in THF (84/16, *v*/*v*).
Deprotection: 3% trichloro acetic acid in 1,2-dichloroethane

Cleavage from the solid support and removal of the base labile protecting groups were performed by treatment with concentrated ammonium hydroxide (33% NH_3) at 25°C overnight in a 1.5 mL screw-capped tube. The solid support was filtered off and the solvent was removed under reduced pressure. The residue was dissolved in water. The crude product was purified by denaturing PAGE. Gels (0.7x200x300 mm) were prepared using a 20% acrylamide solution containing 7 M urea in 1x TBE. After polymerization gels were run with 1x TBE buffer (89 mM Tris, 89 mM boric acid, 2 mM EDTA, pH 8.3)) for 2.5 h at a constant power (35 W). Product bands were visualized with a TLC plate under UV irradiation. The oligonucleotides were extracted with TEN buffer (10 mM Tris-HCl, 0.1 mM EDTA, 300 mM NaCl, pH 8.0) and recovered after precipitation with ethanol. DNA strands containing the TFF moiety needed an additional RP-HPLC purification step. Purification was performed on an ÄKTAmicro from GE Healthcare using a Nucleodur reversed-phase column (C18, 250x8 mm, 100 Å, 7 µm) from Machery Nagel at a flow rate of 2 mL min^{-1}. Linear gradients from 10–40% B over 50 min of buffer A (100 mM TEAA in water, pH 7.0) and buffer B (100 mM TEAA in MeCN/water 9:1, *v*/*v*) were used for purification. Chromatograms were monitored at 260 nm and 320 nm. All runs were performed at 40°C. HPLC purified oligos were lyophilized, dissolved in TEN buffer and precipitated with ethanol.

Purified oligonucleotides were analyzed by anion exchange HPLC using an ÄKTApurifier from GE Healthcare with a DNAPac PA 200 column (2x250 mm) from Thermo Scientific at a flow rate of 0.5 mL min^{-1}. Linear gradients from 0–48% B over 24 min of buffer A (25 mM Tris-HCl, 6 M urea, pH 8.0) and buffer B (25 mM Tris-HCl, 0.5 M $NaClO_4$, 6 M urea, pH 8.0) were used for analysis. Chromatograms were monitored at 260 nm. All analyses were performed at 40°C. High resolution ESI mass spectra were recorded on a Bruker micrOTOF-Q III spectrometer.

Labeling of 5'-alkyne functionalized oligonucleotides

5'-Alkyne functionalized oligonucleotides were fluorescently labeled using copper(I)-catalyzed alkyne-azide cycloaddition (CuAAC). Sulfo-Cy3-azide was obtained by Lumiprobe GmbH or Sulfo-Cy5-azide was purchased from Jena Bioscience GmbH. The freeze-dried DNA oligonucleotide (5 nmol) was dissolved in water (5 µL) and mixed with a DMSO/*t*BuOH mixture (3:1, 3 µL). A solution of azide (0.63 µL, 50 mM) in DMSO/*t*BuOH (3:1, v/v) was added. A freshly prepared solution of CuBr (0.63 µL, 100 mM) in DMSO/ *t*BuOH (3:1) was combined with a solution of tris(benzyltriazolylmethyl)amine (1.26 µL, 100 mM) in DMSO/ *t*BuOH (3:1) and then added to the reaction mixture. After 3 h of incubation in the dark at 37 °C, the reaction mixture was purified by PAGE (20% polyacrylamide).

UV/VIS spectroscopy / Thermal denaturing experiments

UV denaturing melting experiments were recorded on Varian Cary100 equipped with a 6x6 Multicell Block Peltier Series II cell changer and a VARIAN CARY Temperature Controller. Absorption was measured at 250, 260 and 280 nm. The absorption was recorded with a spectral bandwidth of 1 nm and the averaging time was set to 2s. The temperature cycle was programmed as follows:
20 °C to 90 °C
90 °C to 10 °C
10 °C to 90 °C
90 °C to 10 °C
10 °C to 90 °C
The first ramp was performed for annealing and was not considered for further melting temperature analysis. The heating rate was set to 0.5 °C min^{-1}. 500 µL sample in phosphate buffer (100 mM NaCl, 10 mM sodium phosphate, pH 7.0) with a duplex concentration of 1 µM, 2 µM and 5 µM was measured in 10 mm quartz cuvettes from VARIAN and 300µL sample in phosphate buffer (100 mM NaCl, 10 mM sodium phosphate, pH 7.0) with a duplex concentration of 10 µM and 20 µM were measured in 1 mm quartz cuvettes from HELLMA. The samples in the cuvettes were overlaid with silicone oil.

The obtained curves were fitted in a two-state transition model with upper and lower limit to obtain the melting temperature T_m. Absorption of the melted duplexes was used to recalculate $c_{total.}$ For the estimation of the thermodynamic parameters melting curves were analyzed according to Breslauer et al:[8]

$$\frac{1}{T_m} = \frac{(n-1)}{\Delta H^0}\ln c_{total} + \frac{\Delta S^0 - (n-1)\cdot R \cdot \ln 2n}{\Delta H^0} \quad (1)$$

Assuming a bimolecular association of two non-self-complementary strands leads to a molecularity = 2:

$$\frac{1}{T_m} = \frac{\ln c_{total}}{\Delta H^0} + \frac{\Delta S^0 - R\cdot \ln 4}{\Delta H^0} \quad (2)$$

Using van't Hoff analysis enthalpy ΔH^0 could be obtained from the slope and entropy ΔS^0 from the intercept of a linear fit of ln c_{total} vs $1/T_m$. Afterwards the free energy ΔG^{298} was calculated using the Gibbs-Helmholtz equation with T = 298 K:

$$\Delta G^{298} = \Delta H^0 - T\Delta S^0 \quad (3)$$

Calculation of double mutant cycle

The replacement with fluorinated tolanes leads to additional interactions with the surrounding DNA and the other tolane unit. The changes in free energy contributed to DNA/tolane interactions are defined as $\Delta\Delta G_{X7}$ for a substitution with a TFF at position X7, and $\Delta\Delta G_{Y18}$ for a substitution with a TFF at position Y18, respectively. $\Delta\Delta G_{het}$ is defined as the additionally energy gained by the introduction of a tolane heteropair. Starting from the THH homodimer the free energies of the other systems can be described as followed:

$$\Delta G_{FH} = \Delta G_{HH} + \Delta\Delta G_{X7} + \Delta\Delta G_{het} \quad (4)$$

$$\Delta G_{HF} = \Delta G_{HH} + \Delta\Delta G_{Y18} + \Delta\Delta G_{het} \quad (5)$$

$$\Delta G_{FF} = \Delta G_{HH} + \Delta\Delta G_{X7} + \Delta\Delta G_{het} \quad (6)$$

Solving the system of linear equations leads to following terms for $\Delta\Delta G_{X7}$, $\Delta\Delta G_{Y18}$, $\Delta\Delta G_{het}$:

$$\Delta\Delta G_{X7} = \frac{-\Delta G_{HH} + \Delta G_{FH} - \Delta G_{HF} + \Delta G_{FF}}{2} \quad (7)$$

$$\Delta\Delta G_{Y18} = \frac{-\Delta G_{HH} - \Delta G_{FH} + \Delta G_{HF} + \Delta G_{FF}}{2} \quad (8)$$

$$\Delta\Delta G_{het} = \frac{-\Delta G_{HH} + \Delta G_{FH} + \Delta G_{HF} - \Delta G_{FF}}{2} \quad (9)$$

Fluorescence spectroscopy

Fluorescence spectra and time-course measurements were recorded on a JASCO FP-8300 spectrofluorometer equipped with a FCT-817S cell changer and a F12 temperature control device from Julabo. All measurements were performed in a FMM-200 5 mm quartz fluorescence microcell from JASCO with a magnetic stir bar using a FMH-802 5 mm microcell jacket from JASCO as cell adapter. Emission spectra were recorded for Cy3 (excitation: 546 nm; emission: 561–750 nm) and Cy5 (excitation: 646 nm; emission: 661–750 nm) with following settings:

- Ex bandwidth: 5 nm
- Em bandwidth: 5 nm
- Response: 0.2 s
- PMT:voltage 480 V
- Data interval: 0.5 nm
- Scan speed: 500 nm/min

Time-course measurements wererecorded with following settings:

- Ex wavelength: 546 nm
- Em wavelength: 565 nm
- Ex bandwidth: 5 nm
- Em bandwidth: 5 nm
- Response: 0.2 s
- PMT:voltage 480 V
- Data interval: 0.2 s

FRET exchange experiments

A solution of a Cy3 and Cy5 labelled DNA duplex (0.104 µM, 270 µL) strands was prepared in phosphate buffer (104 mM NaCl, 10.4 mM phosphate, pH7.0). The duplex was annealed by heating to 95°C for 1 min and cooling down to room temperature over 30 min. The sample was transferred into a cuvette and incubated at 33°C for 5 min. Emission spectra of Cy3 and Cy5 was measured. The starting

Cy3 (F0) emission was recorded for 1 min and the FRET exchange experiment was started by adding an unlabeled single strand (2.8 µM, 10 µL), which sequence is comparable to the Cy5 labeled single strand. The increase of Cy3 emission (*F*) was recorded for 30 min if the labeled duplex contains a THH homopair or 60 min for a TFF homopair. An oligo with a C_3-spacer instead of the tolane moiety was added in excess (240 µM, 10 µL) to obtain the maximal Cy3 fluorescence (F_{total}) after 60 min. Emission spectra of Cy3 was measured to check if the Cy5 emission completely vanished.

The displacement curves were normalized with following equation:

$$F_{\mathrm{norm}} = \frac{F - F_0}{F_{total} - F_0} \quad (10)$$

The resulted curve was fitted with a reaction kinetic first order to obtain the total strand displacement (F_{max}) and the displacement rate (k_{obs}):

$$F_{\mathrm{norm}} = F_{\mathrm{max}} \cdot (1 - e^{-x \cdot k_{\mathrm{obs}}}) + y_0 \quad (11)$$

To obtain the mean curve the first measurement point after the addition of the unlabeled single strand was set to zero.

NMR spectroscopy of DNA duplexes

After HPLC purification, all the oligonucleotides were precipitated overnight at -20°C with five volumes of $LiClO_4$ 2% w/v in acetone. The pellet was then solved in ddH_2O and lyophilized to remove residual acetone. The complementary oligonucleotides were annealed in the final volume of the NMR sample (180 µL) by heating to 95°C 5 minutes and slowly cooling down to room temperature overnight. The samples used for NMR assignments contained 0.2-1 mM DNA duplex and were dissolved in either 90% H_2O/10% D_2O or 99.95% D_2O containing NMR buffer (20 mM NaPi buffer at pH 7.0, 100 mM NaCl).
The samples used for CLEANEX-PM experiments contained 0.2 mM DNA duplex and were dissolved in 90% H_2O/10% D_2O containing 10 mM NaPi buffer (pH 7.0) and 100 mM NaCl.
The same buffer stock solution was used for all the samples.
3-(Trimethylsilyl)-1-propanesulfonic acid (DSS) was added to all the samples as internal reference for ^{1}H. ^{13}C, ^{19}F and ^{31}P were indirectly referenced from the ^{1}H chemical shift.[9]
Nearly complete assignment of ^{1}H, ^{13}C, ^{19}F and ^{31}P resonances was achieved by means of 2D homo- and heteronuclear NMR experiments and following established assignment strategies (i.e., imino-imino NOESY walk and sequential intra-strand H1'(n)-H8/6(n+1)-H1'(n+1) NOESY walk).
All the NMR experiments were performed on a Bruker Avance III 600 NMR spectrometer equipped with a DCH ^{13}C / ^{1}H cryoprobe, a Bruker Avance III 600 NMR spectrometer equipped with a BBFO room temperature probe or a Bruker Avance III HD 400 equipped with a BBFO room temperature probe.
The NMR spectra were acquired and processed using the software Topspin 3.2 (Bruker BioSpin, Germany). The spectra analysis was performed using Sparky 3.114[10] or NMRFAM-SPARKY.[11] Integrals of the NOESY cross peaks shown in Figure S18-19 were calculated using the integration module of Sparky 3.114 (integration method: Gaussian fit).
2D ^{1}H,^{1}H NOESY spectra recorded on the samples in 90% H_2O/10% D_2O used jump-return-echo (excitation maximum on the middle of the imino region)[12] or excitation sculpting with gradients[13] for water suppression and contained a 200 ms mixing time. 1D ^{19}F NMR and 2D ^{1}H,^{19}F HOESY spectra were recorded on the samples containing fluorinated tolane units in 90% H_2O/10% D_2O. The 2D ^{1}H,^{19}F HOESY used excitation sculpting for water suppression[13] and contained a mixing time of 200 ms. 1D ^{31}P spectra were recorded with power-gated decoupling.
^{1}H,^{13}C HSQC for aromatic region ($^{1}J_{CH}$ 200Hz) and for sugar region ($^{1}J_{CH}$ 160Hz), DQF-COSY[14], TOCSY[13, 15], ^{1}H,^{1}H NOESY[16], ^{1}H,^{31}P HSQC spectra ($^{1}J_{PH}$ 20 Hz) were recorded on the samples in 99.95% D_2O for assignment. NOESY and COSY data show that the sugar pucker of most deoxyribose residues is C2'-endo, except for some residues adjacent to the modification site and closing base pairs
Water hydrogen exchange rates of imino protons (k_{EX}) were measured using a 1D version of the CLEANEX-PM pulse sequence[17] according to the protocol described before.[18] A series of exchange experiments with different mixing times τ_m (5, 25, 50, 50, 100, 100, 150, 200, 300, 400, 400, 500 ms) were performed using the standard Bruker pulse program zgcxesgp, employing excitation sculpting with gradients as water suppression scheme.[13] The Bruker standard program zgesgp, employing as well excitation sculpting with gradients as water suppression scheme,[13] was used as reference experiment, without CLEANEX-PM element and water saturation pulse. 2560 scans were recorded for each experiment using an interscan delay of 2.0 s and 16 dummy scans. The spectra were processed using a line broadening factor of 10 Hz and the peak intensities were used for the data analysis.
The water ^{1}H longitudinal relaxation rate R_{1w} (s^{-1}) was determined for each sample using a saturation recovery experiment as described by Szulik et al.[19]
For each imino signal, the ratio between the intensity of peak in the CLEANEX-PM experiment (I) and the intensity of the peak in the reference experiment (I_0) was plotted as a function of the mixing time (τ_m). The imino proton water exchange rate k_{EX} (s^{-1}) and the apparent imino proton relaxation rate R_{1A} were obtained by fitting the resulting curve to the following equation:

$$\frac{I}{I_0} = \frac{k_{\mathrm{EX}}}{(R_{1\mathrm{A}} + k_{\mathrm{EX}} - R_{1\mathrm{W}})} \cdot (e^{-R_{1\mathrm{W}} \cdot \tau_{\mathrm{m}}} - e^{(-R_{1\mathrm{A}} + k_{\mathrm{EX}})\tau_{\mathrm{m}}}) \quad (12)$$

With:
I = intensity of the imino peak at the mixing time τ_m
I_0 = intensity of the imino peak in the reference experiment
k_{EX} = imino proton to water exchange rate (s^{-1})
τ_m = mixing time (s)
R_{1w} = water ^{1}H longitudinal relaxation rate (s^{-1})
R_{1A} = imino ^{1}H apparent relaxation rate (combination of longitudinal and transverse relaxation rate, s^{-1})

The fitting was performed with Mathematica 8.0. The error for the fitted parameter results from the fitting.

Computational methods

ORCA version 4.2.1[20] was used for the calculation of the DFT-optimized geometries and electrostatic potential maps. The acyclic backbone was replaced with a methyl group. B3LYP functional with D3BJ dispersion correction[21], a def2-TZVP basis set[22] and the corresponding auxiliary basis set for the RIJCOSX approximation[23] on all atoms was used for the calculation.

Electrostatic potential maps were generated from a cube file of the electron density with a resolution of 80×80×80 grid points using a python script that is available online.[24]

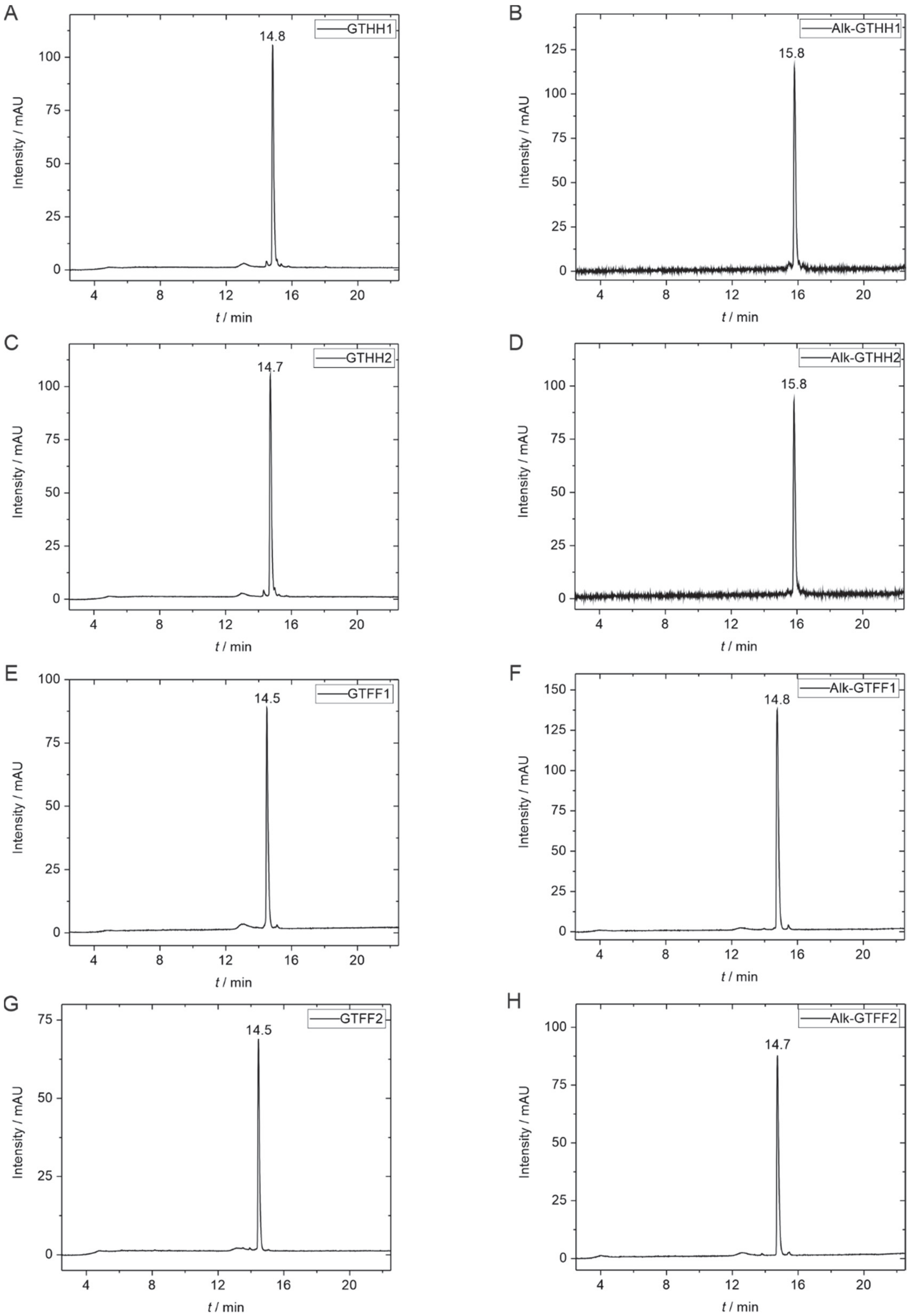

Figure S20 Analytical anion exchange HPLC chromatograms of the DNA oligonucleotides GTHH1 (A), Alk-GTHH1 (B), GTHH2 (C), Alk-GTHH2 (D), GTFF1 (E), Alk-GTFF1(F), GTFF2 (G) and Alk-GTFF2(H).

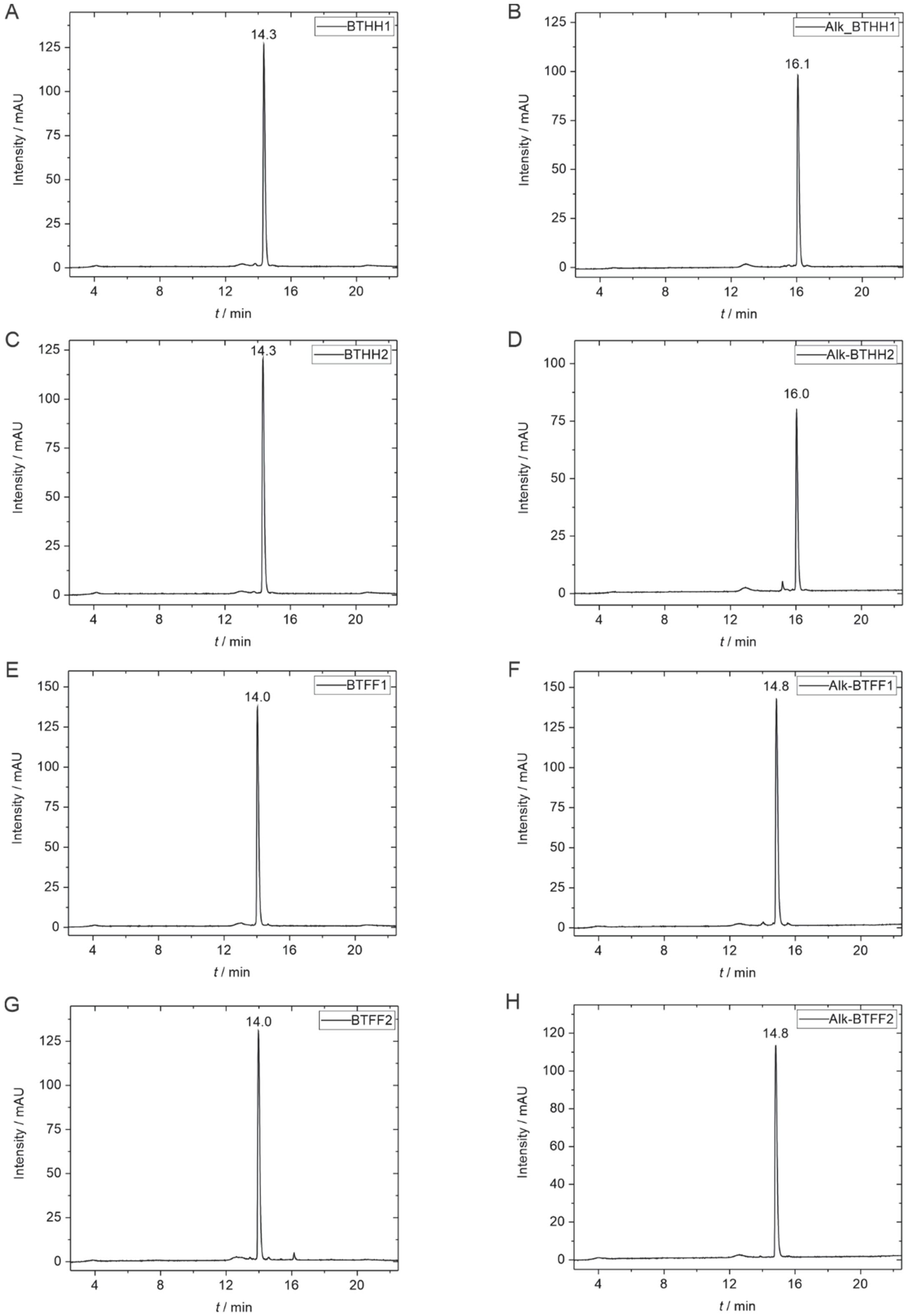

Figure 21 Analytical anion exchange HPLC chromatograms of the DNA oligonucleotides BTHH1 (A), Alk-BTHH1 (B), BTHH2 (C), Alk-BTHH2 (D), BTFF1 (E), Alk-BTFF1(F), BTFF2 (G) and Alk-BTFF2(H).

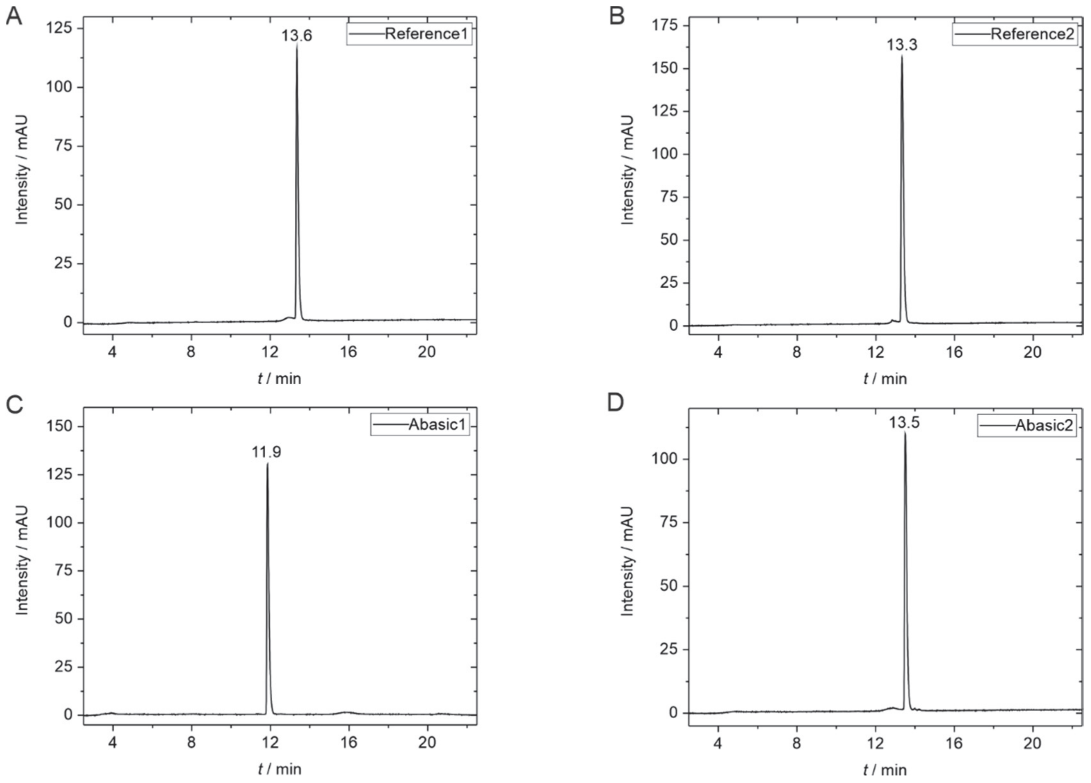

Figure S22 Analytical anion exchange HPLC chromatograms of the DNA oligonucleotides Reference1 (A), Reference2 (B), Abasic1 (C) and Abasic2 (D).

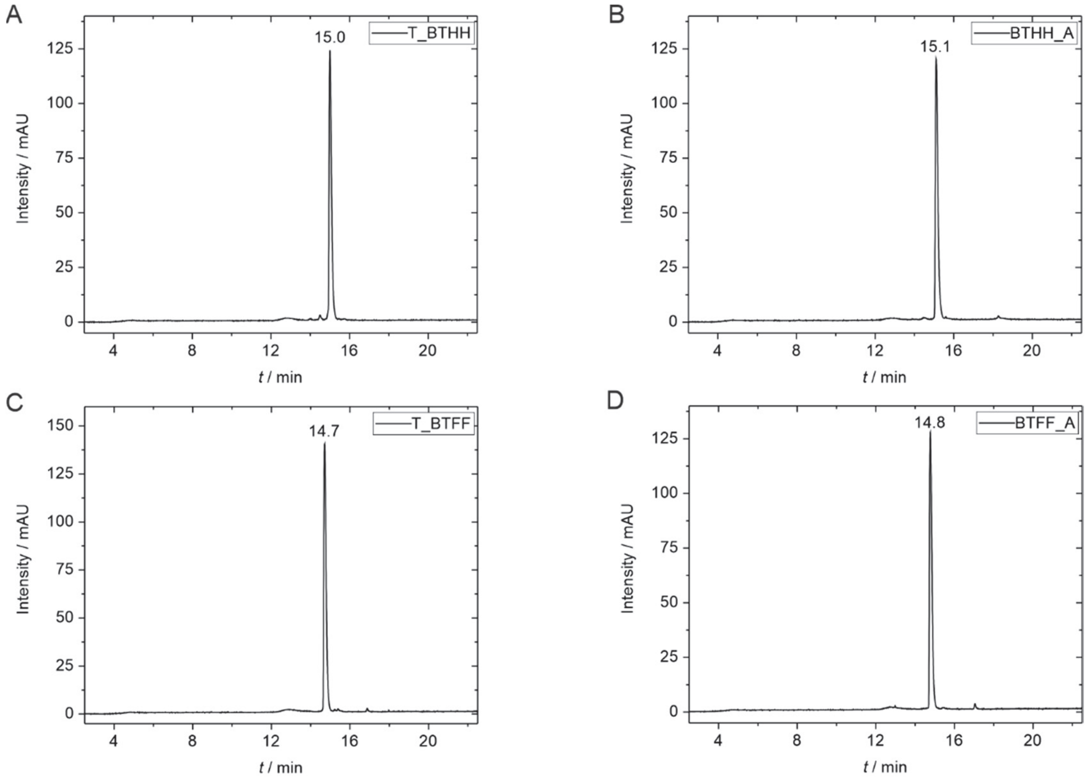

Figure S23 Analytical anion exchange HPLC chromatograms of the DNA oligonucleotides T_BTHH (A), BTHH_A (B), T_BTFF (C) and BTFF_A (D).

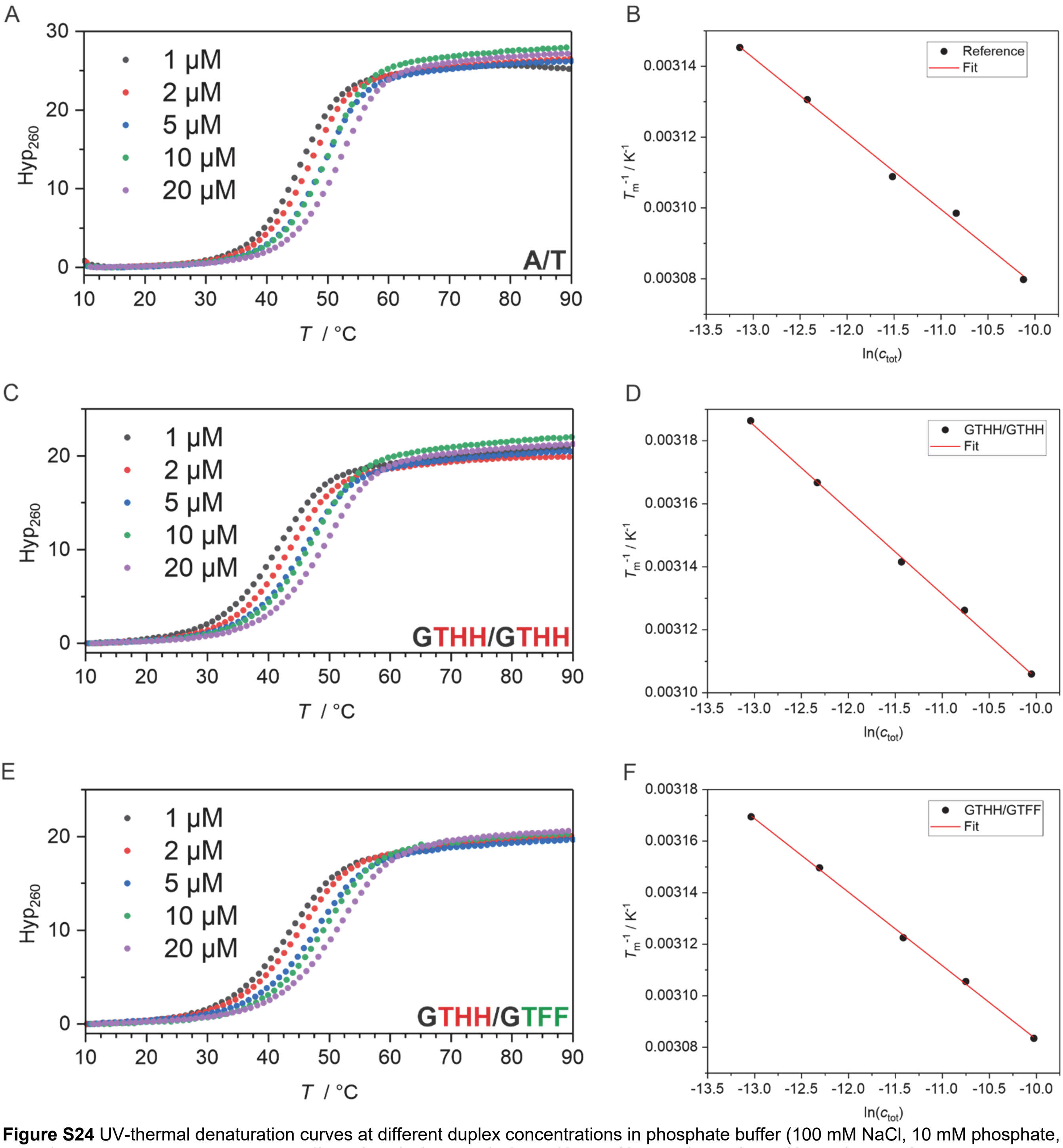

Figure S24 UV-thermal denaturation curves at different duplex concentrations in phosphate buffer (100 mM NaCl, 10 mM phosphate, pH7.0) and the corresponding van't Hoff plot for T/A (A and B), GTHH/GTHH (C and D) and GTHH/GTFF (E and F).

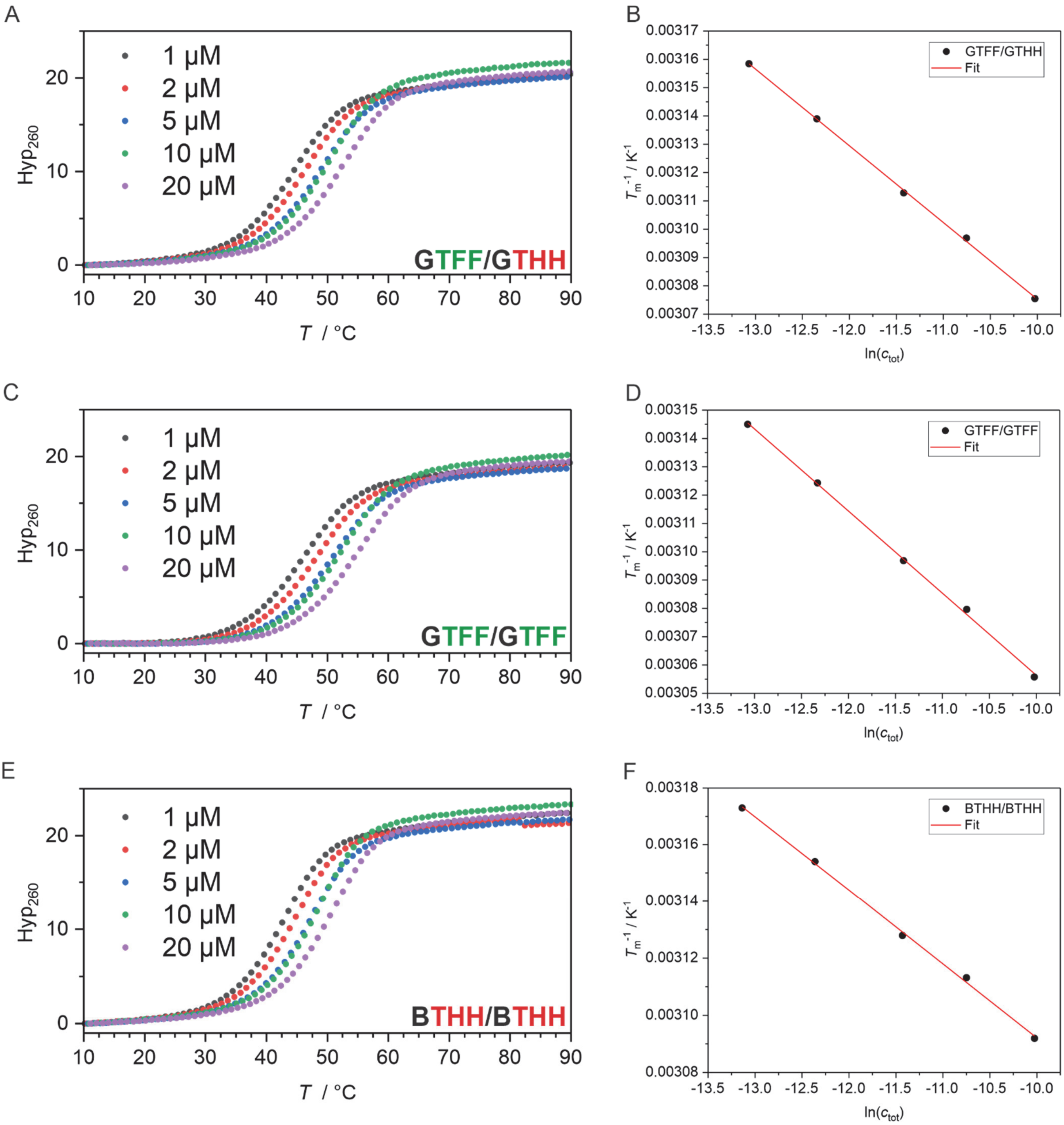

Figure S25 UV-thermal denaturation curves at different duplex concentrations in phosphate buffer (100 mM NaCl, 10 mM phosphate, pH7.0) and the corresponding van't Hoff plot for GTFF/GTHH (A and B), GTFF/GTFF (C and D) and BTHH/BTHH (E and F).

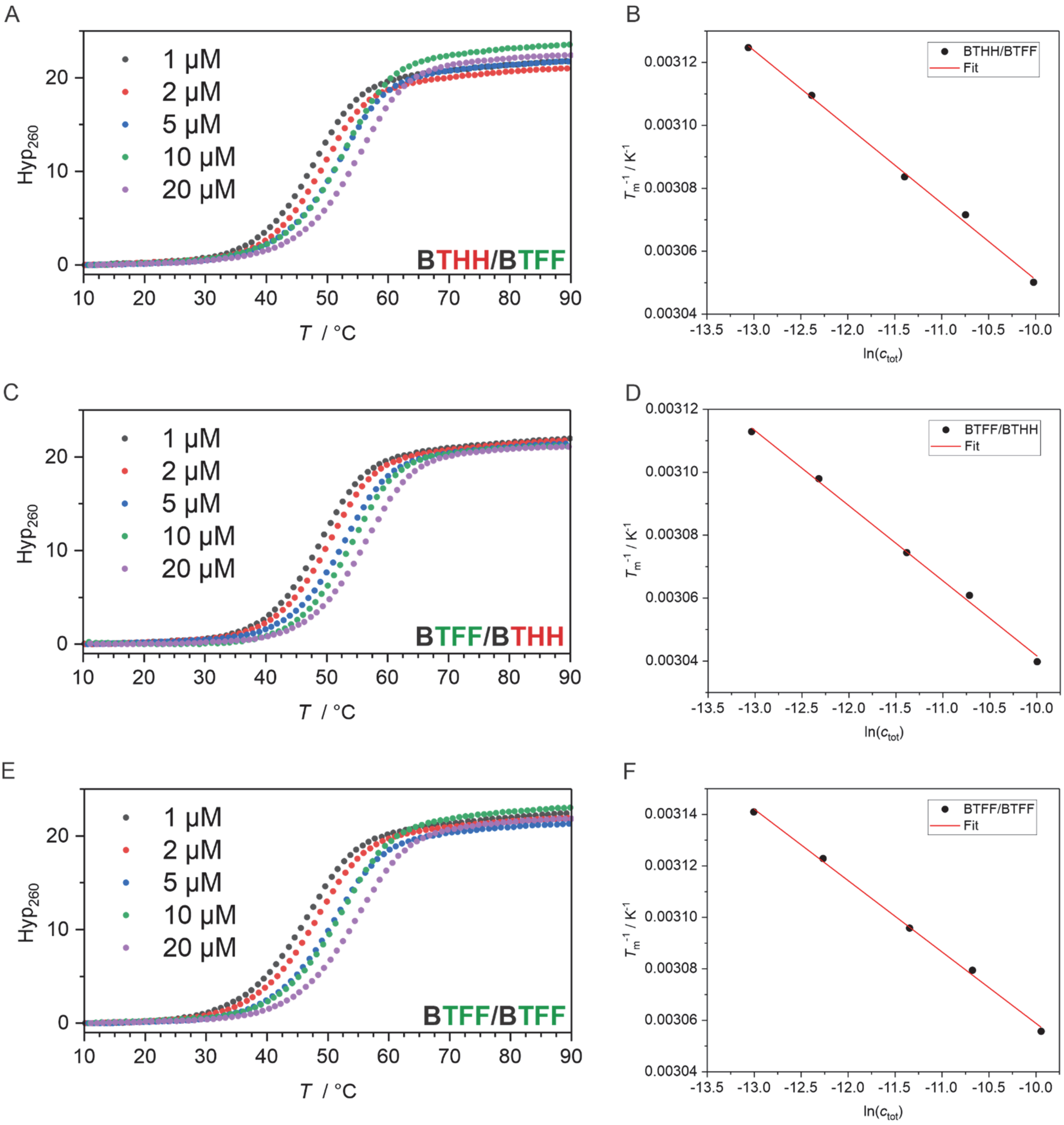

Figure S26 UV-thermal denaturation curves at different duplex concentrations in phosphate buffer (100 mM NaCl, 10 mM phosphate, pH7.0) and the corresponding van't Hoff plot for BTHH/BTFF (A and B), BTFF/BTHH (C and D) and BTFF/BTFF (E and F).

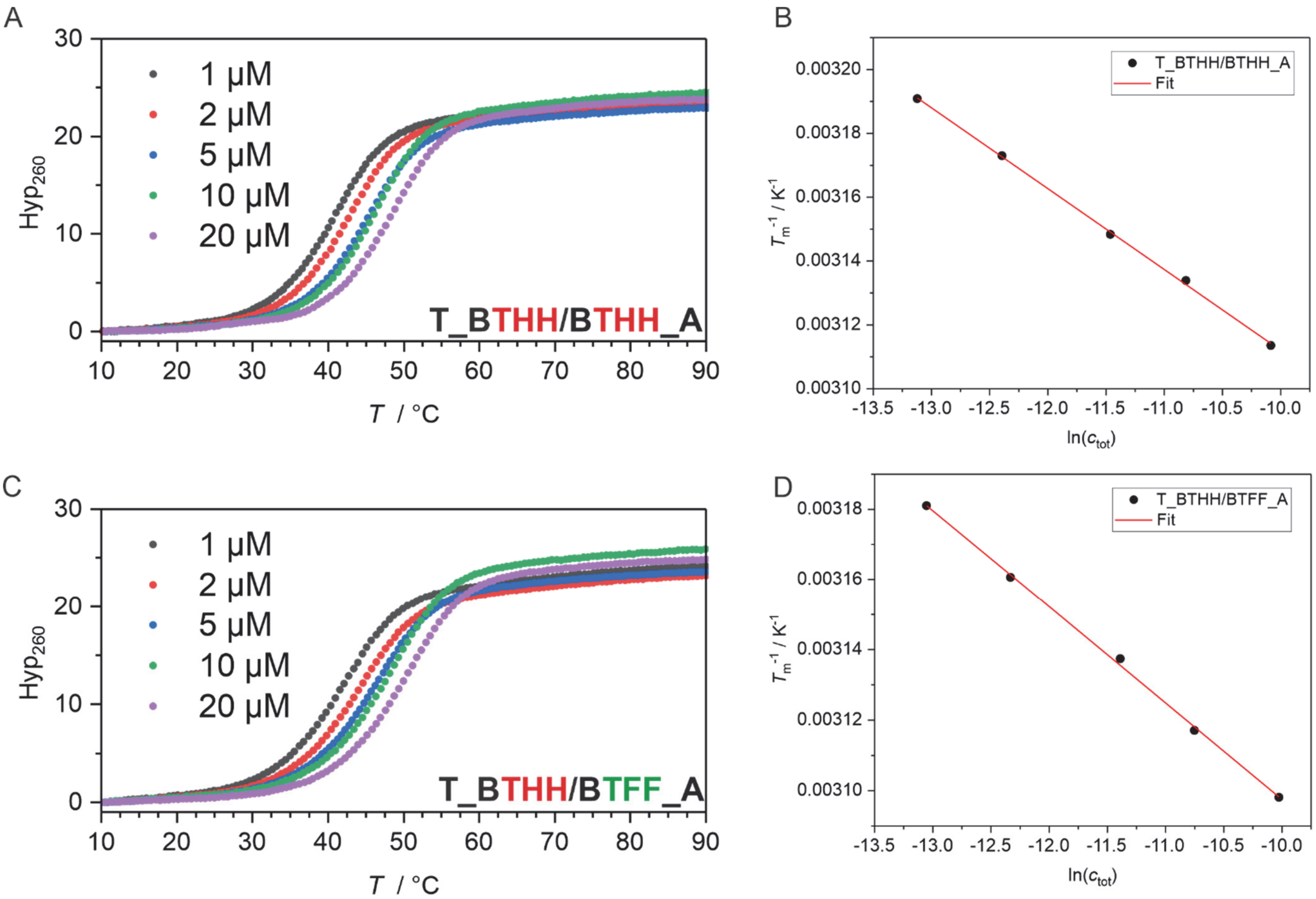

Figure S27 UV-thermal denaturation curves at different duplex concentrations in phosphate buffer (100 mM NaCl, 10 mM phosphate, pH7.0) and the corresponding van't Hoff plot for T_BTHH/BTHH_A (A and B) and T_BTHH/BTFF (C and D).

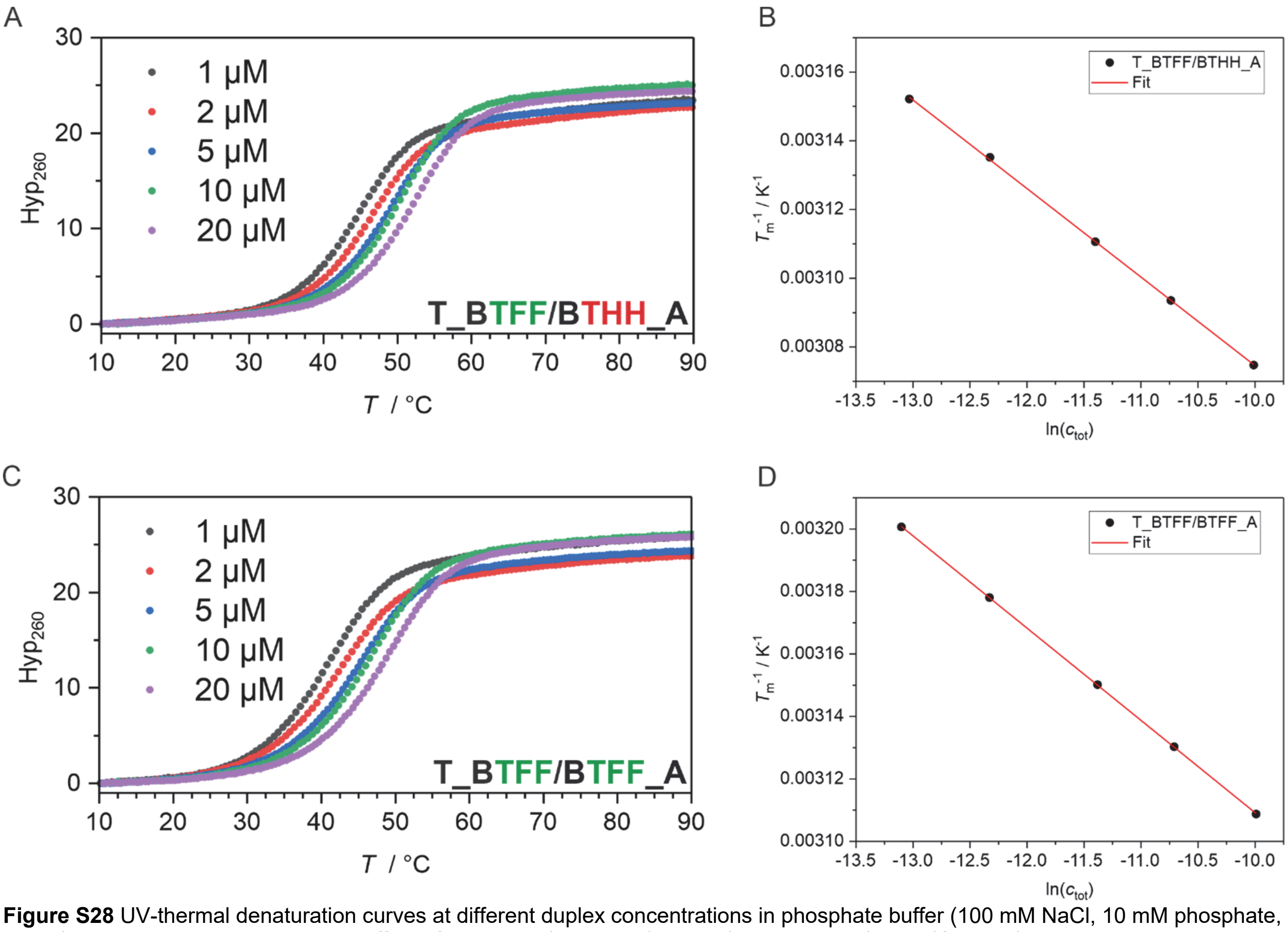

Figure S28 UV-thermal denaturation curves at different duplex concentrations in phosphate buffer (100 mM NaCl, 10 mM phosphate, pH7.0) and the corresponding van't Hoff plot for T_BTFF/BTHH_A (A and B) and T_BTFF/BTFF (C and D).

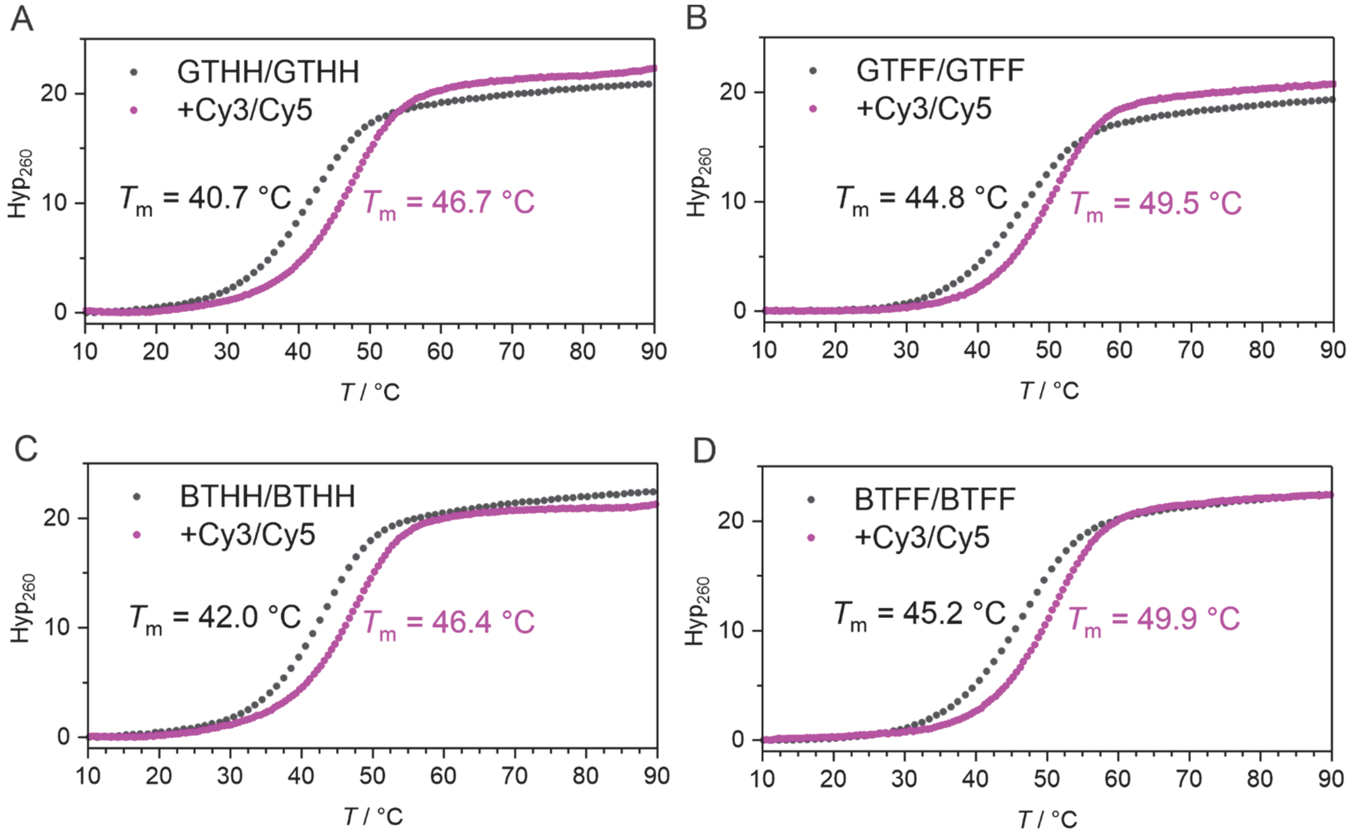

Figure S29 UV-thermal denaturation curves of homopairs with and without Cy3/Cy5 label of GTHH/GTHH (A), GTFF/GTFF (B), BTHH/BTHH (C) and BTFF/BTFF (D) at 1µM duplexes concentration in phosphate buffer (100 mM NaCl, 10 mM phosphate, pH7.0).

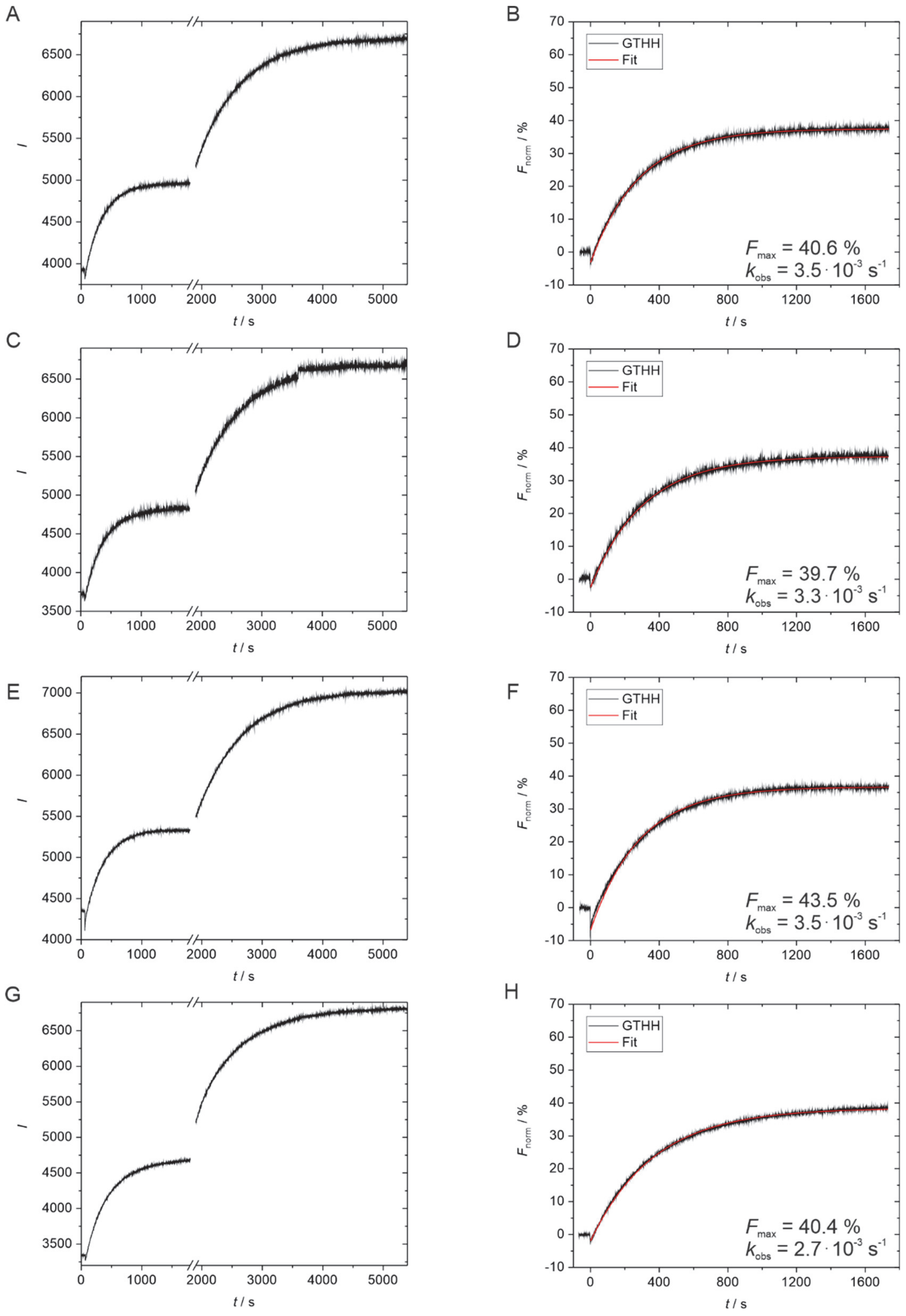

Figure S30 Replicates of the FRET exchange experiment with labeled GTHH/GTHH duplex and unlabeled GTHH single strand. A), C), E) and G) show the change in Cy3 fluorescence observed for the DNA duplex upon strand displacement. The corresponding normalized curves are shown in B), D), F) and H).

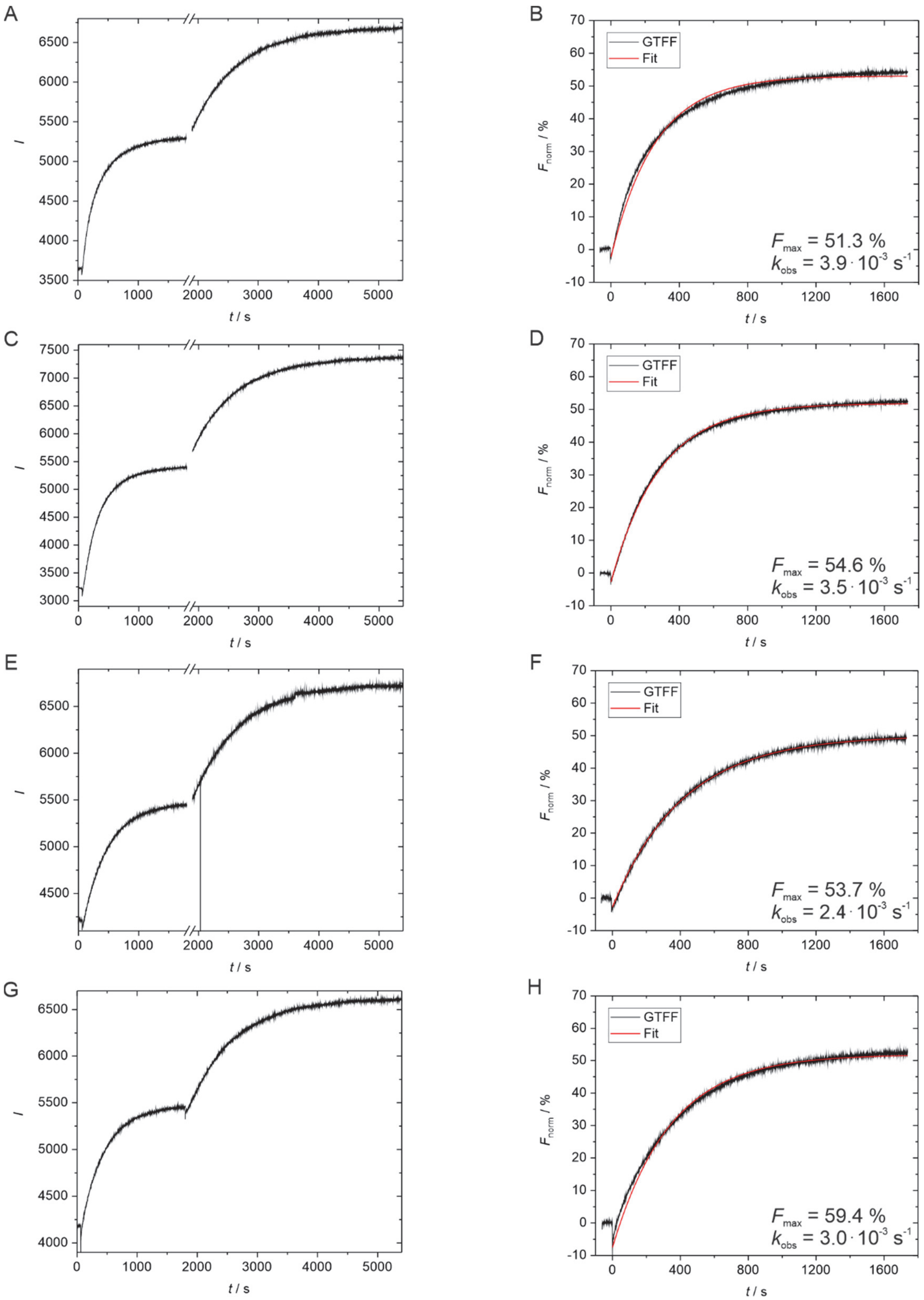

Figure S31 Replicates of the FRET exchange experiment with labeled GTHH/GTHH duplex and unlabeled GTFF single strand. A), C), E) and G) show the change in Cy3 fluorescence observed for the DNA duplex upon strand displacement. The corresponding normalized curves are shown in B), D), F) and H).

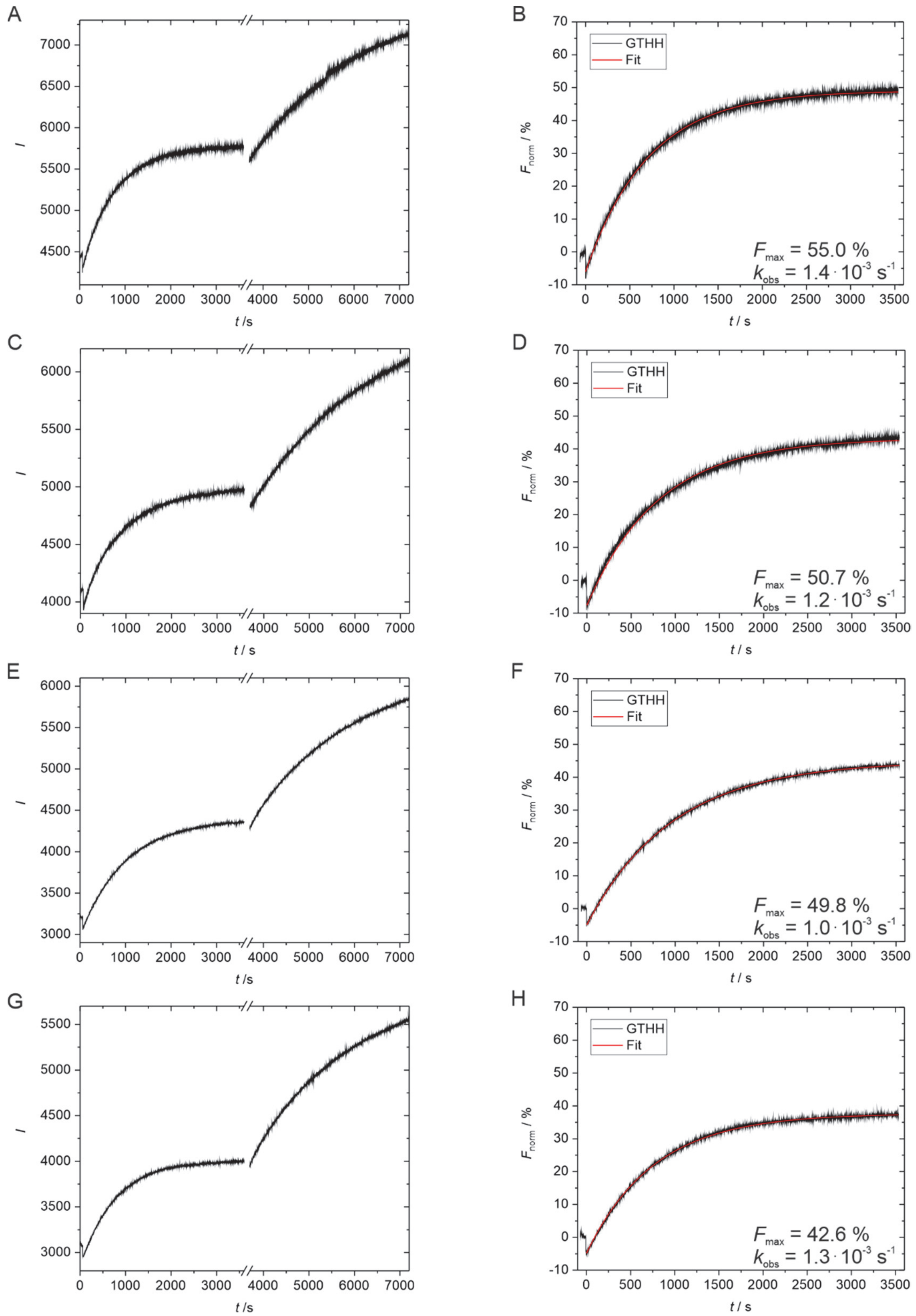

Figure S32 Replicates of the FRET exchange experiment with labeled GTFF/GTFF duplex and unlabeled GTHH single strand. A), C), E) and G) show the change in Cy3 fluorescence observed for the DNA duplex upon strand displacement. The corresponding normalized curves are shown in B), D), F) and H).

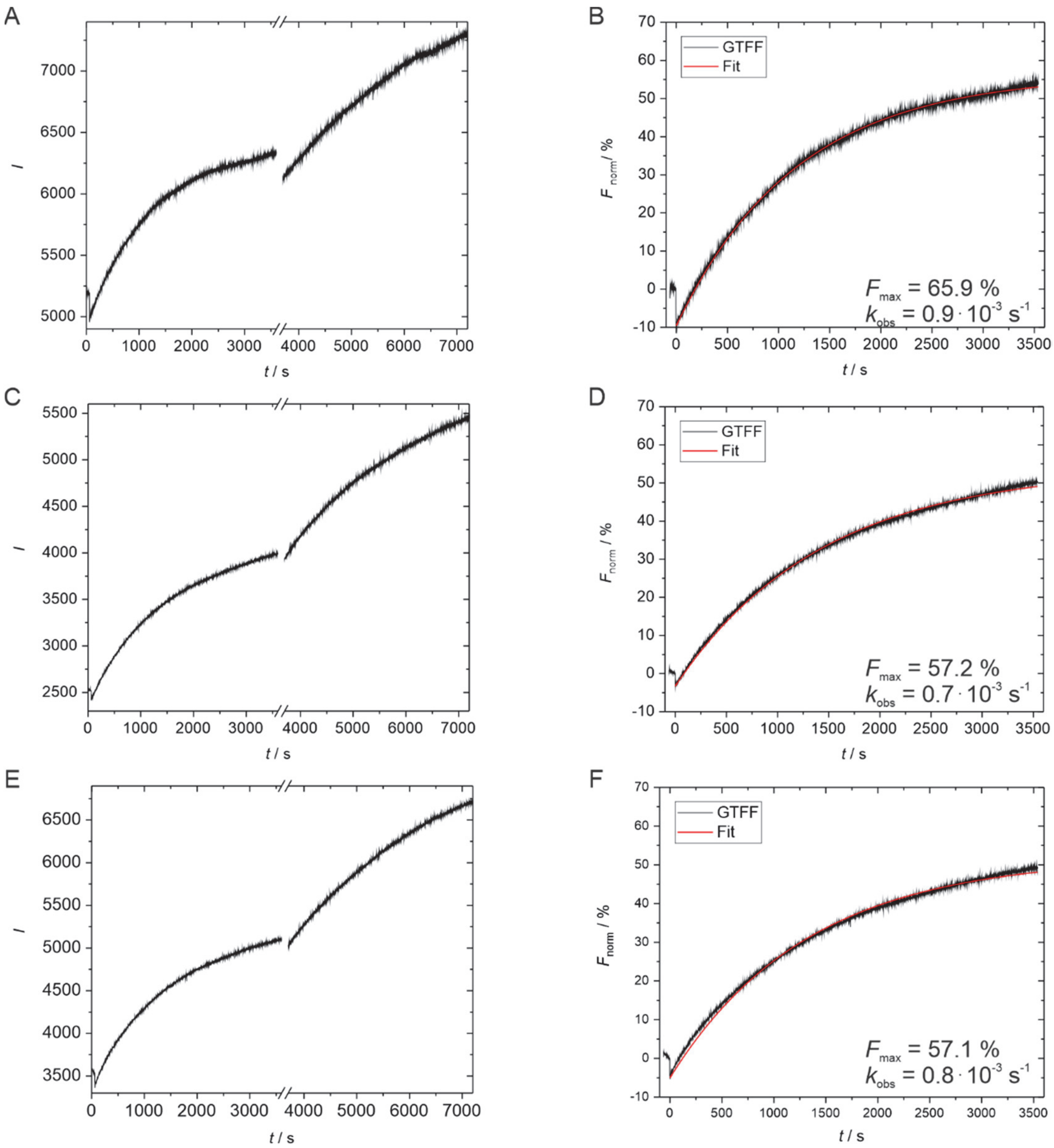

Figure S33 Replicates of the FRET exchange experiment with labeled GTFF/GTFF duplex and unlabeled GTFF single strand. A), C) and E) show the change in Cy3 fluorescence observed for the DNA duplex upon strand displacement. The corresponding normalized curves are shown in B), D) and F).

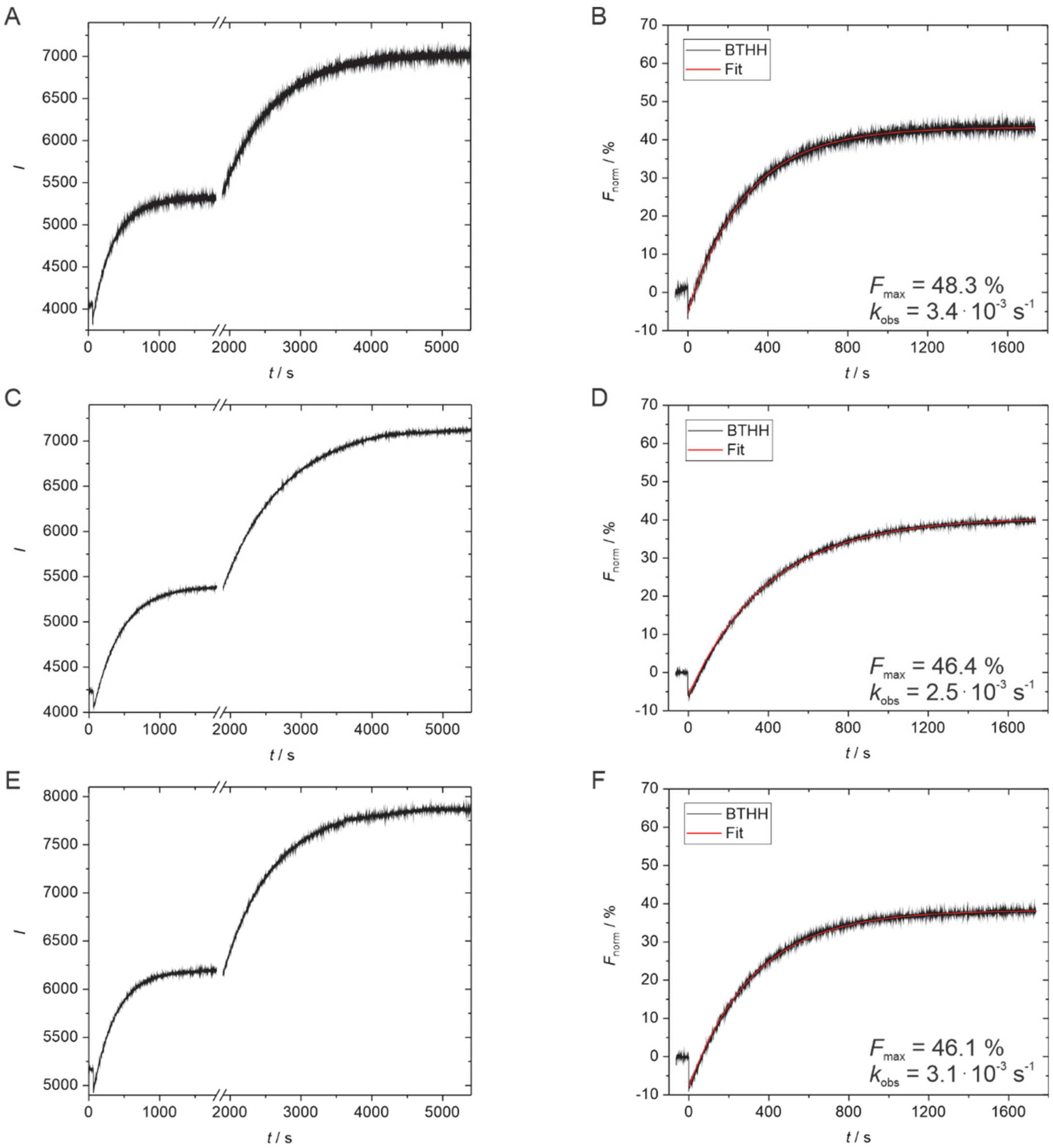

Figure S34 Replicates of the FRET exchange experiment with labeled BTHH/BTHH duplex and unlabeled BTHH single strand. A), C) and E) show the change in Cy3 fluorescence observed for the DNA duplex upon strand displacement. The corresponding normalized curves are shown in B), D) and F).

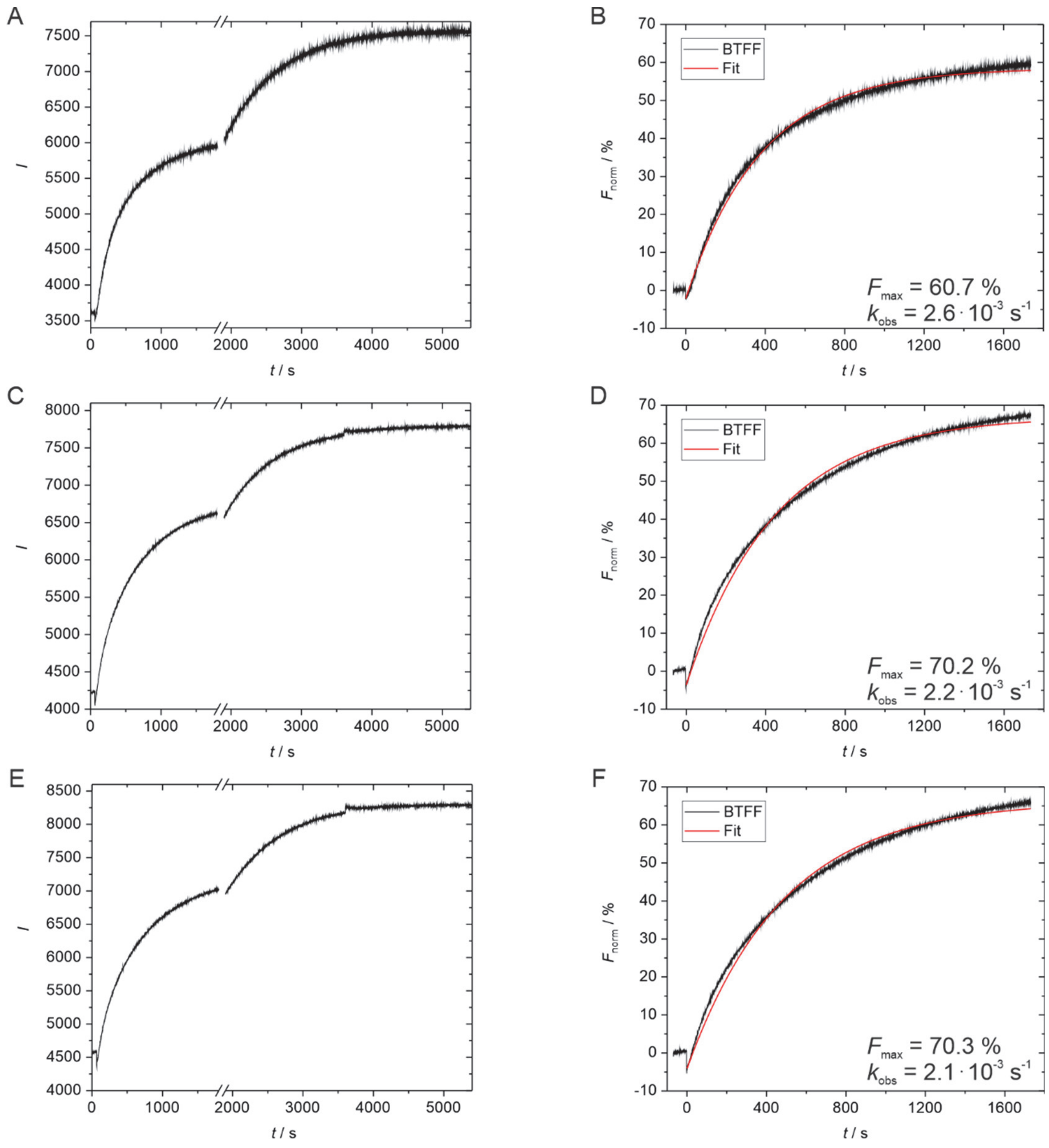

Figure S35 Replicates of the FRET exchange experiment with labeled BTHH/BTHH duplex and unlabeled BTFF single strand. A), C) and E) show the change in Cy3 fluorescence observed for the DNA duplex upon strand displacement. The corresponding normalized curves are shown in B), D) and F).

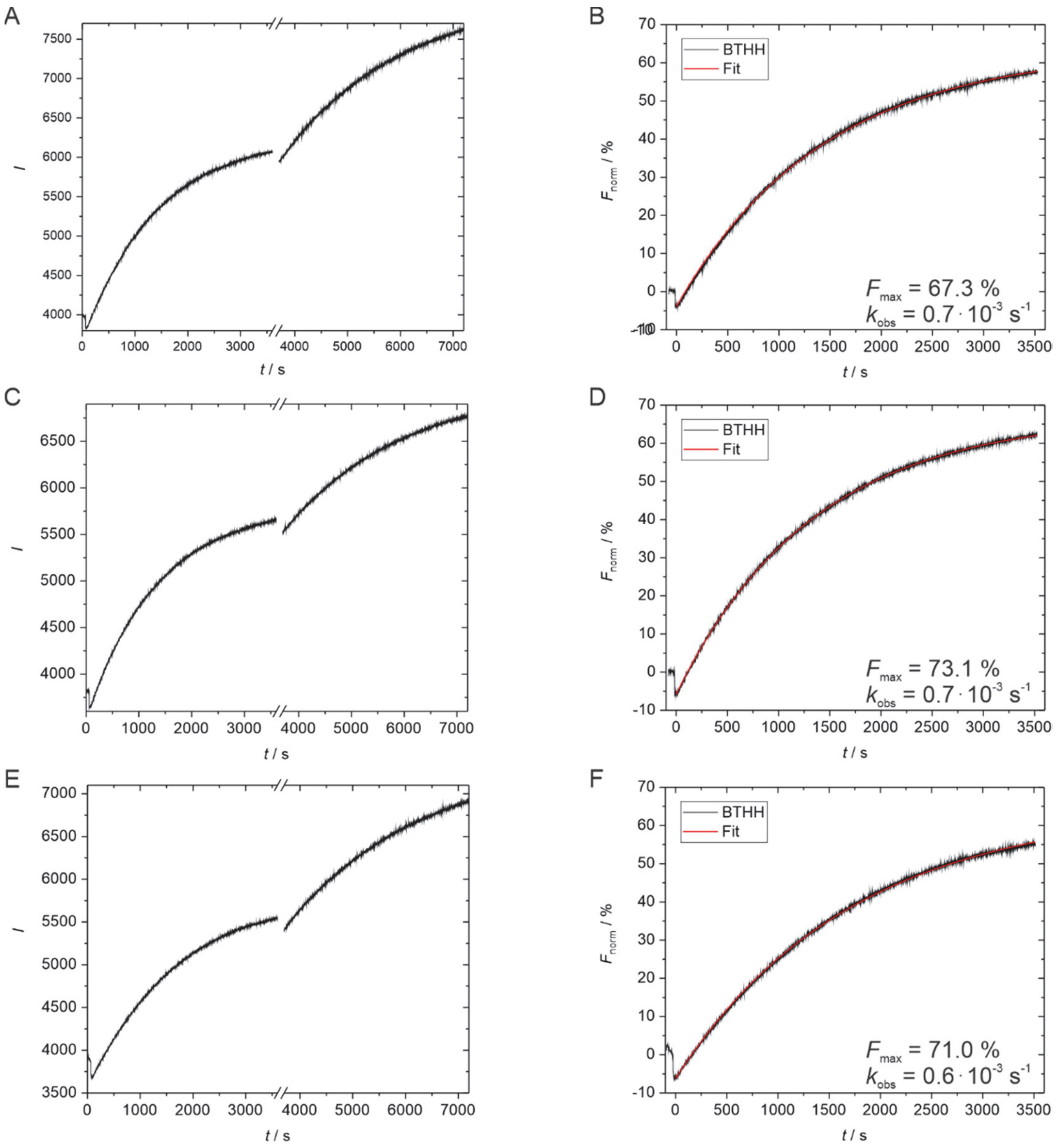

Figure S36 Replicates of the FRET exchange experiment with labeled BTFF/BTFF duplex and unlabeled BTHH single strand. A), C) and E) show the change in Cy3 fluorescence observed for the DNA duplex upon strand displacement. The corresponding normalized curves are shown in B), D) and F).

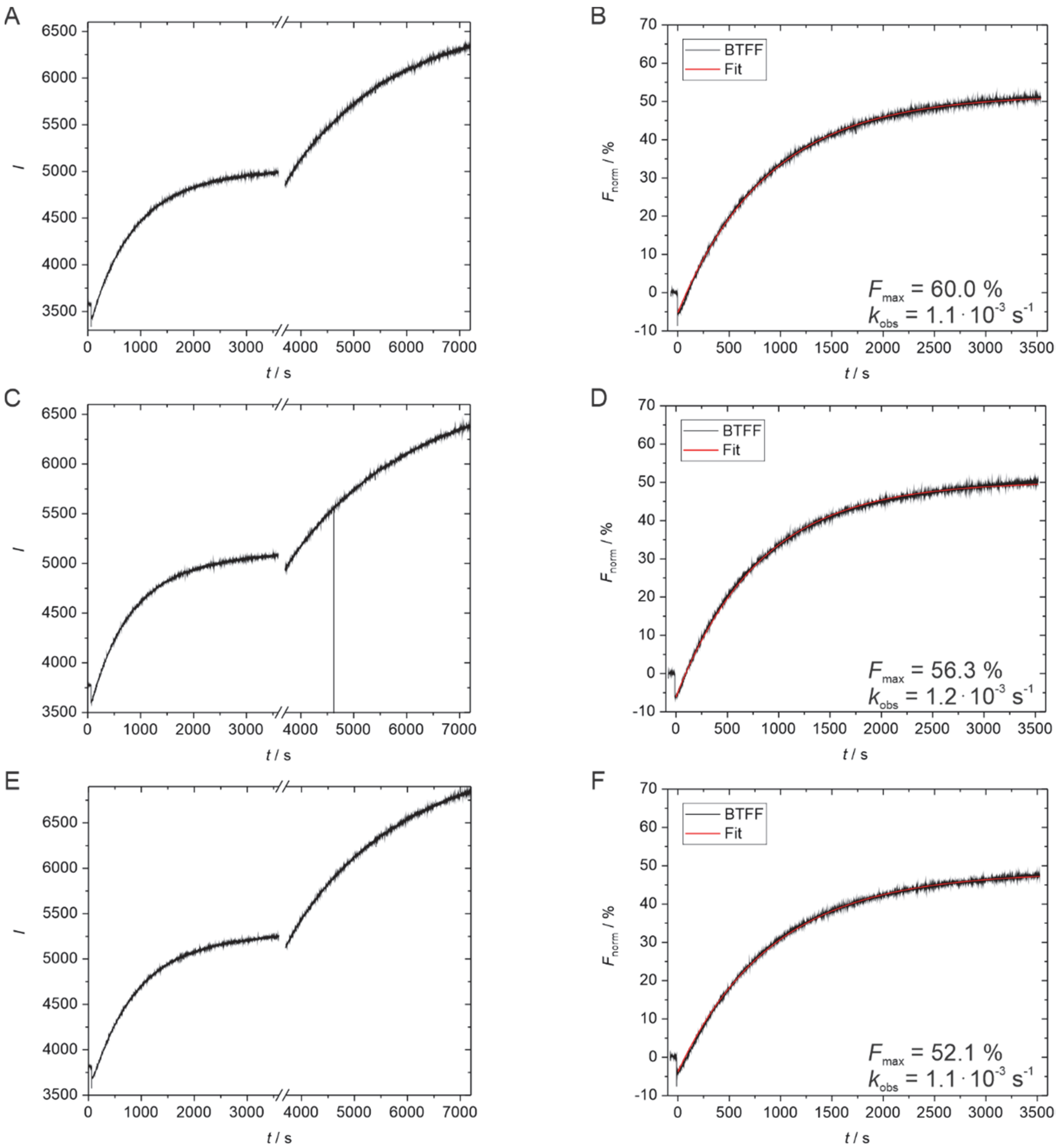

Figure S37 Replicates of the FRET exchange experiment with labeled BTFF/BTFF duplex and unlabeled BTFF single strand. A), C) and E) show the change in Cy3 fluorescence observed for the DNA duplex upon strand displacement. The corresponding normalized curves are shown in B), D) and F).

Cartesian coordinates of tolanes

THH

C	-1.759410	4.321276	-1.132375
C	-1.256967	3.080863	-0.751653
C	-2.118365	2.028645	-0.482118
C	-3.507204	2.201416	-0.588432
C	-4.003003	3.456338	-0.974737
C	-3.134340	4.502843	-1.243302
C	-4.393185	1.128296	-0.307974
C	-5.146792	0.214169	-0.068880
C	-6.027186	-0.861421	0.214140
C	-5.528749	-2.142028	0.514141
C	-6.382429	-3.188968	0.791553
C	-7.766866	-2.992734	0.779799
C	-8.280493	-1.729772	0.484283
C	-7.414515	-0.681144	0.205497
C	-9.935772	-3.942712	1.069103
O	-8.524408	-4.083263	1.065777
H	-1.083832	5.140634	-1.341397
H	-0.188025	2.932843	-0.664487
H	-1.729918	1.063504	-0.185790
H	-5.072450	3.595447	-1.059140
H	-3.531534	5.465231	-1.540122
H	-4.458808	-2.300667	0.524678
H	-6.001401	-4.174979	1.021608
H	-9.345389	-1.551841	0.468704
H	-7.816974	0.296809	-0.022322
H	-10.333788	-4.923031	1.319391
H	-10.261135	-3.215740	1.819032
H	-10.308062	-3.640015	0.086024

TFF

C	0.339944	2.854171	-1.040635
C	0.850815	1.572705	-0.794919
C	0.018545	0.513847	-0.495071
C	-1.372144	0.668566	-0.411248
C	-1.876577	1.947254	-0.666141
C	-1.048782	3.010247	-0.981925
C	-2.210303	-0.413719	-0.073037
C	-2.906978	-1.348105	0.232859
C	-3.697828	-2.451821	0.616329
C	-3.128725	-3.554399	1.267723
C	-3.891330	-4.640784	1.660468
C	-5.257292	-4.648895	1.405759
C	-5.850976	-3.571932	0.758939
C	-5.076457	-2.491551	0.372133
O	1.224510	3.814418	-1.355611
C	0.991643	5.165082	-0.923938
F	-1.608706	4.200468	-1.254596
F	-3.192757	2.158687	-0.619128
F	0.559328	-0.681846	-0.261334
F	2.170793	1.378123	-0.843937
F	-1.822112	-3.564618	1.525041
F	-3.324968	-5.674045	2.284253
F	-5.998572	-5.686968	1.781407
F	-7.161361	-3.586951	0.514709
F	-5.667482	-1.471151	-0.247921
H	1.962507	5.651164	-0.973159
H	0.285780	5.671083	-1.579698
H	0.622565	5.180972	0.102684

References

[1] N. Phetrak, T. Rukkijakan, J. Sirijaraensre, S. Prabpai, P. Kongsaeree, C. Klinchan, P. Chuawong, *J. Org. Chem.* **2013**, *78*, 12703-12709.
[2] S. Zhang, J. C. Chaput, *Curr. Protoc. Nucleic Acid Chem.* **2010**, *Chapter 4*, Unit4 40.
[3] O. Flogel, M. G. Okala Amombo, H. U. Reissig, G. Zahn, I. Brudgam, H. Hartl, *Chem. Eur. J.* **2003**, *9*, 1405-1415.
[4] S. D. Lepore, Y. He, *J. Org. Chem.* **2003**, *68*, 8261-8263.
[5] M. Poppe, C. Chen, F. Liu, S. Poppe, C. Tschierske, *Chem. Eur. J.* **2017**, *23*, 7196-7200.
[6] A. H. El-Sagheer, R. Kumar, S. Findlow, J. M. Werner, A. N. Lane, T. Brown, *ChemBioChem* **2008**, *9*, 50-52.
[7] K. Wawrzyniak-Turek, C. Höbartner, *Chem. Commun.* **2014**, *50*, 10937-10940.
[8] L. A. Marky, K. J. Breslauer, *Biopolymers* **1987**, *26*, 1601-1620.
[9] a) T. Maurer, H. R. Kalbitzer, *J. Magn. Reson. B* **1996**, *113*, 177-178; b)D. S. Wishart, C. G. Bigam, J. Yao, F. Abildgaard, H. J. Dyson, E. Oldfield, J. L. Markley, B. D. Sykes, *J. Biomol. NMR* **1995**, *6*, 135-140.
[10] T. D. Goddard, D. G. Kneller, *University of California, San Francisco.*
[11] W. Lee, M. Tonelli, J. L. Markley, *Bioinformatics* **2015**, *31*, 1325-1327.
[12] V. Sklenář, A. Bax, *J. Magn. Res.* **1987**, *74*, 469-479.
[13] T. L. Hwang, A. J. Shaka, *J. Magn. Reson., Series A* **1995**, *112*, 275-279.
[14] A. E. Derome, M. P. Williamson, *J. Magn. Reson.* **1990**, *88*, 177-185.
[15] A. J. Shaka, C. J. Lee, A. Pines, *J. Magn. Reson.* **1988**, *77*, 274-293.
[16] a) J. Jeener, B. H. Meier, P. Bachmann, R. R. Ernst, *J. Chem. Phys.* **1979**, *71*, 4546-4553; b) R. Wagner, S. Berger, *J. Magn. Reson. A* **1996**, *123*, 119-121.
[17] a) T. L. Hwang, S. Mori, A. J. Shaka, P. C. M. van Zijl, *J. Am. Chem. Soc.* **1997**, *119*, 6203-6204; b) T. L. Hwang, P. C. van Zijl, S. Mori, *J. Biomol. NMR* **1998**, *11*, 221-226; c)Y.-M. Lee, E.-H. Lee, Y.-J. Seo, Y.-M. Kang, J.-H. Ha, H.-E. Kim, J.-H. Lee, *Bull. Korean Chem.* **2009**, *30*, 2197-2198.
[18] a) E. Strebitzer, A. Rangadurai, R. Plangger, J. Kremser, M. A. Juen, M. Tollinger, H. M. Al-Hashimi, C. Kreutz, *Chem. Eur. J.* **2018**, *24*, 18903-18906; b) R. Bereiter, M. Himmelstoss, E. Renard, E. Mairhofer, M. Egger, K. Breuker, C. Kreutz, E. Ennifar, R. Micura, *Nucleic Acids Res.* **2021**, *49*, 4281-4293.
[19] M. W. Szulik, M. Voehler, M. P. Stone, *Curr. Protoc. Nucl. Acid Chem.* **2014**, *59*, 7 20 21-18.
[20] a) F. Neese, *Wiley Interdiscip. Rev. Comput. Mol. Sci.* **2012**, *2*, 73-78; b) F. Neese, *Wiley Interdiscip. Rev. Comput. Mol. Sci.* **2018**, *8*, e1327.
[21] a) S. Grimme, J. Antony, S. Ehrlich, H. Krieg, *J. Chem. Phys.* **2010**, *132*, 154104; b) S. Grimme, S. Ehrlich, L. Goerigk, *J. Comput. Chem.* **2011**, *32*, 1456-1465.
[22] a) A. Schäfer, H. Horn, R. Ahlrichs, *J. Chem. Phys.* **1992**, *97*, 2571-2577; b) F. Weigend, R. Ahlrichs, *Phys. Chem. Chem. Phys.* **2005**, *7*, 3297-3305.
[23] F. Weigend, *Phys. Chem. Chem. Phys.* **2006**, *8*, 1057-1065.
[24] M. Retegan, **2019**, "https://gist.github.com/mretegan/5501553".
